# JunB is a key regulator of multiple myeloma bone marrow angiogenesis

**DOI:** 10.1038/s41375-021-01271-9

**Published:** 2021-05-18

**Authors:** Fengjuan Fan, Stefano Malvestiti, Sonia Vallet, Judith Lind, Jose Manuel Garcia-Manteiga, Eugenio Morelli, Qinyue Jiang, Anja Seckinger, Dirk Hose, Hartmut Goldschmidt, Andreas Stadlbauer, Chunyan Sun, Heng Mei, Martin Pecherstorfer, Latifa Bakiri, Erwin F. Wagner, Giovanni Tonon, Martin Sattler, Yu Hu, Pierfrancesco Tassone, Dirk Jaeger, Klaus Podar

**Affiliations:** 1 Institute of Hematology, Union Hospital, Tongji Medical College, Huazhong University of Science and Technology, Wuhan, China; 2 Department of Medical Oncology, National Center for Tumor Diseases (NCT), University of Heidelberg, Heidelberg, Germany; 3 Department of Internal Medicine II, University Hospital Krems, Krems an der Donau, Austria; 4 Molecular Oncology and Hematology Unit, Karl Landsteiner University of Health Sciences, Krems an der Donau, Austria; 5 Center for Translational Genomics and Bioinformatics, IRCCS San Raffaele Scientific Institute, Milan, Italy; 6 Department of Experimental and Clinical Medicine, University “Magna Græcia” of Catanzaro, Catanzaro, Italy; 7 Department of Medicine, Harvard Medical School, Boston, MA USA; 8 University Hospital Heidelberg, Heidelberg, Germany; 9 Laboratory of Hematology and Immunology & Laboratory for Myeloma Research, Vrije Universiteit Brussel (VUB) Belgium, Brussels, Belgium; 10 Department of Neurosurgery, Friedrich-Alexander University (FAU) Erlangen-Nürnberg, Erlangen, Germany; 11 Institute of Medical Radiology, University Hospital St. Pölten, Karl Landsteiner University of Health Sciences, Krems an der Donau, Austria; 12 Genes & Disease Group, Department of Dermatology, Medical University of Vienna (MUW), Vienna, Austria; 13 Genes & Disease Group, Department of Laboratory Medicine, Medical University of Vienna (MUW), Vienna, Austria; 14 Functional Genomics of Cancer Unit, Experimental Oncology Division, IRCCS San Raffaele Scientific Institute, Milan, Italy; 15 Department of Surgery, Brigham and Women’s Hospital, Boston, MA USA

**Keywords:** Cell signalling, Myeloma

## Abstract

Bone marrow (BM) angiogenesis significantly influences disease progression in multiple myeloma (MM) patients and correlates with adverse prognosis. The present study shows a statistically significant correlation of the AP-1 family member JunB with VEGF, VEGFB, and IGF1 expression levels in MM. In contrast to the angiogenic master regulator Hif-1α, JunB protein levels were independent of hypoxia. Results in tumor-cell models that allow the induction of JunB knockdown or JunB activation, respectively, corroborated the functional role of JunB in the production and secretion of these angiogenic factors (AFs). Consequently, conditioned media derived from MM cells after JunB knockdown or JunB activation either inhibited or stimulated in vitro angiogenesis. The impact of JunB on MM BM angiogenesis was finally confirmed in a dynamic 3D model of the BM microenvironment, a xenograft mouse model as well as in patient-derived BM sections. In summary, in continuation of our previous study (Fan et al., 2017), the present report reveals for the first time that JunB is not only a mediator of MM cell survival, proliferation, and drug resistance, but also a promoter of AF transcription and consequently of MM BM angiogenesis. Our results thereby underscore worldwide efforts to target AP-1 transcription factors such as JunB as a promising strategy in MM therapy.

## Introduction

Multiple myeloma (MM), the second most common hematologic malignancy, is characterized by excess clonal proliferation of malignant plasma cells within the bone marrow (BM), renal disease, immunodeficiency, and osteolytic bone lesions. MM development involves early and late genetic changes and, even more importantly, selective conditions by the BM microenvironment such as BM neovascularization (“angiogenesis”) [1].

Enhanced microvessel density (MVD) [2], endothelial activity [3], capillary permeability, and perfusion [4] have been reported in the BM microenvironment of MM patients. Increasing BM angiogenesis parallels disease transition from Monoclonal Gammopathy of Unknown Significance (MGUS) to MM, correlates with MM progression and poor prognosis [2, 5, 6], and decreases with successful anti-MM treatment [6–9]. While MGUS and non-active MM represent “avascular” phases, active MM is associated with clonal tumor cell expansion and an “angiogenic switch” representing the “vascular” phase of plasma cell malignancies. The increase in MVD within the BM is triggered by oncogene-mediated expression and secretion of cytokines and pro-angiogenic growth factors. Our own and other previous studies have demonstrated that pro-angiogenic factors (AFs) VEGF, IGF1, bFGF, VEGFB, HGF, CTGF, TGFA, IL15, and ADM are expressed and secreted within the BM microenvironment [10, 11]. Similar to MVD, AF levels decrease during MM treatment [12, 13]. Importantly, based on the antiangiogenic activity of thalidomide and the discovery that MVD plays a key role in MM pathogenesis, thalidomide was used empirically to treat MM patients in the late 1990s. Remarkable clinical responses [14–16] rendered thalidomide to be the first antiangiogenic agent to enter tumor therapy and heralded the era of novel agents that revolutionized MM treatment strategies. Similar to thalidomide and its derivates, the immunomodulatory drugs (IMiDs) and also proteasome inhibitors (PIs) mediate anti-MM activity, at least in part, via inhibition of BM angiogenesis [17]. IMiDs and PIs now represent essential backbones in MM therapy; and IMiD/PI-containing treatment strategies significantly improve patient survival. Nevertheless, MM remains an incurable disease. The identification of novel targets and the development of derived efficient anti-MM agents are therefore needed.

Regulation of AF expression (e.g., VEGF) is a complex process involving a plethora of transcriptional regulators. The activator protein-1 (AP-1) transcription factor (TF) family member JunB is essential for mammalian placentation via the establishment of a proper feto-maternal circulatory system [18]. Moreover, preclinical studies have indicated a key role for JunB as a critical activator of AFs, VEGF in particular, in breast cancer, renal cell carcinoma, and teratocarcinoma [19, 20]. Our own studies demonstrated a critical role for JunB in MM cell proliferation, survival, and drug resistance [21]. However, the impact of JunB activity on BM angiogenesis, a hallmark of MM pathogenesis, has not been yet investigated. Here we show for the first time a role of JunB in MM BM angiogenesis and reveal a novel facet to the pathophysiologic functions of this TF in MM pathogenesis. These data thereby strongly support ongoing efforts to develop JunB-targeting agents for MM treatment to target multiple aspects of the disease and further enhance patient outcome.

## Materials and methods

### Reagents

Recombinant human interleukin-6 (IL6) protein was from R&D Systems (Minneapolis, MN, USA); the MEK1/2 inhibitor U0126, the NFκB inhibitor BAY 11-7085, dexamethasone, doxycycline, 4-hydroxytamoxifen (4-OHT), and DMSO were from Sigma (St Louis, MO, USA). Antibodies against JunB (C-11: Cat# sc-8051, RRID:AB_2130023) and extracellular signal-regulated kinase 2/ERK2 (D-2: Cat# sc-1647, RRID:AB_627547) were obtained from Santa Cruz Biotechnology (Heidelberg, Germany), and the antibody against Hif-1α (Cat# 565924, RRID:AB_2739388) from BD Biosciences (Heidelberg, Germany).

### Cell culture and transient transfection

MM cell lines (MM.1S, ATCC Cat# CRL-2974, RRID:CVCL_8792; RPMI 8226, CLS Cat# 300431/p771_RPMI_8226, RRID:CVCL_0014; U266, DSMZ Cat# ACC-9, RRID:CVCL_0566; KMS-11, JCRB Cat# JCRB1642, RRID:CVCL_4V71; MR20, RRID:CVCL_0509) were purchased from ATCC (Manassas, VA, USA) and DSMZ (Braunschweig, Germany). Human MM cell lines, stroma cell line KM-105, as well as primary bone marrow stroma cells (BMSCs) were cultured in RPMI-1640 medium supplemented with 10% or 20% heat-inactivated fetal bovine serum, 1% penicillin/streptomycin, and 2 mM L-glutamine (all from Gibco, Thermo Fisher Scientific Inc., Waltham, MA, USA). All experiments were conducted using cells that have undergone less than 20 passages after thawing. All cell lines were authenticated through short tandem repeat testing; and tested regularly for *Mycoplasma* negativity. For hypoxia, cells were incubated in a hypoxia chamber (COY Laboratory Products, Ann Arbor, MI, USA) with a computerized Oxygen controller to maintain an atmosphere of 0.5–1% O_2_, 5% CO_2_, and 37 °C.

Indicated cell lines were transiently transfected with indicated plasmids or a small interfering RNA (siRNA) SMARTpool for JunB and a non-targeting control (mock) siRNA (Dharmacon RNA Technologies, Lafayette, CO, USA), respectively, using the Nucleofactor 2b device together with the Cell Line Nucleofector Kit V Solution (Lonza Biosciences, Basel, Switzerland). Human umbilical vein endothelial cells (HUVECs, ATCC Cat# CRL-1730, RRID: CVCL_2959) were purchased from Lonza and maintained in EGM-2MV media (Lonza Biosciences, Basel, Switzerland) containing 2% fetal bovine serum. Tumor cell-stroma cell co-culture experiments were performed as previously described [21].

### Primary human BMSCs and MM cells

Primary human BMSCs were isolated from BM aspirates after informed consent was obtained in accordance with the Declaration of Helsinki. The collection and use of primary cells have been approved by the Ethics committee of the Medical Faculty, University of Heidelberg (approval number 229/2003 and S-152/2010) and by the Ethics committee of Lower Austria (approval number GS1-EK-4/407-2019). Isolation, purification, and culture of BMSCs and primary MM cells were performed as previously described [21].

### Retroviral/lentiviral constructs and transduction

For lentivirus-mediated inducible shRNA knockdown, MM cells were transiently or stably transduced with a pRSIT17-U6Tet-sh-CMV-TetRep-2A-TagGFP2-2A-Puro lentivirus, in which the JunB-specific shRNA sequence (shJunB #1 from RNAi Consortium shRNA Libraries [clone ID: TRCN0000014943], TRC/Broad Institute, Cambridge, MA, USA) or scramble control sequence was inserted resulting in TetR-shJunB and TetR-SCR (Cellecta/Biocat, Heidelberg, Germany), as described previously [21]. JunB-ER/MM.1S cells, which constitutively express a chimeric protein consisting of JunB fused with the hormone-binding domain of an estrogen receptor (ER), were generated, as previously described. MM.1S cells carrying the empty vector IRES-GFP served as a control [21, 22]. Transcriptional JunB activation was triggered by treatment with 4-OHT (100–200 nM).

### Cell lysis and western blot analysis

Whole cell lysates were prepared in RIPA lysis buffer (150 mM NaCl, 10 mM Tris pH7.2, 0.1% SDS, 1% Triton X-100, 1% Deoxycholate, 5 mM EDTA) supplied with Halt Protease and Phosphatase Inhibitor Cocktail (Pierce, Darmstadt, Germany). Western blot analysis was performed with indicated antibodies as previously described [21].

### Quantitative reverse transcription-polymerase chain reaction (RT-qPCR)

Cells were treated as indicated. RNA was extracted using TRIzol (Invitrogen, Carlsbad, CA, USA). cDNA samples were analyzed by quantitative reverse qPCR using QuantiFast SYBR Green PCR kit (Qiagen, Hilden, Germany), as previously described [21]. Primers used are shown in Supplementary Table 1.

### Enzyme-linked immunosorbent assay (ELISA)

VEGF, VEGFB, and IGF1 levels secreted by TetR-SCR/MM.1S and TetR-shJunB/MM.1S cells treated with doxycycline and stimulated with IL6 or secreted by JunB-ER/MM.1S cells treated with 4-OHT were quantified, as previously described [21, 23]. Soluble VEGF and IGF1 were detected with Quantikine ELISA kits (R&D Systems, Minneapolis, MN, USA), according to the manufacturer’s instructions. Soluble VEGFB was detected with a VEGFB ELISA kit (Mybiosource, San Diego, CA, USA), according to the manufacturer’s instructions.

### Gene expression profile

Comparative expression analyses for JunB and AF mRNA expression were performed in combined GSE5900 (healthy donors: *n* = 22; MGUS: *n* = 44) and GSE2658 (newly diagnosed [NDMM] patients: *n* = 559) datasets [24, 25]. Correlative expression analyses for JunB mRNA and AF mRNA expression in NDMM (*n* = 73) and relapsed/refractory [RRMM] patients (*n* = 28) were performed using the GSE6477 dataset [26]. Values were log2 transformed and scaled to the *Z*-score across genes in each group separately. A clustering of samples was applied (correlation distance, complete algorithm). Calculations and pictures were produced in R (R Project for Statistical Computing, RRID:SCR_001905; http://www.r-project.org/) and with the package pheatmap (pheatmap, RRID:SCR_016418). Gene set enrichment analysis (GSEA) was performed as previously described [27] (GSEA v2.0, RRID:SCR_003199; http://www.broad.mit.edu/gsea) using gene sets as permutation type and 1000 permutations and signal to noise as a metric for ranking genes.

### Clinical outcomes in MM to personal assessment of genetic profile (CoMMpass)

Use of the Multiple Myeloma Research Foundation (MMRF) CoMMpass data (release IA12-https://research.mmrf.org) was approved by the data access use committee and downloaded from dbGaP. Dataset is available at https://research.themmrf.org/ longitudinal, prospective observational study.

### Broad Institute Cancer Cell Line Encyclopedia (CCLE) analysis

mRNA levels of JunB and AFs in a series of MM cell lines were obtained from the CCLE website (https://portals.broadinstitute.org/ccle/), an online encyclopedia of a compilation of gene expression, chromosomal copy number, and massively parallel sequencing data from 947 human cancer cell lines [28, 29].

### Chromatin immunoprecipitation sequencing (ChIP-seq) analysis

ChIP was performed as previously described [30], and ChIP-seq (ChIP-seq, RRID:SCR_001237) was conducted by Igenebook (Wuhan, China). In brief, MM.1S cells cultured in T75 flasks were treated with 25 ng/ml IL6. After 24 h, MM cells were cross-linked with 1% formaldehyde for 10 min at room temperature, followed by quenching with glycine. An amount of 10 μl of monoclonal anti-JunB (C37F9) antibody (Cell Signaling Technology Cat# 3753, RRID: AB_2130002) was added to 10 μg of sheared chromatin (approximately 4 × 10^6^ cells) with dilution at 1:50 to immunoprecipitate JunB-DNA complexes. The ChIP DNA library concentrations were quantitated by Qubit 3.0 Fluorometer (Thermo Fisher Scientific Inc., Waltham, MA, USA) and the size of the fragments was examined by Qsep1 Bio-Fragment Analyzer (BiOptic). Libraries were sequenced using sequencing strategy PE150 on an Illumina HiSeq X Ten System (Illumina) to obtain 20 million reads per sample. Resulting fastq files of clean reads were aligned to human genome hg38_94 using BWA (version 0.7.15-r1140) to generate bam files (BWA, RRID:SCR_010910; http://bio-bwa.sourceforge.net/). Peaks were called using MACS (version 2.1.1.20160309) with *p* < 0.001 and visualized by Integrative Genomics Viewer (version 2.8.9, Broad Institute). Biological replicates were performed for the ChIP-seq and the genes in common were used for further analysis. Raw data are available upon request.

### Gene Ontology, motif analysis, and prediction of JunB-bound regions

The JunB target gene sets identified by ChIP-seq were used for Gene Ontology (GO) enrichment analysis using gene annotation and analysis resource Metascape (Metascape, RRID:SCR_016620; http://metascape.org/gp/index.html#/main/step1; version of database: updated on September 16, 2020, setting: Express Analysis) [31]. Motif analysis was performed with Homer (version 3) (http://homer.ucsd.edu/homer/motif/). TF binding profile open source databases and programs JASPAR (8th release in 2020) (JASPAR, RRID:SCR_003030; http://jaspar.genereg.net) [32] and PROMO (version 3.0.2, using version 8.3 of TRANScription FACtor (TRANSFAC) database) (ALGEN-PROMO, RRID:SCR_016926; http://alggen.lsi.upc.es/cgi-bin/promo_v3/promo/promoinit.cgi?dirDB=TF_8.3) [33, 34] were used to predict putative JunB binding sites in the promoter regions (2000 bp region upstream of transcription start site) of VEGF, VEGFB, and IGF1, as well as in the peak sequences from ChIP-seq results (Supplementary Table 2).

### Protein–protein interaction enrichment analysis

Protein–protein interaction enrichment analyses of JunB target genes were performed using STRING, version 11.0 (STRING, RRID:SCR_005223; https://string-db.org/) [35], which is a database of known and predicted protein–protein interactions including direct (physical) and indirect (functional) associations. The network was then analyzed and visualized using Cytoscape, version 1.6.1 (Cytoscape, RRID:SCR_003032; https://cytoscape.org/) [36], and the Molecular Complex Detection (MCODE) (version 1.5) [37] was applied to identify densely connected network components. KEGG (KEGG, RRID:SCR_012773; https://www.kegg.jp/) pathway and GO biological processes enrichment analysis was carried out using Metascape (Metascape, RRID:SCR_016620; version updated on September 16, 2020; http://metascape.org/gp/index.html#/main/step1).

### Wound-healing assay

Wound-healing assays were performed to assess cell migration. Briefly, 1 × 10^5^/ml HUVECs were seeded in 12-well plates. When 90% confluence was reached (about 48–36 h after seeding), a linear wound was created by scraping the cell mono-layer with a 200-μl tip. Cells were washed twice with PBS to remove cell detritus and cultured in conditioned medium (CM) at 37 °C and 5% CO_2_. Wound closure was measured by taking microscopic pictures at 10× magnification of five randomly selected fields every 3 h up to 12 h.

### In vitro angiogenesis assay

The antiangiogenic potential of CM derived from doxycycline-mediated JunB silencing in TetR-shJunB/MM was studied by using an in vitro angiogenesis assay (ECMatrix^TM^) kit (Chemicon, Temecula, CA, USA), as per the manufacturer’s instructions. Tube formation was assessed using an inverted light/fluorescence microscope at 4×–10× magnification. Photographs are representative of each group and three independent experiments.

### Cell viability assays

AlamarBlue assay was performed to assess the cell viability according to the manufacturer’s instructions (Thermo Fisher Scientific Inc., Waltham, MA, USA). All experiments were performed in triplicate or quadruplicate.

### In vivo studies

The 6- to 8-week-old Fox Chase nonobese diabetic/severe combined immunodeficient (NOD/SCID) female mice (Harlan Laboratories Inc., Indianapolis, IN, USA) were housed and monitored in the Animal Research Facility at Magna Græcia, Catanzaro, Italy. All experimental procedures and protocols have been approved by the institutional ethical committee at the Magna Græcia University, Catanzaro. Mice were subcutaneously inoculated with 5 × 10^6^ TetR-SCR/MM.1S or TetR-shJunB/MM.1S cells together with 1.5 × 10^6^ human-derived BMSCs and Matrigel (BD Biosciences, San Jose, CA, USA) in 100 μl of RPMI-1640 medium into the left and right flanks. The mice were randomized (8 mice/group), and induction of viral expression was obtained by addition of doxycycline to the drinking water. In accordance with the institutional guidelines, mice were killed when their tumors reached 1.5 cm in diameter, or in the event of paralysis or major compromise in their quality of life. Four-micrometer-thick sections of formalin-fixed tissue were used for staining with JunB (Cell Signaling Technology Cat# 3753, RRID:AB_2130002), CD31 (Novus Cat# NBP2-47785-0.1 mg, RRID:AB_2864381), and Ki67 (Abcam Cat# ab16667, RRID:AB_302459) antibodies, as well as with hematoxylin/eosin (HE, VECTOR Laboratories Burlingame, USA), in a humid chamber at room temperature. For image capturing, the Leica DM IRB microscope was connected to an Optimetrics camera and exported to MagnaFire software.

### 3D model and confocal microscopy

To generate the three-dimensional (3D) model, 1 × 10^5^ KM-105 stroma cells were seeded overnight onto poly-ε-caprolactone scaffolds (PCLS) (3D BioTek®, Bridgewater, NJ, USA) as described previously [38]. Based on the experimental condition, stroma-loaded scaffolds were cultured with or without 1 × 10^6^ GFP+ TetR-shJunB/MM.1S cells in the presence or absence of doxycycline for 4 h and then transferred in a Rotary Cell Culture System (RCCS^TM^) bioreactor (Synthecon Inc., Houston TX, USA). After a brief incubation, Fluorescent-tagged-HUVE cells (CellTracker™ Deep Red dye, Invitrogen, USA) were added into the rotating vessel and co-cultured for up to 72 h in RPMI-1640 medium plus 2% FBS. Scaffolds were then fixed in 4% PFA, stained with DAPI and imaged using confocal microscopy Leica TCS SP8 X (Vienna, Austria). Image processing and analysis was performed with FiJi ImageJ [39].

### Bone marrow biopsies of MM patients, immunohistochemical analysis, and quantification

BM biopsies were obtained from ten patients with NDMM. These processes were conducted in accordance with the Declaration of Helsinki, and studies were approved by the Ethics committee of Union Hospital, Tongji Medical College, Huazhong University of Science and Technology (approval number [2020] (0454-01)). Patient characteristics are summarized in Supplementary Table 3. Formalin-fixed paraffin-embedded sections from BM biopsies were cut at 3–5 μm and stained with HE. Immunohistochemical staining was performed using monoclonal antibodies against CD31 (EPR17259) (Abcam Cat# ab182981, Cambridge, MA, USA) and JunB (C37F9) (Cell Signaling Technology Cat# 3753, RRID:AB_2130002). The histological stainings were captured by a NanoZoomer S360 digital slide scanner with NDP.view2 software (Hamamatsu Photonics). For evaluation of CD31 MVD in BM, the average of counts from three to four high-power microscope fields (40×) of each section of each BM sample was used in the analysis. MVD was defined as the number of vessels per high-power field counted in the area of highest vascular density (called “hot spot”), as previously reported [40, 41]: low, ≤25 microvessels; medium, >25 microvessels but ≤50 microvessels; and high, >50 microvessels. To quantitatively analyze JunB staining, the average percentage of JunB-positive cells was determined using NIH ImageJ (1.52)/ImmunoRatio software (https://imagej.net/Welcome; ImageJ, RRID:SCR_003070) [42] on the same high-power microscope fields (40×). Statistical analysis was performed using one-way ANOVA analysis followed by Tukey’s multiple comparisons test with GraphPad Prism 7.04 Software (GraphPad Software, San Diego, CA, USA) (https://www.graphpad.com/; GraphPad Prism, RRID:SCR_002798). Data were expressed as mean ± SD. *p* < 0.05 was considered as significant.

### Statistical analysis

The Pearson correlation coefficient was used to measure linear relationship between JunB and AF mRNA expression levels among NDMM and RRMM in the GSE6477 dataset [26]; and among molecular risk groups of NDMM in GSE2658 [25] by IBM SPSS for Windows v 26 (https://www.ibm.com/uk-en/analytics/spss-statistics-software; SPSS, RRID:SCR_002865). The minimal level of significance was *p* < 0.05. The statistical significance of differences observed in treated versus control cultures was determined by means of an unpaired Student’s *t-*test. Data were presented as the mean ± SD from at least three independent experiments. Differences were considered statistically significant at *p* < 0.05. Comparison of JunB in cell lines with wildtype KRAS or NRAS versus in cell lines with mutated KRAS or NRAS was performed with one-way ANOVA (GraphPad).

## Results

### JunB expression correlates with expression profiles of angiogenic factors in MM cells

To investigate whether JunB affects production and secretion of AFs by MM cells and thereby BM angiogenesis, we first performed a comparative supervised analysis of JunB and AFs in healthy donors versus MGUS and MM samples utilizing publicly available datasets GSE5900 and GSE2658 (*n* = 625) [24, 25]. Our analysis shows a gradual increase of mRNA levels of AFs VEGF, VEGFB, IGF1, PIGF, HGF, CTGF, TGFB1, IL6, and IL15, along with mRNA levels of JunB from healthy donors to MGUS and MM (Supplementary Fig. 1).

**Fig. 1:**
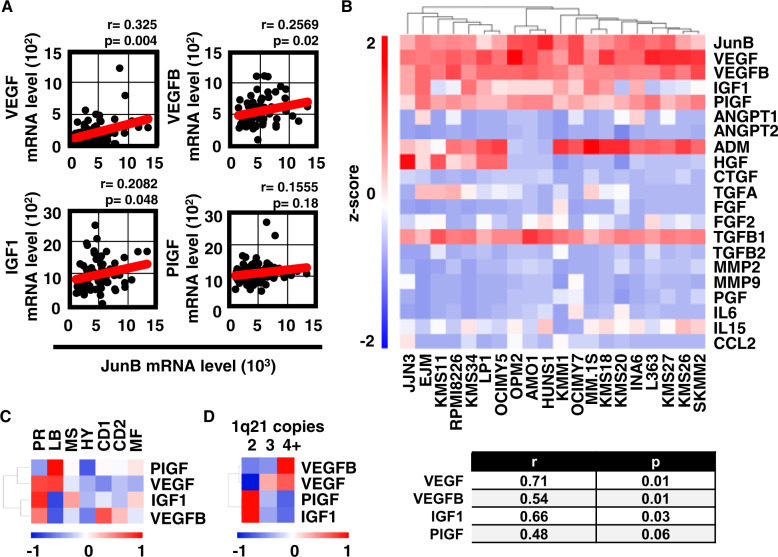
JunB expression correlates with expression profiles of angiogenic factors in MM cell line and primary cells. **A** Correlation of JunB and angiogenic factor (AF) expression in primary samples of NDMM patients. Data analyses of samples within the GSE6477 dataset of NDMM patients indicate a statistically significant association for JunB with VEGF, VEGFB, and IGF1 but not with PIGF [26]. The Pearson correlation coefficient was calculated to evaluate the correlation between JunB and indicated AFs. The minimal level of significance was *p* < 0.05. **B** Heatmap of mRNA expression values (log2 and *Z*-score scaled across genes) for the indicated genes in MM cell line cells was obtained from data extracted from the Cancer Cell Line Encyclopedia (CCLE) database. The Pearson correlation coefficient was calculated to evaluate the correlation between JunB and indicated AFs. The minimal level of significance was *p* < 0.05. **C** Correlation of JunB and AF expression differs in the GSE2658 dataset of NDMM patients dependent on the molecular risk group. Heatmap of *r* values calculated on samples with known molecular information obtained from the GSE2658 dataset [26]. HYperdiploid (HY) group 4, Low Bone disease (LB) group 2, CD1 group 5, CD2 group 6, PRoliferation (PR) group 1, MMSET (MS) group 3, MAF/MAFB (MF) group 7. **D** Correlation of JunB and AF expression differs dependent on the number of 1q21 copies. Heatmap of *r* values calculated on samples with known molecular information obtained from the GSE2658 dataset [24] of NDMM patients.

Correlation scores between JunB and Hif-1α, and AFs were determined next with the aim to identify independent and also potentially redundant angiogenic roles of these TFs within the BM microenvironment. Indeed, besides the cellular and extracellular compartments within the humoral milieu, the complexity of the BM architecture is based on highly heterogenous O_2_ levels, with the endosteal niche representing the most hypoxic, and the vascular niche representing the best oxygenated region [43]. MM cells predominantly adapt to hypoxia via the hypoxia-inducing TF Hif-1α, a critical regulator of genes contributing to epithelial-mesenchymal transition, motility, immune evasion, drug resistance, metabolic reprogramming, metastasis, and maybe most importantly to tumor-associated angiogenesis [44]. Hypoxia and Hif-1α levels increase in the BM of MM patients during disease progression due to rapidly proliferating tumor cells and promote MM cell dissemination through acquisition of epithelial to mesenchymal transition-like features, bone destruction, and MM BM angiogenesis [45–50]. Utilizing the uniform GSE6477 dataset of NDMM patients, a significant positive Pearson correlation score was calculated for JunB expression and VEGF, VEGFB, and IGF1; but not for JunB and PIGF (Fig. 1A), CCL2, MMP9, IL8, IL15, IGF2BP2, IGF2BP3, ADM, PGF, CTGF, TGFA, HGF, and TGFB1 (Supplementary Fig. 2A, B). In contrast to JunB, expression levels of Hif-1α did not correlate with VEGF, VEGFB, and IGF1 (Supplementary Fig. 3A), but with CCL2, IL8, and IGF2BP3 (Supplementary Fig. 3B). For RRMM patients in the same dataset, we did not observe significant correlations between expression levels of JunB (Supplementary Fig. 4A, B) or Hif-1α (Supplementary Fig. 5) and VEGF, VEGFB, and IGF1. However, JunB expression levels also did not correlate with other AFs investigated in this patient population (Supplementary Fig. 4B), whereas Hif-1α expression levels strongly correlated with CCL2, MMP9, IGF2BP2, and IGF2BP3 expression levels (Supplementary Fig. 5). In agreement with these data, JunB also did not correlate with Hif-1α expression levels, neither in samples derived from NDMM nor from RRMM patients (Supplementary Fig. 6). In addition, no statistically significant correlation score was obtained for AP-1 TFs cJun and cFos with VEGF, VEGFB, or IGF1 (Supplementary Fig. 7). Further supporting a role for JunB in signaling pathways of AFs in MM cells, Gene Set Enrichment Analysis (GSEA) (https://www.gsea-msigdb.org/gsea/index.jsp) [27] of JunB low versus JunB high quartiles identified a positive correlation of JunB and groups of genes within the VEGF- and the IGF1-signaling pathways with a Normalized Enrichment Score of 1.83 (*p* = 0.01) and 1.54 (*p* = 0.007) and a False Discovery Rate *q* value of 0.04 and 0.08, respectively (Supplementary Fig. 8). Similar to primary tumor cells, a significant positive Pearson correlation score was also observed for JunB and VEGF, VEGFB, and IGF1 in a selected number of MM cell lines deposited in the CCLE database [28] (Fig. 1B). The correlation of JunB expression with PIGF, ADM, and TGFB1 was not significant in this dataset (data not shown). Taken together, these results suggest a regulatory role of JunB upon AFs VEGF, VEGFB, and IGF1 in MM cells and support the existence of two independent JunB- and Hif-1α-mediated transcriptional programs for angiogenesis.

### Correlation of JunB and angiogenic factor expression differs in molecular risk groups

While being defined by the same histology, MM is a highly heterogenous and genomically complex disease. Predominantly driven by recurrent translocations and hyperdiploidy, MM transcriptoms belong to seven distinct molecular groups. These subgroups are strongly influenced by cMAF, MAFB, CCND1, and CCND3 and MMSET-activating translocations and hyperdiploidy. Specifically, the HYperdiploid (HY) group 4, the Low Bone disease (LB) group 2, the CD1 group 5, and the CD2 group 6 are associated with good prognosis (low risk groups), while the PRoliferation (PR) group 1, the MMSET (MS) group 3, and the MAF/MAFB (MF) group 7 are associated with poor prognosis (high risk groups). In the clinical context, patients from the MS and PR groups are less likely to benefit from autologous stem cell transplantation [25]. Moreover, gains of 1q21 have been associated with an adverse effect on overall survival. Of note, MM patients with four or more copies of 1q have a less favorable prognosis than those with three 1q copies [51–53].

We therefore evaluated whether the correlation between JunB and AFs differs among these molecular risk groups. Our analysis demonstrates a positive correlation of gene expression for JunB and VEGF in molecular groups PR (*r* = 0.37, *p* = 0.01) and LB (*r* = 0.38, *p* = 0.003); for JunB and IGF1 in molecular groups PR (*r* = 0.39, *p* = 0.006) and MS (*r* = 0.27, *p* = 0.02); and JunB and VEGFB in molecular groups PR (*r* = 0.4, *p* = 0.005), CD1 (*r* = 0.45, *p* = 0.01) and CD2 (*r* = 0.32, *p* = 0.01) (Fig. 1C). While we have not observed a positive correlation of JunB and PIGF across MM samples (Fig. 1A), a significant positive Pearson correlation score was calculated for JunB and PIGF gene expression, in the molecular group LB (*r* = 0.38, *p* = 0.01) (Fig. 1C). Moreover, a statistically significant positive correlation of JunB with VEGF was observed in MM patients harboring 3 (*r* = 0.28, *p* = 0.01) and 4+ (*r* = 0.34, *p* = 0.02) copies of 1q21, and of JunB with PIGF in patients harboring 2 copies of 1q21 (*r* = 0.2, *p* = 0.01). Positive *r* scores of JunB with VEGFB or IGF1, respectively, were not statistically significant (Fig. 1D). Of note, JunB expression levels were similar in all MM patient groups including those with 2, 3, or 4+ 1q21 copies (Supplementary Fig. 9). Ongoing studies seek to identify molecular mechanisms by which JunB expression is correlated with AF levels in 1q21 patients.

Taken together, these data indicate that the correlative expression of JunB with distinct AFs may be linked to the molecular background of tumor cells.

### IL6 but not hypoxia is inducing JunB-dependent production and secretion of angiogenic factors

**Fig. 2:**
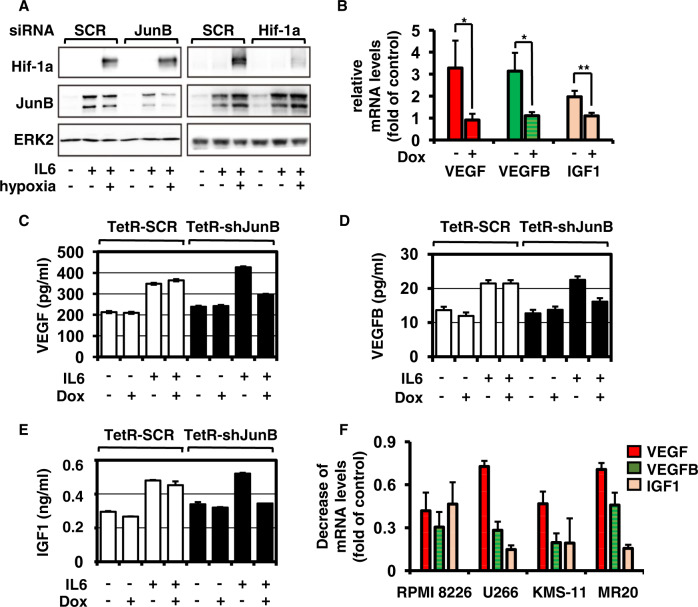
IL6 but not hypoxia is inducing JunB-dependent production and secretion of angiogenic factors. **A** Impact of hypoxia on JunB *versus* Hif-1α expression. MM.1S cells were transiently transfected with non-targeting control (SCR) siRNA, siJunB, or siHif-1α and then treated with IL6 (25 ng/ml) and 1% O_2_ (hypoxia) or left untreated. After 9 h, cell lysates were immunoblotted with antibodies against Hif-1α and JunB. ERK2 served as a loading control. **B** Doxycycline (Dox)-mediated JunB knockdown in TetR-shJunB/MM.1S inhibits IL6-induced production of AFs. TetR-shJunB/MM cells were cultured in RPMI-1640 medium with IL6 in the presence or absence of doxycycline (1 μg/ml). Expression profiles of VEGF, VEGFB, and IGF1 in TetR-shJunB/MM cells were determined using RT-qPCR. Data represent mean ± SD for triplicate samples of three independent experiments. *p < 0.05; **p < 0.01. **C**–**E** Doxycycline-mediated JunB knockdown in TetR-shJunB/MM.1S inhibits IL6-induced secretion of AFs. TetR-SCR/MM.1S or TetR-shJunB/MM.1S cells were cultured in RPMI-1640 medium with or without IL6 in the presence or absence of doxycycline (1 μg/ml). Supernatants from equal numbers of cells (1.2 × 10^6^) were collected after 18 h and analyzed for VEGF (**C**), VEGFB (**D**), and IGF1 (**E**) protein levels by ELISA. Supernatants from IL6-stimulated TetR-SCR/MM.1S cells served as a control. Data are expressed a mean ± SD of culture triplicates. **F** siRNA-mediated JunB knockdown in RPMI 8226, U266, KMS-11, and MR20 downregulates IL6-induced production of VEGF, VEGFB, and IGF1. MM.1S cells were transiently transfected with mock (200 nM) and JunB siRNA (200 nM). Expression profiles of VEGF, VEGFB, and IGF1 in indicated MM cell lines were determined using RT-qPCR. Data represent the decrease of VEGF, VEGFB, and IGF1 expression levels in MM cells transfected with siJunB versus mock control and represent mean ± SD for triplicate samples of three independent experiments.

Based on our results indicating that JunB and Hif-1α induce different transcriptional programs, we next investigated the functional impact of hypoxia on JunB versus Hif-1α protein levels within the BM microenvironment. Importantly, our results demonstrate that Hif-1α knockdown does not change IL6-induced JunB upregulation, neither under normoxic nor under hypoxic conditions. Conversely, hypoxia increases Hif-1α levels, even after JunB knockdown (Fig. 2A).

While these results exclude a functional impact of hypoxia on JunB expression levels, our previous studies have demonstrated that JunB upregulation in MM cells is predominantly mediated via cytokines secreted by BMSCs, IL6 in particular [21]. Having observed a positive correlation of JunB with AF mRNA levels, we next investigated whether JunB regulates the expression of AFs within the MM BM microenvironment using a doxycycline-inducible shRNA strategy. Specifically, we utilized TetR-shJunB/MM.1S cells [21, 22], in which tetracycline can induce JunB knockdown. VEGF, VEGFB, and IGF1 mRNA levels were significantly decreased in doxycycline-treated versus untreated TetR-shJunB/MM cells co-cultured with BMSCs (data not shown). Moreover, doxycycline-induced JunB silencing in TetR-shJunB/MM.1S cells potently reduced IL6-triggered expression (Fig. 2B) and secretion of VEGF, VEGFB, and IGF1 (Fig. 2C–E), as determined by RT-qPCR and ELISA, respectively. In contrast, expression levels of other AFs including ADM, TGFB1, and FGF remained unchanged (data not shown). Consistently, a significant decrease of VEGF, VEGFB, and IGF1 expression was also observed upon transient knockdown of JunB in four additional IL6-stimulated MM cell lines RPMI 8226, U266, KMS-11, and MR20 (Fig. 2F).

Taken together, these results demonstrate that JunB and Hif-1α expression levels are functionally regulated by distinct features within the BM microenvironment, the cellular compartment and liquid milieu, and hypoxia, respectively. Moreover, they demonstrate that JunB is mediating production and secretion of AFs VEGF, VEGFB, and IGF1.

### JunB increases production and secretion of angiogenic factors in a MEK/MAPK- and NFκB-dependent, but Ras-independent manner

**Fig. 3:**
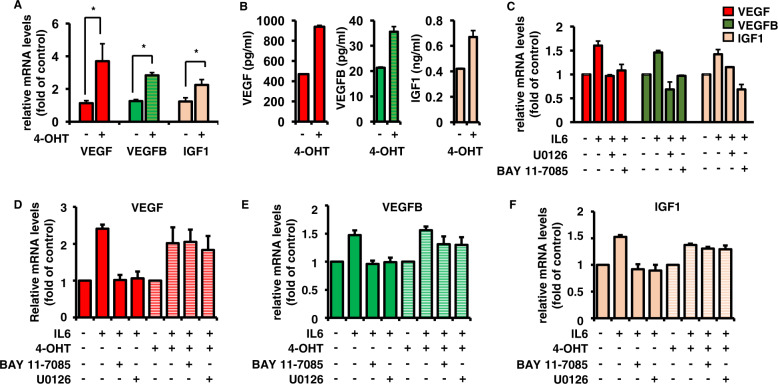
4-OHT-induced JunB activity in JunB-ER/MM.1S cells increases production and secretion of angiogenic factors. **A** 4-OHT-induced JunB activation in JunB-ER/MM.1S cells induces expression of VEGF, VEGFB, and IGF1. JunB-ER/MM.1S cells were cultured in RPMI-1640 medium and treated without or with 4-OHT (100 nM) for 16 h. Expression profiles of VEGF, VEGFB, and IGF1 in JunB-ER/MM.1S cells were determined using RT-qPCR. Data represent mean ± SD for triplicate samples of three independent experiments. *p < 0.01. **B** 4-OHT-induced JunB activity enhances the secretion of VEGF, VEGFB, and IGF1 in JunB-ER/MM.1S cells. JunB-ER/MM.1S cells were treated without or with 4-OHT, as indicated. Supernatants from equal numbers of cells (1.2 × 10^6^) were collected after 48 h and analyzed for protein levels of VEGF, VEGFB, and IGF1 by ELISA. Data represent mean ± SD for triplicate samples of three independent experiments. **C** IL6-induced expression of VEGF, VEGFB, and IGF1 is MEK/MAPK- and NFκB-dependent. MM.1S cells were pretreated with U0126 and BAY 11-7085 or left untreated, and then cultured in RPMI-1640 medium with or without IL6. Expression profiles of VEGF, VEGFB, and IGF1 in MM.1S cells were determined by RT-qPCR with B2M as an endogenous control. Data represent mean ± SD for triplicate samples of three independent experiments. **D**–**F** 4-OHT-induced JunB activation rescues BAY 11-7085- and U0126-mediated inhibition of VEGF, VEGFB, and IGF1 mRNA levels. JunB-ER/MM.1S cells were cultured alone or with IL6 for 4 h with or without U0126 and BAY 11-7085; and 4-OHT (200 nM) for an additional 2 h. Cells were then harvested, lysed, and expression levels of VEGF (**D**), VEGFB (**E**), and IGF1 (**F**) were determined by RT-qPCR with B2M as an endogenous control. Each value is shown as mean ± SD of three independent experiments.

To further support a functional role of JunB in AF generation and secretion and thereby in MM BM angiogenesis, we utilized JunB-ER/MM.1S cells [21, 22], which constitutively express a chimeric protein consisting of JunB fused with the hormone-binding domain of an ER. JunB-ER/MM.1S cells treated with 4-OHT versus untreated control induced expression of VEGF, VEGFB, and IGF1, as evidenced by RT-qPCR (Fig. 3A). Consequently, a significant increase of VEGF, VEGFB, and IGF1 secretion was measured upon 4-OHT-induced JunB activation (Fig. 3B). We previously demonstrated that IL6-triggered JunB upregulation is MEK/MAPK- and NFκB-dependent, but PI3K/Akt-independent [21]. Consistent with these data, the MEK inhibitor U0126 and the NFκB inhibitor BAY 11-7085 downregulated IL6-triggered expression of VEGF, VEGFB, and IGF1 in MM cells (Fig. 3C). Conversely, 4-OHT-induced JunB activation rescued the suppression of VEGF, VEGFB, and IGF1 generation by BAY 11-7085 and U0126, respectively, further confirming a key role for JunB in AF production (Fig. 3D–F).

Activating mutations of the K- or N-Ras oncogene are present in up to 50% of MM patients; with an increasing prevalence through disease progression and development of plasma cell leukemia. The majority of Ras mutations involve N-Ras, most often at codon 61 [54–57]. Given that JunB and its target genes are under the control of MEK/ERK, we investigated next whether JunB is an actionable target in Ras-mutated MM patients. Our results show similar JunB expression levels in Ras wildtype and K/N-Ras-mutated primary MM cells and cell line cells (Supplementary Fig. 10). Moreover, siJunB-induced inhibition of AFs VEGF, VEGFB, and IGF1 was similar in MM cell lines harboring Ras mutations (RPMI 8226, MR20) versus MM cell lines harboring Ras wildtype (U266, KMS-11) (Fig. 2F).

Taken together, these results show that the impact of JunB activity on the generation of AFs is MEK/ERK- and NF-κB-dependent, but Ras status-independent.

### VEGF and IGF1 are direct transcriptional targets of JunB in MM cells

JunB binding sites in DNA sequences within promoter and enhancer regions of VEGF, VEGFB, and IGF1 were identified using PROMO as well as the JASPAR database [32, 58, 59]. Our analysis proposes the presence of JunB binding sites in the promoter regions of VEGF (predicted binding site sequences: TTTGAATCATCA, CACTGACTAAC, GGGTGAGTGAG), VEGFB (predicted binding site sequences: GTGTGGTCAGC, TTGTGACCCATTG), and IGF1 (predicted binding site sequences: CATTACACAT, CTGTGAGTCAGTG, GGATTACTCAC) (Supplementary Table 2).

**Fig. 4:**
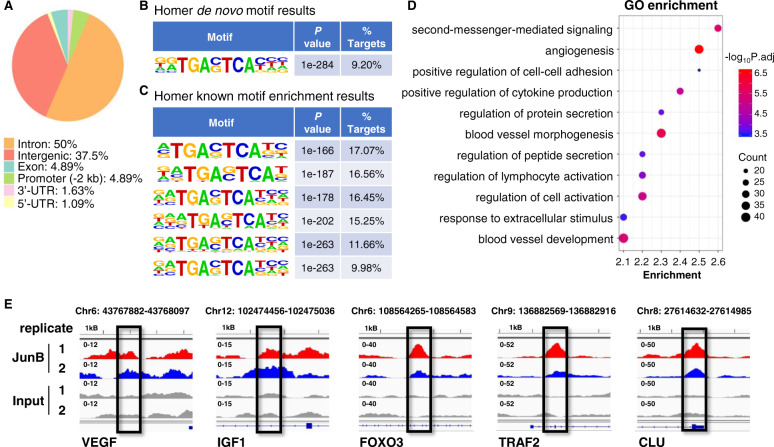
VEGF and IGF1 are direct transcriptional targets of JunB in MM cells. **A**–**C** Motif analysis of JunB-enriched sites in MM.1S cells treated with IL6 identified by chromatin immunoprecipitation sequencing (ChIP-seq). **A** Peak locations across the genome. The number indicates percent of ChIP-seq distribution. **B** Homer de novo motif results. **C** Homer known motif enrichment results. **D** Enriched Gene Ontology (GO) analysis of JunB target genes by Metascape. JunB direct target genes in MM.1S cells treated with IL6 were identified by ChIP-seq and subjected to GO enrichment analysis. **E** Representative ChIP-seq peaks located on JunB direct target genes VEGF, IGF1, FOXO3, TRAF2, and CLU, visualized by genome browser Integrative Genomics Viewer (IGV). Biological duplicates were performed for the ChIP-seq.

ChIP-seq analysis was performed next to identify direct JunB target genes in MM.1S cells stimulated with IL6. We identified significant enrichment of ChIP-seq peaks distributed across the genome, corresponding to 1046 unique genes common in both replicates (Fig. 4A and Supplementary Tables 4 and 5). Motif analysis with Homer was used to investigate genome-wide JunB-enriched sites. Both de novo and known enrichment results showed significant presence of JunB/AP-1 consensus motifs, e.g. Motif TGA(C/G)TCA, indicating that JunB ChIP-seq achieved a good enrichment of the putative JunB/AP-1 target genes (Fig. 4B, C). GO analysis of target genes showed an enrichment of GO terms associated with second messenger-mediated signaling, angiogenesis, blood vessel morphogenesis, regulation of cell activation, regulation of peptide/protein secretion, and positive regulation of cytokine production, as well as positive regulation of cell–cell adhesion (Fig. 4D and Supplementary Table 5). To further reveal the relationship between JunB target genes, we carried out protein–protein interaction enrichment analysis using STRING. The resultant network contains the subset of proteins that were connected directly (physically) and indirectly (functionally) with at least one other member (Supplementary Fig. 11A). The densely connected components in the network were then identified using Cytoscape and MCODE (Supplementary Fig. 11B–D). Since the highly interconnected regions, called MCODE clusters, are often protein complexes and parts of pathways, KEGG pathway and GO biological processes enrichment analysis was next applied to each cluster independently for functional description. Our results showed that genes in Cluster 1 were enriched in positive regulation of cytosolic calcium ion concentration, leukocyte chemotaxis, chemokine signaling pathway, and positive regulation of vasculature development. Genes in Cluster 2 were enriched in positive regulation of cell adhesion, and second messenger-mediated signaling; and genes in Cluster 3 were enriched in cytokine–cytokine receptor interaction, pathways in cancer, and JAK-STAT signaling pathway (Supplementary Table 6). Among direct JunB targets we identified AFs VEGF and IGF1. In addition, several genes that have been reported to be JunB/AP-1 targets, such as FOXO3, TRAF2, and CLU, were also identified (Fig. 4E and Supplementary Table 7). Thus, ChIP-seq data revealed mechanisms of target gene regulation by JunB in MM cells and further support a functional role of JunB in MM angiogenesis.

### JunB knockdown in MM cells results in inhibition of in vitro and in vivo angiogenesis

**Fig. 5:**
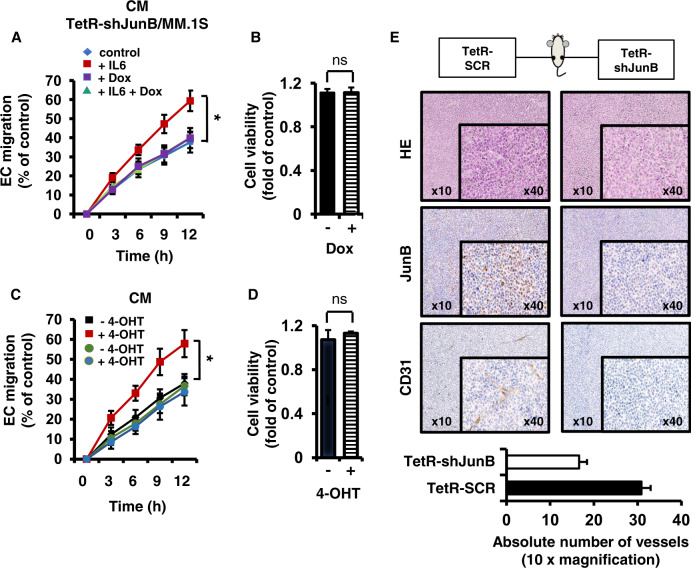
JunB knockdown in MM cells results in inhibition of in vitro and in vivo angiogenesis. **A** Supernatant derived from MM cells upon JunB knockdown inhibits endothelial cell (EC) migration. TetR-shJunB/MM cells were cultured in RPMI-1640 medium plus IL6 in the presence or absence of doxycycline (1 μg/ml). Conditioned media (CM) were collected after 24 h and wound healing was determined at indicated time points (closure of EC layer after mechanical disruption; mean width of initial cell gap = 200 μm). Data represent mean ± SD for triplicate samples. *p < 0.01. **B** Cell viability of MM cells treated as described in (**A**) after 24 h. Data represent mean ± SD for triplicate samples. ns non-significant. **C** Supernatant derived from MM cells after 4-OHT-induced JunB activation stimulates in vitro angiogenesis. JunB-ER/MM cells were cultured in RPMI-1640 medium and treated with 4-OHT (100 nM). CM were collected after 24 h and wound healing was determined as described in **A**. Black and red squares, JunB-ER/MM.1S; green and blue circles, IRES-GFP/MM.1S cells. *p < 0.01. **D** Cell viability of JunB-ER/MM cells after 4-OHT treatment for 24 h. ns non-significant. **E** Induced JunB silencing inhibits angiogenesis in a murine xenograft MM model. NOD/SCID mice were injected subcutaneously with TetR-SCR/MM.1S and TetR-shJunB/MM.1S together with human-derived BMSCs and matrigel into the left and right flank, respectively, and fed with doxycycline in their drinking water for 5 weeks. Representative microscopic images of tumor sections stained with antibodies against JunB and CD31 (10× and 40× magnification) (upper panel). In vivo angiogenesis was quantified by counting the number of vessels in 15 random view fields at 10× magnification. Values represent average ± SEM (95% CI of difference: 8.60–19.94) (lower panel).

Angiogenesis is a complex multistep process that requires a coordinated activity of endothelial cells (ECs). An indicator of the angiogenic effect of cytokines and growth factors is their ability to stimulate EC migration and to thereby enable tubuli formation. We next investigated whether JunB-regulated expression and secretion of indicated AFs has an impact on BM angiogenesis. As evidenced by wound-healing (Fig. 5A) and matrigel assays (Supplementary Fig. 12), decreased vascular EC migration and endothelial tubuli formation was observed in the presence of CM derived from doxycycline-treated versus untreated TetR-shJunB/MM cells following IL6 stimulation. Importantly, MM cell viability of doxycycline-treated and untreated cells was comparable at all time points investigated, thereby excluding an effect on angiogenesis due to cell death (Fig. 5B). Conversely, CM derived from 4-OHT-treated versus untreated JunB-ER/MM cells strongly induced EC migration (Fig. 5C). In contrast, CM derived from 4-OHT-treated control IRES-GFP/MM.1S cells induced similar EC migration as CM derived from untreated IRES-GFP/MM.1S cells. MM cell viability of 4-OHT-treated and untreated cells was comparable at all time points investigated (Fig. 5D).

In order to translate our in vitro data in vivo, NOD/SCID mice were injected subcutaneously with TetR-SCR/MM.1S and TetR-shJunB/MM.1S together with human-derived BMSCs and matrigel into the left and right flank, respectively, and fed with doxycycline for 5 weeks. Treatment with doxycycline significantly inhibited JunB and CD31 protein levels in tumors formed by TetR-shJunB/MM.1S but not in tumors formed by TetR-SCR/MM.1S (Fig. 5E). These data strongly support that JunB-mediated AF generation and secretion contributes to MM BM angiogenesis; and that inhibition of tumor growth, as evidenced by a significant decrease of Ki67 staining (Supplementary Fig. 13 [21]) by JunB knockdown, is mediated, at least in part, via antiangiogenesis.

**Fig. 6:**
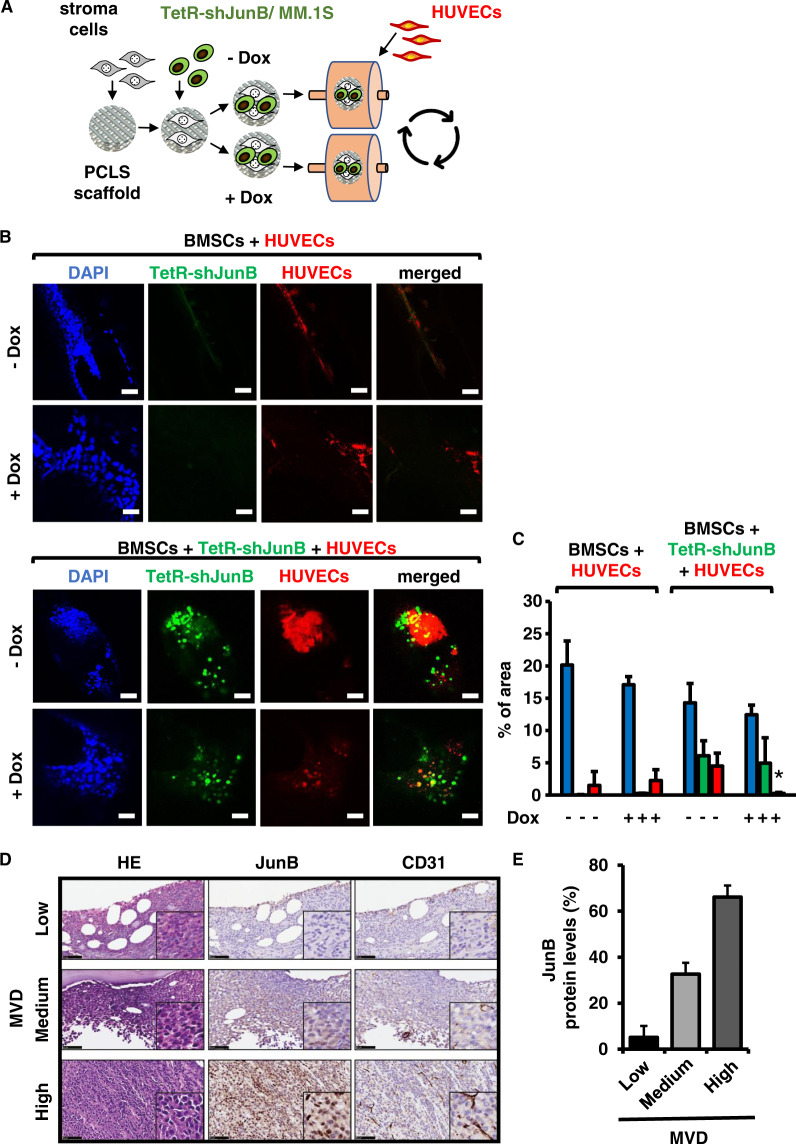
Positive expression of JunB in MM cells correlates with vessel density in an innovative 3D MM model and in patient-derived bone marrow biopsies. **A**–**C** Positive expression of JunB in MM cells correlates with bone marrow microvessel density in a 3D MM model. **A** Generation of a 3D MM microenvironment. KM-105 stroma cells were pre-seeded overnight onto poly-ε-caprolactone scaffolds (PCLS). TetR-shJunB/MM.1S cells were then added in the presence or absence of doxycycline, transferred into the bioreactor together with fluorescent-tagged-HUVE cells (CellTracker™ Deep Red dye) co-cultured for up to 72 h in RPMI-1640 media with 2% FBS. **B** Representative Z-stack confocal images of GFP+ TetR-shJunB/MM.1S cells (green) together with stroma cells, alone, or together with fluorescence-tagged HUVE cells (red). Scale bars = 100 μm. **C** Quantification of DAPI-positive, GFP+ TetR-shJunB/MM.1S and fluorescence-tagged HUVE cells in Z-stack confocal images of the 3D cultures. Image processing and analysis was performed with FiJi ImageJ. All cells (DAPI stain, blue), TetR-shJunB/MM.1S cells (green), HUVECs (red); *p < 0.005. **D**-**E **Positive expression of JunB in MM cells correlates with vessel density in patient-derived bone marrow biopsies. **D** Hematoxylin and eosin (HE) (left panels), JunB (middle vertical panels), and CD31 (right panels) immunohistochemical staining of BM sections from three representative MM patients with low (negative) JunB expression and MVD (upper panel), intermediate JunB expression and MVD (middle panels), and high JunB expression and MVD (lower panels), respectively (40× magnification). Scale bars = 50 μm. The quantification was performed as described in “Materials and Methods”; results for each BM sample are listed in Supplementary Table 3. **E** The percentage of JunB-positive cells is shown as mean ± SD and is associated with increased BM MVD. *p* = 0.0036 using one-way ANOVA analysis followed by Tukey’s multiple comparisons test.

Innovative 3D co-culture models replicate BM-specific structural features and closely recreate functional tumor-stroma cell and -extracellular matrix interactions [60, 61]. They therefore evolve as concise surrogates of the MM BM microenvironment. In order to further support our in vitro and in vivo data, we next utilized the biodegradable polymer PCL [62] as a bone-mimicking scaffold together with the stroma cell line KM-105, tumor cells, and ECs in the dynamic 3D-RCCS^TM^ bioreactor RCCS-1 (Synthecon Inc., Houston, USA). Of note, in contrast to passive encapsulation in 3D-hydrogel cell cultures, this model allows active environment-driven cell adherence to scaffolds. Results obtained with this innovative 3D co-culture model demonstrate that angiogenesis is inhibited by doxycycline-induced JunB knockdown thereby confirming our in vitro and in vivo data (Fig. 6A–C and Supplementary Fig. 14).

Finally, in agreement with these data, immunostaining for JunB and CD31 on BM samples derived from ten NDMM patients showed a significant correlation between JunB expression in BM plasma cells and MVD measurement (ANOVA *p* = 0.0036) (Fig. 6D, E and Supplementary Table 3). Of interest, patients with high versus medium and low JunB/MVD presented with adverse cytogenetics including 1q21 amplification (see also Fig. 1D), del17p and t(4;14) (Supplementary Table 3). In summary, these results further support a key role for JunB in the transcriptional regulation of AFs ultimately leading to MM BM angiogenesis.

## Discussion

BM angiogenesis represents a hallmark of MM pathogenesis. In continuation and extension to our previous study [21], the present report reveals for the first time that JunB is not only a mediator of MM cell survival, proliferation, and drug resistance, but also a promoter of AF transcription and consequently of MM BM angiogenesis. Specifically, our analyses determined a significant correlation between expression levels of JunB and AFs VEGF, VEGFB, and IGF1 in primary MM cells derived from NDMM but not from RRMM patients. These data are consistent with both, a decreasing dependency of MM cells on the BM microenvironment and decreasing BM MVD during disease progression. In contrast to other cancers such as renal cell carcinoma [19], we did not observe a correlation between JUNB, MMP2, and MMP9 expression, highlighting the dependency of JunB transcriptional programs on the tumor cell type [19, 63]. Different stimuli within the microenvironment as well as complex interactions between different dimerization partners and other TFs are presumably critical. In contrast to JunB, hypoxia and Hif-1α levels increase in the BM of MM patients during disease progression due to rapidly proliferating tumor cells and promote MM cell dissemination and bone destruction [45–50]. We therefore hypothesize that based on specific regulatory features within the BM microenvironment (hypoxia, the cellular compartment, and the humoral milieu in particular) JunB- and Hif-1α-transcriptional programs differentially regulate the expression of distinct target genes such as AFs. Our ongoing studies investigate the therapeutic value of combining AF-inhibitors with hypoxia-targeting agents in MM.

Moreover, our results suggest that JunB-induced generation of AFs is dependent on the molecular background of tumor cells. Ongoing studies seek to delineate molecular mechanisms by which JunB expression is correlated with VEGF, VEGFB, and IGF1 in the PR group, as well as with VEGF in patients with 3 or 4+ copies of 1q21. The functional relevance of JunB on AFs VEGF, VEGFB, and IGF1 production and secretion and resultant angiogenesis was subsequently demonstrated. Of interest, while JunB expression and function are MEK/MAPK- and NFκB-dependent, they are Ras-independent. In agreement with these data, recent findings showed that despite the high prevalence of Ras mutations in MM cells, only a small percentage of cases is associated with increased ERK phosphorylation [64].

Subsequent ChIP-seq analyses demonstrated that JunB directly regulates the expression of target genes associated with angiogenesis, cytokine and growth factor production and secretion, as well as cell–cell adhesion in MM cells. However, while PROMO and JASPAR database analyses predicted JunB binding sites in the promoter regions of VEGF, VEGFB, and IGF1, ChIP-seq identified direct binding of JunB only to the VEGF and IGF1, but not to the VEGFB locus. One explanation for this inconsistency is the high sensitivity but abysmal selectivity of these prediction analyses [58]. The antiangiogenic activity together with the inhibition of MM cell proliferation was subsequently also confirmed in an innovative dynamic 3D cell culture system and in an in vivo MM model.

Following thalidomide, the first clinically approved antiangiogenic agent, several other antiangiogenic inhibitors have been approved for cancer therapy during the last two decades. Most of these agents are directed against VEGF and VEGF receptor tyrosine kinases. Despite exciting preclinical in vitro and in vivo results, clinical approaches to specifically target the pre-eminent AF VEGF in MM have been disappointing. The abundance of AFs other than VEGF, their functional redundancy, and dynamic changes within the patient BM microenvironment throughout the disease are among the most likely reasons. We and others therefore hypothesized that the highest therapeutic impact of targeting VEGF and other AFs is achieved in early, predominantly VEGF-dependent stages of the disease, and with a cocktail of antiangiogenic inhibitors rather than with a single VEGF inhibitor [17]. As an exciting, alternative approach for the combined inhibition of co-regulated AFs and resultant MM BM angiogenesis, the present study strongly supports the high potential of therapeutically targeting JunB. Indeed, strategies to directly target TFs are heralded as the most direct and promising targets in cancer therapy with a potentially high therapeutic index. While nuclear hormone receptor transcription factors (NHR-TFs) for which derivatives of natural ligands (e.g., dexamethasone, prednisolone) are well-established MM therapeutics, non-NHR-TFs including AP-1 have been considered “un-druggable” until most recently. However, this paradigm does not hold true any longer. Advances of our knowledge on the complex composition of non-NHR-TFs and on their binding to specific DNA sequences have propelled the development of novel strategies to target TFs including inhibition of their expression; the induction of their degradation; the disruption of their interaction either with functionally critical protein binding partners or the DNA; and the regulation of their binding at the epigenetic level by modulation of the chromatin accessibility. Of note, T-5224, a non-peptic small molecule inhibitor (Toyama Chemical and Kitasato University), has been the first selective AP-1 inhibitor to enter a clinical phase II study. Several other compounds are under preclinical investigation and in the clinical pipeline [65–67].

**Fig. 7:**
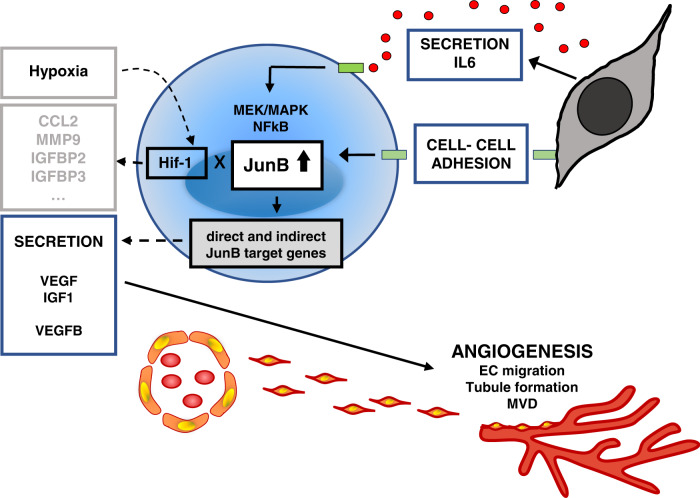
JunB regulates expression and secretion of angiogenic factors and thereby BM angiogenesis in MM. In contrast to Hif-1α which is induced by hypoxia in MM BM microenvironment, JunB upregulation in MM cell is triggered predominantly by IL-6 secreted from bone marrow stroma cells (BMSC) and also by direct MM: BMSC cell-cell contact. Importantly, induced upregulation of JunB is a critical, MEK/MAPK- and NFκB- dependent but Ras status- independent mediator for the transcription of key angiogenic factors VEGF, VEGFB and IGF1, and thereby MM BM angiogenesis, particularly in early MM.

In summary, our data demonstrate for the first time that stroma-, but not hypoxia-induced upregulation of JunB is a critical, MEK/MAPK- and NFκB-dependent but Ras-independent mediator for the transcription of key AFs VEGF, VEGFB and IGF1, and thereby MM BM angiogenesis, particularly in early MM (Fig. 7). Our results thereby add another facet of JunB to the pathophysiologic functions in MM and underscore worldwide efforts to directly target specific pathognomic AP-1 TFs such as JunB as one of the most promising future strategies in cancer and in MM therapy in particular.

## Supplementary information

Supplementary Figures 1–14 and Tables 1–3

Supplementary Table 4 (Unique JunB binding peaks identified by ChIP-seq.)

Supplementary Table 5 (Unique JunB binding targets identified by ChIP-seq.)

Supplementary Table 6 (KEGG and GO enrichment analysis of JunBand its target AFs, and of each MCODE cluster in Supplementary Fig. 11B-D.)

Supplementary Table 7 (JASPAR analysis of putative JunB binding sites in the peak sequences fromChIP-seq results related to Fig. 4E.)

## References

[1] Vacca A, Ribatti D (2006). Bone marrow angiogenesis in multiple myeloma. Leukemia.

[2] Vacca A, Ribatti D, Roncali L, Ranieri G, Serio G, Silvestris F (1994). Bone marrow angiogenesis and progression in multiple myeloma. Br J Haematol.

[3] Vacca A, Ria R, Semeraro F, Merchionne F, Coluccia M, Boccarelli A (2003). Endothelial cells in the bone marrow of patients with multiple myeloma. Blood.

[4] Nosàs-Garcia S, Moehler T, Wasser K, Kiessling F, Bartl R, Zuna I (2005). Dynamic contrast-enhanced MRI for assessing the disease activity of multiple myeloma: a comparative study with histology and clinical markers. J Magn Reson Imaging.

[5] Vacca A, Ribatti D, Presta M, Minischetti M, Iurlaro M, Ria R (1999). Bone marrow neovascularization, plasma cell angiogenic potential, and matrix metalloproteinase-2 secretion parallel progression of human multiple myeloma. Blood.

[6] Rajkumar SV, Leong T, Roche PC, Fonseca R, Dispenzieri A, Lacy MQ (2000). Prognostic value of bone marrow angiogenesis in multiple myeloma. Clin Cancer Res.

[7] Kumar S, Fonseca R, Dispenzieri A, Lacy MQ, Lust JA, Witzig TE (2002). Bone marrow angiogenesis in multiple myeloma: effect of therapy. Br J Haematol.

[8] Hillengass J, Wasser K, Delorme S, Kiessling F, Zechmann C, Benner A (2007). Lumbar bone marrow microcirculation measurements from dynamic contrast-enhanced magnetic resonance imaging is a predictor of event-free survival in progressive multiple myeloma. Clin Cancer Res.

[9] Kumar S, Witzig TE, Timm M, Haug J, Wellik L, Kimlinger TK (2004). Bone marrow angiogenic ability and expression of angiogenic cytokines in myeloma: evidence favoring loss of marrow angiogenesis inhibitory activity with disease progression. Blood.

[10] Hose D, Moreaux J, Meissner T, Seckinger A, Goldschmidt H, Benner A (2009). Induction of angiogenesis by normal and malignant plasma cells. Blood.

[11] Podar K, Anderson KC. Targeting multiple myeloma tumor angiogenesis: focus on VEGF. 2013. 10.1007/978-1-4614-4666-8_15.

[12] Kumar S, Gertz MA, Dispenzieri A, Lacy MQ, Wellik LA, Fonseca R (2004). Prognostic value of bone marrow angiogenesis in patients with multiple myeloma undergoing high-dose therapy. Bone Marrow Transpl.

[13] Neben K, Moehler T, Egerer G, Kraemer A, Hillengass J, Benner A (2001). High plasma basic fibroblast growth factor concentration is associated with response to thalidomide in progressive multiple myeloma. Clin Cancer Res.

[14] Kumar S, Witzig TE, Dispenzieri A, Lacy MQ, Wellik LE, Fonseca R (2004). Effect of thalidomide therapy on bone marrow angiogenesis in multiple myeloma. Leukemia.

[15] D’Amato RJ, Loughnan MS, Flynn E, Folkman J (1994). Thalidomide is an inhibitor of angiogenesis. Proc Natl Acad Sci USA.

[16] Singhal S, Mehta J, Desikan R, Ayers D, Roberson P, Eddlemon P (1999). Antitumor activity of thalidomide in refractory multiple myeloma. N Engl J Med.

[17] Podar K, Anderson KC (2011). Emerging therapies targeting tumor vasculature in multiple myeloma and other hematologic and solid malignancies. Curr Cancer Drug Targets.

[18] Schorpp-Kistner M, Wang ZQ, Angel P, Wagner EF (1999). JunB is essential for mammalian placentation. EMBO J.

[19] Kanno T, Kamba T, Yamasaki T, Shibasaki N, Saito R, Terada N (2012). JunB promotes cell invasion and angiogenesis in VHL-defective renal cell carcinoma. Oncogene.

[20] Reiss Y, Knedla A, Tal AO, Schmidt MHH, Jugold M, Kiessling F (2009). Switching of vascular phenotypes within a murine breast cancer model induced by angiopoietin-2. J Pathol.

[21] Fan F, Bashari MH, Morelli E, Tonon G, Malvestiti S, Vallet S (2017). The AP-1 transcription factor JunB is essential for multiple myeloma cell proliferation and drug resistance in the bone marrow microenvironment. Leukemia.

[22] Bakiri L, Lallemand D, Bossy-Wetzel E, Yaniv M (2000). Cell cycle-dependent variations in c-Jun and JunB phosphorylation: a role in the control of cyclin D1 expression. EMBO J.

[23] Zhang J, Sattler M, Tonon G, Grabher C, Lababidi S, Zimmerhackl A (2009). Targeting angiogenesis via a c-Myc/hypoxia-inducible factor-1alpha-dependent pathway in multiple myeloma. Cancer Res.

[24] Zhan F, Barlogie B, Arzoumanian V, Huang Y, Williams DR, Hollmig K (2007). Gene-expression signature of benign monoclonal gammopathy evident in multiple myeloma is linked to good prognosis. Blood.

[25] Zhan F, Huang Y, Colla S, Stewart JP, Hanamura I, Gupta S (2006). The molecular classification of multiple myeloma. Blood.

[26] Chng WJ, Kumar S, Vanwier S, Ahmann G, Price-Troska T, Henderson K (2007). Molecular dissection of hyperdiploid multiple myeloma by gene expression profiling. Cancer Res.

[27] Subramanian A, Tamayo P, Mootha VK, Mukherjee S, Ebert BL, Gillette MA (2005). Gene set enrichment analysis: a knowledge-based approach for interpreting genome-wide expression profiles. Pro Natl Acad Sci USA.

[28] Barretina J, Caponigro G, Stransky N, Venkatesan K, Margolin AA, Kim S (2012). The Cancer Cell Line Encyclopedia enables predictive modelling of anticancer drug sensitivity. Nature.

[29] Ghandi M, Huang FW, Jané-Valbuena J, Kryukov GV, Lo CC, McDonald ER (2019). Next-generation characterization of the Cancer Cell Line Encyclopedia. Nature.

[30] Carr TM, Wheaton JD, Houtz GM, Ciofani M (2017). JunB promotes Th17 cell identity and restrains alternative CD4+ T-cell programs during inflammation. Nat Commun.

[31] Zhou Y, Zhou B, Pache L, Chang M, Khodabakhshi AH, Tanaseichuk O (2019). Metascape provides a biologist-oriented resource for the analysis of systems-level datasets. Nat Commun.

[32] Fornes O, Castro-Mondragon JA, Khan A, van der Lee R, Zhang X, Richmond PA (2020). JASPAR 2020: update of the open-access database of transcription factor binding profiles. Nucleic Acids Res.

[33] Messeguer X, Escudero R, Farré D, Núñez O, Martínez J, Albà MM (2002). PROMO: detection of known transcription regulatory elements using species-tailored searches. Bioinformatics.

[34] Farré D, Roset R, Huerta M, Adsuara JE, Roselló L, Albà MM (2003). Identification of patterns in biological sequences at the ALGGEN server: PROMO and MALGEN. Nucleic Acids Res.

[35] Szklarczyk D, Gable AL, Lyon D, Junge A, Wyder S, Huerta-Cepas J (2019). STRING v11: protein-protein association networks with increased coverage, supporting functional discovery in genome-wide experimental datasets. Nucleic Acids Res.

[36] Shannon P, Markiel A, Ozier O, Baliga NS, Wang JT, Ramage D (2003). Cytoscape: a software environment for integrated models of biomolecular interaction networks. Genome Res.

[37] Bader GD, Hogue CWV (2003). An automated method for finding molecular complexes in large protein interaction networks. BMC Bioinforma.

[38] Persson M, Lehenkari PP, Berglin L, Turunen S, Finnilä MAJ, Risteli J (2018). Osteogenic differentiation of human mesenchymal stem cells in a 3D woven scaffold. Sci Rep.

[39] Schindelin J, Arganda-Carreras I, Frise E, Kaynig V, Longair M, Pietzsch T (2012). Fiji: an open-source platform for biological-image analysis. Nat Methods.

[40] Roussou M, Tasidou A, Dimopoulos MA, Kastritis E, Migkou M, Christoulas D (2009). Increased expression of macrophage inflammatory protein-1alpha on trephine biopsies correlates with extensive bone disease, increased angiogenesis and advanced stage in newly diagnosed patients with multiple myeloma. Leukemia.

[41] Rana C, Sharma S, Agrawal V, Singh U (2010). Bone marrow angiogenesis in multiple myeloma and its correlation with clinicopathological factors. Ann Hematol.

[42] Schneider CA, Rasband WS, Eliceiri KW (2012). NIH Image to ImageJ: 25 years of image analysis. Nat Methods.

[43] Sipkins DA, Wei X, Wu JW, Runnels JM, Côté D, Means TK (2005). In vivo imaging of specialized bone marrow endothelial microdomains for tumour engraftment. Nature.

[44] Schito L, Semenza GL (2016). Hypoxia-inducible factors: master regulators of cancer progression. Trends Cancer.

[45] Azab AK, Hu J, Quang P, Azab F, Pitsillides C, Awwad R (2012). Hypoxia promotes dissemination of multiple myeloma through acquisition of epithelial to mesenchymal transition-like features. Blood.

[46] Storti P, Bolzoni M, Donofrio G, Airoldi I, Guasco D, Toscani D (2013). Hypoxia-inducible factor (HIF)-1α suppression in myeloma cells blocks tumoral growth in vivo inhibiting angiogenesis and bone destruction. Leukemia.

[47] Colla S, Storti P, Donofrio G, Todoerti K, Bolzoni M, Lazzaretti M (2010). Low bone marrow oxygen tension and hypoxia-inducible factor-1α overexpression characterize patients with multiple myeloma: role on the transcriptional and proangiogenic profiles of CD138(+) cells. Leukemia.

[48] Zhang J, Sattler M, Tonon G, Grabher C, Lababidi S, Zimmerhackl A, et al. Targeting angiogenesis via a c-Myc/hypoxia-inducible factor-1α-dependent pathway in multiple myeloma. Cancer Res. 2009;69. 10.1158/0008-5472.CAN-08-4603. 10.1158/0008-5472.CAN-08-4603 19509231

[49] Hu J, Handisides DR, Van Valckenborgh E, De Raeve H, Menu E, Vande Broek I (2010). Targeting the multiple myeloma hypoxic niche with TH-302, a hypoxia-activated prodrug. Blood.

[50] Storti P, Donofrio G, Colla S, Airoldi I, Bolzoni M, Agnelli L (2011). HOXB7 expression by myeloma cells regulates their pro-angiogenic properties in multiple myeloma patients. Leukemia.

[51] Walker BA, Boyle EM, Wardell CP, Murison A, Begum DB, Dahir NM (2015). Mutational spectrum, copy number changes, and outcome: results of a sequencing study of patients with newly diagnosed myeloma. J Clin Oncol.

[52] Carrasco DR, Tonon G, Huang Y, Zhang Y, Sinha R, Feng B (2006). High-resolution genomic profiles define distinct clinico-pathogenetic subgroups of multiple myeloma patients. Cancer Cell.

[53] Hanamura I, Stewart JP, Huang Y, Zhan F, Santra M, Sawyer JR (2006). Frequent gain of chromosome band 1q21 in plasma-cell dyscrasias detected by fluorescence in situ hybridization: incidence increases from MGUS to relapsed myeloma and is related to prognosis and disease progression following tandem stem-cell transplantation. Blood.

[54] Liu P, Leong T, Quam L, Billaud J-N, Kay NE, Greipp P (1996). Activating mutations of N- and K-ras in multiple myeloma show different clinical associations: analysis of the Eastern Cooperative Oncology Group phase III trial. Blood.

[55] Neri A, Murphy JP, Cro L, Ferrero D, Tarella C, Baldini L (1989). Ras oncogene mutation in multiple myeloma. J Exp Med.

[56] Corradini P, Ladetto M, Voena C, Palumbo A, Inghirami G, Knowles DM (1993). Mutational activation of N- and K-ras oncogenes in plasma cell dyscrasias. Blood.

[57] Bezieau S, Devilder MC, Avet-Loiseau H, Mellerin MP, Puthier D, Pennarun E (2001). High incidence of N and K-Ras activating mutations in multiple myeloma and primary plasma cell leukemia at diagnosis. Hum Mutat.

[58] Wasserman WW, Sandelin A (2004). Applied bioinformatics for the identification of regulatory elements. Nat Rev Genet.

[59] Stormo GD (2013). Modeling the specificity of protein-DNA interactions. Quant Biol.

[60] Ferrarini M, Steimberg N, Boniotti J, Berenzi A, Belloni D, Mazzoleni G (2017). 3D-dynamic culture models of multiple myeloma. Methods Mol Biol.

[61] Papadimitriou K, Kostopoulos IV, Tsopanidou A, Orologas-Stavrou N, Kastritis E, Tsitsilonis O, et al. Ex vivo models simulating the bone marrow environment and predicting response to therapy in multiple myeloma. Cancers (Basel). 2020;12. 10.3390/cancers12082006. 10.3390/cancers12082006 PMC7463609 32707884

[62] Calimeri T, Battista E, Conforti F, Neri P, Di Martino MT, Rossi M (2011). A unique three-dimensional SCID-polymeric scaffold (SCID-synth-hu) model for in vivo expansion of human primary multiple myeloma cells. Leukemia.

[63] Schmidt D, Textor B, Pein OT, Licht AH, Andrecht S, Sator-Schmitt M (2007). Critical role for NF-kappaB-induced JunB in VEGF regulation and tumor angiogenesis. EMBO J.

[64] Xu J, Pfarr N, Endris V, Mai EK, Md Hanafiah NH, Lehners N (2017). Molecular signaling in multiple myeloma: association of RAS/RAF mutations and MEK/ERK pathway activation. Oncogenesis.

[65] Ye N, Ding Y, Wild C, Shen Q, Zhou J (2014). Small molecule inhibitors targeting activator protein 1 (AP-1). J Med Chem.

[66] Sun X, Gao H, Yang Y, He M, Wu Y, Song Y (2019). PROTACs: great opportunities for academia and industry. Signal Transduct Target Ther.

[67] Li S, Vallet S, Sacco A, Roccaro A, Lentzsch S, Podar K (2019). Targeting transcription factors in multiple myeloma: evolving therapeutic strategies. Expert Opin Investig Drugs.

