# Publisher Correction: JunB is a key regulator of multiple myeloma bone marrow angiogenesis

**DOI:** 10.1038/s41375-021-01367-2

**Published:** 2021-09-06

**Authors:** Fengjuan Fan, Stefano Malvestiti, Sonia Vallet, Judith Lind, Jose Manuel Garcia-Manteiga, Eugenio Morelli, Qinyue Jiang, Anja Seckinger, Dirk Hose, Hartmut Goldschmidt, Andreas Stadlbauer, Chunyan Sun, Heng Mei, Martin Pecherstorfer, Latifa Bakiri, Erwin F. Wagner, Giovanni Tonon, Martin Sattler, Yu Hu, Pierfrancesco Tassone, Dirk Jaeger, Klaus Podar

**Affiliations:** 1 Institute of Hematology, Union Hospital, Tongji Medical College, Huazhong University of Science and Technology, Wuhan, China; 2 Department of Medical Oncology, National Center for Tumor Diseases (NCT), University of Heidelberg, Heidelberg, Germany; 3 Department of Internal Medicine II, University Hospital Krems, Krems an der Donau, Austria; 4 Molecular Oncology and Hematology Unit, Karl Landsteiner University of Health Sciences, Krems an der Donau, Austria; 5 Center for Translational Genomics and Bioinformatics, IRCCS San Raffaele Scientific Institute, Milan, Italy; 6 Department of Experimental and Clinical Medicine, University “Magna Græcia” of Catanzaro, Catanzaro, Italy; 7 Department of Medicine, Harvard Medical School, Boston, MA USA; 8 University Hospital Heidelberg, Heidelberg, Germany; 9 Laboratory of Hematology and Immunology & Laboratory for Myeloma Research, Vrije Universiteit Brussel (VUB) Belgium, Brussels, Belgium; 10 Department of Neurosurgery, Friedrich-Alexander University (FAU) Erlangen-Nürnberg, Erlangen, Germany; 11 Institute of Medical Radiology, University Hospital St. Pölten, Karl Landsteiner University of Health Sciences, Krems an der Donau, Austria; 12 Genes & Disease Group, Department of Dermatology, Medical University of Vienna (MUW), Vienna, Austria; 13 Genes & Disease Group, Department of Laboratory Medicine, Medical University of Vienna (MUW), Vienna, Austria; 14 Functional Genomics of Cancer Unit, Experimental Oncology Division, IRCCS San Raffaele Scientific Institute, Milan, Italy; 15 Department of Surgery, Brigham and Women’s Hospital, Boston, MA USA

**Keywords:** Cell signalling, Myeloma

Correction to: *Leukemia* 10.1038/s41375-021-01271-9, published online 18 May 2021

The article JunB is a key regulator of multiple myeloma bone marrow angiogenesis, written by Fengjuan Fan, Stefano Malvestiti, Sonia Vallet, Judith Lind, Jose Manuel Garcia-Manteiga, Eugenio Morelli, Qinyue Jiang, Anja Seckinger, Dirk Hose, Hartmut Goldschmidt, Andreas Stadlbauer, Chunyan Sun, Heng Mei, Martin Pecherstorfer, Latifa Bakiri, Erwin F. Wagner, Giovanni Tonon, Martin Sattler, Yu Hu, Pierfrancesco Tassone, Dirk Jaeger & Klaus Podar, was originally published electronically on the publisher’s internet portal on 18 May 2021 without open access. With the author(s)’ decision to opt for Open Choice the copyright of the article changed on 29.06.2021 to © The Author(s) 2021 and the article is forthwith distributed under a Creative Commons Attribution.

**Open Access** This article is licensed under a Creative Commons Attribution 4.0 International License, which permits use, sharing, adaptation, distribution and reproduction in any medium or format, as long as you give appropriate credit to the original author(s) and the source, provide a link to the Creative Commons licence, and indicate if changes were made.

The images or other third party material in this article are included in the article’s Creative Commons licence, unless indicated otherwise in a credit line to the material. If material is not included in the article’s Creative Commons licence and your intended use is not permitted by statutory regulation or exceeds the permitted use, you will need to obtain permission directly from the copyright holder.

To view a copy of this licence, visit http://creativecommons.org/licenses/by/4.0/

