# Supplementary material for: JunB is a key regulator of multiple myeloma bone marrow angiogenesis
Source: Leukemia. 2021 May 18;35(12):3509–25. doi: 10.1038/s41375-021-01271-9 (PMC8632680; doi:10.1038/s41375-021-01271-9)
Supplement: Supplementary file 1 — Supplementary Figures 1–14 and Tables 1–3 [file 41375_2021_1271_MOESM1_ESM.pdf]

SUPPLEMENTARY DATA

Supplementary Figures 1-14;

Supplementary Tables 1-3.

Supplementary Figures

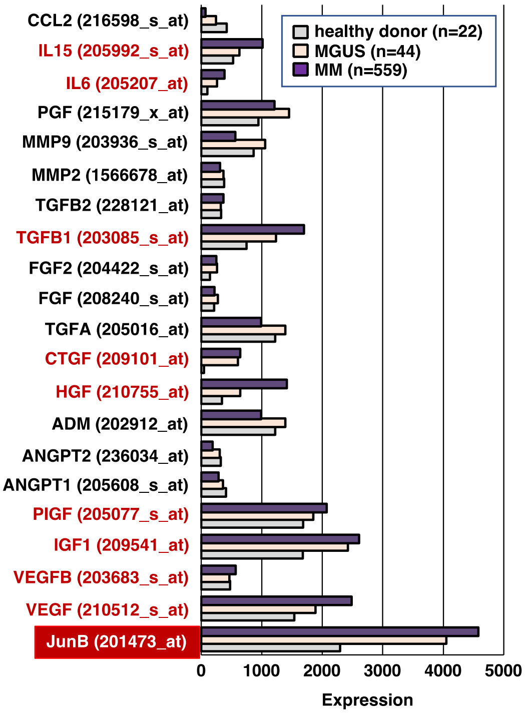

Supplementary Figure 1: Comparative supervised analysis of JunB and angiogenic factors (AFs). Raw expression values of JunB and AFs in healthy donors (n = 22) *versus* MGUS (n = 44) and MM (n = 559) samples utilizing publicly available datasets GSE 2658 and GSE5900. In red, JunB and AFs, for which the Pearson correlation score was subsequently calculated.

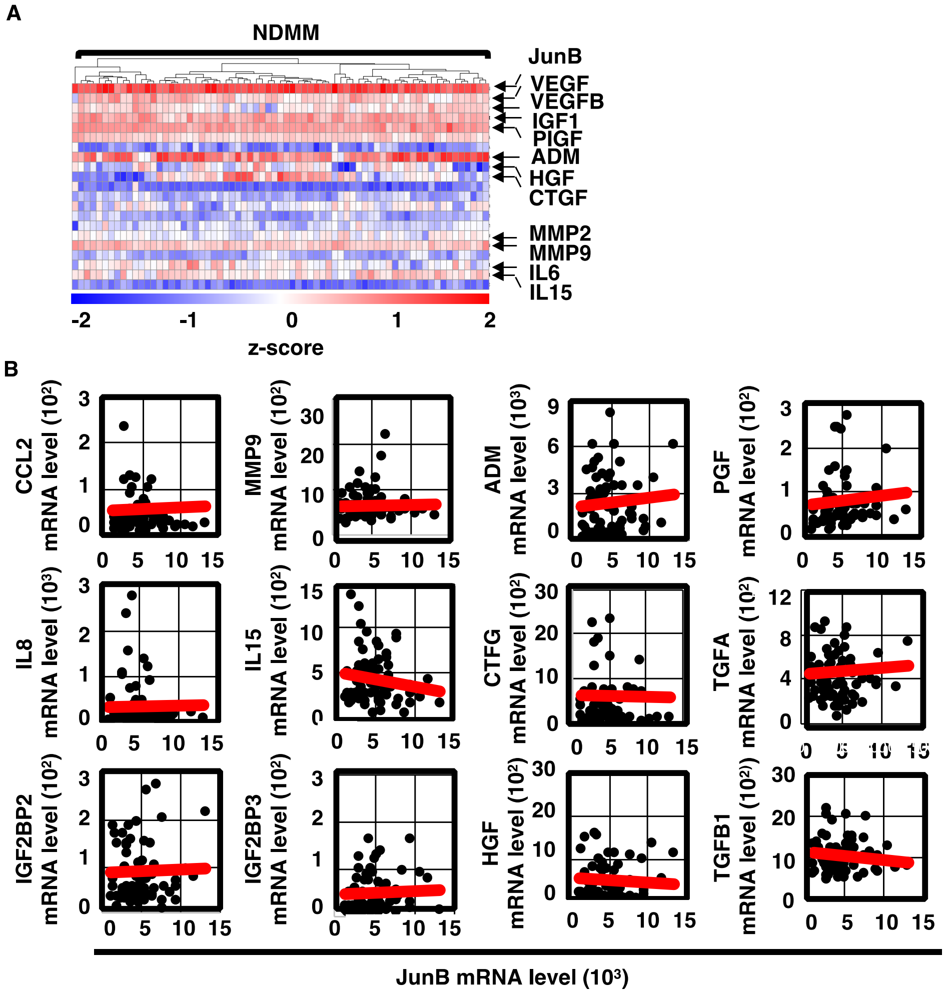

	r	p
CCL2	0.01	0.89
MMP9	0.03	0.79
IL8	0.01	0.92
IL15	-0.14	0.23
IGF2BP2	0.03	0.80
IGF2BP3	0.02	0.80
ADM	0.06	0.57
PGF	0.07	0.56
CTFG	0.00	0.99
TGFA	0.04	0.72
HGF	-0.20	0.07
TGFB1	-0.10	0.39

Supplementary Figure 2: In contrast to VEGF, VEGFB, and IGF1, JunB expression levels do not correlate with other AFs in primary MM cells obtained from newly diagnosed patients. (A) Heatmap of expression values (log2 and Z-score scaled across genes) of samples obtained from newly diagnosed MM patients (NDMM) for indicated genes within the GSE 6477 dataset. **(B)** The Pearson score was calculated to evaluate the correlation between JunB and AFs in samples of the NDMM subgroup within the GSE 6477 dataset. The minimal level of significance was $p < 0.05$. Table represents Pearson correlation scores and *p*- values.

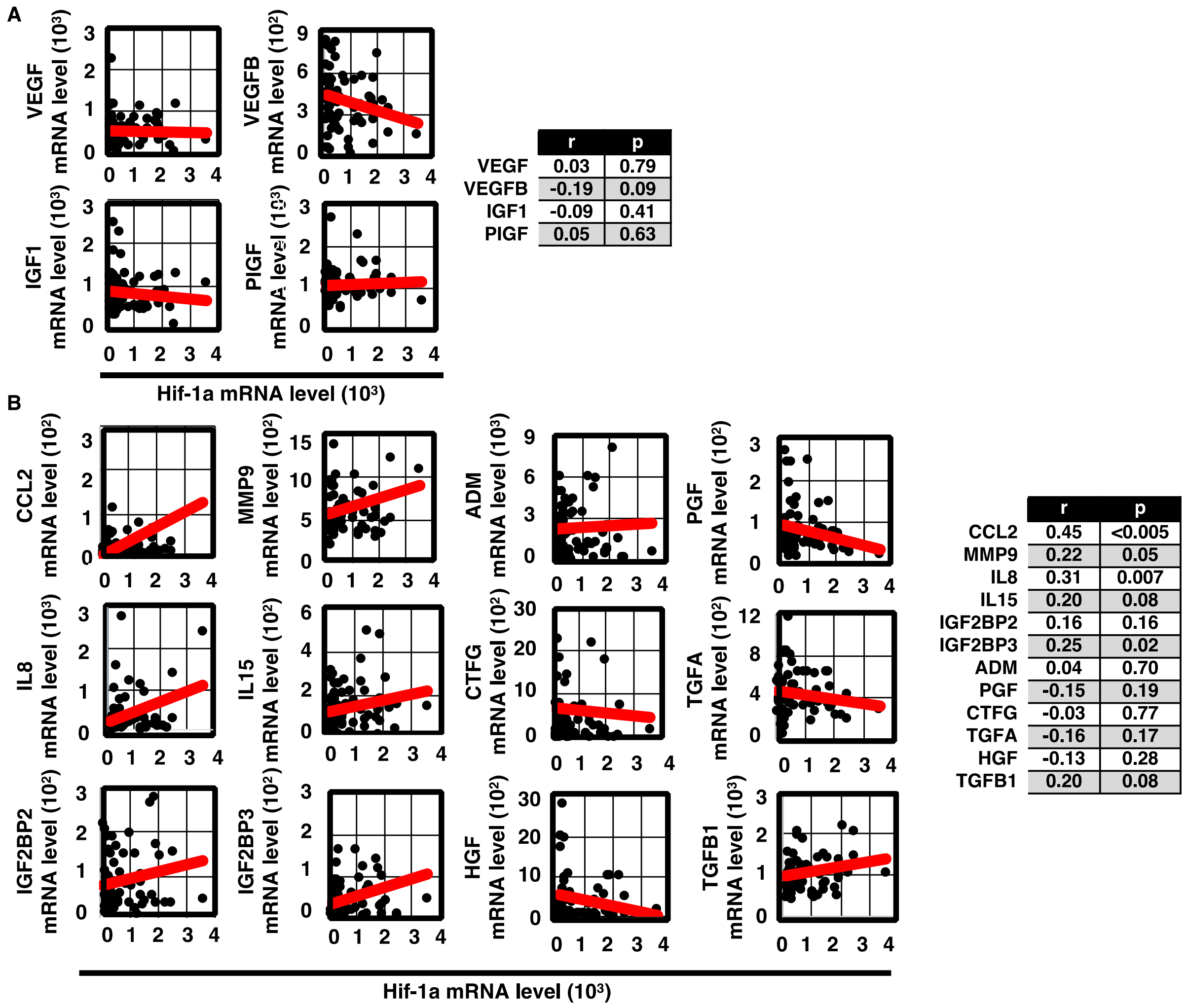

	r	p
VEGF	0.03	0.79
VEGFB	-0.19	0.09
IGF1	-0.09	0.41
PIGF	0.05	0.63

	r	p
CCL2	0.45	<0.005
MMP9	0.22	0.05
IL8	0.31	0.007
IL15	0.20	0.08
IGF2BP2	0.16	0.16
IGF2BP3	0.25	0.02
ADM	0.04	0.70
PGF	-0.15	0.19
CTFG	-0.03	0.77
TGFA	-0.16	0.17
HGF	-0.13	0.28
TGFB1	0.20	0.08

Supplementary Figure 3: In contrast to JunB, Hif-1α expression levels do not correlate with VEGF, VEGFB, IGF1 (A) but with CCL2, IL8, and IGF2BP3 (B) in newly diagnosed MM (NDMM) patients. The Pearson score was calculated to evaluate the correlation between Hif-1α and AFs in samples of the NDMM subgroup within the GSE 6477 dataset. The minimal level of significance was $p < 0.05$. Table represents Pearson correlation scores and *p*- values.

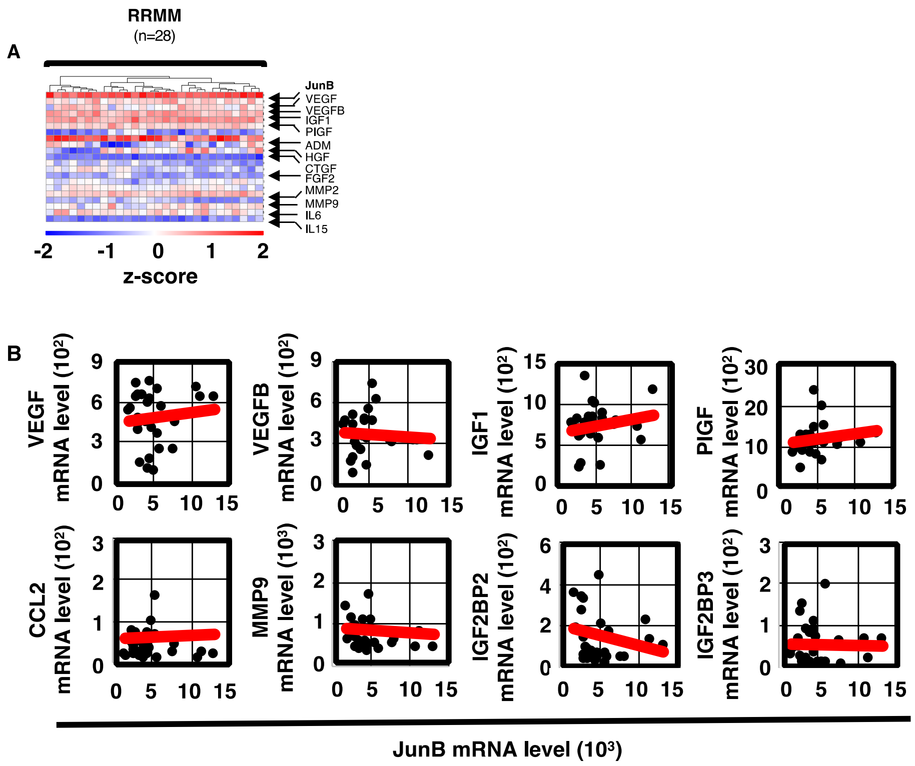

	r	p
VEGF	0.11	0.56
VEGFB	-0.07	0.72
IGF1	0.18	0.33
PIGF	0.17	0.36
CCL2	0.03	0.87
MMP9	-0.04	0.82
IGF2BP2	-0.18	0.35
IGF2BP3	0.02	0.91

Supplementary Figure 4: In contrast to NDMM, expression profiles of JunB and AFs VEGF, VEGFB, and IGF1 do not correlate in primary MM cells obtained from patients with relapsed/ refractory MM (RRMM). (A) Heatmap of expression values (log2 and Z-score scaled across genes) of samples obtained from RRMM patients for indicated genes within the GSE 6477 dataset. **(B)** The Pearson score was calculated to evaluate the correlation between JunB and AFs in samples of primary MM cells obtained from patients with RRMM within the GSE 6477 dataset. The minimal level of significance was $p < 0.05$.

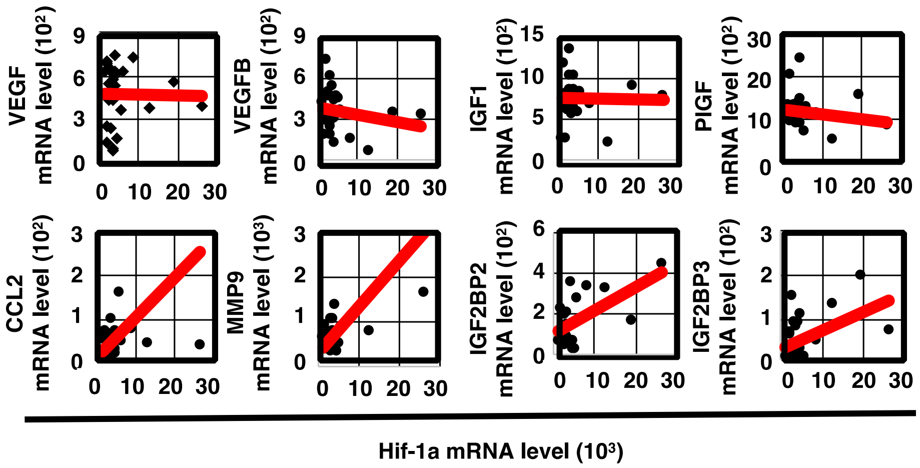

	r	p
VEGF	0.01	0.96
VEGFB	-0.19	0.31
IGF1	0.01	0.92
PIGF	-0.17	0.38
CCL2	0.56	0.007
MMP9	0.66	<0.001
IGF2BP2	0.42	0.02
IGF2BP3	0.47	0.01

Supplementary Figure 5: Hif-1α expression levels do not correlate with VEGF, VEGFB, IGF1 but with CCL2, MMP9, IGF2BP2 and IGF2BP3 in relapsed/ refractory MM (RRMM) patients. The Pearson score was calculated to evaluate the correlation between Hif-1α and AFs in samples of the RRMM subgroup within the GSE 6477 dataset. The minimal level of significance was $p < 0.05$. Table represents Pearson correlation scores and *p*- values.

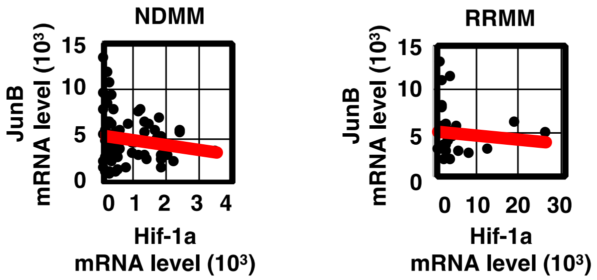

NDMM	r	p
Hif1 : JunB	-0.14	0.22

RRMM	r	p
Hif1 : JunB	-0.10	0.59

Supplementary Figure 6: JunB and Hif-1α expression levels neither correlate in samples obtained from newly diagnosed (NDMM) nor in samples obtained from relapsed/ refractory MM (RRMM) patients. The Pearson score was calculated to evaluate the correlation between JunB and Hif-1α in samples of primary MM cells obtained from ND and RR MM patients within the GSE 6477 dataset. The minimal level of significance was $p < 0.05$. Tables represent Pearson correlation scores and *p*- values.

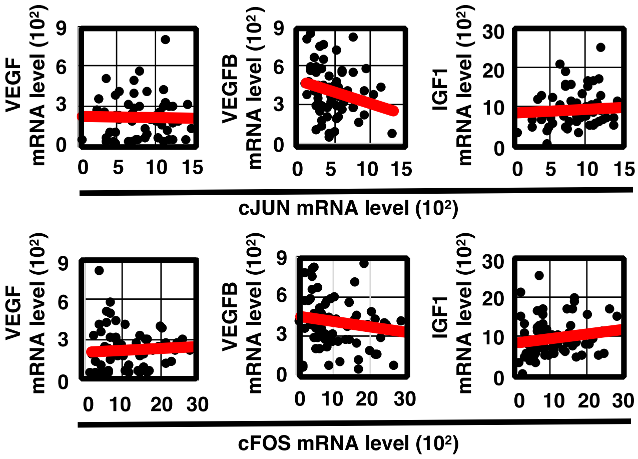

	r	p
VEGF	0.04	0.75
VEGFB	-0.21	0.08
IGF1	0.12	0.31

	r	p
VEGF	0.06	0.61
VEGFB	-0.17	0.14
IGF1	0.19	0.10

Supplementary Figure 7: Correlation of AP-1 TFs other than JunB with angiogenic factors (AFs). No statistically significant correlation score was obtained for AP-1 TFs JUN and FOS. The Pearson correlation coefficient was used to evaluate the correlation between AP-1 TFs JUN and FOS and AFs VEGF, VEGFB, and IGF1 within the GSE 6477 dataset. The minimal level of significance was $p < 0.05$.

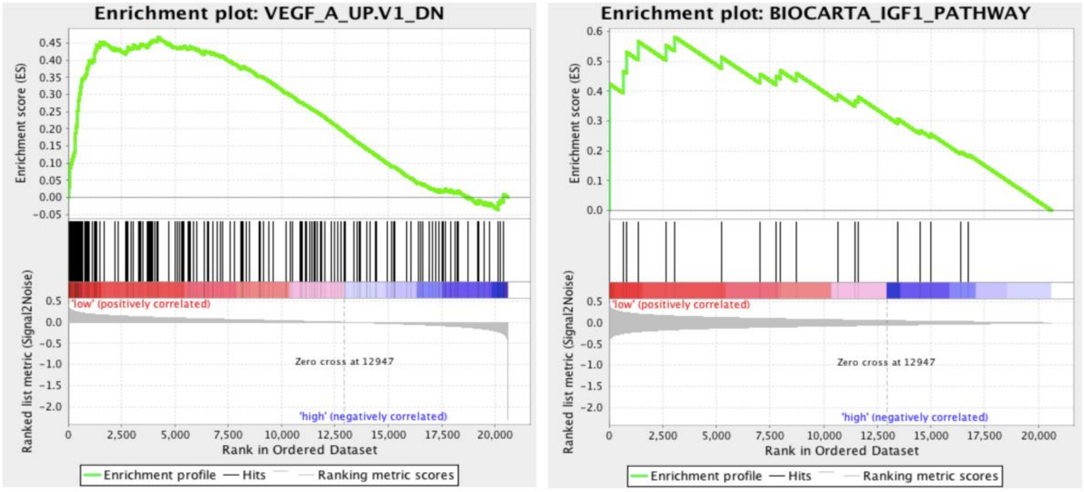

Supplementary Figure 8: GSEA of VEGF- and IGF1- signaling pathways. Gene Set Enrichment Analysis (GSEA) of JunB low *versus* JunB high expressing NDMM patients within GSE2658 dataset demonstrates statistically significant, concordant differences between JunB and a priori defined set of genes within the VEGF- and IGF1- signaling pathways.

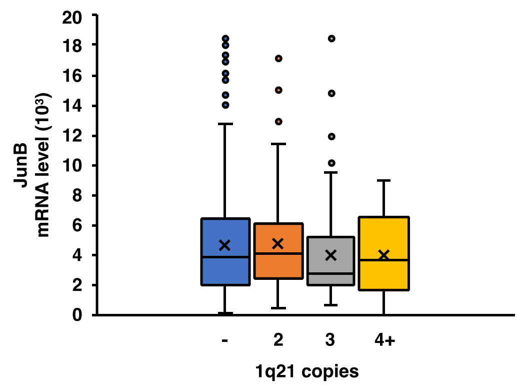

Supplementary Figure 9: JunB expression levels are not dependent on 1q21 copy numbers. JunB expression was investigated in patients with 1q21 amplification utilizing the GSE2658 dataset.

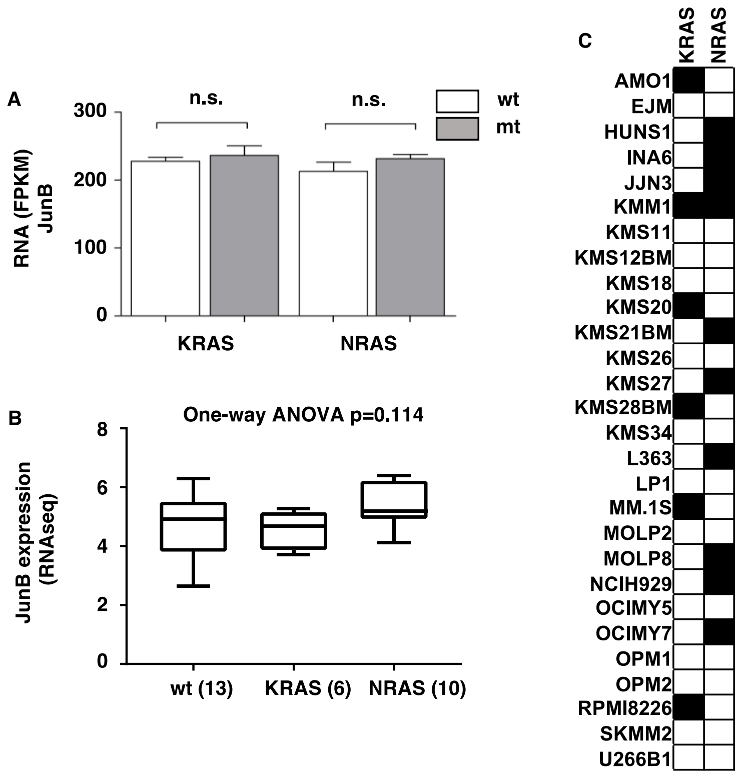

Supplementary Figure 10: JunB expression levels are independent of mutant Ras. (A) JunB expression levels do not differ in MM patients harboring Ras wildtype (wt) *versus* Ras mutations (mt). JunB expression levels in patients with wildtype NRAS *versus* mutant NRAS (p = 0.2387) or wildtype KRAS *versus* mutant KRAS (p = 0.6233) were analyzed in the CoMMpass IA12 dataset (n-908)**.** n.s. non- significant. **(B, C)** JunB expression levels do not differ in the CCLE dataset of MM cell lines. JunB levels were compared in MM cell lines harboring wildtype (wt) KRAS and NRAS *versus* cell lines harboring mutated KRAS or mutated NRAS with one-way ANOVA analysis (p = 0.114). **(C)**

MM cell lines harboring KRas mutations: AMO-1: A146T-het; KMM1: G13D-homo; KMS20: G12S-homo; KMS28BM: G125-homo; MM.1S: G12A-het; RPMI-8226: G12A-het; MM cell lines harbouring NRas mutations: HUNS1: D108N-het; INA-6: G12D-het; JJN3: Q61K-het; KMM1: G13D-homo; KMS21BM: Q61L-het; KMS27: Q61R-het; L363: Q61H-het; MOLP8: Q61L-het; NCIH929: G13D-het; OCIMY7: Q61K-het.

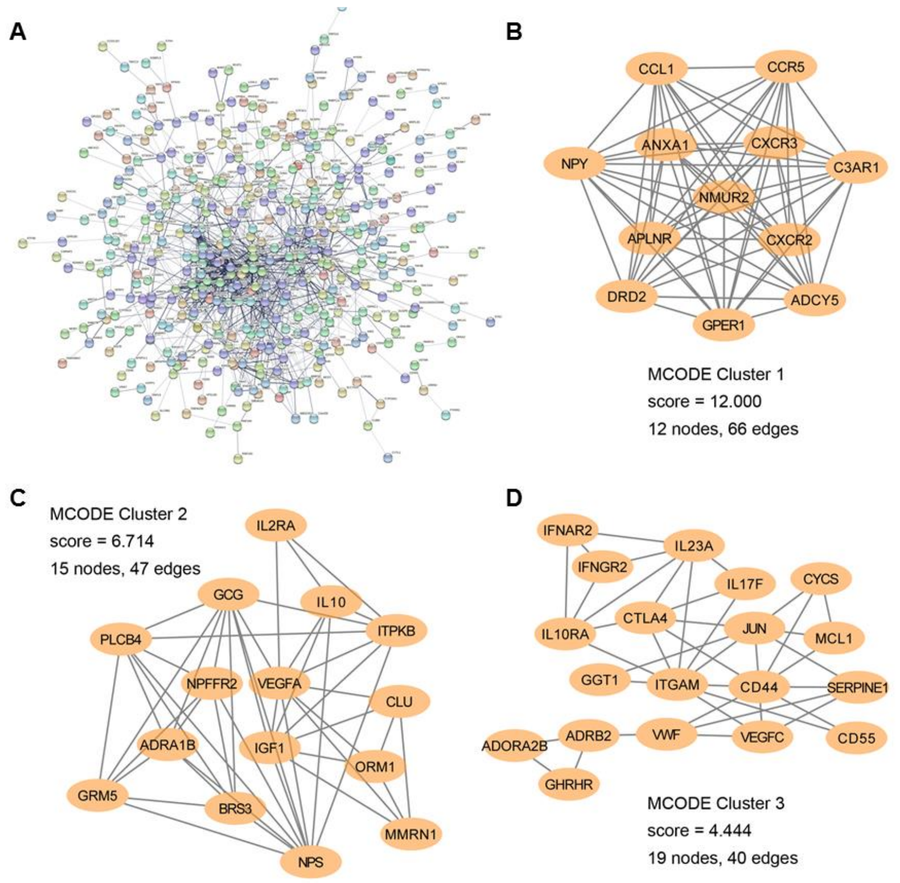

Supplementary Figure 11: Protein-protein interaction network and MCODE components identified in JunB target genes. (A) Protein-protein interaction network analyzed by STRING with minimum required interaction score of 0.400 (medium confidence). Network nodes represent all the proteins produced by a single, protein-coding gene locus. Edges represent protein-protein associations including text-mining, experiments, databases, co-expression, neighborhood, gene fusion, and co-occurrence. Line thickness indicates the strength of data support. Disconnected nodes in the network were hidden. **(B-D)** MCODE clusters analyzed by Cytoscape. Three top scored clusters in the network from (A) are shown. All analyses were carried out with default settings.

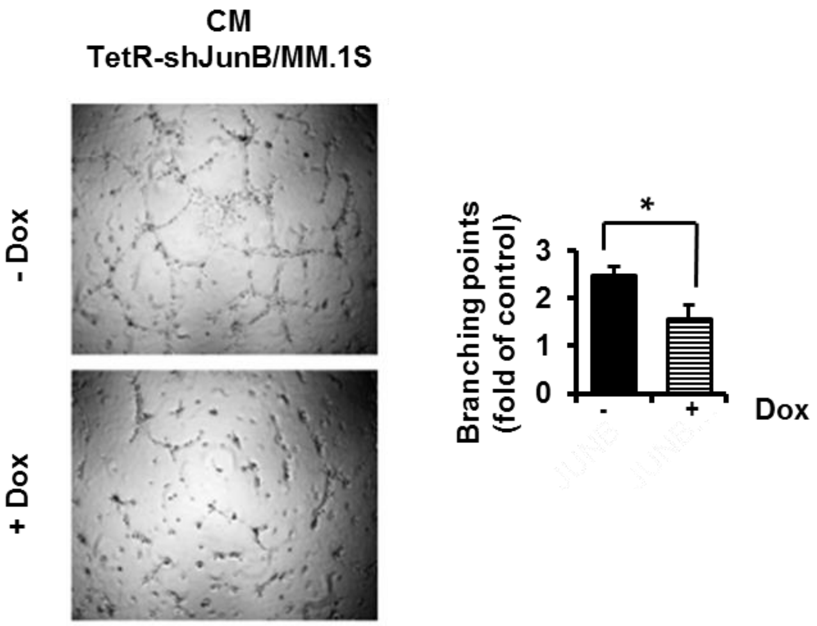

Supplementary Figure 12: Supernatant derived from MM cells upon JunB- knockdown inhibits endothelial tubule formation. TetR-shJunB/ MM cells were cultured in RPMI-1640 medium with IL6 in the presence or absence of doxycycline (1 µg/ml). Conditioned media (CM) were collected after 24 hours and tubule formation was assessed after 4-6 hours using an inverted light microscope at 4-10x magnification. Photographs of tubule formation are representative of three independent experiments (left panel). The branching points of endothelial tubule were counted independently by two individuals in several random view fields/ wells, values were averaged, and statistically significant differences were measured using the Student's *t*-test. Data represent mean ± SD for triplicate samples. * $p < 0.01$ (right panel).

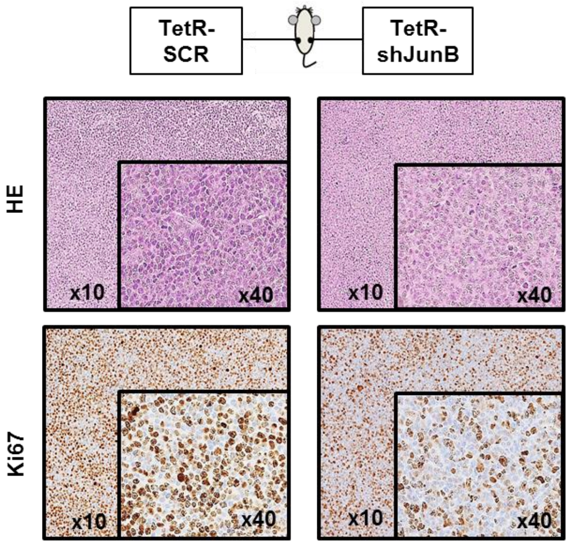

Supplementary Figure 13: Induced JunB silencing inhibits tumor growth in a murine xenograft MM model. TetR-SCR/ MM.1S or TetR-shJunB/ MM.1S were injected subcutaneously together with human-derived BMSCs and matrigel into the left and right flanks of mice, respectively, and fed with doxycycline in their drinking water for 5 weeks. Representative microscopic images of tumor sections stained with hematoxylin and eosin (HE) and Ki-67 are shown.

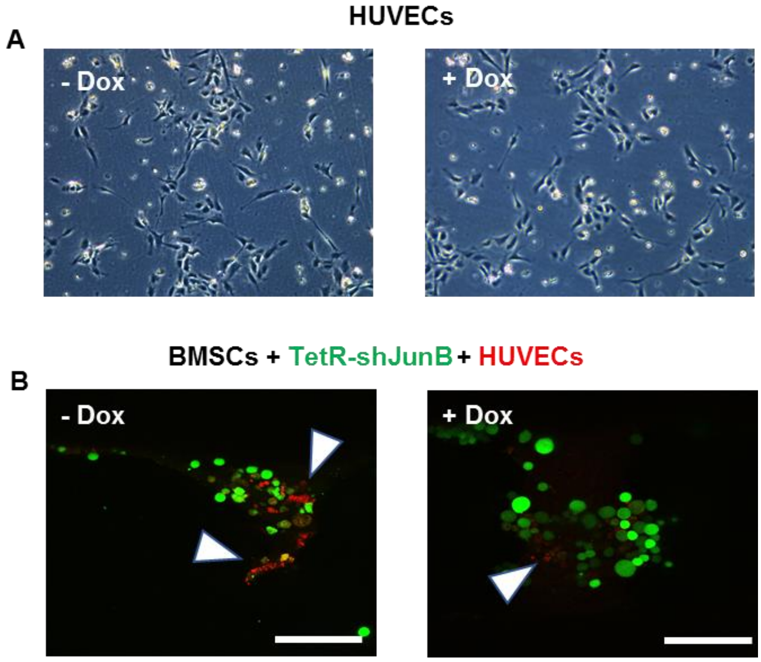

Supplementary Figure 14: (A) Doxycycline is not toxic for HUVECs. HUVECs were cultured for 5 days and treated with doxycycline or left untreated. Representative images of HUVECs. **(B)** Representative Z-stack confocal images of GFP+ Tet-shJunB/ MM.1S cells (green) together with stroma cells, and fluorescence-tagged HUVECs (Red) (40x magnification). Scale bars 100 μm.

Supplementary Tables

Supplementary Table 1: Primers used for quantitative real-time PCR.

Gene	Forward (5'-3')	Reverse (5'-3')
VEGF	CTACCTCCACCATGCCAAGT	GCAGTAGCTGCGCTGATAGA
VEGFB	GACATCACCCATCCCACTCCA	TCTGAAAAGCCATGTGTCACC
IGF1	ATGCTCTTCAGTTCGTGTGTG	GGGTCTTGGGCATGTCGGTG
B2M	TGCTGTCTCCATGTTTGATGTATCT	TCTCTGCTCCCCACCTCTAAGT

B2M: beta-2-microglobulin.

Supplementary Table 2. Prediction of JunB-bound regions at the VEGF, VEGFB, and IGF1 loci. With PROMO and JASPAR, two transcription factor binding site open source databases and prediction programs, we identified putative JunB binding sites in the promoter regions (-2000 bp to -1 bp) of VEGF, VEGFB, and IGF1 genes. All of the position numbers are indicated relative to the transcription start site as +1.

VEGF	Start	End	Predicted site sequence	Public datasets
1	-1131	-1120	TTTGAATCATCA	JASPAR
2	-940	-930	CACTGACTAAC	JASPAR, PROMO
3	-493	-483	GGGTGAGTGAG	JASPAR
**VEGFB**	**Start**	**End**	**Predicted site sequence**	**Public datasets**
1	-1661	-1651	GTGTGGTCAGC	PROMO
2	-706	-694	TTGTGACCCATTG	JASPAR, PROMO
**IGF1**	**Start**	**End**	**Predicted site sequence**	**Public datasets**
1	-1780	-1771	CATTACACAT	JASPAR
2	-1138	-1126	CTGTGAGTCAGTG	JASPAR, PROMO
3	-629	-619	GGATTACTCAC	JASPAR

Supplementary Table 3: Information of MM patients.

Pts No.	Gender	Age	Treatment	R-ISS	S&D	Cytogenetics	JunB expression (%)	MVD
1	Male	51	Newly diagnosed	III	III B	del13q14<br>del1p32<br>del8p<br>t(6;14)	11.2	Low
2	Male	62	Newly diagnosed	n/a	n/a	n/a	2.7	Low
3	Male	67	Newly diagnosed	III	III B	del17p13<br>1q21ampl<br>del1p32<br>6q23ampl<br>t(14q32)	60.4	High
4	Female	70	Newly diagnosed	III	n/a	del13q14<br>del17p13<br>1q21ampl	46.2	High
5	Female	73	Newly diagnosed (relapsed later on)	n/a	III A	1q21ampl<br>6q23	91.9	High
6	Male	58	Newly diagnosed	III	III A	del1p32<br>1q21ampl<br>6q23ampl	35.2	Medium
7	Female	54	Newly diagnosed	n/a	III B	n/a	18.9	Medium
8	Female	50	Newly diagnosed	n/a	II A	n/a	37.3	Medium
9	Male	69	Newly diagnosed (extramedullary MM relapsed later on)	III	III A	n/a	39	Medium
10	Female	60	Newly diagnosed	I	III A	6q23ampl	1.4	Low

R-ISS: Revised International Staging System; S&D: Durie-Salmon Staging System; MVD: microvessel density; n/a: not applicable.

Supplementary Table 4. Unique JunB binding peaks identified by ChIP-seq.

Supplementary Table 5. Unique JunB binding targets identified by ChIP-seq. ENSEMBL Gene ID was converted to Gene Symbol and annotated using Metascape (version of database: updated on 2020-09-16, setting: Express Analysis).

Supplementary Table 6. KEGG pathway and GO biological processes enrichment analysis of JunB and its target AFs, as well as each MCODE cluster in **Suppl. Figure 11B-D** using Metascape.

Supplementary Table 7. JASPAR analysis of putative JunB binding sites in the peak sequences from ChIP-seq results related to **Figure 4E**.
