# Supplementary material for: JunB is a key regulator of multiple myeloma bone marrow angiogenesis
Source: Leukemia. 2021 May 18;35(12):3509–25. doi: 10.1038/s41375-021-01271-9 (PMC8632680; doi:10.1038/s41375-021-01271-9)
Supplement: Supplementary file 2 — Supplementary Table 4 (Unique JunB binding peaks identified by ChIP-seq.) [file 41375_2021_1271_MOESM2_ESM.pdf]

Chromosome	Peak Start	Peak End	Length (bp)	Abs Summit	-LOG10 (pvalue)	Fold Enrichment	-LOG10 (qvalue)	Gene Feature	Ensembl Gene ID	Gene Start-Gene End	Strand	Distance to TSS (bp)
1	2971037	2971400	364	2971180	4.1849	3.24157	0.52478	intergenic	ENSG00000284745	2960657-2968707	-	-2511
1	3289642	3289876	235	3289789	3.20396	2.64557	0.26167	ENSG00000142611:intron	ENSG00000272235	3306635-3310096	-	20337
1	3329841	3330049	209	3329988	6.35124	4.29576	1.83857	ENSG00000142611:intron	ENSG00000272235	3306635-3310096	-	-19848
1	4359447	4359769	323	4359475	3.34993	2.92244	0.30123	intergenic	ENSG00000235054	4412026-4424689	+	-52418
1	5692316	5692594	279	5692560	4.56295	3.60388	0.59373	intergenic	ENSG00000236948	5561708-5668295	-	-24159
1	6784416	6784700	285	6784563	11.53275	5.55571	6.4016	ENSG00000171735:Promoter:ENSG00000237436:intron	ENSG00000237436	6783891-6784843	-	285
1	7242454	7242662	209	7242619	5.39424	4.00432	1.09512	ENSG00000171735:intron	ENSG00000207056	7219359-7219524	+	23198
1	12051997	12052314	318	12052162	10.64217	4.64231	5.60051	intergenic	ENSG00000120949	12063376-12144207	+	-11221
1	12161875	12162130	256	12162017	12.44681	6.03265	7.23865	intergenic	ENSG00000283789	12166942-12167038	+	-4940
1	12264651	12264857	207	12264657	3.64836	3.01523	0.30123	ENSG00000048707:intron	ENSG00000048707	12230029-12512047	+	34724
1	12303332	12303669	338	12303642	3.77589	3.20345	0.30123	ENSG00000048707:intron	ENSG00000048707	12230029-12512047	+	73471
1	13589779	13590158	380	13589880	3.56795	3.06614	0.30123	ENSG00000162493:intron	ENSG00000162493	13583464-13617957	+	6504
1	14040070	14040339	270	14040193	10.33373	5.50729	5.31704	ENSG00000189337:intron	ENSG00000252151	14124232-14124335	-	84131
1	15126745	15126962	218	15126855	3.3168	2.90065	0.30123	ENSG00000175147:intron	ENSG00000171729	15152531-15220480	+	-25678
1	15150562	15150772	211	15150643	3.46407	2.89822	0.30123	ENSG00000175147:intron;ENSG00000171729:Promoter	ENSG00000171729	15152531-15220480	+	-1864
1	15571878	15572187	310	15572019	5.25679	3.77493	1.07467	ENSG00000116138:intron	ENSG00000272510	15565610-15565956	-	-6076
1	17397618	17397950	333	17397867	4.00982	3.24654	0.45978	ENSG00000276747:intron	ENSG00000227751	17406759-17407382	+	-8975
1	20109449	20109800	352	20109636	6.26578	4.40475	1.75945	intergenic	ENSG00000117215	20111938-20119566	-	9942
1	21294648	21294881	234	21294711	5.84361	3.7038	1.44168	ENSG00000117298:intron;ENSG00000231105:intron	ENSG00000231105	21293289-21299774	+	1475
1	22025829	22026114	286	22025981	15.77144	6.65577	10.31754	ENSG00000218510:intron;ENSG00000228397:exon;ENSG00000285721:intron;ENSG00000285959:intron	ENSG00000228397	22023989-22026048	-	77
1	22822567	22822831	265	22822714	6.49439	4.38367	1.95029	ENSG00000133216:intron	ENSG00000225952	22835712-22836849	-	14150
1	23150847	23151175	329	23151087	3.94601	3.20555	0.42361	ENSG00000169641:intron	ENSG00000229010	23140324-23141142	+	10686
1	24541107	24541418	312	24541125	4.91514	3.56573	0.82412	ENSG00000264443:intron	ENSG00000264443	24538801-24556024	-	14762
1	25022891	25023159	269	25023007	7.56656	4.19944	2.85958	ENSG00000264371:Promoter	ENSG00000264371	25023502-25023586	+	-477
1	25883731	25883975	245	25883849	5.01585	3.49609	0.89533	intergenic	ENSG00000229786	25887359-25887610	+	-3506
1	27952486	27952709	224	27952512	4.02276	3.25486	0.46996	ENSG00000130768:intron;ENSG00000227050:intron	ENSG00000158156	27959461-27968096	+	-6864
1	27973524	27973765	242	27973588	4.33895	3.45881	0.59373	ENSG00000158161:exon;ENSG00000158161:three_prime UTR	ENSG00000158156	27959461-27968096	+	14183
1	28601680	28602024	345	28601856	4.64049	3.52611	0.64891	intergenic	ENSG00000188060	28592199-28595443	+	9652
1	29896723	29896942	220	29896837	4.88847	3.6826	0.81688	intergenic	ENSG00000270927	29904864-29905357	-	8525
1	30361776	30362011	236	30361988	4.03577	3.26323	0.47041	intergenic	ENSG00000236335	30409559-30411638	-	49745
1	36386512	36386761	250	36386615	12.08765	6.27606	6.91951	ENSG00000196182:Promoter	ENSG00000196182	36339623-36385896	-	-740
1	37739190	37739541	352	37739428	9.78373	4.69287	4.83465	ENSG00000200421:Promoter:ENSG00000183317:intron	ENSG00000200421	37737954-37738054	-	-1311
1	39394675	39394946	272	39394764	11.10484	6.16387	6.01942	ENSG00000127603:intron	ENSG00000217897	39304293-39304898	-	-89912
1	44575013	44575288	276	44575185	3.29424	2.88581	0.30123	ENSG00000187147:intron	ENSG00000263381	44545492-44545552	+	29658
1	44713312	44713567	256	44713463	8.18881	4.69185	3.39457	ENSG00000198520:intron	ENSG00000199377	44721785-44721902	-	8463
1	47211181	47211438	258	47211324	3.99693	3.23826	0.45978	intergenic	ENSG00000226252	47225796-47230750	+	-14487
1	48312813	48313177	365	48313044	3.77589	3.20345	0.30123	ENSG00000132122:intron	ENSG00000224986	48325079-48326123	-	13128
1	48967553	48967950	398	48967754	4.56295	3.60388	0.59373	ENSG00000186094:intron	ENSG00000229846	49025594-49187585	+	-57843

1	49184249	49184550	302	49184433	4.56295	3.60388	0.59373	ENSG00000186094:intron;ENSG00000229846:intron	ENSG00000230114	49257410-49269285	+	-73011
1	49293043	49293333	291	49293127	3.77589	3.20345	0.30123	ENSG00000186094:intron	ENSG00000230114	49257410-49269285	+	35777
1	49339114	49339332	219	49339236	6.09579	4.29768	1.6498	ENSG00000186094:intron	ENSG00000230114	49257410-49269285	+	81812
1	49184249	49184550	302	49184433	4.56295	3.60388	0.59373	ENSG00000186094:intron;ENSG00000229846:intron	ENSG00000230114	49257410-49269285	+	-73011
1	49293043	49293333	291	49293127	3.77589	3.20345	0.30123	ENSG00000186094:intron	ENSG00000230114	49257410-49269285	+	35777
1	49339114	49339332	219	49339236	6.09579	4.29768	1.6498	ENSG00000186094:intron	ENSG00000230114	49257410-49269285	+	81812
1	49764924	49765159	236	49765017	4.62651	3.51732	0.63659	ENSG00000186094:intron	ENSG00000237478	49691261-49691523	−	-73518
1	50254961	50255430	470	50255272	4.56295	3.60388	0.59373	ENSG00000284645:intron;ENSG00000284696:intron	ENSG00000284645	50252568-50258994	+	2627
1	53472272	53472542	271	53472363	4.86328	3.5342	0.79494	intergenic	ENSG00000143006	53459398-53467488	+	13008
1	56525593	56525975	383	56525654	5.58512	3.97816	1.25272	ENSG00000162407:intron	ENSG00000284686	56173432-56524495	−	-1288
1	58784024	58784272	249	58784143	10.65162	5.29115	5.60305	ENSG00000234807:Promoter;ENSG00000177606:five_prime_UTR;ENSG00000177606:exon	ENSG00000177606	58780787-58784327	−	179
1	59020216	59020589	374	59020391	14.11396	6.05711	8.77833	ENSG00000237352:intron	ENSG00000228121	58999675-59000494	+	20727
1	59123968	59124225	258	59124195	4.16942	3.34931	0.52478	intergenic	ENSG00000235215	59055998-59078116	−	-45980
1	60899141	60899467	327	60899312	10.09471	6.00647	5.09428	ENSG00000162599:intron	ENSG00000231252	60659630-60867998	−	-31305
1	61318803	61319129	327	61318968	12.20111	6.5755	7.01985	ENSG00000162599:intron	ENSG00000237853	61248944-61253510	−	-65455
1	62426992	62427437	446	62427105	3.49438	2.91742	0.30123	intergenic	ENSG00000162607	62436296-62451804	+	-9082
1	62959254	62959630	377	62959556	7.17425	4.80518	2.52654	intergenic	ENSG00000237163	62905179-62905482	+	54262
1	65486736	65486988	253	65486848	4.56295	3.60388	0.59373	ENSG00000116678:intron;ENSG00000237852:intron	ENSG00000237852	65486405-65494188	+	456
1	65812041	65812372	332	65812168	11.53275	5.55571	6.4016	ENSG00000184588:intron	ENSG00000184588	65792513-66374579	+	19693
1	66013462	66013697	236	66013467	3.72445	3.16947	0.30123	ENSG00000184588:intron	ENSG00000227466	66042499-66050718	−	37139
1	66119258	66119598	341	66119490	4.1849	3.24157	0.52478	ENSG00000184588:intron	ENSG00000223152	66094460-66094546	−	-24881
1	66860820	66861164	345	66861009	6.19166	4.35803	1.7362	ENSG00000152763:intron	ENSG00000231080	66826941-66828558	+	34050
1	67013568	67013895	328	67013793	10.81138	6.21673	5.74678	ENSG00000116704:intron	ENSG00000116704	66999331-67054099	−	40368
1	67037590	67037801	212	67037653	4.31475	3.20475	0.59373	ENSG00000116704:intron	ENSG00000116704	66999331-67054099	−	16404
1	67339645	67339973	329	67339826	4.07388	3.28775	0.47041	ENSG00000081985:intron	ENSG00000081985	67307363-67397090	+	32445
1	67495903	67496120	218	67496084	3.30549	2.89321	0.30123	intergenic	ENSG00000235200	67522346-67532612	+	-26335
1	67610296	67610999	704	67610610	5.10865	3.82229	0.9653	intergenic	ENSG00000242482	67656830-67657129	−	46482
1	75249625	75249911	287	75249656	4.94982	3.72146	0.84841	ENSG00000137968:intron	ENSG00000206999	75183044-75183147	+	66723
1	75265789	75266007	219	75265957	3.77589	3.20345	0.30123	ENSG00000137968:intron	ENSG00000206999	75183044-75183147	+	82853
1	76270600	76270882	283	76270638	4.56295	3.60388	0.59373	ENSG00000184005:intron	ENSG00000223905	76353582-76354246	−	83505
1	78383564	78384098	535	78383969	4.07388	3.28775	0.47041	ENSG00000122420:intron	ENSG00000212308	78375163-78375268	−	-8562
1	79892705	79892927	223	79892837	5.87556	4.15944	1.46959	intergenic	ENSG00000285409	79967732-80051404	−	158588
1	79926089	79926311	223	79926261	3.77589	3.20345	0.30123	intergenic	ENSG00000285409	79967732-80051404	−	125204
1	82346279	82346554	276	82346495	3.77589	3.20345	0.30123	ENSG00000233290:intron	ENSG00000233290	82212412-82848205	−	501789
1	82799425	82799637	213	82799550	5.39424	4.00432	1.09512	ENSG00000233290:intron	ENSG00000233290	82212412-82848205	−	48674
1	82831029	82831267	239	82831238	4.56295	3.60388	0.59373	ENSG00000233290:intron	ENSG00000233290	82212412-82848205	−	17057
1	85977685	85978048	364	85977797	5.6497	4.01836	1.29426	ENSG00000171502:intron	ENSG00000229505	86029853-86030589	+	-51987
1	86560966	86561320	355	86561111	6.02088	4.25059	1.59152	ENSG00000016602:intron	ENSG00000224466	86552624-86553342	−	-7800
1	87312515	87312737	223	87312533	4.29558	3.43077	0.59373	intergenic	ENSG00000143013	87328467-87348923	+	-15841
1	89054614	89054861	248	89054717	5.50408	3.92781	1.18712	ENSG00000117228:exon	ENSG00000117228	89052318-89065360	−	10623
1	90088954	90089171	218	90089136	4.919	3.70193	0.82412	intergenic	ENSG00000162664	89995111-90035531	+	93951
1	90490782	90491102	321	90490958	3.61899	3.09983	0.30123	intergenic	ENSG00000229201	90510909-90533472	−	42530
1	107936516	107936770	255	107936623	8.1168	5.20561	3.35333	ENSG00000134215:intron	ENSG00000230489	107964442-107994607	+	-27799
1	125180102	125180310	209	125180205	416.09158	2.03467	406.90308	intergenic	ENSG00000224857	121573945-121580524	−	-3599681

1	125183667	125183883	217	125183785	25.70222	1.96749	19.87808	intergenic	ENSG00000224857	121573945-121580524	-	-3603250
1	125180102	125180310	209	125180205	416.09158	2.03467	406.90308	intergenic	ENSG00000224857	121573945-121580524	-	-3599681
1	125183667	125183883	217	125183785	25.70222	1.96749	19.87808	intergenic	ENSG00000224857	121573945-121580524	-	-3603250
1	125180102	125180310	209	125180205	416.09158	2.03467	406.90308	intergenic	ENSG00000224857	121573945-121580524	-	-3599681
1	125183667	125183883	217	125183785	25.70222	1.96749	19.87808	intergenic	ENSG00000224857	121573945-121580524	-	-3603250
1	125180102	125180310	209	125180205	416.09158	2.03467	406.90308	intergenic	ENSG00000224857	121573945-121580524	-	-3599681
1	125183667	125183883	217	125183785	25.70222	1.96749	19.87808	intergenic	ENSG00000224857	121573945-121580524	-	-3603250
1	146793758	146793984	227	146793808	3.77589	3.20345	0.30123	ENSG00000276975:intron	ENSG00000274450	146862728-146862845	-	68974
1	147438254	147438477	224	147438394	4.2966	3.31094	0.59373	ENSG00000278811:intron	ENSG00000272443	147445578-147446133	+	-7213
1	147549539	147549797	259	147549690	10.40398	5.34918	5.38351	ENSG00000116128:intron	ENSG00000116128	147541411-147626216	+	8256
1	149939122	149939521	400	149939281	6.8151	3.95242	2.22092	ENSG00000264522:three_prime_UTR;ENSG00000264522:exon	ENSG00000014914	149928650-149936869	-	-2452
1	150584894	150585223	330	150585186	4.11506	3.19834	0.49781	intergenic	ENSG00000143384	150574550-150579738	-	-5320
1	150613305	150613779	475	150613719	4.93372	3.32789	0.83448	ENSG00000143420:intron	ENSG00000276103	150608506-150608623	-	-4918
1	152048143	152048417	275	152048259	7.57779	3.50621	2.86844	ENSG00000163191:Promoter	ENSG00000163191	152032505-152047907	-	-372
1	152438152	152438410	259	152438185	4.83688	3.51817	0.77787	intergenic	ENSG00000143536	152409242-152414263	-	-24017
1	152438152	152438410	259	152438185	4.83688	3.51817	0.77787	intergenic	ENSG00000143536	152409242-152414263	-	-24017
1	155301129	155301375	247	155301198	5.75051	3.78743	1.36222	ENSG00000143627:intron	ENSG00000143627	155289292-155301438	-	186
1	157620322	157620597	276	157620488	3.76336	3.08858	0.30123	intergenic	ENSG00000236731	157629938-157630728	-	10269
1	157626031	157626526	496	157626233	6.43548	4.18632	1.91127	intergenic	ENSG00000236731	157629938-157630728	-	4450
1	157842889	157843164	276	157843038	7.52827	4.49198	2.82287	ENSG00000073754:intron	ENSG00000227425	157861196-157861460	+	-18170
1	158248070	158248404	335	158248209	6.4824	4.21404	1.94623	intergenic	ENSG00000158477	158254136-158258269	+	-5899
1	158621887	158622132	246	158622073	3.78699	3.10368	0.30881	ENSG00000163554:intron	ENSG00000198967	158605267-158612514	+	16742
1	158819698	158819939	242	158819827	8.96392	5.12897	4.09818	intergenic	ENSG00000180409	158808398-158809335	-	-10483
1	158847133	158847706	574	158847567	3.49438	2.91742	0.30123	ENSG00000163563:intron	ENSG00000163563	158831316-158849506	+	16103
1	158910132	158910353	222	158910300	5.68188	3.88989	1.31322	intergenic	ENSG00000163564	158930795-158977054	+	-20553
1	158952699	158953045	347	158952887	6.4824	4.21404	1.94623	ENSG00000163564:intron	ENSG00000163564	158930795-158977054	+	22076
1	159015605	159015822	218	159015671	5.95396	3.63426	1.53177	ENSG00000163565:intron	ENSG00000163565	158999967-159055155	+	15746
1	159031248	159031632	385	159031454	4.78394	3.61651	0.75881	ENSG00000163565:intron	ENSG00000163565	158999967-159055155	+	31472
1	159015605	159015822	218	159015671	5.95396	3.63426	1.53177	ENSG00000163565:intron	ENSG00000163565	158999967-159055155	+	15746
1	159031248	159031632	385	159031454	4.78394	3.61651	0.75881	ENSG00000163565:intron	ENSG00000163565	158999967-159055155	+	31472
1	159068171	159068572	402	159068425	7.46137	4.62446	2.76343	ENSG00000163568:intron	ENSG00000233473	159081132-159081795	-	13424
1	159089080	159089362	283	159089361	3.49438	2.91742	0.30123	ENSG00000163568:intron	ENSG00000233473	159081132-159081795	-	-7425
1	159068171	159068572	402	159068425	7.46137	4.62446	2.76343	ENSG00000163568:intron	ENSG00000233473	159081132-159081795	-	13424
1	159089080	159089362	283	159089361	3.49438	2.91742	0.30123	ENSG00000163568:intron	ENSG00000233473	159081132-159081795	-	-7425
1	159142155	159142449	295	159142156	3.20396	2.64557	0.26167	ENSG00000163568:intron	ENSG00000163568	159062483-159147096	-	4794
1	159360936	159361187	252	159360986	3.53832	2.94528	0.30123	ENSG00000228560:intron	ENSG00000251686	159366160-159367089	+	-5099
1	159720489	159720692	204	159720587	5.77031	4.0936	1.37979	intergenic	ENSG00000132693	159712288-159714589	-	-6001
1	159728083	159728438	356	159728159	3.90845	3.18146	0.39391	intergenic	ENSG00000132693	159712288-159714589	-	-13671
1	159890021	159890234	214	159890129	5.17085	3.34134	1.01289	ENSG00000213085:intron	ENSG00000266458	159899978-159900079	-	9952
1	162109919	162110314	396	162109983	5.99467	4.07842	1.56946	ENSG00000198929:intron	ENSG00000198929	162069773-162370475	+	40343
1	163852214	163852461	248	163852293	4.76929	3.60726	0.74587	intergenic	ENSG00000212538	163923669-163923873	+	-71332
1	163917089	163917338	250	163917095	3.23881	2.84938	0.2852	intergenic	ENSG00000212538	163923669-163923873	+	-6456
1	164105399	164105633	235	164105422	5.07624	3.80169	0.93914	intergenic	ENSG00000212538	163923669-163923873	+	181846
1	163852214	163852461	248	163852293	4.76929	3.60726	0.74587	intergenic	ENSG00000212538	163923669-163923873	+	-71332
1	163917089	163917338	250	163917095	3.23881	2.84938	0.2852	intergenic	ENSG00000212538	163923669-163923873	+	-6456
1	164105399	164105633	235	164105422	5.07624	3.80169	0.93914	intergenic	ENSG00000212538	163923669-163923873	+	181846
1	164817968	164818248	281	164818239	4.55755	3.47401	0.59373	ENSG00000185630:intron	ENSG00000271917	164828435-164829952	+	-10327
1	165365685	165365954	270	165365795	4.82831	3.64454	0.77787	intergenic	ENSG00000162761	165201866-165356715	-	-9104
1	165727071	165727315	245	165727239	3.32816	2.90812	0.30123	intergenic	ENSG00000273365	165706555-165707284	-	-19908

1	165827530	165827836	307	165827749	3.20594	2.48841	0.26185	ENSG00000143179:Promoter;ENSG00000143183:five_prime_UTR;ENSG00000143183:exon	ENSG00000143183	165724292-165827755	-	72
1	168144632	168144902	271	168144747	3.45699	2.99296	0.30123	intergenic	ENSG00000143147	168079542-168137667	-	-7099
1	172254973	172255215	243	172255038	5.13473	3.43925	0.98202	ENSG00000197959:intron	ENSG00000230630	172138396-172144840	-	-110253
1	173283421	173283648	228	173283636	4.1849	3.24157	0.52478	intergenic	ENSG00000232751	173351688-173352004	+	-68154
1	174057638	174057891	254	174057812	3.32816	2.90812	0.30123	intergenic	ENSG00000225713	174090135-174090474	-	32710
1	174841967	174842209	243	174842009	6.38722	4.15784	1.8702	ENSG00000152061:intron	ENSG00000231378	174849666-174850168	-	8080
1	175263610	175263841	232	175263750	3.6499	2.91341	0.30123	intergenic	ENSG00000233946	175297079-175297240	+	-33354
1	176067253	176067477	225	176067385	5.56876	3.96799	1.238	ENSG00000143207:intron	ENSG00000228686	176017276-176018760	+	50088
1	177582118	177582339	222	177582192	7.79913	5.0097	3.0731	ENSG00000227579:intron	ENSG00000227579	177393286-177597709	-	15481
1	177686314	177686557	244	177686492	4.29558	3.43077	0.59373	intergenic	ENSG00000236720	177700523-177710330	+	-14088
1	177700413	177700693	281	177700648	4.1849	3.24157	0.52478	ENSG00000236720:exon	ENSG00000236720	177700523-177710330	+	29
1	177800612	177800958	347	177800761	3.14236	2.78607	0.22463	intergenic	ENSG00000236720	177700523-177710330	+	100261
1	177686314	177686557	244	177686492	4.29558	3.43077	0.59373	intergenic	ENSG00000236720	177700523-177710330	+	-14088
1	177700413	177700693	281	177700648	4.1849	3.24157	0.52478	ENSG00000236720:exon	ENSG00000236720	177700523-177710330	+	29
1	177800612	177800958	347	177800761	3.14236	2.78607	0.22463	intergenic	ENSG00000236720	177700523-177710330	+	100261
1	179678527	179678811	285	179678660	4.00982	3.24654	0.45978	ENSG00000162782:intron	ENSG00000243062	179730216-179742697	+	-51547
1	179899322	179899640	319	179899579	3.4812	3.00892	0.30123	ENSG00000143337:intron;ENSG00000264916:Promoter	ENSG00000264916	179900261-179900564	+	-780
1	183956696	183957010	315	183956851	8.06337	4.79751	3.31375	ENSG00000198756:intron	ENSG00000198756	183929853-184037729	-	80876
1	183959987	183960229	243	183960058	5.04416	3.78131	0.91864	ENSG00000198756:intron	ENSG00000198756	183929853-184037729	-	77621
1	183992969	183993229	261	183993189	5.40925	3.86905	1.10135	ENSG00000198756:intron	ENSG00000198756	183929853-184037729	-	44630
1	183956696	183957010	315	183956851	8.06337	4.79751	3.31375	ENSG00000198756:intron	ENSG00000198756	183929853-184037729	-	80876
1	183959987	183960229	243	183960058	5.04416	3.78131	0.91864	ENSG00000198756:intron	ENSG00000198756	183929853-184037729	-	77621
1	183992969	183993229	261	183993189	5.40925	3.86905	1.10135	ENSG00000198756:intron	ENSG00000198756	183929853-184037729	-	44630
1	184331002	184331225	224	184331123	3.95865	3.21367	0.43404	ENSG00000231791:intron	ENSG00000231791	184329070-184332826	+	2043
1	184844019	184844277	259	184844129	4.84177	3.65305	0.77787	ENSG00000135842:intron	ENSG00000252222	184821427-184821489	-	-22658
1	184994172	184994438	267	184994256	3.49438	2.91742	0.30123	intergenic	ENSG00000260976	185001526-185008683	+	-7221
1	185306117	185306478	362	185306288	3.97135	3.22182	0.44441	ENSG00000116679:intron	ENSG00000116679	185296387-185317329	-	11032
1	185614177	185614446	270	185614285	6.17229	4.34582	1.71923	ENSG00000228309:intron	ENSG00000228309	185558371-185628527	-	14216
1	185617800	185618011	212	185617845	5.39424	4.00432	1.09512	ENSG00000228309:intron	ENSG00000228309	185558371-185628527	-	10622
1	186434641	186434856	216	186434659	4.07388	3.28775	0.47041	ENSG00000229739:Promoter	ENSG00000229739	186435160-186470291	+	-412
1	187015864	187016075	212	187015895	4.32442	3.44941	0.59373	intergenic	ENSG00000230426	187070699-187686622	+	-54730
1	187042323	187042623	301	187042406	6.74377	4.53757	2.15867	intergenic	ENSG00000230426	187070699-187686622	+	-28226
1	187172468	187172752	285	187172638	5.39424	4.00432	1.09512	ENSG00000230426:intron	ENSG00000230426	187070699-187686622	+	101910
1	187185461	187185753	293	187185576	8.53333	5.2656	3.7098	ENSG00000230426:intron	ENSG00000230426	187070699-187686622	+	114907
1	187197278	187197770	493	187197389	4.1849	3.24157	0.52478	ENSG00000230426:intron	ENSG00000230426	187070699-187686622	+	126824
1	187279428	187279697	270	187279496	3.34993	2.92244	0.30123	ENSG00000230426:intron	ENSG00000230426	187070699-187686622	+	208863
1	187015864	187016075	212	187015895	4.32442	3.44941	0.59373	intergenic	ENSG00000230426	187070699-187686622	+	-54730
1	187042323	187042623	301	187042406	6.74377	4.53757	2.15867	intergenic	ENSG00000230426	187070699-187686622	+	-28226
1	187172468	187172752	285	187172638	5.39424	4.00432	1.09512	ENSG00000230426:intron	ENSG00000230426	187070699-187686622	+	101910
1	187185461	187185753	293	187185576	8.53333	5.2656	3.7098	ENSG00000230426:intron	ENSG00000230426	187070699-187686622	+	114907
1	187197278	187197770	493	187197389	4.1849	3.24157	0.52478	ENSG00000230426:intron	ENSG00000230426	187070699-187686622	+	126824
1	187279428	187279697	270	187279496	3.34993	2.92244	0.30123	ENSG00000230426:intron	ENSG00000230426	187070699-187686622	+	208863
1	187015864	187016075	212	187015895	4.32442	3.44941	0.59373	intergenic	ENSG00000230426	187070699-187686622	+	-54730
1	187042323	187042623	301	187042406	6.74377	4.53757	2.15867	intergenic	ENSG00000230426	187070699-187686622	+	-28226
1	187172468	187172752	285	187172638	5.39424	4.00432	1.09512	ENSG00000230426:intron	ENSG00000230426	187070699-187686622	+	101910
1	187185461	187185753	293	187185576	8.53333	5.2656	3.7098	ENSG00000230426:intron	ENSG00000230426	187070699-187686622	+	114907
1	187197278	187197770	493	187197389	4.1849	3.24157	0.52478	ENSG00000230426:intron	ENSG00000230426	187070699-187686622	+	126824

1	187279428	187279697	270	187279496	3.34993	2.92244	0.30123	ENSG00000230426:intron	ENSG00000230426	187070699-187686622	+	208863
1	188610998	188611217	220	188611069	4.56295	3.60388	0.59373	intergenic	ENSG00000235083	188671352-188671759	+	-60245
1	189316939	189317246	308	189317119	4.07388	3.28775	0.47041	intergenic	ENSG00000226196	189132349-189133292	-	-183800
1	192092218	192092442	225	192092371	4.91514	3.56573	0.82412	intergenic	ENSG00000150681	192158456-192185815	+	-66126
1	192952071	192952331	261	192952261	4.31475	3.20475	0.59373	intergenic	ENSG00000285280	192167785-192957713	-	5512
1	193171469	193171685	217	193171499	4.33895	3.45881	0.59373	ENSG00000134371:intron	ENSG00000162630	193179044-193186654	-	15077
1	193595586	193595805	220	193595609	4.35356	3.46825	0.59373	intergenic	ENSG00000226640	193678893-193727035	+	-83198
1	193609000	193609307	308	193609134	5.38044	3.99551	1.09512	intergenic	ENSG00000226640	193678893-193727035	+	-69740
1	193658230	193658453	224	193658422	4.87332	3.67301	0.80353	intergenic	ENSG00000226640	193678893-193727035	+	-20552
1	193595586	193595805	220	193595609	4.35356	3.46825	0.59373	intergenic	ENSG00000226640	193678893-193727035	+	-83198
1	193609000	193609307	308	193609134	5.38044	3.99551	1.09512	intergenic	ENSG00000226640	193678893-193727035	+	-69740
1	193658230	193658453	224	193658422	4.87332	3.67301	0.80353	intergenic	ENSG00000226640	193678893-193727035	+	-20552
1	194919149	194919462	314	194919239	6.26578	4.40475	1.75945	ENSG00000285718:intron	ENSG00000285718	194785516-194931310	-	12005
1	199381840	199382176	337	199382049	7.37304	4.74897	2.68538	ENSG00000231718:intron	ENSG00000236468	199387140-199388475	+	-5132
1	199546029	199546246	218	199546144	6.26578	4.40475	1.75945	intergenic	ENSG00000236468	199387140-199388475	+	158997
1	200866569	200866922	354	200866693	30.54666	9.93365	24.60392	intergenic	ENSG00000231333	200863807-200864152	+	2938
1	201842827	201843120	294	201843087	3.6499	2.91341	0.30123	ENSG00000198700:intron	ENSG00000231871	201688258-201829559	-	-13414
1	202909759	202910063	305	202909947	4.35972	3.35026	0.59373	ENSG00000117153:intron;ENSG00000230280:Promoter	ENSG00000230280	202911811-202912729	+	-1900
1	203381488	203381731	244	203381563	5.93384	3.89305	1.51725	intergenic	ENSG00000271588	203400265-203400581	-	18972
1	203607367	203607609	243	203607569	4.76422	3.12858	0.74587	intergenic	ENSG00000058668	203626560-203744081	+	-19072
1	203946015	203946276	262	203946212	6.04362	4.10812	1.61132	intergenic	ENSG00000237379	203954639-203955780	+	-8494
1	205584844	205585084	241	205585040	3.90052	3.06626	0.38783	ENSG00000174514:intron	ENSG00000206762	205595040-205595147	+	-10076
1	206050722	206051105	384	206050852	14.68026	5.94142	9.2998	ENSG00000263961:Promoter:ENSG00000285417:intron	ENSG00000263961	206052722-206102459	+	-1809
1	206784438	206784729	292	206784599	19.16017	7.883	13.561	intergenic	ENSG00000136634	206767601-206772494	-	-12089
1	207260994	207261262	269	207261188	4.04884	3.27164	0.47041	ENSG00000237074:intron	ENSG00000237074	207240121-207309292	+	21006
1	207269243	207269556	314	207269350	3.88371	3.16559	0.37576	ENSG00000237074:intron	ENSG00000237074	207240121-207309292	+	29278
1	207300868	207301107	240	207300992	6.06008	4.11811	1.6243	ENSG00000237074:intron	ENSG00000196352	207321375-207386804	+	-20388
1	207469515	207469767	253	207469604	4.06198	3.28009	0.47041	ENSG00000117322:intron	ENSG00000117322	207454229-207489895	+	15411
1	207603017	207603244	228	207603198	4.11506	3.19834	0.49781	ENSG00000203710:intron;ENSG00000236911:intron	ENSG00000236911	207551924-207606555	-	3425
1	207659711	207659941	231	207659767	4.35972	3.35026	0.59373	ENSG00000197721:intron	ENSG00000226289	207658453-207659142	+	1372
1	209312099	209312329	231	209312110	3.53038	3.04135	0.30123	intergenic	ENSG00000227940	209325455-209328532	+	-13241
1	209673372	209673648	277	209673457	7.40928	4.11883	2.71399	ENSG00000227591:intron;ENSG00000123689:Promoter	ENSG00000123689	209675419-209676388	+	-1909
1	209747762	209748058	297	209747890	13.05915	5.39155	7.81101	intergenic	ENSG00000236136	209744372-209745214	+	3537
1	211939906	211940179	274	211940092	8.98232	4.95279	4.10924	intergenic	ENSG00000233626	211936248-211936634	+	3794
1	211951978	211952204	227	211952070	9.22287	5.47958	4.30742	ENSG00000143493:intron	ENSG00000233626	211936248-211936634	+	15842
1	211939906	211940179	274	211940092	8.98232	4.95279	4.10924	intergenic	ENSG00000233626	211936248-211936634	+	3794
1	211951978	211952204	227	211952070	9.22287	5.47958	4.30742	ENSG00000143493:intron	ENSG00000233626	211936248-211936634	+	15842
1	212074590	212074849	260	212074715	6.40497	4.32872	1.88441	ENSG00000143476:intron	ENSG00000264358	212077612-212077685	+	-2893
1	212225885	212226404	520	212225967	4.04884	3.27164	0.47041	ENSG00000226251:intron	ENSG00000226251	212225277-212238977	-	12833
1	212426822	212427041	220	212426942	4.91672	3.56669	0.82412	intergenic	ENSG00000117691	212432886-212446379	+	-5955
1	214404229	214404495	267	214404371	6.57074	4.43068	2.0119	ENSG00000152104:intron	ENSG00000228470	214344171-214357615	-	-46746
1	215766737	215767038	302	215766794	4.42776	3.51628	0.59373	ENSG00000042781:intron	ENSG00000229242	215886581-215901464	+	-119694
1	215767997	215768311	315	215768093	4.50398	3.56566	0.59373	ENSG00000042781:intron	ENSG00000229242	215886581-215901464	+	-118427
1	215766737	215767038	302	215766794	4.42776	3.51628	0.59373	ENSG00000042781:intron	ENSG00000229242	215886581-215901464	+	-119694
1	215767997	215768311	315	215768093	4.50398	3.56566	0.59373	ENSG00000042781:intron	ENSG00000229242	215886581-215901464	+	-118427
1	217850303	217850664	362	217850545	5.39424	4.00432	1.09512	ENSG00000228247:exon;ENSG00000162814:intron	ENSG00000228247	217850402-217850633	-	150

1	218459964	218460264	301	218460100	23.87116	8.93408	18.10983	intergenic	ENSG00000281453	218442625-218443996	+	17488
1	219542450	219542671	222	219542508	6.49439	4.38367	1.95029	intergenic	ENSG00000238232	219557191-219557701	+	-14631
1	220611465	220611687	223	220611619	3.658	3.12558	0.30123	ENSG00000116141:intron	ENSG00000233012	220625739-220628056	+	-14163
1	221839874	221840129	256	221839994	11.51664	5.74538	6.39216	ENSG00000227925:intron	ENSG00000227925	221827665-221840666	-	665
1	221961741	221961974	234	221961845	11.51693	6.17716	6.39216	ENSG00000238042:intron	ENSG00000228437	221966340-221984964	+	-4483
1	222035410	222035794	385	222035755	3.4812	3.00892	0.30123	intergenic	ENSG00000232679	222041704-222064763	-	29161
1	223726750	223727041	292	223726869	14.34352	6.37517	8.98905	ENSG00000162909:intron	ENSG00000162909	223701592-223776018	+	25303
1	226662800	226663227	428	226662957	14.77784	5.80629	9.39293	ENSG00000228382:intron;ENSG00000143772:intron	ENSG00000228548	226668896-226676345	+	-5883
1	226711935	226712182	248	226712098	4.93372	3.32789	0.83448	ENSG00000143772:intron	ENSG00000143772	226631689-226739323	-	27265
1	227223334	227223661	328	227223459	4.91514	3.56573	0.82412	ENSG00000143776:intron	ENSG00000228729	227234268-227235355	+	-10771
1	229135283	229135539	257	229135397	5.63467	4.009	1.29399	intergenic	ENSG00000236975	229092814-229094905	-	-40505
1	229441074	229441441	368	229441215	18.30409	7.95497	12.73054	ENSG00000069248:three_prime_UTR;ENSG00000069248:exon;ENSG00000226920:Promoter	ENSG00000226920	229440283-229441020	-	-237
1	230084652	230084941	290	230084785	5.66875	3.88202	1.31047	ENSG00000143641:intron	ENSG00000143641	230057989-230282124	+	26807
1	230781639	230781884	246	230781788	7.34679	4.23292	2.66603	ENSG00000244137:intron;ENSG00000135773:intron	ENSG00000244137	230710697-230795492	-	13731
1	231224848	231225092	245	231225008	5.22474	3.89619	1.05478	ENSG00000143633:intron	ENSG00000143633	231223762-231241187	-	16217
1	233294964	233295212	249	233295093	8.13775	4.49748	3.3598	ENSG00000135749:intron	ENSG00000135749	232983434-233295713	-	625
1	234066380	234066601	222	234066408	4.07388	3.28775	0.47041	ENSG00000183780:intron	ENSG00000236101	234026850-234027227	-	-39263
1	234270724	234270947	224	234270845	5.48809	3.9179	1.17181	ENSG00000231272:intron;ENSG00000183780:intron;ENSG00000236244:intron	ENSG00000236244	234268582-234272500	-	1665
1	234608089	234608407	319	234608211	12.26758	5.7359	7.07326	ENSG00000228830:intron;ENSG00000168264:intron	ENSG00000228830	234607007-234609483	+	1240
1	234668576	234668832	257	234668685	7.77693	4.99605	3.05159	ENSG00000228044:intron	ENSG00000241475	234660270-234667104	+	8433
1	234772371	234772675	305	234772528	15.77144	6.65577	10.31754	intergenic	ENSG00000279261	234757618-234760056	-	-12466
1	234942203	234942425	223	234942346	3.88371	3.16559	0.37576	intergenic	ENSG00000237520	234957230-234959989	+	-14916
1	234960195	234960422	228	234960251	5.02628	3.63351	0.90528	ENSG00000238005:intron	ENSG00000237520	234957230-234959989	+	3078
1	234962779	234963004	226	234962927	4.47965	3.18758	0.59373	ENSG00000238005:intron	ENSG00000237520	234957230-234959989	+	5661
1	234942203	234942425	223	234942346	3.88371	3.16559	0.37576	intergenic	ENSG00000237520	234957230-234959989	+	-14916
1	234960195	234960422	228	234960251	5.02628	3.63351	0.90528	ENSG00000238005:intron	ENSG00000237520	234957230-234959989	+	3078
1	234962779	234963004	226	234962927	4.47965	3.18758	0.59373	ENSG00000238005:intron	ENSG00000237520	234957230-234959989	+	5661
1	235011703	235012078	376	235011866	21.00541	6.96346	15.3404	intergenic	ENSG00000258082	234979646-234980804	-	-31086
1	237174379	237174611	233	237174520	4.84177	3.65305	0.77787	ENSG00000198626:intron	ENSG00000252396	237120806-237121109	+	53688
1	238278423	238278716	294	238278667	4.07388	3.28775	0.47041	ENSG00000234464:intron	ENSG00000234464	238276036-238287016	+	2533
1	238289391	238289618	228	238289528	5.39424	4.00432	1.09512	intergenic	ENSG00000234464	238276036-238287016	+	13468
1	238296189	238296547	359	238296333	5.39424	4.00432	1.09512	intergenic	ENSG00000234464	238276036-238287016	+	20331
1	239915044	239915342	299	239915164	6.40497	4.32872	1.88441	intergenic	ENSG00000231979	239915438-239916955	-	1762
1	241928871	241929110	240	241929062	3.658	3.12558	0.30123	intergenic	ENSG00000235990	241916122-241916543	+	12868
1	242800960	242801253	294	242801056	4.56295	3.60388	0.59373	intergenic	ENSG00000215800	242772619-242773103	+	28487
1	243596398	243596715	318	243596569	12.09514	6.7541	6.92642	ENSG00000117020:intron	ENSG00000226766	243624665-243625056	-	28500
1	244135381	244135873	493	244135756	4.91514	3.56573	0.82412	intergenic	ENSG00000237759	244107364-244109011	+	28262
1	244628981	244629261	281	244629032	4.53506	3.5858	0.59373	ENSG00000179397:intron	ENSG00000121644	244652934-244709033	+	-23813
1	245686481	245686736	256	245686527	4.62651	3.51732	0.63659	ENSG00000162849:exon	ENSG00000231612	245673731-245676478	-	-10130
1	245891850	245892164	315	245892063	4.22478	3.38503	0.55558	ENSG00000185420:intron	ENSG00000235096	246025896-246035603	+	-133889
10	3758110	3758356	247	3758285	9.06429	5.18652	4.17525	ENSG00000229672:intron	ENSG00000229672	3751066-3763226	+	7166
10	3805852	3806078	227	3805998	7.62455	4.90256	2.91067	intergenic	ENSG00000067082	3775995-3785281	-	-20683

10	5720861	5721143	283	5721111	3.77589	3.20345	0.30123	ENSG00000226647:intron;ENSG00000108021:intron	ENSG00000226647	5712173-5744067	-	23065
10	6052643	6052940	298	6052815	18.70722	9.0361	13.12543	ENSG00000134460:intron	ENSG00000134460	6010688-6062370	-	9579
10	6141251	6141478	228	6141371	10.00381	5.52486	5.01796	intergenic	ENSG00000170525	6144933-6254644	+	-3569
10	10109903	10110237	335	10110062	4.56295	3.60388	0.59373	intergenic	ENSG00000224788	10058721-10063502	+	51348
10	12633806	12634186	381	12634144	4.56295	3.60388	0.59373	ENSG00000183049:intron	ENSG00000283699	12653137-12653197	-	19201
10	14470328	14470591	264	14470529	3.658	3.12558	0.30123	intergenic	ENSG00000151474	13643705-14462142	-	-8317
10	17029557	17029845	289	17029700	9.09099	5.60604	4.18865	ENSG00000107611:intron	ENSG00000107611	16823965-17129817	-	100116
10	17228471	17228705	235	17228569	6.90414	4.637	2.29778	ENSG00000229124:intron;ENSG00000026025:intron	ENSG00000026025	17228258-17237593	+	329
10	29659055	29659301	247	29659165	11.88032	6.15899	6.72383	ENSG00000197321:intron	ENSG00000225976	29697934-29698174	+	-38756
10	34994677	34994976	300	34994865	6.26578	4.40475	1.75945	intergenic	ENSG00000283000	34969908-34975578	+	24918
10	35023188	35023420	233	35023264	3.77589	3.20345	0.30123	ENSG00000108094:intron	ENSG00000283000	34969908-34975578	+	53395
10	38930143	38930360	218	38930210	5.89863	4.02031	1.48878	intergenic	ENSG00000233832	38783003-38783108	+	147248
10	38930143	38930360	218	38930210	5.89863	4.02031	1.48878	intergenic	ENSG00000233832	38783003-38783108	+	147248
10	38930143	38930360	218	38930210	5.89863	4.02031	1.48878	intergenic	ENSG00000233832	38783003-38783108	+	147248
10	38930143	38930360	218	38930210	5.89863	4.02031	1.48878	intergenic	ENSG00000233832	38783003-38783108	+	147248
10	42090671	42090880	210	42090774	13.67551	3.09397	8.37417	intergenic	ENSG00000229485	42149309-42149549	-	58774
10	42090671	42090880	210	42090774	13.67551	3.09397	8.37417	intergenic	ENSG00000229485	42149309-42149549	-	58774
10	42090671	42090880	210	42090774	13.67551	3.09397	8.37417	intergenic	ENSG00000229485	42149309-42149549	-	58774
10	42090671	42090880	210	42090774	13.67551	3.09397	8.37417	intergenic	ENSG00000229485	42149309-42149549	-	58774
10	42090671	42090880	210	42090774	13.67551	3.09397	8.37417	intergenic	ENSG00000229485	42149309-42149549	-	58774
10	44011059	44011340	282	44011198	10.09471	6.00647	5.09428	intergenic	ENSG00000233395	43939002-43944466	+	72197
10	44077712	44078058	347	44077920	3.77589	3.20345	0.30123	intergenic	ENSG00000233395	43939002-43944466	+	138882
10	44011059	44011340	282	44011198	10.09471	6.00647	5.09428	intergenic	ENSG00000233395	43939002-43944466	+	72197
10	44077712	44078058	347	44077920	3.77589	3.20345	0.30123	intergenic	ENSG00000233395	43939002-43944466	+	138882
10	45421232	45421470	239	45421335	5.75051	3.78743	1.36222	ENSG00000012779:intron	ENSG00000231964	45444569-45453121	-	31770
10	48963742	48964037	296	48963987	3.34993	2.92244	0.30123	ENSG00000128815:exon	ENSG00000226576	48984563-49018897	+	-20674
10	62187720	62187972	253	62187814	5.39424	4.00432	1.09512	intergenic	ENSG00000182010	62183034-62268707	-	80861
10	71206947	71207259	313	71207034	4.56295	3.60388	0.59373	intergenic	ENSG00000107731	71212569-71302864	+	-5466
10	72133574	72133849	276	72133703	6.5896	4.44231	2.02209	ENSG00000138303:intron	ENSG00000107742	72059034-72089032	-	-44679
10	73118067	73118349	283	73118208	11.96999	6.67886	6.80751	ENSG00000166321:intron	ENSG00000272599	73124572-73125532	-	7324
10	75106900	75107116	217	75106968	3.49438	2.91742	0.30123	ENSG00000079393:intron	ENSG00000079393	75094431-75109221	-	2213
10	80196277	80196511	235	80196415	11.01631	5.89057	5.93639	ENSG00000122359:intron	ENSG00000122359	80150888-80205572	-	9178
10	86272689	86272946	258	86272826	5.39424	4.00432	1.09512	ENSG00000182771:intron	ENSG00000199104	86264693-86264788	-	-8029
10	89701647	89701900	254	89701720	3.6499	2.91341	0.30123	ENSG00000138182:intron	ENSG00000138182	89701609-89774939	+	164
10	92101431	92101730	300	92101568	11.66864	5.82681	6.52722	ENSG00000107864:intron	ENSG00000214549	92118663-92119164	+	-17083
10	97024080	97024349	270	97024194	5.39424	4.00432	1.09512	ENSG00000187122:intron	ENSG00000234855	97102755-97103747	+	-78541
10	99298925	99299149	225	99299069	5.39424	4.00432	1.09512	intergenic	ENSG00000119946	99329098-99394330	+	-30061
10	102776074	102776320	247	102776212	5.07624	3.80169	0.93914	ENSG00000166272:intron	ENSG00000252994	102803928-102804031	+	-27731
10	103471406	103471665	260	103471532	9.91774	5.27833	4.94121	intergenic	ENSG00000183128	103472803-103479240	-	7705
10	113102486	113102729	244	113102556	6.26578	4.40475	1.75945	ENSG00000148737:intron	ENSG00000227560	112987475-112987865	+	115132
10	118154286	118154543	258	118154470	6.26578	4.40475	1.75945	ENSG00000177640:intron	ENSG00000238276	118241563-118267710	+	-87149
10	119705449	119705758	310	119705566	9.06429	5.18652	4.17525	intergenic	ENSG00000198825	119726041-119829278	+	-20438
10	124153109	124153378	270	124153245	5.65806	3.7345	1.30082	intergenic	ENSG00000182022	124007665-124093607	-	-59636
10	125763463	125763772	310	125763614	4.56295	3.60388	0.59373	ENSG00000107938:intron	ENSG00000236991	125725633-125752110	-	-11507
10	125917688	125917949	262	125917839	6.07691	3.57552	1.63695	ENSG00000203780:intron	ENSG00000203780	125896538-126009592	+	21280
10	127423105	127423351	247	127423209	4.56295	3.60388	0.59373	ENSG00000150760:intron	ENSG00000214285	127549368-127552639	+	-126140
11	1572205	1572478	274	1572330	7.01358	3.67516	2.39889	ENSG00000233930:Promoter;ENSG00000184545:Promoter	ENSG00000184545	1554043-1571920	-	-421

11	2772803	2773034	232	2772939	6.19969	4.04772	1.74352	ENSG00000053918:intron	ENSG00000236041	2771355-2771577	+	1563
11	3049862	3050148	287	3049979	9.63171	4.6211	4.69634	ENSG00000110619:intron;ENSG00000201616:Promoter	ENSG00000201616	3048358-3048521	-	-1483
11	3065883	3066100	218	3065945	4.21084	3.37603	0.54478	intergenic	ENSG00000110619	3000921-3057613	-	-8378
11	3930444	3930662	219	3930445	3.49438	2.91742	0.30123	ENSG00000167323:intron	ENSG00000206976	3922566-3922703	+	7986
11	4699740	4700035	296	4699868	14.11396	6.05711	8.77833	intergenic	ENSG00000167332	4680170-4697854	-	-2033
11	8757569	8757806	238	8757644	3.13189	2.77921	0.21867	ENSG00000166444:intron	ENSG00000255159	8768777-8810231	+	-11090
11	8795136	8795497	362	8795344	5.73591	4.07212	1.35894	ENSG00000166444:intron;ENSG00000255159:intron	ENSG00000255159	8768777-8810231	+	26539
11	9565937	9566268	332	9566029	4.91514	3.56573	0.82412	intergenic	ENSG00000166483	9573680-9593457	+	-7578
11	9593657	9593906	250	9593689	4.56295	3.60388	0.59373	intergenic	ENSG00000243964	9606141-9606609	+	-12360
11	9613157	9613445	289	9613263	10.60166	5.08797	5.56659	intergenic	ENSG00000243964	9606141-9606609	+	7159
11	9593657	9593906	250	9593689	4.56295	3.60388	0.59373	intergenic	ENSG00000243964	9606141-9606609	+	-12360
11	9613157	9613445	289	9613263	10.60166	5.08797	5.56659	intergenic	ENSG00000243964	9606141-9606609	+	7159
11	9940957	9941263	307	9941070	3.71102	3.1606	0.30123	ENSG00000133812:intron	ENSG00000254765	9958743-9959790	-	18680
11	10514622	10514897	276	10514795	3.658	3.12558	0.30123	ENSG00000110315:exon;ENSG00000110315:three_prime_UTR	ENSG00000255823	10507886-10509189	-	-5570
11	10657519	10657769	251	10657577	8.95304	4.76203	4.09227	ENSG00000072952:intron	ENSG00000072952	10573090-10693988	-	36344
11	10758898	10759255	358	10759166	4.56295	3.60388	0.59373	ENSG00000198730:intron	ENSG00000198730	10750986-10801625	+	8090
11	11154049	11154540	492	11154343	7.3143	4.5382	2.6406	ENSG00000254401:intron	ENSG00000254957	11135481-11137658	+	18813
11	14807301	14807753	453	14807598	5.39424	4.00432	1.09512	ENSG00000152270:intron	ENSG00000186104	14877439-14892252	-	84725
11	17391702	17391941	240	17391832	7.84906	4.50457	3.11977	intergenic	ENSG00000187486	17385858-17389331	-	-2490
11	17795697	17795944	248	17795718	4.2388	3.39408	0.56297	ENSG00000129159:intron;ENSG00000129158:intron	ENSG00000129159	17734773-17856804	+	61047
11	18577360	18577600	241	18577431	5.24168	3.90698	1.06394	ENSG00000151116:intron	ENSG00000151116	18529608-18588747	-	11267
11	19056029	19056348	320	19056097	3.49438	2.91742	0.30123	ENSG00000183695:exon	ENSG00000183695	19054454-19060681	-	4493
11	19442280	19442571	292	19442474	14.10904	6.25734	8.77684	ENSG00000166833:intron	ENSG00000200687	19401871-19401981	-	-40444
11	19660243	19660499	257	19660348	7.58982	3.93998	2.87738	ENSG00000166833:intron	ENSG00000270607	19710933-19712619	-	52248
11	19678826	19679054	229	19679051	3.02441	2.62206	0.15111	ENSG00000166833:intron	ENSG00000270607	19710933-19712619	-	33679
11	19888196	19888578	383	19888330	6.26578	4.40475	1.75945	ENSG00000166833:intron	ENSG00000254542	19978698-19981337	-	92950
11	19890739	19890948	210	19890850	4.97105	3.59979	0.86452	ENSG00000166833:intron	ENSG00000254542	19978698-19981337	-	90494
11	21071509	21071842	334	21071648	3.77589	3.20345	0.30123	ENSG00000165973:intron	ENSG00000201059	21000880-21000997	-	-70678
11	29513314	29513593	280	29513500	5.39424	4.00432	1.09512	ENSG00000254530:intron	ENSG00000255029	29519075-29552639	+	-5622
11	33596643	33596859	217	33596688	4.49014	3.43176	0.59373	ENSG00000110427:intron	ENSG00000255202	33665219-33696701	-	99950
11	34859977	34860199	223	34860070	4.35972	3.35026	0.59373	ENSG00000149089:intron	ENSG00000110435	34915828-35020591	+	-55740
11	34874980	34875264	285	34875233	4.56295	3.60388	0.59373	ENSG00000149089:three_prime_UTR;ENSG00000149089:exon	ENSG00000110435	34915828-35020591	+	-40706
11	35029907	35030271	365	35030090	8.134	4.66145	3.3598	intergenic	ENSG00000251862	34941836-34941920	+	88252
11	35040672	35040939	268	35040857	10.60417	4.76945	5.56702	intergenic	ENSG00000255521	35132654-35138032	-	97227
11	35144308	35144540	233	35144410	8.17781	4.86367	3.38531	ENSG00000026508:intron	ENSG00000026508	35138869-35232402	+	5554
11	36149719	36150039	321	36149876	11.1261	6.40691	6.0347	ENSG00000179241:intron	ENSG00000280321	36196247-36197901	+	-46368
11	37504102	37504440	339	37504305	9.85502	5.24413	4.88402	intergenic	ENSG00000251838	37702124-37702220	-	197949
11	41309311	41309536	226	41309429	9.59377	5.70253	4.65976	ENSG00000148948:intron	ENSG00000255132	41394594-41426504	+	-85171
11	41320362	41320565	204	41320439	5.6497	4.01836	1.29426	ENSG00000148948:intron	ENSG00000255132	41394594-41426504	+	-74131
11	44461854	44462199	346	44461881	3.38583	2.94608	0.30123	intergenic	ENSG00000255451	44468463-44470429	+	-6437
11	44498335	44498641	307	44498566	4.04884	3.27164	0.47041	intergenic	ENSG00000255451	44468463-44470429	+	30024
11	44461854	44462199	346	44461881	3.38583	2.94608	0.30123	intergenic	ENSG00000255451	44468463-44470429	+	-6437
11	44498335	44498641	307	44498566	4.04884	3.27164	0.47041	intergenic	ENSG00000255451	44468463-44470429	+	30024
11	44862364	44862605	242	44862440	6.32374	4.12047	1.8138	ENSG00000157570:intron	ENSG00000175274	44885902-44951306	-	88822

11	45188236	45188643	408	45188478	4.00982	3.24654	0.45978	ENSG00000019485:intron	ENSG00000254664	45215814-45235292	-	46853
11	46092717	46092956	240	46092915	4.1849	3.24157	0.52478	ENSG00000135365:intron	ENSG00000254653	46116577-46117318	+	-23741
11	55658090	55658310	221	55658170	5.26629	3.39198	1.07888	intergenic	ENSG00000181903	55662200-55666195	+	-4000
11	55722575	55722812	238	55722715	6.26578	4.40475	1.75945	ENSG00000186886:Promoter	ENSG00000186886	55723775-55729621	+	-1082
11	57216309	57216543	235	57216332	4.50398	3.56566	0.59373	intergenic	ENSG00000134817	57233576-57237314	-	20888
11	57685985	57686286	302	57686046	4.14216	3.33173	0.51073	ENSG00000156599:intron	ENSG00000156599	57667746-57701187	+	18389
11	57686566	57686783	218	57686737	3.77589	3.20345	0.30123	ENSG00000156599:intron	ENSG00000156599	57667746-57701187	+	18928
11	59431188	59431416	229	59431239	4.28127	3.42152	0.58762	intergenic	ENSG00000172324	59416968-59426412	-	-4889
11	60594303	60594575	273	60594423	9.09099	5.60604	4.18865	ENSG00000283601:intron	ENSG00000283601	60577855-60608418	+	16583
11	60732186	60732493	308	60732276	3.19543	2.82089	0.25814	ENSG00000214782:intron	ENSG00000214782	60729303-60744212	+	3036
11	60915538	60915812	275	60915675	9.79137	5.02659	4.83571	ENSG00000110108:intron;ENSG00000256944:Promoter	ENSG00000256944	60913165-60914052	-	-1622
11	62556527	62557020	494	62556768	9.79137	5.02659	4.83571	intergenic	ENSG00000124942	62433541-62556235	-	-538
11	63958462	63958736	275	63958539	3.02441	2.62206	0.15111	intergenic	ENSG00000207200	63970469-63970576	+	-11870
11	65382329	65382626	298	65382500	14.11417	6.47532	8.77833	ENSG00000162241:five_prime_UTR;ENSG00000162241:exon	ENSG00000162241	65375191-65383701	-	1224
11	65422107	65422355	249	65422216	5.86439	3.26245	1.4602	ENSG00000277599:Promoter;ENSG00000278144:Promoter;ENSG00000278050:Promoter;ENSG00000245532:Promoter	ENSG00000245532	65422773-65445540	+	-542
11	65588127	65588601	475	65588480	7.49188	4.02246	2.78905	ENSG00000173442:intron	ENSG00000173338	65592854-65595996	-	7632
11	69166550	69166891	342	69166735	18.41914	7.75626	12.84432	ENSG00000261070:intron	ENSG00000261070	69147227-69171564	-	4844
11	69216535	69216780	246	69216720	3.49438	2.91742	0.30123	intergenic	ENSG00000261070	69147227-69171564	-	-45093
11	69220766	69220998	233	69220866	6.90414	4.637	2.29778	intergenic	ENSG00000261070	69147227-69171564	-	-49317
11	69166550	69166891	342	69166735	18.41914	7.75626	12.84432	ENSG00000261070:intron	ENSG00000261070	69147227-69171564	-	4844
11	69216535	69216780	246	69216720	3.49438	2.91742	0.30123	intergenic	ENSG00000261070	69147227-69171564	-	-45093
11	69220766	69220998	233	69220866	6.90414	4.637	2.29778	intergenic	ENSG00000261070	69147227-69171564	-	-49317
11	69166550	69166891	342	69166735	18.41914	7.75626	12.84432	ENSG00000261070:intron	ENSG00000261070	69147227-69171564	-	4844
11	69216535	69216780	246	69216720	3.49438	2.91742	0.30123	intergenic	ENSG00000261070	69147227-69171564	-	-45093
11	69220766	69220998	233	69220866	6.90414	4.637	2.29778	intergenic	ENSG00000261070	69147227-69171564	-	-49317
11	69900496	69900740	245	69900687	3.77589	3.20345	0.30123	intergenic	ENSG00000260348	69909183-69910994	-	10376
11	71197532	71197815	284	71197673	7.17425	4.80518	2.52654	ENSG00000162105:intron	ENSG00000162105	70467855-71252577	-	54904
11	71204374	71204581	208	71204466	4.07388	3.28775	0.47041	ENSG00000162105:intron	ENSG00000162105	70467855-71252577	-	48100
11	72828113	72828393	281	72828241	7.02298	4.36872	2.40809	ENSG00000168010:intron	ENSG00000168010	72814307-72843674	+	13945
11	73779703	73779951	249	73779889	5.42584	3.06915	1.11564	intergenic	ENSG00000175581	73787315-73865133	+	-7488
11	74382116	74382434	319	74382224	4.23481	3.27254	0.56211	ENSG00000165434:intron	ENSG00000254631	74397548-74485742	+	-15273
11	74953693	74954013	321	74953855	5.77031	4.0936	1.37979	ENSG00000118363:intron	ENSG00000118363	74949246-74979031	+	4606
11	77738961	77739192	232	77738984	4.11506	3.19834	0.49781	ENSG00000255409:intron;ENSG00000048649:intron	ENSG00000255409	77738679-77739568	-	492
11	78860284	78860490	207	78860358	3.07013	2.73874	0.17545	ENSG00000149256:intron	ENSG00000254563	78749249-78756480	+	111137
11	79030815	79031203	389	79030967	5.66809	4.02982	1.31021	ENSG00000149256:intron	ENSG00000255345	79092847-79098003	+	-61838
11	80042576	80042795	220	80042696	4.00982	3.24654	0.45978	intergenic	ENSG00000254471	79987512-79989630	+	55173
11	80501606	80501858	253	80501783	6.47779	4.37345	1.94623	intergenic	ENSG00000200146	80527959-80528066	+	-26227
11	81107830	81108134	305	81107834	4.383	3.48731	0.59373	intergenic	ENSG00000285568	81015848-81040236	+	92133
11	82975370	82975613	244	82975575	4.37258	3.23911	0.59373	ENSG00000137502:three_prime_UTR;ENSG00000254698:intron;ENSG00000137502:exon	ENSG00000137509	82823501-82970584	-	-4907

11	82978505	82978770	266	82978594	6.38722	4.15784	1.8702	ENSG00000137502:three_prime_UTR;ENSG00000254698:intron;ENSG00000137502:exon	ENSG00000137509	82823501-82970584	-	-8053
11	82997252	82997522	271	82997418	9.87853	4.44888	4.90548	ENSG00000254698:intron;ENSG00000137502:intron	ENSG00000137509	82823501-82970584	-	-26802
11	82975370	82975613	244	82975575	4.37258	3.23911	0.59373	ENSG00000137502:three_prime_UTR;ENSG00000254698:intron;ENSG00000137502:exon	ENSG00000137509	82823501-82970584	-	-4907
11	82978505	82978770	266	82978594	6.38722	4.15784	1.8702	ENSG00000137502:three_prime_UTR;ENSG00000254698:intron;ENSG00000137502:exon	ENSG00000137509	82823501-82970584	-	-8053
11	82997252	82997522	271	82997418	9.87853	4.44888	4.90548	ENSG00000254698:intron;ENSG00000137502:intron	ENSG00000137509	82823501-82970584	-	-26802
11	83573837	83574150	314	83573969	3.77589	3.20345	0.30123	ENSG00000150672:intron	ENSG00000255311	83643601-83725390	+	-69608
11	83662907	83663123	217	83662925	3.29424	2.88581	0.30123	ENSG00000255311:intron;ENSG00000150672:intron	ENSG00000255311	83643601-83725390	+	19413
11	83791079	83791395	317	83791292	4.45692	3.17469	0.59373	ENSG00000254629:exon;ENSG00000150672:intron	ENSG00000254629	83789976-83791578	-	341
11	84289469	84289681	213	84289601	5.56876	3.96799	1.238	ENSG00000150672:intron	ENSG00000254713	84545130-84546846	-	257271
11	84471684	84472075	392	84471913	4.16942	3.34931	0.52478	ENSG00000150672:intron	ENSG00000254713	84545130-84546846	-	74967
11	84546237	84546494	258	84546451	3.02441	2.62206	0.15111	ENSG00000150672:intron;ENSG00000254713:intron	ENSG00000254713	84545130-84546846	-	481
11	84289469	84289681	213	84289601	5.56876	3.96799	1.238	ENSG00000150672:intron	ENSG00000254713	84545130-84546846	-	257271
11	84471684	84472075	392	84471913	4.16942	3.34931	0.52478	ENSG00000150672:intron	ENSG00000254713	84545130-84546846	-	74967
11	84546237	84546494	258	84546451	3.02441	2.62206	0.15111	ENSG00000150672:intron;ENSG00000254713:intron	ENSG00000254713	84545130-84546846	-	481
11	84946535	84946763	229	84946538	3.53832	2.94528	0.30123	ENSG00000255555:intron;ENSG00000150672:intron	ENSG00000255555	84936688-84955705	+	9960
11	85042434	85042669	236	85042553	4.84177	3.65305	0.77787	ENSG00000150672:intron	ENSG00000213305	85020784-85021655	-	-20896
11	86705036	86705282	247	86705129	4.07388	3.28775	0.47041	ENSG00000254731:intron	ENSG00000254731	86703098-86714092	+	2060
11	87173469	87173772	304	87173682	4.64049	3.52611	0.64891	ENSG00000166575:intron	ENSG00000279836	87121057-87121596	+	52563
11	88295817	88296027	211	88295882	4.00012	3.12743	0.45978	ENSG00000109861:intron	ENSG00000109861	88293591-88337787	-	41865
11	88297826	88298148	323	88298020	5.11088	3.68528	0.9653	ENSG00000109861:intron	ENSG00000109861	88293591-88337787	-	39800
11	88884526	88884820	295	88884677	4.56295	3.60388	0.59373	ENSG00000168959:intron	ENSG00000168959	88504575-89065945	-	181272
11	89057361	89057695	335	89057559	5.39424	4.00432	1.09512	ENSG00000168959:intron	ENSG00000168959	88504575-89065945	-	8417
11	89174823	89175085	263	89174899	4.56295	3.60388	0.59373	intergenic	ENSG00000077498	89177451-89295759	+	-2497
11	89555235	89555477	243	89555317	5.6497	4.01836	1.29426	ENSG00000279056:Promoter:ENSG00000255429:intron	ENSG00000279056	89556859-89557205	+	-1503
11	90326643	90327081	439	90326987	5.39424	4.00432	1.09512	ENSG00000261645:intron	ENSG00000214391	90282559-90284172	+	44302
11	91183598	91183813	216	91183736	3.73797	3.1784	0.30123	intergenic	ENSG00000280124	91184933-91185543	-	1838
11	92156610	92156835	226	92156774	3.29424	2.88581	0.30123	intergenic	ENSG00000242248	92161105-92161889	+	-4383
11	92195992	92196228	237	92196088	6.02088	4.25059	1.59152	intergenic	ENSG00000242248	92161105-92161889	+	35004
11	92214779	92215006	228	92214930	4.56295	3.60388	0.59373	intergenic	ENSG00000242248	92161105-92161889	+	53787
11	92643566	92643778	213	92643702	4.00982	3.24654	0.45978	ENSG00000165323:intron	ENSG00000255506	92748731-92766867	-	123195
11	92716055	92716316	262	92716178	5.07624	3.80169	0.93914	ENSG00000165323:intron	ENSG00000255506	92748731-92766867	-	50682
11	93620183	93620421	239	93620264	3.36259	2.93078	0.30123	intergenic	ENSG00000255515	93609759-93610333	-	-9968
11	93890825	93891054	230	93890841	3.81246	2.91013	0.32594	intergenic	ENSG00000214376	93818231-93850531	-	-40408
11	94674380	94674613	234	94674562	3.29424	2.88581	0.30123	ENSG00000255929:intron	ENSG00000166025	94706430-94876753	+	-31934

11	94850655	94851110	456	94850678	4.7547	3.59806	0.74279	ENSG00000166025:intron	ENSG00000213368	94913046-94913766	+	-62164
11	95147543	95147764	222	95147681	7.68737	4.41643	2.97084	intergenic	ENSG00000270578	95145436-95146004	+	2217
11	95295249	95295525	277	95295349	6.09321	4.13824	1.6498	intergenic	ENSG00000149212	95165512-95232541	-	-62845
11	96536113	96536334	222	96536205	4.06198	3.28009	0.47041	intergenic	ENSG00000256684	96508424-96514748	+	27799
11	97868307	97868698	392	97868612	3.77589	3.20345	0.30123	intergenic	ENSG00000254555	97908252-97908537	-	40035
11	98742460	98743161	702	98743000	4.56295	3.60388	0.59373	intergenic	ENSG00000254599	98676390-98683735	+	66420
11	99091646	99091861	216	99091703	3.81246	2.91013	0.32594	ENSG00000149972:intron	ENSG00000223269	99120175-99120490	+	-28422
11	99172760	99173168	409	99173114	3.03786	2.80302	0.15111	ENSG00000149972:intron	ENSG00000223269	99120175-99120490	+	52788
11	100910981	100911197	217	100911107	6.60855	4.454	2.0406	ENSG00000165895:intron	ENSG00000200047	100839629-100839946	+	71459
11	100936957	100937342	386	100936968	3.77589	3.20345	0.30123	ENSG00000165895:intron	ENSG00000200047	100839629-100839946	+	97520
11	101722872	101723112	241	101723034	3.93344	3.19748	0.41301	ENSG00000137672:intron	ENSG00000254534	101765934-101769961	-	46969
11	101775477	101775714	238	101775688	4.48857	3.55567	0.59373	ENSG00000137672:intron	ENSG00000254534	101765934-101769961	-	-5634
11	102643072	102643324	253	102643204	5.15789	3.85362	1.00126	intergenic	ENSG00000137674	102576834-102625332	-	-17865
11	102671567	102671800	234	102671715	3.89605	3.1735	0.38387	intergenic	ENSG00000281655	102681309-102683913	+	-9626
11	103566924	103567371	448	103567020	4.56295	3.60388	0.59373	intergenic	ENSG00000254824	103409579-103409657	-	-157490
11	103602596	103602817	222	103602801	3.77589	3.20345	0.30123	intergenic	ENSG00000254824	103409579-103409657	-	-193049
11	103848656	103848891	236	103848749	5.07624	3.80169	0.93914	ENSG00000264200:Promoter:ENSG00000254987:intron	ENSG00000264200	103849905-103849980	+	-1132
11	104552610	104552865	256	104552669	5.77031	4.0936	1.37979	ENSG00000256422:intron	ENSG00000256422	104445867-104609321	-	56584
11	104554394	104554663	270	104554621	4.07388	3.28775	0.47041	ENSG00000256422:intron	ENSG00000256422	104445867-104609321	-	54793
11	104552610	104552865	256	104552669	5.77031	4.0936	1.37979	ENSG00000256422:intron	ENSG00000256422	104445867-104609321	-	56584
11	104554394	104554663	270	104554621	4.07388	3.28775	0.47041	ENSG00000256422:intron	ENSG00000256422	104445867-104609321	-	54793
11	104705882	104706113	232	104705996	6.58155	3.96836	2.01637	intergenic	ENSG00000270449	104682382-104682581	-	-23416
11	107442924	107443164	241	107443062	3.76336	3.08858	0.30123	ENSG00000152404:intron	ENSG00000152404	107326344-107457844	-	14800
11	107505802	107506007	206	107505969	4.07388	3.28775	0.47041	ENSG00000137760:intron	ENSG00000152404	107326344-107457844	-	-48060
11	107634227	107634542	316	107634318	4.72575	3.5798	0.71873	ENSG00000110675:intron	ENSG00000254758	107642886-107643871	-	9487
11	108957539	108957766	228	108957628	6.4824	4.21404	1.94623	ENSG00000203334:Promoter	ENSG00000203334	108957717-108959797	+	-65
11	109461526	109461765	240	109461598	3.28305	2.87845	0.30123	ENSG00000255028:intron	ENSG00000254890	109486967-109487424	-	25779
11	110331342	110331705	364	110331505	4.64049	3.52611	0.64891	intergenic	ENSG00000254416	110355129-110406400	+	-23606
11	110367230	110367457	228	110367319	4.49014	3.43176	0.59373	ENSG00000254416:intron	ENSG00000254416	110355129-110406400	+	12214
11	110331342	110331705	364	110331505	4.64049	3.52611	0.64891	intergenic	ENSG00000254416	110355129-110406400	+	-23606
11	110367230	110367457	228	110367319	4.49014	3.43176	0.59373	ENSG00000254416:intron	ENSG00000254416	110355129-110406400	+	12214
11	112412565	112412805	241	112412779	3.9293	2.97803	0.41132	ENSG00000254968:intron	ENSG00000254968	112393117-112621729	+	19567
11	113230798	113231029	232	113230880	5.32771	3.96184	1.09512	ENSG00000149294:intron	ENSG00000227487	113269531-113273901	-	42988
11	113519752	113519967	216	113519893	6.74377	4.53757	2.15867	intergenic	ENSG00000149295	113409614-113475691	-	-44168
11	114080372	114080642	271	114080491	8.60945	4.92743	3.78062	ENSG00000109906:intron	ENSG00000109906	114059592-114250676	+	20914
11	114636904	114637185	282	114636987	5.68188	3.88989	1.31322	intergenic	ENSG00000137634	114570590-114595762	-	-41282
11	115222452	115222795	344	115222556	21.27201	9.59982	15.59819	ENSG00000182985:intron	ENSG00000252870	115128217-115128373	-	-94250
11	116002217	116002584	368	116002317	4.2388	3.39408	0.56297	intergenic	ENSG00000239600	115951104-115951491	-	-50909
11	116002217	116002584	368	116002317	4.2388	3.39408	0.56297	intergenic	ENSG00000239600	115951104-115951491	-	-50909
11	117254145	117254379	235	117254187	5.56616	3.82068	1.238	ENSG00000167257:intron	ENSG00000252992	117263798-117263952	+	-9536
11	118012256	118012498	243	118012404	7.37023	4.57095	2.68538	ENSG00000255274:intron	ENSG00000110324	117986347-118003037	+	26029
11	119376555	119376777	223	119376672	4.813	3.37843	0.77787	ENSG00000254740:intron;ENSG00000036672:intron	ENSG00000254740	119372705-119381613	+	3960
11	119494775	119495050	276	119494917	4.73338	3.4555	0.72598	ENSG00000245248:intron	ENSG00000255377	119444990-119445519	-	-49393
11	121036007	121036271	265	121036112	3.20396	2.64557	0.26167	ENSG00000285509:intron;ENSG00000154114:intron	ENSG00000285509	121024124-121113108	+	12014
11	121655969	121656196	228	121656189	3.28281	2.78383	0.30123	intergenic	ENSG00000246790	121447330-121453013	-	-203069
11	121754996	121755354	359	121755279	4.94982	3.72146	0.84841	intergenic	ENSG00000252556	122004354-122004451	+	-249179
11	122284860	122285211	352	122284931	4.23481	3.27254	0.56211	ENSG00000255248:intron	ENSG00000255219	122295189-122308019	+	-10154

11	122810463	122810713	251	122810492	3.02441	2.62206	0.15111	ENSG00000154127:exon;ENSG00000285909:intron;ENSG00000154127:three_prime_UTR	ENSG00000109943	122838499-122872639	+	-27911
11	123732337	123732554	218	123732443	5.53627	3.9478	1.2149	ENSG00000166261:intron	ENSG00000166261	123724176-123741675	-	9230
11	123904010	123904235	226	123904142	8.15538	5.03729	3.3763	ENSG00000181518:intron	ENSG00000181518	123902166-123909229	+	1956
11	124727351	124727570	220	124727471	5.31489	3.67153	1.09512	intergenic	ENSG00000154146	124739845-124747210	+	-12385
11	125036951	125037178	228	125037165	3.6499	2.91341	0.30123	ENSG00000149548:intron	ENSG00000134955	125063066-125090312	+	-26002
11	127252175	127252562	388	127252365	8.134	4.66145	3.3598	intergenic	ENSG00000273409	127271028-127337033	+	-18660
11	128241810	128242060	251	128241884	5.2271	3.75666	1.05667	intergenic	ENSG00000272575	128208748-128241441	+	33186
11	128342881	128343101	221	128342989	4.49014	3.43176	0.59373	intergenic	ENSG00000272575	128208748-128241441	+	134242
11	128241810	128242060	251	128241884	5.2271	3.75666	1.05667	intergenic	ENSG00000272575	128208748-128241441	+	33186
11	128342881	128343101	221	128342989	4.49014	3.43176	0.59373	intergenic	ENSG00000272575	128208748-128241441	+	134242
11	128388423	128388670	248	128388585	4.64049	3.52611	0.64891	intergenic	ENSG00000276176	128522389-128522449	+	-133843
11	128757294	128757556	263	128757368	7.09309	4.24847	2.46969	ENSG00000151702:intron	ENSG00000254703	128691671-128696023	-	-61401
11	129739591	129739869	279	129739710	4.19696	3.36708	0.53407	intergenic	ENSG00000255188	129788063-129788190	-	48460
11	130847539	130847808	270	130847693	4.07518	3.28858	0.47041	ENSG00000254842:intron	ENSG00000254842	130844190-130865561	-	17888
11	132357824	132358102	279	132357894	13.90053	6.58824	8.57994	intergenic	ENSG00000279090	132403359-132404060	-	46097
11	132510344	132510645	302	132510556	3.49438	2.91742	0.30123	ENSG00000183715:intron	ENSG00000283489	132525808-132525887	+	-15314
11	134157842	134158237	396	134157942	4.51692	3.44854	0.59373	ENSG00000151503:exon;ENSG00000151503:three_prime_UTR	ENSG00000254616	134173094-134173680	+	-15055
12	25450	25676	227	25563	5.39424	4.00432	1.09512	ENSG00000226210:intron	ENSG00000226210	14521-32015	-	6452
12	6142136	6142425	290	6142262	5.39424	4.00432	1.09512	intergenic	ENSG00000110799	5948873-6124770	-	-17510
12	7806477	7806706	230	7806626	4.56295	3.60388	0.59373	intergenic	ENSG00000199912	7800360-7800467	-	-6124
12	8066355	8066670	316	8066471	12.86462	6.48314	7.62811	ENSG00000171860:five_prime_UTR;ENSG00000171860:exon	ENSG00000171860	8058301-8066471	-	-41
12	8648662	8648903	242	8648820	5.6497	4.01836	1.29426	ENSG00000197614:intron	ENSG00000197614	8637345-8662888	-	14106
12	9237304	9237621	318	9237447	5.39424	4.00432	1.09512	ENSG00000256069:intron	ENSG00000283429	9239466-9239551	-	2089
12	9364401	9365061	661	9364510	5.6497	4.01836	1.29426	intergenic	ENSG00000260423	9367463-9397617	+	-2732
12	19498114	19498342	229	19498187	4.56295	3.60388	0.59373	ENSG00000139154:Promoter	ENSG00000240328	19508903-19509245	+	-10675
12	19894795	19895113	319	19894945	10.09471	6.00647	5.09428	ENSG00000255910:intron	ENSG00000256849	19941520-19943018	+	-46566
12	22118010	22118367	358	22118330	3.77589	3.20345	0.30123	ENSG00000111728:intron;ENSG00000283582:intron	ENSG00000274624	22104490-22105320	-	-12868
12	24172177	24172398	222	24172229	8.34229	4.44647	3.53121	ENSG00000134532:intron	ENSG00000216192	24212420-24212495	+	-40133
12	24950546	24950774	229	24950599	5.02824	3.7712	0.90545	ENSG00000255921:intron;ENSG00000060982:Promoter	ENSG00000060982	24810021-24949459	-	-1200
12	25333722	25334263	542	25334105	9.10792	5.4109	4.20447	intergenic	ENSG00000278743	25385669-25386241	-	52249
12	25811227	25811475	249	25811270	3.49438	2.91742	0.30123	ENSG00000256686:intron	ENSG00000256686	25782705-25814171	-	2820
12	26298955	26299323	369	26299157	13.05915	5.39155	7.81101	ENSG00000256234:intron;ENSG00000255750:intron;ENSG00000123096:intron	ENSG00000255858	26315714-26317813	+	-16575
12	27179835	27180186	352	27180020	13.8647	5.41602	8.5517	intergenic	ENSG00000256226	27105150-27161393	-	-18617
12	27530001	27530229	229	27530194	5.2587	3.91783	1.07467	ENSG00000110841:intron	ENSG00000110841	27523430-27695564	+	6684
12	28793725	28794096	372	28793953	5.6497	4.01836	1.29426	intergenic	ENSG00000256513	28821974-28825831	-	31921
12	28962184	28962496	313	28962402	4.56295	3.60388	0.59373	intergenic	ENSG00000278687	28978583-28978685	+	-16243
12	29496466	29496721	256	29496683	4.14216	3.33173	0.51073	ENSG00000187950:intron	ENSG00000187950	29412473-29497686	-	1093
12	29516425	29516684	260	29516532	5.6497	4.01836	1.29426	ENSG00000133687:intron	ENSG00000257456	29519730-29529974	+	-3176
12	29782253	29782481	229	29782301	5.74381	3.92704	1.36222	ENSG00000133687:intron	ENSG00000133687	29500839-29784759	-	2392
12	30138219	30138447	229	30138313	3.77589	3.20345	0.30123	intergenic	ENSG00000276431	30169880-30170091	-	31758

12	30760704	30760934	231	30760843	5.2079	3.88546	1.04111	ENSG00000246331:intron;ENSG00000235884:intron	ENSG00000235884	30755166-30802602	+	5652
12	31024458	31024870	413	31024720	3.50566	3.02505	0.30123	ENSG00000245614:intron	ENSG00000256984	31012502-31015806	+	12161
12	32229881	32230112	232	32229962	4.84177	3.65305	0.77787	ENSG00000258134:Promoter:ENSG00000151746:intron	ENSG00000258134	32227409-32228154	-	-1842
12	32734711	32734973	263	32734765	5.24168	3.90698	1.06394	ENSG00000139131:intron;ENSG00000087470:intron	ENSG00000275854	32736929-32737660	-	2818
12	32906797	32907055	259	32906868	10.40026	5.97008	5.38122	intergenic	ENSG00000057294	32790744-32896840	-	-10085
12	32906797	32907055	259	32906868	10.40026	5.97008	5.38122	intergenic	ENSG00000057294	32790744-32896840	-	-10085
12	34497332	34497544	213	34497387	4.56295	3.60388	0.59373	intergenic	ENSG00000256614	34249480-34249958	+	247957
12	37294539	37294759	221	37294647	3.77589	3.20345	0.30123	intergenic	ENSG00000258368	37575586-37576384	+	-280937
12	38543185	38543487	303	38543341	9.64493	5.73344	4.70826	intergenic	ENSG00000257645	38532894-38533050	-	-10285
12	39752731	39753006	276	39752885	8.7445	5.0039	3.90503	ENSG00000180116:intron	ENSG00000199571	39819749-39819883	-	67015
12	40722887	40723163	277	40723006	5.39424	4.00432	1.09512	ENSG00000018236:intron	ENSG00000257680	40728810-40729897	+	-5785
12	41769898	41770184	287	41770037	14.54384	6.70083	9.16949	intergenic	ENSG00000257784	41764188-41765581	+	5852
12	46219696	46219912	217	46219715	3.6499	2.91341	0.30123	ENSG00000111371:intron	ENSG00000274591	46239105-46239473	-	19669
12	46360924	46361176	253	46361115	4.69706	3.56173	0.69764	ENSG00000134294:exon	ENSG00000258096	46371462-46373778	+	-10412
12	46665012	46665258	247	46665165	6.26578	4.40475	1.75945	ENSG00000257261:intron	ENSG00000271642	46682070-46682713	-	17578
12	47812896	47813200	305	47813058	15.47791	5.45626	10.04498	ENSG00000061273:intron	ENSG00000274737	47817450-47817966	-	4918
12	50833992	50834215	224	50834157	6.49439	4.38367	1.95029	intergenic	ENSG00000243075	50841497-50841785	-	7682
12	51189323	51189585	263	51189439	6.82552	4.41829	2.2309	ENSG00000184271:exon;ENSG00000184271:three_prime UTR	ENSG00000278126	51201683-51202581	-	13127
12	51301988	51302346	359	51302247	3.23881	2.84938	0.2852	ENSG00000110934:intron	ENSG00000110934	51281037-51324668	-	22501
12	53518731	53519024	294	53518992	3.32816	2.90812	0.30123	ENSG00000170653:intron;ENSG00000267281:intron	ENSG00000257550	53513983-53517608	+	4894
12	56338672	56338905	234	56338831	7.17593	4.45747	2.52803	ENSG00000110944:intron	ENSG00000110944	56334173-56340410	+	4615
12	56443397	56443725	329	56443514	4.1849	3.24157	0.52478	ENSG00000111602:intron	ENSG00000111602	56416372-56449403	-	5842
12	56713512	56713728	217	56713607	5.66875	3.88202	1.31047	ENSG00000196531:exon	ENSG00000196531	56712427-56731628	-	18008
12	56818892	56819116	225	56819038	4.14216	3.33173	0.51073	intergenic	ENSG00000258679	56822984-56874268	+	-3980
12	57631869	57632075	207	57631960	4.69815	3.31233	0.69764	ENSG00000135454:exon	ENSG00000135454	57623409-57633355	-	1383
12	58425543	58425792	250	58425627	7.3115	4.37011	2.6406	intergenic	ENSG00000258133	58394595-58395138	+	31072
12	59787269	59787536	268	59787337	7.17425	4.80518	2.52654	intergenic	ENSG00000237176	59812101-59812576	+	-24699
12	63024766	63025350	585	63025212	4.56295	3.60388	0.59373	intergenic	ENSG00000257235	63004337-63006541	-	-18516
12	64088442	64088722	281	64088579	7.17425	4.80518	2.52654	ENSG00000196935:intron;ENSG00000255886:intron	ENSG00000255886	64038561-64097618	-	9036
12	65355593	65355805	213	65355738	5.6497	4.01836	1.29426	ENSG00000174099:intron	ENSG00000215208	65418730-65420027	-	64328
12	65440176	65440436	261	65440265	4.56295	3.60388	0.59373	ENSG00000174099:intron	ENSG00000215208	65418730-65420027	-	-20278
12	66453356	66453632	277	66453479	4.44284	3.52605	0.59373	intergenic	ENSG00000256285	66563523-66563694	+	-110029
12	66508095	66508311	217	66508284	3.77589	3.20345	0.30123	intergenic	ENSG00000256285	66563523-66563694	+	-55320
12	66697576	66697825	250	66697683	4.76929	3.60726	0.74587	ENSG00000155974:intron	ENSG00000256420	66563966-66569194	-	-128506
12	67449490	67449796	307	67449649	13.59613	6.89707	8.29827	ENSG00000203585:intron	ENSG00000203585	67443104-67590771	+	6538
12	67450265	67450477	213	67450321	4.11506	3.19834	0.49781	ENSG00000203585:intron	ENSG00000203585	67443104-67590771	+	7266
12	71174017	71174256	240	71174154	5.24168	3.90698	1.06394	ENSG00000127324:intron	ENSG00000258053	71047401-71118247	-	-55889
12	71195485	71195835	351	71195660	5.6497	4.01836	1.29426	ENSG00000127324:intron	ENSG00000258053	71047401-71118247	-	-77412
12	71174017	71174256	240	71174154	5.24168	3.90698	1.06394	ENSG00000127324:intron	ENSG00000258053	71047401-71118247	-	-55889
12	71195485	71195835	351	71195660	5.6497	4.01836	1.29426	ENSG00000127324:intron	ENSG00000258053	71047401-71118247	-	-77412
12	73498762	73498983	222	73498860	7.17425	4.80518	2.52654	intergenic	ENSG00000243164	73648665-73649039	+	-149793
12	75944244	75944865	622	75944385	6.58155	3.96836	2.01637	ENSG00000258077:intron	ENSG00000243420	75957832-75958130	-	13576
12	75977907	75978231	325	75978038	14.29237	6.34938	8.94032	ENSG00000258077:intron	ENSG00000258077	75563201-75984015	-	5946
12	76669380	76669820	441	76669438	4.56295	3.60388	0.59373	intergenic	ENSG00000243071	76660404-76660884	+	9195

12	76836952	76837272	321	76837139	4.56295	3.60388	0.59373	ENSG00000186908:intron	ENSG00000257910	76878192-76880352	+	-41080
12	77002305	77002608	304	77002574	6.26578	4.40475	1.75945	intergenic	ENSG00000257199	76984078-76984669	-	-17787
12	78515542	78515867	326	78515679	5.39424	4.00432	1.09512	ENSG00000257165:intron	ENSG00000257165	78448994-78540675	-	24971
12	79561490	79561853	364	79561785	5.25679	3.77493	1.07467	intergenic	ENSG00000257956	79558781-79559166	+	2890
12	81582907	81583203	297	81582994	6.26578	4.40475	1.75945	ENSG00000139220:intron	ENSG00000258375	81545325-81546450	-	-36604
12	82843830	82844055	226	82843908	5.56876	3.96799	1.238	ENSG00000179104:intron	ENSG00000179104	82686879-83134870	+	157063
12	84058180	84058396	217	84058201	3.77589	3.20345	0.30123	intergenic	ENSG00000285191	84147303-84154338	-	96050
12	85518681	85518949	269	85518869	4.53506	3.5858	0.59373	intergenic	ENSG00000257855	85567877-85568239	+	-49062
12	88960295	88960798	504	88960471	6.49439	4.38367	1.95029	intergenic	ENSG00000246363	89010680-89019679	-	59133
12	89469373	89469632	260	89469503	8.65039	5.33675	3.81818	ENSG00000139323:intron	ENSG00000226982	89500092-89502670	+	-30590
12	89661064	89661285	222	89661274	3.18471	2.81386	0.2494	ENSG00000070961:intron	ENSG00000271614	89708958-89712590	+	-47784
12	90930706	90931024	319	90930891	5.39424	4.00432	1.09512	ENSG00000196243:intron;ENSG00000197651:intron	ENSG00000196243	90918022-90948669	+	12842
12	91007868	91008108	241	91008064	4.84177	3.65305	0.77787	intergenic	ENSG00000083782	90963678-91005026	-	-2961
12	91023474	91023903	430	91023583	5.39424	4.00432	1.09512	intergenic	ENSG00000083782	90963678-91005026	-	-18662
12	91007868	91008108	241	91008064	4.84177	3.65305	0.77787	intergenic	ENSG00000083782	90963678-91005026	-	-2961
12	91023474	91023903	430	91023583	5.39424	4.00432	1.09512	intergenic	ENSG00000083782	90963678-91005026	-	-18662
12	91778937	91779277	341	91779158	12.43382	5.82026	7.22732	intergenic	ENSG00000258224	91871121-91871230	-	92123
12	92055993	92056284	292	92056131	7.47727	4.8125	2.77843	ENSG00000257242:intron	ENSG00000257242	91984975-92142914	-	86776
12	92539248	92539567	320	92539414	13.43669	6.56761	8.1476	intergenic	ENSG00000258262	92530459-92531324	+	8948
12	93759440	93759665	226	93759583	5.87556	4.15944	1.46959	ENSG00000169372:intron	ENSG00000258274	93707790-93737823	-	-21729
12	93948870	93949108	239	93949047	4.56295	3.60388	0.59373	intergenic	ENSG00000271382	93945040-93947813	+	3948
12	94561001	94561235	235	94561090	8.17399	4.36112	3.38452	ENSG00000266099:Promoter	ENSG00000266099	94561788-94561859	+	-670
12	94640569	94640865	297	94640739	11.04042	5.90426	5.95914	ENSG00000057704:intron	ENSG00000057704	94567123-94650562	-	9845
12	95700507	95700740	234	95700574	4.76422	3.12858	0.74587	ENSG00000074527:intron	ENSG00000212448	95617688-95617786	+	82935
12	96195626	96195897	272	96195741	15.35374	6.89446	9.92645	ENSG00000111145:intron	ENSG00000111145	96194381-96269835	+	1380
12	96502551	96502910	360	96502771	16.55362	7.28351	11.05902	ENSG00000188596:intron	ENSG00000188596	96489570-96875555	+	13160
12	98028953	98029226	274	98029071	7.17425	4.80518	2.52654	intergenic	ENSG00000263890	97995382-97995448	-	-33641
12	98147058	98147306	249	98147089	3.77589	3.20345	0.30123	ENSG00000258312:intron	ENSG00000258312	98113013-98292445	-	145263
12	98153620	98153939	320	98153711	3.77589	3.20345	0.30123	ENSG00000258312:intron	ENSG00000258312	98113013-98292445	-	138666
12	98249311	98249575	265	98249544	3.03786	2.80302	0.15111	ENSG00000258312:intron	ENSG00000258312	98113013-98292445	-	43002
12	98274900	98275139	240	98275012	5.70747	3.2883	1.33527	ENSG00000258312:intron	ENSG00000258312	98113013-98292445	-	17426
12	99624741	99624947	207	99624878	6.26578	4.40475	1.75945	ENSG00000185046:intron	ENSG00000180219	99647752-99650046	+	-22908
12	99628357	99628602	246	99628506	5.39424	4.00432	1.09512	ENSG00000185046:intron	ENSG00000180219	99647752-99650046	+	-19273
12	101201457	101201753	297	101201602	6.22868	4.22079	1.75945	ENSG00000256870:intron	ENSG00000207414	101199270-101199377	-	-2227
12	101309178	101309487	310	101309257	3.98411	3.23002	0.45207	ENSG00000120800:intron	ENSG00000120800	101280108-101386616	+	29224
12	102465376	102465640	265	102465417	5.39424	4.00432	1.09512	ENSG00000017427:intron	ENSG00000017427	102395873-102481744	-	16236
12	102474456	102475036	581	102474515	4.38813	3.49062	0.92326	ENSG00000017427:intron	ENSG00000017427	102395873-102481744	-	6998
12	103505362	103505643	282	103505485	14.14747	7.21453	8.80937	ENSG00000257994:exon	ENSG00000257994	103505410-103506174	-	672
12	105648762	105648980	219	105648905	5.68188	3.88989	1.31322	intergenic	ENSG00000257859	105704202-105744062	+	-55331
12	106557063	106557314	252	106557109	4.56295	3.60388	0.59373	ENSG00000257545:intron	ENSG00000111783	106582906-106762803	+	-25718
12	106561067	106561283	217	106561082	4.15576	3.3405	0.52015	ENSG00000257545:intron	ENSG00000111783	106582906-106762803	+	-21731
12	107558966	107559372	407	107559232	5.39424	4.00432	1.09512	ENSG00000151136:intron	ENSG00000257579	107610033-107617721	-	58552
12	107616712	107617040	329	107616953	4.50398	3.56566	0.59373	ENSG00000151136:intron;ENSG00000257579:intron	ENSG00000257579	107610033-107617721	-	845
12	108568909	108569157	249	108568984	6.58155	3.96836	2.01637	ENSG00000136003:exon;ENSG00000136003:three_prime UTR	ENSG00000136003	108562581-108569384	+	6451
12	108617422	108617639	218	108617546	6.58155	3.96836	2.01637	intergenic	ENSG00000257221	108628686-108641318	+	-11156
12	108838438	108838706	269	108838619	10.01593	5.14387	5.02923	ENSG00000207622:Promoter:ENSG00000084112:intron	ENSG00000207622	108836907-108837006	-	-1565

12	109040438	109040757	320	109040608	5.39424	4.00432	1.09512	ENSG00000135093:intron	ENSG00000256262	109052349-109053952	-	13355
12	113422160	113422400	241	113422255	9.09099	5.60604	4.18865	ENSG00000139410:exon;ENSG00000135094:intron;ENSG00000139410:five_prime_UTR	ENSG00000139410	113422236-113438276	+	43
12	124390893	124391122	230	124390914	3.49438	2.91742	0.30123	ENSG00000196498:intron	ENSG00000255965	124383380-124383510	+	7627
12	124453411	124453836	426	124453677	7.17425	4.80518	2.52654	ENSG00000196498:intron	ENSG00000214650	124513221-124514813	+	-59598
12	126772353	126772672	320	126772519	13.26553	7.20777	7.98923	ENSG00000256128:Promoter	ENSG00000256128	126729786-126772411	-	-101
13	27131181	27131504	324	27131326	13.26553	7.20777	7.98923	ENSG00000152484:intron	ENSG00000232162	27162854-27169135	+	-31512
13	41019242	41019491	250	41019347	4.56295	3.60388	0.59373	ENSG00000120690:Promoter	ENSG00000229473	40992778-40993331	-	-26035
13	47950065	47950501	437	47950413	4.56295	3.60388	0.59373	ENSG00000136143:intron	ENSG00000260388	47930152-47932622	-	-17660
13	50511072	50511360	289	50511222	5.39424	4.00432	1.09512	ENSG00000176124:intron	ENSG00000229323	50520932-50527449	-	16233
13	77000480	77000765	286	77000657	3.49438	2.91742	0.30123	ENSG00000283208:intron;ENSG00000005812:intron;ENSG00000102805:intron	ENSG00000283208	76992077-77129589	+	8545
13	79338708	79338917	210	79338861	3.77589	3.20345	0.30123	ENSG00000139746:intron	ENSG00000252496	79389533-79389631	-	50819
13	79385848	79386079	232	79385997	7.17425	4.80518	2.52654	ENSG00000139746:intron	ENSG00000252496	79389533-79389631	-	3668
13	80318178	80318420	243	80318379	4.84177	3.65305	0.77787	intergenic	ENSG00000136158	80335975-80340951	-	22652
13	82620080	82620345	266	82620310	4.56295	3.60388	0.59373	intergenic	ENSG00000237099	82823130-82824136	+	-202918
13	84199127	84199455	329	84199347	3.34993	2.92244	0.30123	intergenic	ENSG00000285742	84063105-84069408	+	136185
13	94171610	94171814	205	94171739	3.77589	3.20345	0.30123	ENSG00000183098:intron;ENSG00000236520:intron	ENSG00000236520	94154192-94187991	-	16279
13	94213554	94213860	307	94213607	4.50398	3.56566	0.59373	ENSG00000183098:intron	ENSG00000236520	94154192-94187991	-	-25715
13	95749664	95749887	224	95749761	3.02441	2.62206	0.15111	ENSG00000102580:intron	ENSG00000276809	95744725-95745765	+	5050
13	97318106	97318346	241	97318227	4.91514	3.56573	0.82412	ENSG00000139793:intron	ENSG00000202290	97362971-97363101	-	44875
13	99151607	99151910	304	99151743	7.86641	5.0511	3.13611	intergenic	ENSG00000230871	99173592-99174490	+	-21834
13	99375109	99375392	284	99375283	4.76929	3.60726	0.74587	ENSG00000134882:intron	ENSG00000228808	99372172-99372686	+	3078
13	109156759	109157009	251	109156987	4.07388	3.28775	0.47041	ENSG00000041515:intron	ENSG00000236242	109163901-109201483	-	44599
13	109162257	109162494	238	109162452	5.74381	3.92704	1.36222	ENSG00000041515:intron	ENSG00000236242	109163901-109201483	-	39108
13	112376158	112376374	217	112376290	5.68492	4.04031	1.31535	ENSG00000153498:Promoter	ENSG00000153498	112376318-112434689	+	-52
13	112562420	112562659	240	112562453	3.73797	3.1784	0.30123	ENSG00000126216:intron	ENSG00000126216	112485004-112588205	-	25666
13	113647873	113648092	220	113647984	3.76336	3.08858	0.30123	intergenic	ENSG00000186009	113648803-113658186	-	10204
14	22464394	22464670	277	22464519	11.1261	6.40691	6.0347	ENSG00000211829:intron;ENSG00000251002:intron	ENSG00000211829	22462931-22465787	+	1600
14	57060574	57060817	244	57060703	8.17554	4.86236	3.38452	ENSG00000248550:intron	ENSG00000259136	57044645-57044960	-	-15735
14	68210806	68211045	240	68210902	7.17425	4.80518	2.52654	ENSG00000182185:intron	ENSG00000243546	68236242-68236539	-	25614
14	95265535	95265786	252	95265647	12.86462	6.48314	7.62811	ENSG00000165959:intron	ENSG00000165959	95181939-95319906	-	54246
14	105582051	105582303	253	105582176	8.99866	4.4694	4.12107	intergenic	ENSG00000211890	105583730-105588395	-	6218
15	20353342	20353666	325	20353513	17.93691	5.26834	12.38107	ENSG00000258654:intron	ENSG00000258654	20344735-20359166	+	8768
15	20421765	20422045	281	20421897	3.58279	2.53746	0.30123	ENSG00000180229:intron	ENSG00000258654	20344735-20359166	+	77169
15	20353342	20353666	325	20353513	17.93691	5.26834	12.38107	ENSG00000258654:intron	ENSG00000258654	20344735-20359166	+	8768
15	20421765	20422045	281	20421897	3.58279	2.53746	0.30123	ENSG00000180229:intron	ENSG00000258654	20344735-20359166	+	77169
15	23423172	23423565	394	23423383	12.79845	6.68291	7.56866	intergenic	ENSG00000261418	23430838-23435212	+	-7470
15	24594695	24594948	254	24594876	4.56295	3.60388	0.59373	ENSG00000259905:intron	ENSG00000185823	24675867-24683393	+	-81046
15	25071226	25071569	344	25071485	4.56295	3.60388	0.59373	ENSG00000206727:Promoter	ENSG00000207093	25070431-25070526	+	966
15	27518565	27518895	331	27518622	5.39424	4.00432	1.09512	ENSG00000259168:intron;ENSG00000182256:intron	ENSG00000259168	27483034-27541991	-	23261
15	27554111	27554326	216	27554135	4.56295	3.60388	0.59373	intergenic	ENSG00000259168	27483034-27541991	-	-12227
15	28219380	28219603	224	28219474	4.56295	3.60388	0.59373	ENSG00000128731:intron	ENSG00000256338	28315650-28316059	+	-96159
15	29709818	29710052	235	29709945	5.73591	4.07212	1.35894	intergenic	ENSG00000259644	29728185-29729531	-	19596

15	29821404	29821668	265	29821535	12.43382	5.82026	7.22732	ENSG00000259523:Promoter:ENSG00000104067:intron	ENSG00000259523	29822630-29824081	+	-1094
15	32839510	32839740	231	32839709	4.58495	3.49121	0.61075	ENSG00000248905:intron	ENSG00000259392	32836982-32837326	-	-2298
15	32872569	32872773	205	32872624	4.91514	3.56573	0.82412	ENSG00000248905:intron	ENSG00000259392	32836982-32837326	-	-35344
15	34254888	34255110	223	34254994	4.31475	3.20475	0.59373	ENSG00000259468:Promoter:ENSG00000140199:intron	ENSG00000259468	34255285-34257832	+	-286
15	37912985	37913300	316	37913256	3.76522	3.1964	0.30123	intergenic	ENSG00000166069	37921938-37967724	+	-8796
15	38069091	38069372	282	38069186	11.10484	6.16387	6.01942	intergenic	ENSG00000236914	38069480-38072959	-	3728
15	38084476	38084861	386	38084622	5.73591	4.07212	1.35894	intergenic	ENSG00000236914	38069480-38072959	-	-11709
15	38086064	38086272	209	38086111	3.6499	2.91341	0.30123	intergenic	ENSG00000236914	38069480-38072959	-	-13208
15	38069091	38069372	282	38069186	11.10484	6.16387	6.01942	intergenic	ENSG00000236914	38069480-38072959	-	3728
15	38084476	38084861	386	38084622	5.73591	4.07212	1.35894	intergenic	ENSG00000236914	38069480-38072959	-	-11709
15	38086064	38086272	209	38086111	3.6499	2.91341	0.30123	intergenic	ENSG00000236914	38069480-38072959	-	-13208
15	40098772	40099030	259	40098869	10.76909	4.43321	5.71076	ENSG00000104081:intron	ENSG00000104081	40087889-40108892	-	9991
15	43440754	43441096	343	43440955	9.06429	5.18652	4.17525	ENSG00000067369:intron	ENSG00000243871	43391383-43391727	-	-49197
15	43649253	43649495	243	43649414	5.68188	3.88989	1.31322	ENSG00000166762:intron	ENSG00000224677	43649122-43649280	+	251
15	43948685	43949201	517	43948805	5.75051	3.78743	1.36222	ENSG00000171877:intron	ENSG00000185607	43989060-43990184	-	41241
15	44308436	44308659	224	44308566	5.26629	3.39198	1.07888	ENSG00000166734:intron	ENSG00000166734	44288728-44415758	+	19819
15	44726719	44726951	233	44726809	11.66864	5.82681	6.52722	intergenic	ENSG00000185880	44728987-44767829	+	-2152
15	45061380	45061620	241	45061578	3.49438	2.91742	0.30123	ENSG00000140263:intron	ENSG00000259418	45041715-45058707	-	-2792
15	47841294	47841524	231	47841369	4.76929	3.60726	0.74587	ENSG00000259572:intron	ENSG00000259572	47803383-47846236	-	4827
15	49133974	49134221	248	49134178	3.77589	3.20345	0.30123	ENSG00000166200:intron	ENSG00000200120	49137761-49137863	-	3766
15	51058661	51058876	216	51058734	4.29558	3.43077	0.59373	ENSG00000259240:intron;ENSG00000183578:intron	ENSG00000259204	51064608-51069586	-	10818
15	51243862	51244107	246	51243954	8.23437	4.24593	3.43385	ENSG00000259240:intron;ENSG00000137869:intron	ENSG00000266593	51242189-51242264	+	1795
15	52006397	52006793	397	52006674	4.08844	3.29712	0.48089	ENSG00000069956:intron	ENSG00000274528	52017166-52018032	-	11437
15	52021186	52021418	233	52021314	4.45692	3.17469	0.59373	ENSG00000069956:intron	ENSG00000259438	52010998-52019095	-	-2206
15	53504304	53504588	285	53504462	5.39424	4.00432	1.09512	intergenic	ENSG00000252066	53651976-53652123	+	-147530
15	55279739	55280027	289	55279881	9.72009	5.17084	4.77897	ENSG00000069974:intron	ENSG00000276533	55288848-55289346	-	9463
15	55279739	55280027	289	55279881	9.72009	5.17084	4.77897	ENSG00000069974:intron	ENSG00000276533	55288848-55289346	-	9463
15	56371821	56372059	239	56371938	8.17554	4.86236	3.38452	ENSG00000151575:intron	ENSG00000259941	56394320-56396785	-	24845
15	56393640	56393853	214	56393700	3.77589	3.20345	0.30123	intergenic	ENSG00000259941	56394320-56396785	-	3039
15	58332205	58332445	241	58332341	8.28323	5.11428	3.47693	ENSG00000128918:intron	ENSG00000166035	58410568-58569843	+	-78243
15	58673775	58674142	368	58673813	6.26578	4.40475	1.75945	ENSG00000137845:intron	ENSG00000270986	58672295-58672933	+	1663
15	59052285	59052512	228	59052454	6.78327	4.56203	2.19117	intergenic	ENSG00000241640	59049398-59050385	-	-2013
15	60255505	60255733	229	60255654	6.51641	4.07877	1.96373	intergenic	ENSG00000240163	60390370-60390682	+	-134751
15	60660540	60660830	291	60660669	5.27581	3.92874	1.08581	ENSG00000069667:intron	ENSG00000277413	60677108-60677272	-	16587
15	63430497	63430762	266	63430627	5.53627	3.9478	1.2149	ENSG00000259459:intron	ENSG00000259268	63456293-63469351	-	38722
15	66415534	66415810	277	66415648	3.53038	3.04135	0.30123	ENSG00000169032:intron;ENSG00000261102:Promoter	ENSG00000261102	66414932-66415198	-	-473
15	67077275	67077485	211	67077399	4.10177	3.30571	0.49129	ENSG00000166949:intron	ENSG00000259347	66984107-67065268	-	-12111
15	67091269	67091529	261	67091427	8.99866	4.4694	4.12107	ENSG00000166949:intron	ENSG00000259347	66984107-67065268	-	-26130
15	69241305	69241577	273	69241348	4.07388	3.28775	0.47041	ENSG00000138604:intron	ENSG00000259504	69278674-69295322	+	-37233
15	69714255	69714526	272	69714381	9.6365	5.31664	4.70027	intergenic	ENSG00000259496	69803315-69804046	-	89656
15	69727205	69727503	299	69727235	3.81246	2.91013	0.32594	intergenic	ENSG00000259496	69803315-69804046	-	76692
15	69755638	69755919	282	69755790	9.27287	5.50951	4.35502	intergenic	ENSG00000259496	69803315-69804046	-	48268
15	69761036	69761269	234	69761113	8.9436	5.11735	4.08953	intergenic	ENSG00000259496	69803315-69804046	-	42894
15	69714255	69714526	272	69714381	9.6365	5.31664	4.70027	intergenic	ENSG00000259496	69803315-69804046	-	89656
15	69727205	69727503	299	69727235	3.81246	2.91013	0.32594	intergenic	ENSG00000259496	69803315-69804046	-	76692
15	69755638	69755919	282	69755790	9.27287	5.50951	4.35502	intergenic	ENSG00000259496	69803315-69804046	-	48268

15	69761036	69761269	234	69761113	8.9436	5.11735	4.08953	intergenic	ENSG00000259496	69803315-69804046	-	42894
15	69714255	69714526	272	69714381	9.6365	5.31664	4.70027	intergenic	ENSG00000259496	69803315-69804046	-	89656
15	69727205	69727503	299	69727235	3.81246	2.91013	0.32594	intergenic	ENSG00000259496	69803315-69804046	-	76692
15	69755638	69755919	282	69755790	9.27287	5.50951	4.35502	intergenic	ENSG00000259496	69803315-69804046	-	48268
15	69761036	69761269	234	69761113	8.9436	5.11735	4.08953	intergenic	ENSG00000259496	69803315-69804046	-	42894
15	69714255	69714526	272	69714381	9.6365	5.31664	4.70027	intergenic	ENSG00000259496	69803315-69804046	-	89656
15	69727205	69727503	299	69727235	3.81246	2.91013	0.32594	intergenic	ENSG00000259496	69803315-69804046	-	76692
15	69755638	69755919	282	69755790	9.27287	5.50951	4.35502	intergenic	ENSG00000259496	69803315-69804046	-	48268
15	69761036	69761269	234	69761113	8.9436	5.11735	4.08953	intergenic	ENSG00000259496	69803315-69804046	-	42894
15	69979904	69980142	239	69980047	7.3143	4.5382	2.6406	intergenic	ENSG00000207965	70079371-70079468	-	99445
15	70602185	70602420	236	70602191	3.20396	2.64557	0.26167	ENSG00000259227:exon	ENSG00000259227	70602123-70602516	+	179
15	70669183	70669435	253	70669283	6.4824	4.21404	1.94623	ENSG00000137831:exon	ENSG00000280515	70635248-70637314	+	34060
15	71643380	71643678	299	71643548	3.49438	2.91742	0.30123	ENSG00000187720:intron	ENSG00000278408	71547279-71549832	-	-93696
15	71984906	71985233	328	71985065	3.77589	3.20345	0.30123	ENSG00000261632:intron;ENSG00000066933:intron	ENSG00000261632	71972205-72040265	+	12864
15	71985490	71985731	242	71985698	3.6499	2.91341	0.30123	ENSG00000261632:intron;ENSG00000066933:intron	ENSG00000261632	71972205-72040265	+	13405
15	72692597	72692846	250	72692701	4.56295	3.60388	0.59373	ENSG00000140463:intron	ENSG00000140463	72686178-72738476	+	6543
15	74021984	74022288	305	74022064	5.68188	3.88989	1.31322	ENSG00000140464:intron	ENSG00000140464	73994672-74047812	+	27463
15	75043112	75043364	253	75043205	8.62542	3.92169	3.79549	intergenic	ENSG00000138621	75023554-75117462	+	19683
15	76408790	76409023	234	76408930	4.56295	3.60388	0.59373	ENSG00000140386:intron	ENSG00000261232	76472098-76472365	+	-63192
15	81301844	81302117	274	81301990	10.64217	4.64231	5.60051	ENSG00000172349:intron	ENSG00000271725	81303214-81309391	-	7411
15	81496473	81496722	250	81496680	3.53038	3.04135	0.30123	ENSG00000259543:intron;ENSG00000259343:intron	ENSG00000259594	81554002-81696780	+	-57405
15	81509376	81509614	239	81509436	4.56295	3.60388	0.59373	ENSG00000259543:intron;ENSG00000259343:intron	ENSG00000259594	81554002-81696780	+	-44507
15	81514851	81515092	242	81514987	5.8934	4.17062	1.48561	ENSG00000259543:intron;ENSG00000259343:intron	ENSG00000259594	81554002-81696780	+	-39031
15	81496473	81496722	250	81496680	3.53038	3.04135	0.30123	ENSG00000259543:intron;ENSG00000259343:intron	ENSG00000259594	81554002-81696780	+	-57405
15	81509376	81509614	239	81509436	4.56295	3.60388	0.59373	ENSG00000259543:intron;ENSG00000259343:intron	ENSG00000259594	81554002-81696780	+	-44507
15	81514851	81515092	242	81514987	5.8934	4.17062	1.48561	ENSG00000259543:intron;ENSG00000259343:intron	ENSG00000259594	81554002-81696780	+	-39031
15	81807738	81807971	234	81807919	5.39424	4.00432	1.09512	ENSG00000259692:intron	ENSG00000259692	81660481-81871125	-	63271
15	81880216	81880528	313	81880386	5.39424	4.00432	1.09512	intergenic	ENSG00000259692	81660481-81871125	-	-9246
15	85330921	85331282	362	85331139	5.26629	3.39198	1.07888	ENSG00000218052:Promoter	ENSG00000218052	85255368-85330334	-	-767
15	85413517	85413763	247	85413645	10.65162	5.29115	5.60305	ENSG00000170776:intron;ENSG00000259630:Promoter	ENSG00000259630	85415227-85415633	+	-1587
15	85978463	85978707	245	85978540	5.19114	3.87478	1.02732	intergenic	ENSG00000259608	85958716-85958971	-	-19613
15	88638818	88639085	268	88638989	9.95162	4.34993	4.97164	ENSG00000172183:intron	ENSG00000172183	88636152-88656483	+	2799
15	88764922	88765141	220	88764999	4.14216	3.33173	0.51073	intergenic	ENSG00000259676	88797412-88798734	-	33703
15	89303271	89303477	207	89303364	4.42776	3.51628	0.59373	ENSG00000140525:intron	ENSG00000275101	89326738-89326810	-	23436
15	90075515	90075747	233	90075615	5.6982	3.75745	1.3285	ENSG00000259291:intron;ENSG00000140548:intron;ENSG00000276376:Promoter	ENSG00000276376	90076423-90076486	+	-792
15	92156388	92156623	236	92156476	5.29302	3.93971	1.09512	ENSG00000260661:intron;ENSG00000176463:intron	ENSG00000258761	92162797-92172436	-	15931
15	96211518	96211800	283	96211657	3.77589	3.20345	0.30123	ENSG00000247809:intron;ENSG00000275443:intron	ENSG00000278146	96230896-96231145	+	-19237

15	96399326	96399702	377	96399431	4.56295	3.60388	0.59373	ENSG00000259542:intron;ENSG00000259275:intron	ENSG00000259542	96393145-96405189	-	5675
15	101193134	101193378	245	101193162	4.22478	3.38503	0.55558	ENSG00000131873:intron	ENSG00000259182	101168529-101170821	+	24726
15	101199785	101200118	334	101199935	13.91069	5.59892	8.58776	ENSG00000131873:intron	ENSG00000259182	101168529-101170821	+	31422
15	101193134	101193378	245	101193162	4.22478	3.38503	0.55558	ENSG00000131873:intron	ENSG00000259182	101168529-101170821	+	24726
15	101199785	101200118	334	101199935	13.91069	5.59892	8.58776	ENSG00000131873:intron	ENSG00000259182	101168529-101170821	+	31422
15	101233511	101233736	226	101233648	4.88847	3.6826	0.81688	ENSG00000131873:intron	ENSG00000131873	101175722-101251932	-	18309
16	72621	72831	211	72783	8.23437	4.24593	3.43385	ENSG00000007384:Promoter;ENSG00000278995:Promoter	ENSG00000278995	74682-74961	+	-1956
16	1722277	1722558	282	1722415	6.45945	4.36218	1.93323	ENSG00000138834:intron	ENSG00000261399	1713526-1714208	-	-8209
16	8124376	8124660	285	8124569	3.49438	2.91742	0.30123	intergenic	ENSG00000260003	8276262-8295700	-	171182
16	8145462	8145716	255	8145537	3.03786	2.80302	0.15111	intergenic	ENSG00000260003	8276262-8295700	-	150111
16	9970262	9970509	248	9970424	4.84177	3.65305	0.77787	ENSG00000183454:intron	ENSG00000261810	10033683-10037297	+	-63298
16	9986478	9986724	247	9986666	3.77589	3.20345	0.30123	ENSG00000183454:intron	ENSG00000261810	10033683-10037297	+	-47082
16	10738858	10739222	365	10739012	11.81017	6.34701	6.66017	intergenic	ENSG00000103274	10743785-10769351	+	-4745
16	10871195	10871441	247	10871315	5.62443	3.85549	1.28474	ENSG00000262151:intron	ENSG00000179583	10866221-10943021	+	5096
16	11328591	11328813	223	11328653	8.85591	4.88357	4.00947	ENSG00000175643:intron	ENSG00000199668	11315066-11315178	+	13635
16	11682660	11682956	297	11682749	9.43096	5.20133	4.50508	ENSG00000153066:intron	ENSG00000184602	11668413-11679159	+	14394
16	12027130	12027482	353	12027279	10.65162	5.29115	5.60305	ENSG00000048471:intron	ENSG00000260488	11976850-11977850	-	-49455
16	13218936	13219202	267	13219082	4.56295	3.60388	0.59373	ENSG00000237515:intron	ENSG00000262116	13197605-13204907	-	-14161
16	13864996	13865247	252	13865047	4.383	3.48731	0.59373	intergenic	ENSG00000175595	13920156-13952345	+	-55035
16	15674488	15674722	235	15674616	6.22868	4.22079	1.75945	ENSG00000072864:intron	ENSG00000262380	15683289-15684570	+	-8684
16	16102512	16102735	224	16102617	4.58409	3.36557	0.61075	ENSG00000103222:intron	ENSG00000241067	16094191-16095209	-	-7414
16	17029914	17030172	259	17030116	5.32771	3.96184	1.09512	intergenic	ENSG00000279490	17082294-17085049	-	55006
16	17259403	17259666	264	17259561	16.55362	7.28351	11.05902	ENSG00000103489:intron	ENSG00000279620	17249673-17251395	-	-8139
16	19384708	19384925	218	19384820	6.36907	4.30669	1.85528	ENSG00000259925:intron	ENSG00000280265	19392212-19393468	-	8652
16	23335767	23336239	473	23336147	8.9436	5.11735	4.08953	ENSG00000168447:intron	ENSG00000168447	23278230-23381299	+	57772
16	24231755	24232038	284	24231946	4.00012	3.12743	0.45978	intergenic	ENSG00000260264	24236103-24252863	+	-4207
16	25048800	25049071	272	25048837	3.81246	2.91013	0.32594	ENSG00000262587:intron	ENSG00000262587	25031743-25058109	+	17192
16	25607917	25608209	293	25608101	7.73286	4.96898	3.01041	intergenic	ENSG00000182601	25692025-26137688	+	-83962
16	25656142	25656372	231	25656325	3.63193	3.10837	0.30123	intergenic	ENSG00000182601	25692025-26137688	+	-35768
16	25607917	25608209	293	25608101	7.73286	4.96898	3.01041	intergenic	ENSG00000182601	25692025-26137688	+	-83962
16	25656142	25656372	231	25656325	3.63193	3.10837	0.30123	intergenic	ENSG00000182601	25692025-26137688	+	-35768
16	27355572	27355808	237	27355746	3.6499	2.91341	0.30123	ENSG00000077238:intron	ENSG00000274092	27313386-27314101	-	-41588
16	27537654	27537872	219	27537772	4.84177	3.65305	0.77787	ENSG00000077235:intron	ENSG00000077235	27459554-27549913	-	12150
16	31265420	31265641	222	31265506	5.24168	3.90698	1.06394	ENSG00000169896:intron	ENSG00000169896	31259966-31332892	+	5564
16	34572644	34572851	208	34572736	6.13491	1.76265	1.68471	intergenic	ENSG00000284209	34353615-34371659	-	-201088
16	34576409	34576618	210	34576499	25.81676	2.33136	19.98989	intergenic	ENSG00000284209	34353615-34371659	-	-204854
16	34582973	34583566	594	34583058	62.27531	1.92033	55.53629	intergenic	ENSG00000284209	34353615-34371659	-	-211610
16	34588038	34588281	244	34588175	84.55322	2.54151	77.40413	intergenic	ENSG00000284209	34353615-34371659	-	-216500
16	34572644	34572851	208	34572736	6.13491	1.76265	1.68471	intergenic	ENSG00000284209	34353615-34371659	-	-201088
16	34576409	34576618	210	34576499	25.81676	2.33136	19.98989	intergenic	ENSG00000284209	34353615-34371659	-	-204854
16	34582973	34583566	594	34583058	62.27531	1.92033	55.53629	intergenic	ENSG00000284209	34353615-34371659	-	-211610
16	34588038	34588281	244	34588175	84.55322	2.54151	77.40413	intergenic	ENSG00000284209	34353615-34371659	-	-216500
16	34572644	34572851	208	34572736	6.13491	1.76265	1.68471	intergenic	ENSG00000284209	34353615-34371659	-	-201088
16	34576409	34576618	210	34576499	25.81676	2.33136	19.98989	intergenic	ENSG00000284209	34353615-34371659	-	-204854
16	34582973	34583566	594	34583058	62.27531	1.92033	55.53629	intergenic	ENSG00000284209	34353615-34371659	-	-211610
16	34588038	34588281	244	34588175	84.55322	2.54151	77.40413	intergenic	ENSG00000284209	34353615-34371659	-	-216500
16	46380779	46381005	227	46380860	31.76341	2.63725	25.78696	intergenic	ENSG00000199448	46556088-46556191	-	175299
16	46387841	46388059	219	46387935	79.96134	1.86118	72.84288	intergenic	ENSG00000199448	46556088-46556191	-	168241

16	46388651	46389373	723	46388779	50.07467	2.05922	43.68239	intergenic	ENSG00000199448	46556088-46556191	-	167179
16	46389758	46390096	339	46390009	34.68384	1.57642	28.62212	intergenic	ENSG00000199448	46556088-46556191	-	166264
16	46390510	46390884	375	46390656	39.38259	1.41251	33.20861	intergenic	ENSG00000199448	46556088-46556191	-	165494
16	46394689	46394997	309	46394873	57.95827	1.57452	51.35972	intergenic	ENSG00000199448	46556088-46556191	-	161348
16	46399391	46399627	237	46399496	88.18564	1.90458	80.99057	intergenic	ENSG00000199448	46556088-46556191	-	156682
16	46400469	46401069	601	46400552	17.03701	1.56443	11.52195	intergenic	ENSG00000199448	46556088-46556191	-	155422
16	46380779	46381005	227	46380860	31.76341	2.63725	25.78696	intergenic	ENSG00000199448	46556088-46556191	-	175299
16	46387841	46388059	219	46387935	79.96134	1.86118	72.84288	intergenic	ENSG00000199448	46556088-46556191	-	168241
16	46388651	46389373	723	46388779	50.07467	2.05922	43.68239	intergenic	ENSG00000199448	46556088-46556191	-	167179
16	46389758	46390096	339	46390009	34.68384	1.57642	28.62212	intergenic	ENSG00000199448	46556088-46556191	-	166264
16	46390510	46390884	375	46390656	39.38259	1.41251	33.20861	intergenic	ENSG00000199448	46556088-46556191	-	165494
16	46394689	46394997	309	46394873	57.95827	1.57452	51.35972	intergenic	ENSG00000199448	46556088-46556191	-	161348
16	46399391	46399627	237	46399496	88.18564	1.90458	80.99057	intergenic	ENSG00000199448	46556088-46556191	-	156682
16	46400469	46401069	601	46400552	17.03701	1.56443	11.52195	intergenic	ENSG00000199448	46556088-46556191	-	155422
16	46380779	46381005	227	46380860	31.76341	2.63725	25.78696	intergenic	ENSG00000199448	46556088-46556191	-	175299
16	46387841	46388059	219	46387935	79.96134	1.86118	72.84288	intergenic	ENSG00000199448	46556088-46556191	-	168241
16	46388651	46389373	723	46388779	50.07467	2.05922	43.68239	intergenic	ENSG00000199448	46556088-46556191	-	167179
16	46389758	46390096	339	46390009	34.68384	1.57642	28.62212	intergenic	ENSG00000199448	46556088-46556191	-	166264
16	46390510	46390884	375	46390656	39.38259	1.41251	33.20861	intergenic	ENSG00000199448	46556088-46556191	-	165494
16	46394689	46394997	309	46394873	57.95827	1.57452	51.35972	intergenic	ENSG00000199448	46556088-46556191	-	161348
16	46399391	46399627	237	46399496	88.18564	1.90458	80.99057	intergenic	ENSG00000199448	46556088-46556191	-	156682
16	46400469	46401069	601	46400552	17.03701	1.56443	11.52195	intergenic	ENSG00000199448	46556088-46556191	-	155422
16	46380779	46381005	227	46380860	31.76341	2.63725	25.78696	intergenic	ENSG00000199448	46556088-46556191	-	175299
16	46387841	46388059	219	46387935	79.96134	1.86118	72.84288	intergenic	ENSG00000199448	46556088-46556191	-	168241
16	46388651	46389373	723	46388779	50.07467	2.05922	43.68239	intergenic	ENSG00000199448	46556088-46556191	-	167179
16	46389758	46390096	339	46390009	34.68384	1.57642	28.62212	intergenic	ENSG00000199448	46556088-46556191	-	166264
16	46390510	46390884	375	46390656	39.38259	1.41251	33.20861	intergenic	ENSG00000199448	46556088-46556191	-	165494
16	46394689	46394997	309	46394873	57.95827	1.57452	51.35972	intergenic	ENSG00000199448	46556088-46556191	-	161348
16	46399391	46399627	237	46399496	88.18564	1.90458	80.99057	intergenic	ENSG00000199448	46556088-46556191	-	156682
16	46400469	46401069	601	46400552	17.03701	1.56443	11.52195	intergenic	ENSG00000199448	46556088-46556191	-	155422
16	46380779	46381005	227	46380860	31.76341	2.63725	25.78696	intergenic	ENSG00000199448	46556088-46556191	-	175299
16	46387841	46388059	219	46387935	79.96134	1.86118	72.84288	intergenic	ENSG00000199448	46556088-46556191	-	168241
16	46388651	46389373	723	46388779	50.07467	2.05922	43.68239	intergenic	ENSG00000199448	46556088-46556191	-	167179
16	46389758	46390096	339	46390009	34.68384	1.57642	28.62212	intergenic	ENSG00000199448	46556088-46556191	-	166264
16	46390510	46390884	375	46390656	39.38259	1.41251	33.20861	intergenic	ENSG00000199448	46556088-46556191	-	165494
16	46394689	46394997	309	46394873	57.95827	1.57452	51.35972	intergenic	ENSG00000199448	46556088-46556191	-	161348
16	46399391	46399627	237	46399496	88.18564	1.90458	80.99057	intergenic	ENSG00000199448	46556088-46556191	-	156682
16	46400469	46401069	601	46400552	17.03701	1.56443	11.52195	intergenic	ENSG00000199448	46556088-46556191	-	155422
16	46380779	46381005	227	46380860	31.76341	2.63725	25.78696	intergenic	ENSG00000199448	46556088-46556191	-	175299
16	46387841	46388059	219	46387935	79.96134	1.86118	72.84288	intergenic	ENSG00000199448	46556088-46556191	-	168241
16	46388651	46389373	723	46388779	50.07467	2.05922	43.68239	intergenic	ENSG00000199448	46556088-46556191	-	167179
16	46389758	46390096	339	46390009	34.68384	1.57642	28.62212	intergenic	ENSG00000199448	46556088-46556191	-	166264
16	46390510	46390884	375	46390656	39.38259	1.41251	33.20861	intergenic	ENSG00000199448	46556088-46556191	-	165494
16	46394689	46394997	309	46394873	57.95827	1.57452	51.35972	intergenic	ENSG00000199448	46556088-46556191	-	161348
16	46399391	46399627	237	46399496	88.18564	1.90458	80.99057	intergenic	ENSG00000199448	46556088-46556191	-	156682
16	46400469	46401069	601	46400552	17.03701	1.56443	11.52195	intergenic	ENSG00000199448	46556088-46556191	-	155422
16	46380779	46381005	227	46380860	31.76341	2.63725	25.78696	intergenic	ENSG00000199448	46556088-46556191	-	175299
16	46387841	46388059	219	46387935	79.96134	1.86118	72.84288	intergenic	ENSG00000199448	46556088-46556191	-	168241
16	46388651	46389373	723	46388779	50.07467	2.05922	43.68239	intergenic	ENSG00000199448	46556088-46556191	-	167179
16	46389758	46390096	339	46390009	34.68384	1.57642	28.62212	intergenic	ENSG00000199448	46556088-46556191	-	166264
16	46390510	46390884	375	46390656	39.38259	1.41251	33.20861	intergenic	ENSG00000199448	46556088-46556191	-	165494

16	46394689	46394997	309	46394873	57.95827	1.57452	51.35972	intergenic	ENSG00000199448	46556088-46556191	-	161348
16	46399391	46399627	237	46399496	88.18564	1.90458	80.99057	intergenic	ENSG00000199448	46556088-46556191	-	156682
16	46400469	46401069	601	46400552	17.03701	1.56443	11.52195	intergenic	ENSG00000199448	46556088-46556191	-	155422
16	50121230	50121455	226	50121345	10.60738	5.45811	5.56869	ENSG00000260381:intron	ENSG00000260381	50100338-50121943	-	601
16	50318508	50318728	221	50318614	4.458	3.53587	0.59373	ENSG00000166164:exon;ENSG00000166164:three_prime UTR	ENSG00000274969	50292615-50292675	+	26002
16	53060317	53060624	308	53060473	4.1849	3.24157	0.52478	ENSG00000177200:intron	ENSG00000177200	53055032-53329150	+	5438
16	54501301	54501571	271	54501453	3.6499	2.91341	0.30123	intergenic	ENSG00000283304	54542807-54559100	-	57664
16	56608308	56608617	310	56608457	9.78373	4.69287	4.83465	ENSG00000125148:five_prime_UTR;ENSG00000125148:exon	ENSG00000125148	56608198-56609497	+	264
16	65193719	65193971	253	65193819	4.84177	3.65305	0.77787	ENSG00000260834:intron	ENSG00000259847	65141755-65176713	-	-17131
16	67042843	67043084	242	67042996	3.36259	2.93078	0.30123	ENSG00000067955:intron	ENSG00000067955	67029115-67101058	+	13848
16	67211650	67211895	246	67211766	5.59518	3.83801	1.25935	ENSG00000125122:intron	ENSG00000280163	67205174-67206849	-	-4923
16	71240249	71240467	219	71240353	5.29302	3.93971	1.09512	intergenic	ENSG00000157423	70807377-71230722	-	-9635
16	71255960	71256185	226	71256135	4.07388	3.28775	0.47041	intergenic	ENSG00000157423	70807377-71230722	-	-25350
16	78742505	78742766	262	78742662	15.49818	8.00863	10.06014	ENSG00000186153:intron	ENSG00000243101	78825280-78826006	+	-82645
16	79628520	79628769	250	79628630	7.68921	4.9422	2.9717	intergenic	ENSG00000275040	79619468-79620110	+	9176
17	1080897	1081169	273	1080969	4.60755	3.1497	0.62762	ENSG00000159842:intron	ENSG00000278794	1101006-1101273	-	20240
17	1089417	1089693	277	1089568	4.53041	3.45699	0.59373	ENSG00000159842:intron	ENSG00000278794	1101006-1101273	-	11718
17	1115126	1115718	593	1115287	11.51693	6.17716	6.39216	ENSG00000159842:intron	ENSG00000278794	1101006-1101273	-	-14148
17	1080897	1081169	273	1080969	4.60755	3.1497	0.62762	ENSG00000159842:intron	ENSG00000278794	1101006-1101273	-	20240
17	1089417	1089693	277	1089568	4.53041	3.45699	0.59373	ENSG00000159842:intron	ENSG00000278794	1101006-1101273	-	11718
17	1115126	1115718	593	1115287	11.51693	6.17716	6.39216	ENSG00000159842:intron	ENSG00000278794	1101006-1101273	-	-14148
17	3423298	3423518	221	3423492	4.35356	3.46825	0.59373	ENSG00000159961:exon;ENSG00000159961:three_prime UTR	ENSG00000127780	3432869-3433841	-	10433
17	3558741	3559103	363	3558886	4.86323	3.17994	0.79494	ENSG00000167723:Promoter	ENSG00000167723	3510501-3557995	-	-926
17	5798045	5798309	265	5798245	3.03786	2.80302	0.15111	ENSG00000284837:intron;ENSG00000285471:intron	ENSG00000285471	5772925-6080792	+	25251
17	5825126	5825359	234	5825222	7.41563	4.77491	2.72	ENSG00000284837:intron;ENSG00000285471:intron	ENSG00000285471	5772925-6080792	+	52317
17	6223334	6223570	237	6223453	13.68988	7.2038	8.38804	intergenic	ENSG00000206618	6207162-6207269	+	16289
17	6311688	6311921	234	6311708	4.29558	3.43077	0.59373	intergenic	ENSG00000262609	6322412-6323228	-	11424
17	6346497	6346702	206	6346648	3.03786	2.80302	0.15111	intergenic	ENSG00000262609	6322412-6323228	-	-23371
17	7842696	7842951	256	7842763	4.37258	3.23911	0.59373	ENSG00000132510:intron	ENSG00000132510	7839903-7854796	+	2920
17	8196126	8196394	269	8196277	10.54093	5.83399	5.51529	intergenic	ENSG00000271002	8199122-8199437	-	3177
17	8325236	8325468	233	8325309	5.26135	3.63995	1.07719	intergenic	ENSG00000212206	8329582-8329719	+	-4230
17	8359884	8360137	254	8360087	5.73591	4.07212	1.35894	ENSG00000184619:intron	ENSG00000265749	8365562-8381328	+	-5552
17	8422230	8422474	245	8422344	4.1849	3.24157	0.52478	intergenic	ENSG00000166579	8413130-8490411	+	9221
17	10390423	10390646	224	10390516	5.39424	4.00432	1.09512	ENSG00000272975:intron;ENSG00000133020:exon;ENSG00000272736:intron	ENSG00000272975	10383143-10623886	+	7391
17	10590074	10590467	394	10590420	3.77589	3.20345	0.30123	ENSG00000272975:intron;ENSG00000214970:intron	ENSG00000214970	10579039-10623036	+	11231
17	12177586	12177863	278	12177722	7.17425	4.80518	2.52654	intergenic	ENSG00000265400	12201599-12215267	+	-23875
17	12324325	12324543	219	12324385	3.77589	3.20345	0.30123	intergenic	ENSG00000265400	12201599-12215267	+	122834
17	14321199	14321444	246	14321269	3.77589	3.20345	0.30123	ENSG00000125430:intron	ENSG00000266378	14327334-14329474	+	-6013
17	15959173	15959518	346	15959336	16.55686	6.22102	11.06094	ENSG00000170425:intron	ENSG00000170425	15944916-15975746	+	14429
17	21287966	21288260	295	21288083	15.56859	5.80288	10.12304	ENSG00000034152:intron	ENSG00000034152	21284671-21315240	+	3441

17	22977519	22977740	222	22977631	4.56295	3.60388	0.59373	intergenic	ENSG00000264431	22693324-22694831	-	-282798
17	23016272	23016555	284	23016412	28.66482	3.08636	22.76824	intergenic	ENSG00000264431	22693324-22694831	-	-321582
17	22977519	22977740	222	22977631	4.56295	3.60388	0.59373	intergenic	ENSG00000264431	22693324-22694831	-	-282798
17	23016272	23016555	284	23016412	28.66482	3.08636	22.76824	intergenic	ENSG00000264431	22693324-22694831	-	-321582
17	25242843	25243058	216	25242957	6.4824	4.21404	1.94623	intergenic	ENSG00000263433	26981693-26982090	+	-1738743
17	26603714	26603972	259	26603844	74.61496	2.2178	67.61958	intergenic	ENSG00000263433	26981693-26982090	+	-377850
17	26619462	26619792	331	26619735	11.48992	1.58197	6.36995	intergenic	ENSG00000263433	26981693-26982090	+	-362066
17	26964455	26964673	219	26964540	3.64836	3.01523	0.30123	intergenic	ENSG00000263433	26981693-26982090	+	-17129
17	25242843	25243058	216	25242957	6.4824	4.21404	1.94623	intergenic	ENSG00000263433	26981693-26982090	+	-1738743
17	26603714	26603972	259	26603844	74.61496	2.2178	67.61958	intergenic	ENSG00000263433	26981693-26982090	+	-377850
17	26619462	26619792	331	26619735	11.48992	1.58197	6.36995	intergenic	ENSG00000263433	26981693-26982090	+	-362066
17	26964455	26964673	219	26964540	3.64836	3.01523	0.30123	intergenic	ENSG00000263433	26981693-26982090	+	-17129
17	25242843	25243058	216	25242957	6.4824	4.21404	1.94623	intergenic	ENSG00000263433	26981693-26982090	+	-1738743
17	26603714	26603972	259	26603844	74.61496	2.2178	67.61958	intergenic	ENSG00000263433	26981693-26982090	+	-377850
17	26619462	26619792	331	26619735	11.48992	1.58197	6.36995	intergenic	ENSG00000263433	26981693-26982090	+	-362066
17	26964455	26964673	219	26964540	3.64836	3.01523	0.30123	intergenic	ENSG00000263433	26981693-26982090	+	-17129
17	27332789	27333038	250	27332931	9.48025	4.55013	4.55226	ENSG00000266313:Promoter	ENSG00000266313	27333255-27348491	+	-342
17	27500082	27500431	350	27500275	4.9037	3.69224	0.82412	ENSG00000141068:intron	ENSG00000263368	27526385-27527223	-	26967
17	27532383	27532654	272	27532497	5.39424	4.00432	1.09512	ENSG00000141068:intron	ENSG00000263368	27526385-27527223	-	-5295
17	27500082	27500431	350	27500275	4.9037	3.69224	0.82412	ENSG00000141068:intron	ENSG00000263368	27526385-27527223	-	26967
17	27532383	27532654	272	27532497	5.39424	4.00432	1.09512	ENSG00000141068:intron	ENSG00000263368	27526385-27527223	-	-5295
17	28306105	28306338	234	28306264	3.42114	2.96933	0.30123	ENSG00000265480:intron	ENSG00000265480	28275985-28317783	-	11562
17	28328649	28328985	337	28328774	4.11517	3.31434	0.49781	ENSG00000109083:intron	ENSG00000109083	28328324-28335489	-	6672
17	29192388	29192640	253	29192591	4.66864	3.54383	0.67357	intergenic	ENSG00000264050	29197070-29197785	-	5271
17	32367947	32368281	335	32368033	4.458	3.53587	0.59373	ENSG00000010244:intron	ENSG00000010244	32350116-32381886	+	17997
17	32745924	32746308	385	32746076	7.17425	4.80518	2.52654	ENSG00000176658:intron	ENSG00000201178	32830218-32830329	-	84213
17	32815613	32815889	277	32815740	5.0924	3.81196	0.95207	ENSG00000176658:intron	ENSG00000201178	32830218-32830329	-	14578
17	32745924	32746308	385	32746076	7.17425	4.80518	2.52654	ENSG00000176658:intron	ENSG00000201178	32830218-32830329	-	84213
17	32815613	32815889	277	32815740	5.0924	3.81196	0.95207	ENSG00000176658:intron	ENSG00000201178	32830218-32830329	-	14578
17	34371319	34371562	244	34371387	5.46637	3.76126	1.15159	intergenic	ENSG00000108702	34360327-34363231	-	-8209
17	34387164	34387408	245	34387256	7.42804	4.43549	2.73175	intergenic	ENSG00000108702	34360327-34363231	-	-24054
17	34415372	34415579	208	34415537	5.08244	3.66786	0.94504	intergenic	ENSG00000108702	34360327-34363231	-	-52244
17	35063535	35063790	256	35063704	7.56656	4.19944	2.85958	ENSG00000092871:intron;ENSG00000267618:intron	ENSG00000267457	35073830-35074374	-	10712
17	35922056	35922574	519	35922118	4.56295	3.60388	0.59373	ENSG00000278023:intron	ENSG00000271013	35912634-35918010	-	-4304
17	39082994	39083263	270	39083136	4.11506	3.19834	0.49781	ENSG00000161381:intron;ENSG00000263818:intron	ENSG00000263818	39057018-39113190	+	26110
17	39770051	39770291	241	39770177	9.91774	5.27833	4.94121	ENSG00000161405:intron	ENSG00000141738	39737926-39747291	+	32244
17	40314625	40314917	293	40314719	8.9617	4.6034	4.09625	ENSG00000131759:intron	ENSG00000131759	40309191-40357643	+	5579
17	40587094	40587430	337	40587411	3.02441	2.62206	0.15111	intergenic	ENSG00000279775	40597112-40598585	-	11323
17	40621279	40621556	278	40621475	5.42487	3.87873	1.11519	intergenic	ENSG00000279775	40597112-40598585	-	-22832
17	41453141	41453365	225	41453211	5.0124	3.76115	0.89533	ENSG00000236473:intron	ENSG00000236473	41448182-41454055	-	802
17	43167462	43167701	240	43167536	4.62651	3.51732	0.63659	ENSG00000267002:exon	ENSG00000267595	43168169-43168249	-	668
17	43484608	43484847	240	43484722	6.06008	4.11811	1.6243	ENSG00000067596:intron	ENSG00000067596	43483864-43610338	+	863
17	43497498	43497712	215	43497574	5.96569	4.21594	1.54281	ENSG00000067596:intron	ENSG00000067596	43483864-43610338	+	13740
17	44807641	44808139	499	44808095	4.31475	3.20475	0.59373	ENSG00000182963:intron	ENSG00000267405	44794746-44797783	-	-10106
17	46764486	46764763	278	46764600	3.33182	2.81469	0.30123	intergenic	ENSG00000213326	46721581-46722167	-	-42457
17	48459086	48459307	222	48459218	4.71137	3.57074	0.70668	ENSG00000264451:Promoter	ENSG00000264451	48460369-48466040	+	-1173
17	48505839	48506099	261	48505992	10.78028	5.75701	5.71968	intergenic	ENSG00000120094	48528525-48531001	-	25032
17	48511142	48511552	411	48511272	5.95396	3.63426	1.53177	intergenic	ENSG00000120094	48528525-48531001	-	19654
17	48505839	48506099	261	48505992	10.78028	5.75701	5.71968	intergenic	ENSG00000120094	48528525-48531001	-	25032

17	48511142	48511552	411	48511272	5.95396	3.63426	1.53177	intergenic	ENSG00000120094	48528525-48531001	-	19654
17	49342300	49342518	219	49342453	6.51471	4.39616	1.96373	ENSG00000198740:intron	ENSG00000248714	49361164-49369998	+	-18755
17	51338975	51339358	384	51339186	3.49438	2.91742	0.30123	ENSG00000267452:intron	ENSG00000267452	51336714-51445802	+	2452
17	55705956	55706226	271	55706059	5.11088	3.68528	0.9653	intergenic	ENSG00000166292	55719626-55732121	-	26030
17	58232004	58232226	223	58232141	7.47727	4.8125	2.77843	ENSG00000167419:intron	ENSG00000011143	58205436-58219605	-	-12509
17	59786484	59786849	366	59786676	45.87442	16.35818	39.56588	ENSG00000062716:intron	ENSG00000267637	59784812-59785035	+	1854
17	60545781	60546040	260	60545806	4.16942	3.34931	0.52478	ENSG00000259349:intron	ENSG00000267678	60562009-60562161	-	16251
17	61440131	61440357	227	61440313	5.10865	3.82229	0.9653	intergenic	ENSG00000121075	61452403-61485110	+	-12159
17	64071162	64071415	254	64071262	5.93037	4.03949	1.51498	ENSG00000178607:intron	ENSG00000108622	64002593-64020634	-	-50654
17	64103837	64104079	243	64103985	10.3171	5.11983	5.30248	ENSG00000178607:intron	ENSG00000178607	64039141-64130819	-	26861
17	64793009	64793321	313	64793041	4.56295	3.60388	0.59373	ENSG00000214176:intron	ENSG00000215769	64749662-64781707	-	-11457
17	67199452	67199694	243	67199506	4.36824	3.47775	0.59373	ENSG00000198265:intron	ENSG00000264421	67224302-67225541	+	-24729
17	68402162	68402475	314	68402234	3.29424	2.88581	0.30123	ENSG00000141337:intron	ENSG00000267009	68413622-68524949	+	-11304
17	68774441	68775092	652	68774918	7.1573	4.79461	2.52654	intergenic	ENSG00000267250	68793548-68797822	-	23056
17	71927705	71928192	488	71928030	5.39424	4.00432	1.09512	intergenic	ENSG00000283517	71829869-71871750	-	-56198
17	72863606	72863848	243	72863834	4.1849	3.24157	0.52478	ENSG00000133195:intron	ENSG00000264196	72839038-72839718	+	24688
17	74702213	74702469	257	74702317	4.1849	3.24157	0.52478	ENSG00000186074:intron;ENSG00000172794:intron	ENSG00000186074	74694310-74712978	-	10637
17	76480958	76481334	377	76481211	5.79352	3.81213	1.40054	ENSG00000129667:intron	ENSG00000129667	76470890-76501790	-	20644
17	76812088	76812349	262	76812276	4.07388	3.28775	0.47041	intergenic	ENSG00000267065	76799043-76807102	+	13175
17	77107591	77107860	270	77107674	5.01585	3.49609	0.89533	ENSG00000129657:intron	ENSG00000284250	77089416-77089497	+	18309
17	77325005	77325316	312	77325079	7.36237	4.39861	2.67971	ENSG00000184640:intron	ENSG00000263718	77257736-77281897	-	-43263
17	77600752	77600982	231	77600790	4.31475	3.20475	0.59373	ENSG00000285535:intron	ENSG00000285535	77590531-77680687	+	10335
17	77834447	77834746	300	77834633	8.67675	4.78611	3.84073	intergenic	ENSG00000204283	77879026-77884087	-	49491
17	77843612	77843868	257	77843717	6.41549	4.02177	1.89285	intergenic	ENSG00000204283	77879026-77884087	-	40347
17	77834447	77834746	300	77834633	8.67675	4.78611	3.84073	intergenic	ENSG00000204283	77879026-77884087	-	49491
17	77843612	77843868	257	77843717	6.41549	4.02177	1.89285	intergenic	ENSG00000204283	77879026-77884087	-	40347
17	82708248	82708501	254	82708364	9.43096	5.20133	4.50508	intergenic	ENSG00000263063	82713907-82716255	-	7881
18	1981572	1982085	514	1981886	6.26578	4.40475	1.75945	intergenic	ENSG00000264189	1927726-1929094	-	-52734
18	3586808	3587122	315	3586973	11.26164	6.03046	6.15985	ENSG00000170579:intron	ENSG00000177337	3593731-3598352	+	-6766
18	5884403	5884766	364	5884583	10.09471	6.00647	5.09428	ENSG00000206432:exon;ENSG00000206432:three_prime UTR	ENSG00000264449	5887486-5909122	+	-2902
18	6004798	6005002	205	6004931	7.47727	4.8125	2.77843	ENSG00000154655:intron	ENSG00000200637	6017722-6017839	-	12939
18	6014553	6014768	216	6014633	3.77589	3.20345	0.30123	ENSG00000154655:intron	ENSG00000200637	6017722-6017839	-	3179
18	6219706	6219923	218	6219833	6.26578	4.40475	1.75945	ENSG00000154655:intron	ENSG00000264707	6256746-6260934	+	-36932
18	6235306	6235556	251	6235350	3.53832	2.94528	0.30123	ENSG00000154655:intron	ENSG00000264707	6256746-6260934	+	-21315
18	6291130	6291720	591	6291222	3.76522	3.1964	0.30123	ENSG00000154655:intron	ENSG00000264707	6256746-6260934	+	34678
18	7601233	7601482	250	7601304	5.82254	4.12626	1.42733	ENSG00000173482:intron	ENSG00000173482	7566781-8406861	+	34576
18	8457061	8457321	261	8457237	3.97135	3.22182	0.44441	intergenic	ENSG00000242985	8471830-8472128	+	-14639
18	9066421	9066650	230	9066580	6.87511	4.28345	2.27621	intergenic	ENSG00000178127	9102629-9134345	+	-36094
18	9082824	9083183	360	9082937	6.49439	4.38367	1.95029	intergenic	ENSG00000178127	9102629-9134345	+	-19626
18	9066421	9066650	230	9066580	6.87511	4.28345	2.27621	intergenic	ENSG00000178127	9102629-9134345	+	-36094
18	9082824	9083183	360	9082937	6.49439	4.38367	1.95029	intergenic	ENSG00000178127	9102629-9134345	+	-19626
18	9260571	9260808	238	9260640	4.71137	3.57074	0.70668	ENSG00000101745:intron	ENSG00000273284	9259387-9260390	+	1302
18	9427444	9427741	298	9427581	3.94601	3.20555	0.42361	intergenic	ENSG00000273335	9473420-9474006	-	46414
18	9473789	9474058	270	9474018	4.28414	3.30319	0.58986	ENSG00000273335:Promoter;ENSG00000017797:Promoter	ENSG00000273335	9473420-9474006	-	83
18	9427444	9427741	298	9427581	3.94601	3.20555	0.42361	intergenic	ENSG00000273335	9473420-9474006	-	46414

18	9473789	9474058	270	9474018	4.28414	3.30319	0.58986	ENSG00000273335:Promoter;ENSG00000017797:Promoter	ENSG00000273335	9473420-9474006	-	83
18	9524644	9524936	293	9524701	5.07624	3.80169	0.93914	ENSG00000017797:exon	ENSG00000251994	9518118-9518260	-	-6529
18	10707006	10707497	492	10707378	6.49439	4.38367	1.95029	ENSG00000264843:intron;ENSG00000154864:intron	ENSG00000264843	10704296-10709599	+	2955
18	10878779	10879028	250	10878887	11.51693	6.17716	6.39216	ENSG00000154864:intron	ENSG00000263952	10893616-10908783	+	-14713
18	10955467	10955745	279	10955658	4.36824	3.47775	0.59373	ENSG00000154864:intron	ENSG00000263952	10893616-10908783	+	61989
18	11008057	11008365	309	11008142	3.77589	3.20345	0.30123	ENSG00000154864:intron	ENSG00000263952	10893616-10908783	+	114594
18	10878779	10879028	250	10878887	11.51693	6.17716	6.39216	ENSG00000154864:intron	ENSG00000263952	10893616-10908783	+	-14713
18	10955467	10955745	279	10955658	4.36824	3.47775	0.59373	ENSG00000154864:intron	ENSG00000263952	10893616-10908783	+	61989
18	11008057	11008365	309	11008142	3.77589	3.20345	0.30123	ENSG00000154864:intron	ENSG00000263952	10893616-10908783	+	114594
18	22765712	22765960	249	22765879	4.76929	3.60726	0.74587	ENSG00000265943:intron	ENSG00000101773	22798260-23026488	+	-32424
18	23401803	23402059	257	23401959	4.9037	3.69224	0.82412	ENSG00000134490:intron	ENSG00000134490	23197143-23437961	-	36030
18	24346317	24346546	230	24346375	6.98679	4.68837	2.37506	ENSG00000141447:intron	ENSG00000264365	24364786-24367984	+	-18355
18	26710539	26710770	232	26710663	7.17425	4.80518	2.52654	intergenic	ENSG00000265369	26687620-26703638	-	-7016
18	30016689	30016947	259	30016793	4.56295	3.60388	0.59373	intergenic	ENSG00000283218	30298909-30298960	+	-282091
18	30206545	30206748	204	30206614	4.1849	3.24157	0.52478	intergenic	ENSG00000283218	30298909-30298960	+	-92263
18	30400049	30400362	314	30400243	6.26578	4.40475	1.75945	intergenic	ENSG00000283218	30298909-30298960	+	101296
18	32238192	32238412	221	32238285	4.56295	3.60388	0.59373	intergenic	ENSG00000263904	32245945-32249032	-	10730
18	33809005	33809305	301	33809088	4.56295	3.60388	0.59373	intergenic	ENSG00000199204	34001697-34001799	-	192644
18	34979048	34979467	420	34979192	5.34519	3.973	1.09512	ENSG00000166974:intron	ENSG00000166974	34976927-35143470	+	2330
18	37316791	37317055	265	37316926	4.64049	3.52611	0.64891	ENSG00000101489:intron	ENSG00000279678	37305294-37306333	-	-10589
18	38911388	38911755	368	38911545	3.77589	3.20345	0.30123	intergenic	ENSG00000222704	39016046-39016344	-	104773
18	38912285	38912526	242	38912321	3.03786	2.80302	0.15111	intergenic	ENSG00000222704	39016046-39016344	-	103939
18	38940821	38941237	417	38941047	64.77042	17.53171	57.98073	intergenic	ENSG00000222704	39016046-39016344	-	75315
18	38954374	38954704	331	38954520	5.71883	4.06146	1.34558	intergenic	ENSG00000222704	39016046-39016344	-	61805
18	38911388	38911755	368	38911545	3.77589	3.20345	0.30123	intergenic	ENSG00000222704	39016046-39016344	-	104773
18	38912285	38912526	242	38912321	3.03786	2.80302	0.15111	intergenic	ENSG00000222704	39016046-39016344	-	103939
18	38940821	38941237	417	38941047	64.77042	17.53171	57.98073	intergenic	ENSG00000222704	39016046-39016344	-	75315
18	38954374	38954704	331	38954520	5.71883	4.06146	1.34558	intergenic	ENSG00000222704	39016046-39016344	-	61805
18	43244610	43244836	227	43244673	4.31475	3.20475	0.59373	intergenic	ENSG00000132872	43267877-43277650	-	32927
18	44041420	44041664	245	44041503	4.56295	3.60388	0.59373	intergenic	ENSG00000267716	44039470-44040140	+	2071
18	44709775	44710107	333	44709948	33.90416	12.197	27.85945	ENSG00000152217:intron	ENSG00000280365	44732574-44733315	+	-22633
18	44814105	44814413	309	44814195	4.84177	3.65305	0.77787	ENSG00000152217:intron	ENSG00000280365	44732574-44733315	+	81684
18	44709775	44710107	333	44709948	33.90416	12.197	27.85945	ENSG00000152217:intron	ENSG00000280365	44732574-44733315	+	-22633
18	44814105	44814413	309	44814195	4.84177	3.65305	0.77787	ENSG00000152217:intron	ENSG00000280365	44732574-44733315	+	81684
18	44851335	44851581	247	44851489	7.3143	4.5382	2.6406	ENSG00000152217:intron	ENSG00000265957	44970081-44970166	-	118708
18	44953869	44954072	204	44954001	3.56795	3.06614	0.30123	ENSG00000152217:intron	ENSG00000265957	44970081-44970166	-	16196
18	44969305	44969522	218	44969457	5.68188	3.88989	1.31322	ENSG00000152217:intron	ENSG00000265957	44970081-44970166	-	753
18	48834718	48835028	311	48834881	10.16212	5.61542	5.15724	ENSG00000267764:Promoter:ENSG00000134030:intron	ENSG00000267764	48826050-48834770	-	-102
18	50080934	50081159	226	50081071	5.6497	4.01836	1.29426	ENSG00000167306:intron	ENSG00000267511	50063794-50064440	+	17252
18	50131717	50131965	249	50131836	6.5896	4.44231	2.02209	ENSG00000167306:intron	ENSG00000283343	50126498-50126563	-	-5277
18	50875107	50875352	246	50875274	4.74614	3.46321	0.73612	intergenic	ENSG00000082212	50878733-50954257	+	-3504
18	53642087	53642375	289	53642225	3.77589	3.20345	0.30123	intergenic	ENSG00000242945	53605253-53605725	-	-36505
18	53689515	53689914	400	53689734	3.658	3.12558	0.30123	intergenic	ENSG00000242945	53605253-53605725	-	-83989
18	53834868	53835163	296	53835059	4.56295	3.60388	0.59373	intergenic	ENSG00000242945	53605253-53605725	-	-229290
18	53854454	53854686	233	53854647	3.658	3.12558	0.30123	intergenic	ENSG00000242945	53605253-53605725	-	-248844
18	53642087	53642375	289	53642225	3.77589	3.20345	0.30123	intergenic	ENSG00000242945	53605253-53605725	-	-36505
18	53689515	53689914	400	53689734	3.658	3.12558	0.30123	intergenic	ENSG00000242945	53605253-53605725	-	-83989

18	53834868	53835163	296	53835059	4.56295	3.60388	0.59373	intergenic	ENSG00000242945	53605253-53605725	-	-229290
18	53854454	53854686	233	53854647	3.658	3.12558	0.30123	intergenic	ENSG00000242945	53605253-53605725	-	-248844
18	54388827	54389057	231	54388928	8.62151	5.12271	3.79257	intergenic	ENSG00000264350	54406888-54407114	-	18172
18	54717741	54718116	376	54717922	5.68188	3.88989	1.31322	ENSG00000041353:five_prime_UTR;ENSG00000041353:exon	ENSG00000041353	54717859-54895516	+	69
18	54754058	54754267	210	54754124	3.77589	3.20345	0.30123	ENSG00000041353:intron	ENSG00000267524	54744749-54745581	+	9413
18	54769136	54769494	359	54769408	4.56295	3.60388	0.59373	ENSG00000041353:intron	ENSG00000267524	54744749-54745581	+	24565
18	56581661	56581917	257	56581742	7.17425	4.80518	2.52654	intergenic	ENSG00000091157	56651342-57036606	+	-69553
18	57044205	57044560	356	57044390	4.11517	3.31434	0.49781	intergenic	ENSG00000267225	57027831-57038845	+	16551
18	57127960	57128197	238	57128137	3.55536	3.05783	0.30123	intergenic	ENSG00000267789	57136321-57146998	-	18920
18	58426947	58427198	252	58427062	3.30549	2.89321	0.30123	intergenic	ENSG00000267675	58416764-58417628	+	10308
18	58535500	58535747	248	58535705	5.06016	3.79147	0.9304	ENSG00000198796:exon;ENSG00000267257:intron	ENSG00000267257	58535414-58538552	+	209
18	62027939	62028556	618	62028193	3.40931	2.96154	0.30123	ENSG00000197563:intron	ENSG00000267597	61957272-61958014	-	-70233
18	63083726	63083931	206	63083871	3.02874	2.79685	0.15111	intergenic	ENSG00000267766	63151113-63151320	-	67492
18	63084621	63084861	241	63084666	4.41276	3.50657	0.59373	intergenic	ENSG00000267766	63151113-63151320	-	66579
18	63159546	63159857	312	63159801	3.12148	2.77238	0.20992	ENSG00000171791:intron	ENSG00000267766	63151113-63151320	-	-8381
18	63161203	63161416	214	63161277	3.20396	2.64557	0.26167	ENSG00000171791:intron	ENSG00000267766	63151113-63151320	-	-9989
18	63161748	63162043	296	63161909	11.02474	6.11671	5.94462	ENSG00000171791:intron	ENSG00000267766	63151113-63151320	-	-10575
18	63083726	63083931	206	63083871	3.02874	2.79685	0.15111	intergenic	ENSG00000267766	63151113-63151320	-	67492
18	63084621	63084861	241	63084666	4.41276	3.50657	0.59373	intergenic	ENSG00000267766	63151113-63151320	-	66579
18	63159546	63159857	312	63159801	3.12148	2.77238	0.20992	ENSG00000171791:intron	ENSG00000267766	63151113-63151320	-	-8381
18	63161203	63161416	214	63161277	3.20396	2.64557	0.26167	ENSG00000171791:intron	ENSG00000267766	63151113-63151320	-	-9989
18	63161748	63162043	296	63161909	11.02474	6.11671	5.94462	ENSG00000171791:intron	ENSG00000267766	63151113-63151320	-	-10575
18	64004761	64005111	351	64004940	5.96569	4.21594	1.54281	ENSG00000166401:intron	ENSG00000283667	63966405-63970181	-	-34754
18	64157907	64158142	236	64158003	5.39424	4.00432	1.09512	ENSG00000267134:intron	ENSG00000179676	64080008-64149030	-	-8994
18	64199046	64199277	232	64199135	5.14139	3.84312	0.98688	ENSG00000266952:intron;ENSG00000267134:intron	ENSG00000179676	64080008-64149030	-	-50131
18	66168164	66168449	286	66168352	5.39424	4.00432	1.09512	intergenic	ENSG00000264685	66068749-66069647	-	-98659
18	66847292	66847540	249	66847376	4.47325	3.54574	0.59373	intergenic	ENSG00000238680	66946115-66946222	+	-98699
18	68727602	68727817	216	68727661	4.919	3.70193	0.82412	intergenic	ENSG00000276092	68721081-68721560	+	6628
18	69631527	69631965	439	69631674	6.26578	4.40475	1.75945	ENSG00000206052:intron	ENSG00000265643	69483626-69618694	-	-13051
18	70194219	70194551	333	70194423	4.919	3.70193	0.82412	ENSG00000176225:intron	ENSG00000176225	70003030-70205945	-	11560
18	70736875	70737207	333	70737027	4.56295	3.60388	0.59373	intergenic	ENSG00000263417	70630533-70650744	-	-86296
18	70773867	70774100	234	70774011	4.1849	3.24157	0.52478	intergenic	ENSG00000263417	70630533-70650744	-	-123239
18	71989964	71990205	242	71990081	4.84177	3.65305	0.77787	intergenic	ENSG00000265995	71890408-71944577	-	-45507
18	71994111	71994335	225	71994161	3.77589	3.20345	0.30123	intergenic	ENSG00000265995	71890408-71944577	-	-49645
18	72181275	72181540	266	72181471	5.01585	3.49609	0.89533	intergenic	ENSG00000265995	71890408-71944577	-	-236830
18	71989964	71990205	242	71990081	4.84177	3.65305	0.77787	intergenic	ENSG00000265995	71890408-71944577	-	-45507
18	71994111	71994335	225	71994161	3.77589	3.20345	0.30123	intergenic	ENSG00000265995	71890408-71944577	-	-49645
18	72181275	72181540	266	72181471	5.01585	3.49609	0.89533	intergenic	ENSG00000265995	71890408-71944577	-	-236830
18	73396351	73396572	222	73396502	5.29302	3.93971	1.09512	intergenic	ENSG00000261780	73324940-73349878	+	71521
18	74001455	74001730	276	74001483	4.21084	3.37603	0.54478	ENSG00000263655:intron	ENSG00000263655	73914404-74034161	-	32569
18	77905538	77905743	206	77905681	3.49438	2.91742	0.30123	intergenic	ENSG00000252260	77863463-77863567	-	-42073
18	77910333	77910582	250	77910513	5.39424	4.00432	1.09512	intergenic	ENSG00000252260	77863463-77863567	-	-46890
18	79300327	79300645	319	79300459	5.39424	4.00432	1.09512	ENSG00000166377:intron	ENSG00000267015	79340257-79343972	-	43486
18	79325500	79325730	231	79325566	4.56295	3.60388	0.59373	ENSG00000166377:intron	ENSG00000267015	79340257-79343972	-	18357
19	2865860	2866087	228	2865952	5.968	3.64162	1.54447	ENSG00000172000:Promoter	ENSG00000172000	2867334-2883445	+	-1361
19	3435154	3435417	264	3435278	13.45783	4.60431	8.16847	ENSG00000141905:intron	ENSG00000095932	3473985-3480542	-	45257
19	7080293	7080623	331	7080611	5.39672	3.71993	1.09738	ENSG00000130544:intron	ENSG00000130544	7069443-7087968	+	11014

19	7147324	7147540	217	7147429	8.23437	4.24593	3.43385	ENSG00000171105:intron	ENSG00000130544	7069443-7087968	+	77988
19	10412285	10412509	225	10412431	5.84361	3.7038	1.44168	ENSG00000105401:intron	ENSG00000065989	10416772-10469631	+	-4375
19	10615048	10615302	255	10615066	4.91514	3.56573	0.82412	ENSG00000129353:intron	ENSG00000129353	10602456-10644559	+	12718
19	11536001	11536237	237	11536179	7.01358	3.67516	2.39889	intergenic	ENSG00000130176	11538716-11550323	+	-2597
19	11987929	11988152	224	11988042	5.11088	3.68528	0.9653	ENSG00000219665:intron	ENSG00000219665	11987616-12046275	+	424
19	13323335	13323582	248	13323463	4.56295	3.60388	0.59373	ENSG00000141837:intron	ENSG00000213253	13157958-13158788	-	-164670
19	13516477	13516747	271	13516588	4.42776	3.51628	0.59373	intergenic	ENSG00000141837	13206441-13633025	-	116413
19	14006864	14007087	224	14006998	5.13473	3.43925	0.98202	ENSG00000132005:Promoter	ENSG00000132005	13961537-14007039	-	64
19	15688036	15688312	277	15688175	4.56295	3.60388	0.59373	ENSG00000186204:intron	ENSG00000186204	15672756-15697174	+	15417
19	15883587	15883838	252	15883644	5.74381	3.92704	1.36222	ENSG00000186115:intron	ENSG00000273330	15871666-15871852	-	-11860
19	16929174	16929434	261	16929234	8.28323	5.11428	3.47693	ENSG00000160111:exon	ENSG00000263595	16910489-16910784	-	-18519
19	17135500	17135786	287	17135612	9.5439	5.46423	4.61247	ENSG00000099331:intron	ENSG00000268056	17152587-17168051	-	32408
19	18225622	18226063	442	18226059	4.18632	3.24245	0.52565	ENSG00000285188:intron;ENSG00000105650:intron	ENSG00000268650	18204729-18220480	+	21113
19	20267006	20267435	430	20267205	5.39424	4.00432	1.09512	ENSG00000269055:Promoter:ENSG00000267383:intron	ENSG00000269055	20267816-20268391	+	-596
19	23359332	23359569	238	23359388	4.76929	3.60726	0.74587	ENSG00000167232:intron	ENSG00000269431	23380316-23380876	+	-20866
19	27672527	27672744	218	27672619	6.49439	4.38367	1.95029	intergenic	ENSG00000267696	27638482-27646483	-	-26152
19	30030892	30031113	222	30031002	3.69765	3.15177	0.30123	ENSG00000267006:Promoter	ENSG00000267006	30028740-30029916	-	-1086
19	32514374	32514737	364	32514497	4.16942	3.34931	0.52478	intergenic	ENSG00000105185	32581067-32587452	+	-66512
19	33051592	33051875	284	33051727	3.34993	2.92244	0.30123	ENSG00000131941:intron	ENSG00000267611	33051514-33051701	+	219
19	34722586	34722815	230	34722715	4.84177	3.65305	0.77787	intergenic	ENSG00000274104	34733297-34733837	-	11137
19	37426568	37426863	296	37426804	3.03786	2.80302	0.15111	ENSG00000196437:intron	ENSG00000279936	37401403-37403846	-	-22869
19	37429651	37429901	251	37429701	3.77589	3.20345	0.30123	ENSG00000196437:intron	ENSG00000279936	37401403-37403846	-	-25929
19	39073466	39073910	445	39073789	6.51641	4.07877	1.96373	intergenic	ENSG00000183760	39083912-39111493	+	-10224
19	39403852	39404080	229	39404002	11.58561	4.50718	6.45179	intergenic	ENSG00000128016	39406812-39409412	+	-2846
19	41087847	41088099	253	41087924	5.01585	3.49609	0.89533	ENSG00000197838:Promoter	ENSG00000197838	41088471-41096195	+	-498
19	42209083	42209391	309	42209351	4.14216	3.33173	0.51073	ENSG00000160570:intron	ENSG00000160570	42198597-42220140	-	10903
19	44671386	44671648	263	44671599	4.47325	3.54574	0.59373	ENSG00000186567:exon;ENSG00000186567:five_prime_UTR;ENSG00000266903:intron	ENSG00000279095	44664130-44666158	+	7386
19	46160935	46161199	265	46161054	6.22868	4.22079	1.75945	ENSG00000204866:intron;ENSG00000267922:intron	ENSG00000204866	46143105-46161299	+	17961
19	47241083	47241351	269	47241228	9.05733	4.35472	4.17411	intergenic	ENSG00000105327	47220821-47232766	-	-8450
19	48978873	48979165	293	48978950	23.13206	10.02345	17.39519	ENSG00000104812:intron	ENSG00000087086	48965300-48966878	+	13718
19	51955631	51955924	294	51955899	4.28127	3.42152	0.58762	ENSG00000269235:intron	ENSG00000269235	51949133-51981367	+	6644
19	55881576	55881841	266	55881737	4.56295	3.60388	0.59373	ENSG00000160505:exon;ENSG00000160505:three_prime_UTR	ENSG00000179873	55785396-55836800	-	-44908
19	57283285	57283500	216	57283426	6.36907	4.30669	1.85528	ENSG00000197714:intron	ENSG00000267871	57267219-57280334	-	-3058
19	57472620	57473034	415	57472797	4.56295	3.60388	0.59373	ENSG00000268163:intron;ENSG00000197128:intron	ENSG00000268163	57437969-57477536	-	4709
2	783906	784202	297	783972	4.56295	3.60388	0.59373	ENSG00000237667:intron	ENSG00000223751	742487-747767	+	41566
2	8031942	8032169	228	8032000	4.94982	3.72146	0.84841	ENSG00000235665:intron;ENSG00000236790:intron	ENSG00000229740	8139401-8143269	+	-107346
2	8115464	8115668	205	8115561	3.88371	3.16559	0.37576	ENSG00000236790:intron;ENSG00000235665:intron	ENSG00000229740	8139401-8143269	+	-23835
2	8302034	8302308	275	8302197	18.22361	8.18134	12.65373	ENSG00000236790:intron	ENSG00000235665	7922424-8278084	-	-24086
2	8966862	8967166	305	8966934	4.14216	3.33173	0.51073	ENSG00000143797:intron	ENSG00000143797	8852689-9003813	-	36799
2	9914718	9915003	286	9914833	8.17554	4.86236	3.38452	ENSG00000115750:intron	ENSG00000269973	9936359-9939590	+	-21499

2	10852156	10852408	253	10852372	3.49438	2.91742	0.30123	ENSG00000232056:intron	ENSG00000232056	10847576-10854955	+	4705
2	11260150	11260369	220	11260191	3.69765	3.15177	0.30123	ENSG00000134318:intron	ENSG00000207267	11233987-11234094	+	26272
2	11754294	11754535	242	11754412	8.49733	4.52572	3.67671	ENSG00000134324:intron	ENSG00000230790	11740996-11745301	-	-9113
2	11830285	11830561	277	11830395	16.55362	7.28351	11.05902	intergenic	ENSG00000265172	11836932-11836986	-	6563
2	12460526	12460747	222	12460726	3.77589	3.20345	0.30123	ENSG00000224184:intron	ENSG00000207183	12411397-12411501	-	-49135
2	12501529	12501787	259	12501610	6.21114	4.3703	1.75228	ENSG00000224184:intron	ENSG00000207183	12411397-12411501	-	-90156
2	13432247	13432614	368	13432440	5.68188	3.88989	1.31322	intergenic	ENSG00000229370	13537672-13609168	+	-105242
2	13523239	13523525	287	13523377	16.55362	7.28351	11.05902	intergenic	ENSG00000229370	13537672-13609168	+	-14290
2	13432247	13432614	368	13432440	5.68188	3.88989	1.31322	intergenic	ENSG00000229370	13537672-13609168	+	-105242
2	13523239	13523525	287	13523377	16.55362	7.28351	11.05902	intergenic	ENSG00000229370	13537672-13609168	+	-14290
2	13666961	13667214	254	13667009	4.84177	3.65305	0.77787	intergenic	ENSG00000227718	13723047-13758152	+	-55960
2	13792839	13793060	222	13792973	4.35356	3.46825	0.59373	ENSG00000230448:intron	ENSG00000227718	13723047-13758152	+	69902
2	13666961	13667214	254	13667009	4.84177	3.65305	0.77787	intergenic	ENSG00000227718	13723047-13758152	+	-55960
2	13792839	13793060	222	13792973	4.35356	3.46825	0.59373	ENSG00000230448:intron	ENSG00000227718	13723047-13758152	+	69902
2	15984511	15984808	298	15984678	3.64836	3.01523	0.30123	intergenic	ENSG00000243541	15950688-15950981	-	-33678
2	17255920	17256223	304	17255990	4.56295	3.60388	0.59373	intergenic	ENSG00000214843	17284291-17284895	+	-28220
2	17303094	17303388	295	17303255	7.95772	5.10736	3.21843	intergenic	ENSG00000214843	17284291-17284895	+	18949
2	17626923	17627205	283	17626977	6.09579	4.29768	1.6498	ENSG00000163032:intron	ENSG00000163032	17539125-17657018	+	87938
2	17813763	17814063	301	17813893	6.22868	4.22079	1.75945	intergenic	ENSG00000151379	17816495-17817101	+	-2582
2	17927829	17928044	216	17927874	3.64493	3.11695	0.30123	ENSG00000170745:intron	ENSG00000170745	17877846-18361616	+	50090
2	20929877	20930103	227	20930060	4.68282	3.55276	0.68513	intergenic	ENSG00000236436	20859770-20861661	+	70219
2	21108093	21108303	211	21108163	7.3143	4.5382	2.6406	intergenic	ENSG00000218819	21123916-21143272	+	-15718
2	22716230	22716502	273	22716295	3.77589	3.20345	0.30123	intergenic	ENSG00000222616	22861551-22861884	+	-145185
2	22947302	22947545	244	22947527	4.56295	3.60388	0.59373	intergenic	ENSG00000222616	22861551-22861884	+	85872
2	23714536	23714804	269	23714626	4.81345	3.63515	0.77787	intergenic	ENSG00000283031	23667207-23685453	-	-29216
2	26068627	26068935	309	26068758	6.24595	4.23134	1.75945	ENSG00000084733:intron	ENSG00000199872	26042674-26042781	+	26106
2	28332928	28333314	387	28333070	6.26578	4.40475	1.75945	ENSG00000158019:intron	ENSG00000223522	28307690-28310459	-	-22661
2	28392713	28392999	287	28392845	9.86288	4.19314	4.89167	ENSG00000075426:intron;ENSG00000229951:intron	ENSG00000075426	28392447-28417312	+	408
2	30640606	30641015	410	30640622	4.51948	3.5757	0.59373	ENSG00000172954:three_prime_UTR;ENSG00000172954:exon	ENSG00000278908	30677761-30679895	+	-36951
2	31249113	31249357	245	31249280	5.92936	4.19316	1.51421	ENSG00000013016:intron	ENSG00000013016	31234336-31269447	+	14898
2	32418180	32418457	278	32418275	4.12863	3.32301	0.50868	ENSG00000115760:intron	ENSG00000230046	32377630-32379599	-	-38719
2	33186804	33187118	315	33186965	7.00771	4.7014	2.39483	ENSG00000049323:exon	ENSG00000285577	33274464-33281261	-	94300
2	36492372	36492696	325	36492482	3.03786	2.80302	0.15111	ENSG00000150938:intron	ENSG00000273090	36513254-36513732	-	21198
2	36516461	36516734	274	36516676	4.56295	3.60388	0.59373	ENSG00000150938:intron	ENSG00000273090	36513254-36513732	-	-2865
2	37131455	37131675	221	37131555	4.70801	3.44018	0.70461	ENSG00000055332:intron	ENSG00000224891	37148529-37149304	+	-16964
2	39128961	39129284	324	39129087	7.74622	4.7928	3.02341	ENSG00000202309:Promoter	ENSG00000202309	39128825-39128927	-	-195
2	43293878	43294168	291	43293997	5.40704	3.35205	1.10135	ENSG00000115970:intron	ENSG00000234936	43229572-43233394	+	64450
2	45453922	45454196	275	45454078	4.56295	3.60388	0.59373	ENSG00000068784:intron	ENSG00000239396	45569198-45569492	-	115433
2	45869853	45870152	300	45870076	3.88371	3.16559	0.37576	ENSG00000171132:intron	ENSG00000233829	46003941-46004309	-	134307
2	46413195	46413513	319	46413355	5.75051	3.78743	1.36222	intergenic	ENSG00000187600	46429189-46441833	+	-15835
2	46493550	46493825	276	46493713	11.57675	5.57827	6.44432	ENSG00000250565:intron	ENSG00000253515	46499730-46501278	+	-6043
2	46500238	46500449	212	46500320	4.31475	3.20475	0.59373	ENSG00000250565:intron;ENSG00000253515:intron	ENSG00000253515	46499730-46501278	+	613
2	46909773	46910555	783	46909927	17.58419	7.57485	12.04595	ENSG00000180398:intron	ENSG00000068724	46916156-47076137	+	-5992
2	46985181	46985449	269	46985323	4.60755	3.1497	0.62762	ENSG00000068724:intron	ENSG00000272814	46956614-46956888	-	-28426
2	48669963	48670214	252	48670197	3.49438	2.91742	0.30123	ENSG00000242441:intron;ENSG00000068781:intron;ENSG00000279956:intron	ENSG00000239995	48632855-48633353	-	-36735

2	48997375	48997599	225	48997465	4.50398	3.56566	0.59373	ENSG00000282890:intron;ENSG00000170820:intron	ENSG00000212036	49059602-49059658	+	-62115
2	49042341	49042575	235	49042476	3.03786	2.80302	0.15111	ENSG00000282890:intron;ENSG00000170820:intron	ENSG00000212036	49059602-49059658	+	-17144
2	49055511	49055808	298	49055620	7.17425	4.80518	2.52654	ENSG00000282890:intron;ENSG00000170820:intron	ENSG00000212036	49059602-49059658	+	-3943
2	49579961	49580285	325	49580136	5.39424	4.00432	1.09512	ENSG00000282828:intron	ENSG00000282828	49563397-49595122	+	16725
2	49728112	49728350	239	49728280	3.77589	3.20345	0.30123	intergenic	ENSG00000234253	49878622-49879363	+	-150391
2	50229929	50230190	262	50230091	3.77589	3.20345	0.30123	ENSG00000179915:intron	ENSG00000285548	50324642-50345598	+	-94583
2	50294648	50294902	255	50294678	3.34993	2.92244	0.30123	ENSG00000179915:intron	ENSG00000285548	50324642-50345598	+	-29867
2	53964698	53964915	218	53964785	4.11506	3.19834	0.49781	ENSG00000068878:intron	ENSG00000170634	53970837-54305300	+	-6031
2	54093178	54093414	237	54093326	5.0124	3.76115	0.89533	ENSG00000170634:intron	ENSG00000272156	54082553-54085066	+	10742
2	55050466	55050693	228	55050565	8.2371	4.24725	3.43644	ENSG00000115310:intron	ENSG00000200086	55014417-55014521	−	-36058
2	56934917	56935198	282	56934986	5.39424	4.00432	1.09512	intergenic	ENSG00000271115	57048349-57049336	−	114279
2	61708104	61708336	233	61708227	5.56876	3.96799	1.238	intergenic	ENSG00000213486	61710075-61710978	−	2758
2	62306722	62306950	229	62306868	5.75051	3.78743	1.36222	intergenic	ENSG00000270335	62348947-62350739	−	43903
2	62403890	62404108	219	62404079	3.77589	3.20345	0.30123	intergenic	ENSG00000270335	62348947-62350739	−	-53259
2	62306722	62306950	229	62306868	5.75051	3.78743	1.36222	intergenic	ENSG00000270335	62348947-62350739	−	43903
2	62403890	62404108	219	62404079	3.77589	3.20345	0.30123	intergenic	ENSG00000270335	62348947-62350739	−	-53259
2	62569787	62570021	235	62569863	3.34993	2.92244	0.30123	intergenic	ENSG00000230595	62561057-62561423	−	-8480
2	62656268	62656506	239	62656406	5.27581	3.92874	1.08581	ENSG00000226622:intron	ENSG00000226622	62590254-62662654	−	6267
2	63868485	63868702	218	63868627	4.82831	3.64454	0.77787	ENSG00000169764:intron	ENSG00000251775	63883248-63883391	−	14798
2	64062884	64063121	238	64062985	8.17554	4.86236	3.38452	intergenic	ENSG00000228079	64086352-64088246	−	25244
2	64114101	64114323	223	64114208	6.4824	4.21404	1.94623	ENSG00000197329:intron	ENSG00000228079	64086352-64088246	−	-25965
2	64558356	64558719	364	64558596	3.02441	2.62206	0.15111	ENSG00000119844:intron	ENSG00000252414	64578891-64578997	+	-20354
2	65167127	65167358	232	65167215	4.81345	3.63515	0.77787	intergenic	ENSG00000272025	65158661-65158860	+	8581
2	67290617	67290829	213	67290714	3.03786	2.80302	0.15111	ENSG00000235885:intron	ENSG00000236605	67324626-67325304	+	-33903
2	67346627	67346878	252	67346695	4.00982	3.24654	0.45978	intergenic	ENSG00000236605	67324626-67325304	+	22126
2	67463830	67464300	471	67464037	15.49818	8.00863	10.06014	intergenic	ENSG00000143971	67397318-67412089	+	66746
2	68516383	68516626	244	68516489	3.77589	3.20345	0.30123	ENSG00000169621:intron	ENSG00000214525	68528240-68529452	−	12948
2	68550956	68551238	283	68551150	3.69765	3.15177	0.30123	ENSG00000169621:intron	ENSG00000214525	68528240-68529452	−	-21644
2	68832607	68832903	297	68832712	9.5439	5.46423	4.61247	ENSG00000237576:intron	ENSG00000237576	68832043-68837724	+	711
2	74593756	74593990	235	74593930	4.26704	3.41232	0.58491	ENSG00000159374:intron	ENSG00000233872	74618855-74620979	−	27106
2	75747653	75748069	417	75748038	4.56295	3.60388	0.59373	intergenic	ENSG00000270996	75719119-75720018	+	28741
2	75778764	75779058	295	75778904	4.56295	3.60388	0.59373	intergenic	ENSG00000270996	75719119-75720018	+	59791
2	83884496	83884763	268	83884640	4.56295	3.60388	0.59373	intergenic	ENSG00000228225	84031139-84032358	+	-146510
2	83943262	83943496	235	83943393	4.84177	3.65305	0.77787	intergenic	ENSG00000228225	84031139-84032358	+	-87760
2	85930746	85931030	285	85931003	4.61259	3.50857	0.63149	intergenic	ENSG00000115525	85837119-85905199	−	-25688
2	85999318	85999570	253	85999452	5.01585	3.49609	0.89533	intergenic	ENSG00000115525	85837119-85905199	−	-94244
2	85930746	85931030	285	85931003	4.61259	3.50857	0.63149	intergenic	ENSG00000115525	85837119-85905199	−	-25688
2	85999318	85999570	253	85999452	5.01585	3.49609	0.89533	intergenic	ENSG00000115525	85837119-85905199	−	-94244
2	86251337	86251613	277	86251433	3.45699	2.99296	0.30123	ENSG00000068615:intron	ENSG00000132313	86199354-86213794	+	52120
2	86486854	86487071	218	86487029	3.88371	3.16559	0.37576	ENSG00000115548:intron	ENSG00000200563	86515203-86515308	−	28346
2	88119036	88119301	266	88119152	6.19436	4.19984	1.73845	intergenic	ENSG00000163586	88122981-88128116	−	8948
2	88182674	88182895	222	88182829	4.68282	3.55276	0.68513	ENSG00000144115:exon	ENSG00000144115	88170294-88186636	+	12490
2	88259914	88260166	253	88260024	4.69706	3.56173	0.69764	intergenic	ENSG00000144115	88170294-88186636	+	89745
2	88182674	88182895	222	88182829	4.68282	3.55276	0.68513	ENSG00000144115:exon	ENSG00000144115	88170294-88186636	+	12490
2	88259914	88260166	253	88260024	4.69706	3.56173	0.69764	intergenic	ENSG00000144115	88170294-88186636	+	89745
2	88600042	88600474	433	88600301	12.43382	5.82026	7.22732	ENSG00000172071:intron	ENSG00000234028	88627538-88631821	+	-27280
2	94291509	94291871	363	94291644	3.77589	3.20345	0.30123	intergenic	ENSG00000261522	94575975-94587183	−	295493
2	95159229	95159548	320	95159478	4.23481	3.27254	0.56211	ENSG00000144026:intron	ENSG00000144026	95147329-95165413	−	6025

2	96333768	96333987	220	96333885	3.95541	3.09995	0.4326	ENSG00000121152:Promoter	ENSG00000121152	96335786-96373845	+	-1909
2	97048568	97048803	236	97048712	5.39424	4.00432	1.09512	intergenic	ENSG00000271402	97050728-97051028	+	-2043
2	97111178	97111477	300	97111254	5.39424	4.00432	1.09512	intergenic	ENSG00000135976	97113495-97250199	+	-2168
2	97111178	97111477	300	97111254	5.39424	4.00432	1.09512	intergenic	ENSG00000135976	97113495-97250199	+	-2168
2	97821534	97821857	324	97821739	4.78394	3.61651	0.75881	ENSG00000075568:intron	ENSG00000235734	97827247-97827545	+	-5552
2	98711718	98711978	261	98711824	4.16942	3.34931	0.52478	ENSG00000071073:intron	ENSG00000236564	98694108-98695490	+	17739
2	101622317	101622626	310	101622395	3.34993	2.92244	0.30123	intergenic	ENSG00000071054	101696849-101894689	+	-74378
2	104343507	104343723	217	104343586	4.76929	3.60726	0.74587	intergenic	ENSG00000233955	104395001-104396280	-	52665
2	107027234	107027546	313	107027421	4.56295	3.60388	0.59373	intergenic	ENSG00000226905	106963192-106963674	+	64197
2	107089611	107089919	309	107089653	4.56295	3.60388	0.59373	intergenic	ENSG00000226905	106963192-106963674	+	126572
2	107279166	107279389	224	107279193	3.77589	3.20345	0.30123	ENSG00000229457:intron	ENSG00000225588	107362281-107407329	+	-83004
2	107303890	107304225	336	107304084	3.34993	2.92244	0.30123	ENSG00000229457:intron	ENSG00000225588	107362281-107407329	+	-58224
2	107414443	107414938	496	107414770	5.39424	4.00432	1.09512	ENSG00000237880:intron	ENSG00000229457	107254690-107365873	-	-48817
2	109213890	109214158	269	109214002	3.56683	2.68164	0.30123	ENSG00000172985:intron	ENSG00000228551	109251421-109251646	+	-37397
2	109341395	109341657	263	109341520	10.65162	5.29115	5.60305	ENSG00000172985:intron	ENSG00000265965	109313570-109313625	-	-27900
2	109429690	109430027	338	109429874	5.42487	3.87873	1.11519	ENSG00000172985:intron	ENSG00000265965	109313570-109313625	-	-116233
2	109341395	109341657	263	109341520	10.65162	5.29115	5.60305	ENSG00000172985:intron	ENSG00000265965	109313570-109313625	-	-27900
2	109429690	109430027	338	109429874	5.42487	3.87873	1.11519	ENSG00000172985:intron	ENSG00000265965	109313570-109313625	-	-116233
2	110139363	110139795	433	110139600	3.81246	2.91013	0.32594	ENSG00000144061:intron	ENSG00000144063	110083869-110116566	-	-23012
2	110748406	110748678	273	110748572	7.47982	4.63532	2.78061	ENSG00000153093:intron	ENSG00000153093	110732572-111118222	+	15969
2	111705818	111706120	303	111705996	10.64217	4.64231	5.60051	ENSG00000153107:intron	ENSG00000285016	111429323-111699033	-	-6935
2	111847050	111847401	352	111847312	3.77589	3.20345	0.30123	intergenic	ENSG00000230958	111865797-111866405	+	-18572
2	116320495	116320744	250	116320685	5.24168	3.90698	1.06394	intergenic	ENSG00000271667	116687217-116687387	-	366768
2	116500003	116500294	292	116500178	4.56295	3.60388	0.59373	intergenic	ENSG00000271667	116687217-116687387	-	187239
2	116689106	116689312	207	116689206	4.42776	3.51628	0.59373	ENSG00000271667:Promoter	ENSG00000271667	116687217-116687387	-	-1821
2	117032019	117032260	242	117032194	5.68188	3.88989	1.31322	intergenic	ENSG00000228898	117026536-117027024	+	5603
2	121475103	121475332	230	121475325	4.1849	3.24157	0.52478	ENSG00000074054:intron	ENSG00000265451	121530421-121532705	+	-55204
2	121532956	121533180	225	121533053	5.60156	3.98839	1.26447	ENSG00000074054:intron	ENSG00000264229	121530880-121531007	+	2187
2	127467398	127467601	204	127467556	3.658	3.12558	0.30123	ENSG00000231731:Promoter :ENSG00000163166:intron	ENSG00000202429	127472233-127472415	+	-4734
2	127489033	127489278	246	127489133	4.29558	3.43077	0.59373	ENSG00000231731:intron	ENSG00000202429	127472233-127472415	+	16922
2	130631860	130632065	206	130631984	4.56295	3.60388	0.59373	ENSG00000222038:intron	ENSG00000251757	130622837-130622944	+	9125
2	133344470	133344759	290	133344579	3.77589	3.20345	0.30123	ENSG00000176771:intron	ENSG00000252688	133333828-133333930	+	10786
2	134299140	134299376	237	134299287	6.26578	4.40475	1.75945	ENSG00000152127:intron	ENSG00000234060	134319653-134320078	-	20820
2	134486225	134486459	235	134486257	7.77693	4.99605	3.05159	ENSG00000152128:intron	ENSG00000234060	134319653-134320078	-	-166263
2	135400541	135400843	303	135400823	4.84324	3.65398	0.77787	ENSG00000121988:intron	ENSG00000235651	135510545-135511943	-	111251
2	136242629	136242937	309	136242802	4.69706	3.56173	0.69764	intergenic	ENSG00000279024	136230752-136233245	+	12030
2	138354236	138354472	237	138354341	3.34993	2.92244	0.30123	intergenic	ENSG00000237939	138307318-138307970	+	47035
2	140797784	140798040	257	140797919	21.09374	9.49794	15.42546	ENSG00000168702:intron	ENSG00000238065	140898422-140899106	+	-100510
2	140941766	140942039	274	140941848	3.03786	2.80302	0.15111	ENSG00000168702:intron	ENSG00000238065	140898422-140899106	+	43480
2	142895744	142896078	335	142895930	5.39424	4.00432	1.09512	ENSG00000115919:intron	ENSG00000115919	142877497-143055832	+	18413
2	147298103	147298353	251	147298148	4.42776	3.51628	0.59373	intergenic	ENSG00000207225	147408341-147408448	+	-110113
2	147503680	147504139	460	147503737	4.56295	3.60388	0.59373	intergenic	ENSG00000202074	147495437-147495540	-	-8369
2	147561901	147562105	205	147561925	3.77589	3.20345	0.30123	intergenic	ENSG00000202074	147495437-147495540	-	-66462
2	150133600	150134002	403	150133851	21.43246	10.01079	15.75174	intergenic	ENSG00000230645	150171422-150301080	+	-37621
2	150172684	150173081	398	150173058	3.73797	3.1784	0.30123	ENSG00000230645:intron	ENSG00000230645	150171422-150301080	+	1460
2	150361698	150361942	245	150361790	4.21084	3.37603	0.54478	intergenic	ENSG00000231420	150234891-150257007	-	-104812
2	152101537	152101783	247	152101566	4.72575	3.5798	0.71873	ENSG00000225214:intron	ENSG00000182389	151832767-152099475	-	-2184
2	153168430	153168646	217	153168578	4.32442	3.44941	0.59373	intergenic	ENSG00000214025	153158937-153159683	+	9600
2	153177858	153178065	208	153178007	3.77589	3.20345	0.30123	ENSG00000224612:intron	ENSG00000214025	153158937-153159683	+	19024
2	153621454	153621719	266	153621574	5.68188	3.88989	1.31322	intergenic	ENSG00000227400	153421615-153593288	-	-28298

2	156856803	156857069	267	156856952	5.25679	3.77493	1.07467	intergenic	ENSG00000238069	156777705-156778372	-	-78563
2	156908911	156909114	204	156908959	4.56295	3.60388	0.59373	intergenic	ENSG00000238069	156777705-156778372	-	-130640
2	158071882	158072206	325	158072014	5.6497	4.01836	1.29426	ENSG00000007001:intron	ENSG00000231158	158065544-158066059	+	6499
2	161104259	161104516	258	161104400	9.88148	5.87674	4.90794	ENSG00000224467:intron	ENSG00000136560	161136907-161236221	+	-32520
2	162126536	162126749	214	162126682	4.49014	3.43176	0.59373	intergenic	ENSG00000115263	162142872-162152404	-	25762
2	164025542	164025799	258	164025674	6.49439	4.38367	1.95029	ENSG00000237844:intron	ENSG00000226847	164213538-164214414	+	-187868
2	164536414	164536688	275	164536533	4.79866	3.62581	0.77111	ENSG00000115290:intron	ENSG00000277998	164573740-164574054	+	-37189
2	164896706	164896997	292	164896824	18.08667	8.67376	12.52296	ENSG00000169507:three_prime_UTR;ENSG00000169507:exon;ENSG00000223318:Promoter;ENSG00000236283:intron	ENSG00000223318	164895676-164895777	-	-1074
2	164910154	164910362	209	164910212	6.38722	4.15784	1.8702	ENSG00000169507:intron;ENSG00000236283:intron	ENSG00000223318	164895676-164895777	-	-14480
2	165593151	165593432	282	165593330	4.94982	3.72146	0.84841	ENSG00000178662:intron	ENSG00000178662	165469646-165689407	+	123645
2	165658259	165658655	397	165658619	3.49438	2.91742	0.30123	ENSG00000178662:intron	ENSG00000115339	165747590-165794682	-	136225
2	165966338	165966625	288	165966523	5.39424	4.00432	1.09512	intergenic	ENSG00000236107	165957398-166301784	+	9083
2	168339237	168339505	269	168339419	3.2062	2.82796	0.26185	intergenic	ENSG00000198648	167954019-168248141	-	-91229
2	168489988	168490241	254	168490138	6.26578	4.40475	1.75945	ENSG00000172292:intron	ENSG00000172292	168455861-168775137	+	34253
2	168495577	168495839	263	168495678	7.114	4.7676	2.48944	ENSG00000172292:intron	ENSG00000172292	168455861-168775137	+	39846
2	168489988	168490241	254	168490138	6.26578	4.40475	1.75945	ENSG00000172292:intron	ENSG00000172292	168455861-168775137	+	34253
2	168495577	168495839	263	168495678	7.114	4.7676	2.48944	ENSG00000172292:intron	ENSG00000172292	168455861-168775137	+	39846
2	169218352	169218708	357	169218646	3.77589	3.20345	0.30123	ENSG00000081479:intron	ENSG00000235995	169115194-169115680	+	103335
2	171992055	171992288	234	171992199	5.66809	4.02982	1.31021	ENSG00000115840:intron	ENSG00000172878	171999582-172082430	+	-7411
2	172422651	172422867	217	172422852	3.92092	3.18945	0.4041	intergenic	ENSG00000278924	172423806-172424173	-	1414
2	173642651	173642890	240	173642795	4.74019	3.58891	0.73063	intergenic	ENSG00000237617	173705947-173706148	-	63378
2	173720714	173720938	225	173720780	4.56295	3.60388	0.59373	intergenic	ENSG00000237617	173705947-173706148	-	-14677
2	173952447	173952740	294	173952476	3.37418	2.93841	0.30123	ENSG00000172845:intron	ENSG00000172845	173906458-173965702	-	13109
2	174942105	174942431	327	174942250	3.77589	3.20345	0.30123	ENSG00000128656:intron	ENSG00000128656	174799362-175005369	-	63101
2	176593864	176594144	281	176594042	5.19114	3.87478	1.02732	intergenic	ENSG00000283203	176600979-176601052	-	7048
2	176931874	176932078	205	176931934	5.71883	4.06146	1.34558	ENSG00000206866:Promoter	ENSG00000206866	176929984-176930090	-	-1885
2	176941143	176941453	311	176941290	4.458	3.53587	0.59373	intergenic	ENSG00000206866	176929984-176930090	-	-11207
2	176986812	176987038	227	176986814	3.02441	2.62206	0.15111	intergenic	ENSG00000206866	176929984-176930090	-	-56834
2	176988161	176988434	274	176988292	5.68188	3.88989	1.31322	intergenic	ENSG00000206866	176929984-176930090	-	-58207
2	176931874	176932078	205	176931934	5.71883	4.06146	1.34558	ENSG00000206866:Promoter	ENSG00000206866	176929984-176930090	-	-1885
2	176941143	176941453	311	176941290	4.458	3.53587	0.59373	intergenic	ENSG00000206866	176929984-176930090	-	-11207
2	176986812	176987038	227	176986814	3.02441	2.62206	0.15111	intergenic	ENSG00000206866	176929984-176930090	-	-56834
2	176988161	176988434	274	176988292	5.68188	3.88989	1.31322	intergenic	ENSG00000206866	176929984-176930090	-	-58207
2	176931874	176932078	205	176931934	5.71883	4.06146	1.34558	ENSG00000206866:Promoter	ENSG00000206866	176929984-176930090	-	-1885
2	176941143	176941453	311	176941290	4.458	3.53587	0.59373	intergenic	ENSG00000206866	176929984-176930090	-	-11207
2	176986812	176987038	227	176986814	3.02441	2.62206	0.15111	intergenic	ENSG00000206866	176929984-176930090	-	-56834
2	176988161	176988434	274	176988292	5.68188	3.88989	1.31322	intergenic	ENSG00000206866	176929984-176930090	-	-58207
2	179947139	179947424	286	179947246	5.39424	4.00432	1.09512	ENSG00000163510:intron	ENSG00000280636	179934400-179934538	-	-12743
2	180070291	180070594	304	180070397	5.39424	4.00432	1.09512	intergenic	ENSG00000163510	179944875-180007113	-	-63329
2	180210887	180211119	233	180211082	3.77589	3.20345	0.30123	intergenic	ENSG00000163510	179944875-180007113	-	-203889
2	181151450	181151690	241	181151485	5.07624	3.80169	0.93914	ENSG00000234663:intron	ENSG00000238171	181076050-181105968	-	-45601
2	181255795	181256013	219	181255921	4.44284	3.52605	0.59373	ENSG00000234663:intron	ENSG00000266705	181305592-181305652	-	49748
2	181572181	181572420	240	181572379	5.2587	3.91783	1.07467	ENSG00000188452:intron	ENSG00000188452	181536675-181680665	-	108365
2	181625652	181625869	218	181625761	4.56295	3.60388	0.59373	ENSG00000188452:intron	ENSG00000188452	181536675-181680665	-	54905
2	181631510	181631724	215	181631533	3.03786	2.80302	0.15111	ENSG00000188452:intron	ENSG00000188452	181536675-181680665	-	49048
2	182310779	182311006	228	182310851	4.56295	3.60388	0.59373	ENSG00000115252:intron	ENSG00000242121	182314283-182314544	+	-3391

2	183120087	183120321	235	183120143	4.22478	3.38503	0.55558	ENSG00000163002:intron	ENSG00000163002	183117512-183161680	+	2691
2	185962660	185962937	278	185962725	6.26578	4.40475	1.75945	intergenic	ENSG00000224019	185956586-185957063	-	-5735
2	185981819	185982024	206	185981854	3.77589	3.20345	0.30123	intergenic	ENSG00000224019	185956586-185957063	-	-24858
2	186751840	186752067	228	186751957	5.01585	3.49609	0.89533	ENSG00000144369:intron	ENSG00000227227	186641338-186695287	-	-56666
2	189708357	189708634	278	189708387	3.77589	3.20345	0.30123	ENSG00000151687:intron	ENSG00000151687	189674289-189761193	+	34206
2	190902857	190903088	232	190903023	4.44284	3.52605	0.59373	ENSG00000115419:intron	ENSG00000235852	190880796-190882059	-	-20913
2	191759117	191759361	245	191759213	5.68188	3.88989	1.31322	intergenic	ENSG00000225884	191793424-191820250	+	-34185
2	192891977	192892235	259	192892193	4.07388	3.28775	0.47041	intergenic	ENSG00000278406	192775942-192776073	+	116163
2	193764884	193765148	265	193765069	5.39424	4.00432	1.09512	intergenic	ENSG00000224219	193730554-193730689	-	-34326
2	195487051	195487328	278	195487169	9.03881	5.3697	4.15943	ENSG00000271893:intron	ENSG00000202206	195514035-195514142	+	-26846
2	196930621	196930920	300	196930822	5.60156	3.98839	1.26447	intergenic	ENSG00000197121	196833003-196927796	-	-2974
2	197058426	197058667	242	197058595	3.81246	2.91013	0.32594	ENSG00000065413:intron	ENSG00000224442	197028710-197030143	-	-28403
2	197693489	197693724	236	197693535	4.31475	3.20475	0.59373	ENSG00000222017:intron	ENSG00000222017	197693105-197774823	+	501
2	197960699	197961185	487	197960882	3.02441	2.62206	0.15111	ENSG00000115896:intron	ENSG00000115896	197804701-198572581	+	156240
2	197962011	197962218	208	197962081	4.56295	3.60388	0.59373	ENSG00000115896:intron	ENSG00000115896	197804701-198572581	+	157413
2	203579260	203579514	255	203579397	7.49188	4.02246	2.78905	intergenic	ENSG00000173166	203394344-203535410	-	-43976
2	203583755	203584084	330	203583965	15.24834	6.61394	9.82777	intergenic	ENSG00000173166	203394344-203535410	-	-48509
2	203579260	203579514	255	203579397	7.49188	4.02246	2.78905	intergenic	ENSG00000173166	203394344-203535410	-	-43976
2	203583755	203584084	330	203583965	15.24834	6.61394	9.82777	intergenic	ENSG00000173166	203394344-203535410	-	-48509
2	203811703	203811938	236	203811810	6.4824	4.21404	1.94623	intergenic	ENSG00000206970	203782036-203782143	+	29784
2	203871513	203871947	435	203871682	13.20886	5.81482	7.94737	ENSG00000163599:intron	ENSG00000163599	203867770-203873965	+	3959
2	204027168	204027453	286	204027312	14.89519	6.88771	9.49496	intergenic	ENSG00000235951	204065705-204067363	+	-38395
2	204638121	204638350	230	204638263	4.56295	3.60388	0.59373	ENSG00000116117:intron	ENSG00000116117	204545792-205620162	+	92443
2	205660269	205660532	264	205660356	5.0124	3.76115	0.89533	intergenic	ENSG00000118257	205681989-205798133	+	-21589
2	206462862	206463106	245	206463021	4.07388	3.28775	0.47041	ENSG00000114948:intron	ENSG00000114948	206443538-206621130	+	19445
2	211296032	211296344	313	211296250	5.39424	4.00432	1.09512	intergenic	ENSG00000231017	211298940-211299189	-	3001
2	212052599	212052842	244	212052746	9.09099	5.60604	4.18865	ENSG00000178568:intron	ENSG00000236007	211778272-211779763	+	274448
2	215186754	215186978	225	215186875	5.6497	4.01836	1.29426	intergenic	ENSG00000144452	214931541-215138428	-	-48437
2	215918128	215918360	233	215918244	7.96683	4.92425	3.22527	intergenic	ENSG00000226276	215939307-215941719	-	23475
2	215940855	215941071	217	215940880	4.36824	3.47775	0.59373	ENSG00000226276:intron	ENSG00000226276	215939307-215941719	-	756
2	215918128	215918360	233	215918244	7.96683	4.92425	3.22527	intergenic	ENSG00000226276	215939307-215941719	-	23475
2	215940855	215941071	217	215940880	4.36824	3.47775	0.59373	ENSG00000226276:intron	ENSG00000226276	215939307-215941719	-	756
2	218124301	218124588	288	218124400	4.84324	3.65398	0.77787	ENSG00000180871:Promoter	ENSG00000180871	218125288-218137253	+	-844
2	218623018	218623233	216	218623116	4.30996	3.44006	0.59373	ENSG00000115556:intron	ENSG00000273466	218633255-218634014	-	10889
2	219435606	219435923	318	219435636	4.93185	3.44723	0.83372	ENSG00000072195:intron	ENSG00000072195	219434845-219498287	+	919
2	224726139	224726546	408	224726472	4.56295	3.60388	0.59373	intergenic	ENSG00000283428	224703763-224716365	-	-9977
2	230134476	230134802	327	230134635	7.17425	4.80518	2.52654	ENSG00000225963:intron	ENSG00000283451	230138600-230138783	+	-3961
2	238266415	238266662	248	238266577	5.39424	4.00432	1.09512	ENSG00000132326:intron	ENSG00000283635	238289126-238300185	+	-22588
20	1163145	1163417	273	1163235	9.85502	5.24413	4.88402	ENSG00000125818:intron	ENSG00000215388	1160583-1161697	+	2697
20	1652816	1653152	337	1652861	4.56295	3.60388	0.59373	ENSG00000089012:intron	ENSG00000089012	1629151-1657779	-	4795
20	3444820	3445106	287	3444923	7.17425	4.80518	2.52654	intergenic	ENSG00000225116	3465176-3465444	-	20481
20	4748292	4748608	317	4748511	4.919	3.70193	0.82412	intergenic	ENSG00000180259	4731281-4740668	-	-7781
20	9122420	9122631	212	9122476	3.03786	2.80302	0.15111	ENSG00000101333:intron	ENSG00000101333	9068762-9481242	+	53763
20	9193443	9193663	221	9193554	5.78764	4.10443	1.39623	ENSG00000101333:intron	ENSG00000101333	9068762-9481242	+	124790
20	11962042	11962339	298	11962275	4.84177	3.65305	0.77787	intergenic	ENSG00000236526	11909403-11918677	-	-43513
20	13383095	13383310	216	13383163	4.18316	3.35817	0.52478	intergenic	ENSG00000236811	13392676-13393674	+	-9474
20	19090226	19090466	241	19090295	5.39424	4.00432	1.09512	intergenic	ENSG00000274863	19000708-19056796	-	-33549
20	19090226	19090466	241	19090295	5.39424	4.00432	1.09512	intergenic	ENSG00000274863	19000708-19056796	-	-33549
20	20431115	20431323	209	20431249	4.84177	3.65305	0.77787	ENSG00000188559:intron	ENSG00000173404	20368120-20370949	+	63098
20	20500175	20500441	267	20500247	6.66594	4.48944	2.09177	ENSG00000188559:intron	ENSG00000173404	20368120-20370949	+	132187
20	22533032	22533247	216	22533148	4.55072	3.59595	0.59373	intergenic	ENSG00000259974	22547670-22578642	-	45503

20	22569858	22570182	325	22570094	4.56295	3.60388	0.59373	ENSG00000283932:intron;ENSG00000259974:intron	ENSG00000259974	22547670-22578642	-	8622
20	22605215	22605545	331	22605513	3.61899	3.09983	0.30123	ENSG00000237396:intron	ENSG00000237396	22587521-22607517	-	2137
20	23132846	23133135	290	23133033	6.26578	4.40475	1.75945	ENSG00000233746:Promoter	ENSG00000233746	23125067-23132621	-	-369
20	23138582	23138948	367	23138617	3.77589	3.20345	0.30123	intergenic	ENSG00000233746	23125067-23132621	-	-6143
20	23132846	23133135	290	23133033	6.26578	4.40475	1.75945	ENSG00000233746:Promoter	ENSG00000233746	23125067-23132621	-	-369
20	23138582	23138948	367	23138617	3.77589	3.20345	0.30123	intergenic	ENSG00000233746	23125067-23132621	-	-6143
20	24021733	24021949	217	24021817	4.72575	3.5798	0.71873	intergenic	ENSG00000167390	23989291-23990454	+	32549
20	24842903	24843319	417	24843028	4.813	3.37843	0.77787	intergenic	ENSG00000274173	24931839-24932983	+	-88728
20	25310361	25310630	270	25310473	19.04992	8.08557	13.45281	ENSG00000100997:intron	ENSG00000276952	25284914-25285588	-	-24907
20	28531199	28531628	430	28531237	3.69312	2.14359	0.30123	intergenic	ENSG00000282826	28580708-28602664	-	71251
20	28834222	28834614	393	28834444	6.51641	4.07877	1.96373	intergenic	ENSG00000282826	28580708-28602664	-	-231753
20	28531199	28531628	430	28531237	3.69312	2.14359	0.30123	intergenic	ENSG00000282826	28580708-28602664	-	71251
20	28834222	28834614	393	28834444	6.51641	4.07877	1.96373	intergenic	ENSG00000282826	28580708-28602664	-	-231753
20	28531199	28531628	430	28531237	3.69312	2.14359	0.30123	intergenic	ENSG00000282826	28580708-28602664	-	71251
20	28834222	28834614	393	28834444	6.51641	4.07877	1.96373	intergenic	ENSG00000282826	28580708-28602664	-	-231753
20	28531199	28531628	430	28531237	3.69312	2.14359	0.30123	intergenic	ENSG00000282826	28580708-28602664	-	71251
20	28834222	28834614	393	28834444	6.51641	4.07877	1.96373	intergenic	ENSG00000282826	28580708-28602664	-	-231753
20	31836253	31836491	239	31836407	11.41258	5.13981	6.30043	intergenic	ENSG00000179772	31844300-31845619	-	9247
20	33778660	33778872	213	33778751	4.42449	3.05106	0.59373	ENSG00000131061:intron	ENSG00000230837	33752166-33752543	-	-26222
20	33911589	33911846	258	33911672	3.64836	3.01523	0.30123	intergenic	ENSG00000214200	33912322-33913336	-	1619
20	37135034	37135253	220	37135055	3.34993	2.92244	0.30123	ENSG00000101353:intron	ENSG00000236159	37165773-37166312	-	31169
20	38678834	38679059	226	38678980	4.91514	3.56573	0.82412	intergenic	ENSG00000277965	38711820-38712103	-	33157
20	38874723	38874982	260	38874956	4.94377	3.58317	0.84363	ENSG00000101445:intron	ENSG00000240474	38872769-38873074	+	2083
20	39071526	39071762	237	39071552	4.00982	3.24654	0.45978	intergenic	ENSG00000223864	38977710-38978422	-	-93221
20	39169999	39170207	209	39170038	3.77589	3.20345	0.30123	intergenic	ENSG00000230324	39213776-39224748	-	54645
20	39213309	39213543	235	39213514	5.25679	3.77493	1.07467	intergenic	ENSG00000230324	39213776-39224748	-	11322
20	43751260	43751573	314	43751433	16.24727	7.1237	10.77556	intergenic	ENSG00000124196	43726163-43726998	-	-24418
20	44001726	44001986	261	44001880	9.82127	4.56208	4.86215	ENSG00000124191:intron	ENSG00000241229	44007247-44007537	+	-5391
20	44646893	44647195	303	44647018	7.93683	4.3917	3.19908	ENSG00000196839:intron	ENSG00000196839	44619521-44652233	-	5189
20	44926172	44926391	220	44926309	5.22474	3.89619	1.05478	ENSG00000101104:intron	ENSG00000101104	44910061-44959035	+	16220
20	47825665	47825891	227	47825804	3.34993	2.92244	0.30123	intergenic	ENSG00000238950	47826968-47827044	-	1266
20	47828237	47828558	322	47828391	9.09099	5.60604	4.18865	ENSG00000238950:Promoter	ENSG00000238950	47826968-47827044	-	-1353
20	48562201	48562458	258	48562243	4.41276	3.50657	0.59373	intergenic	ENSG00000238452	48494233-48494309	-	-68020
20	48982553	48982789	237	48982670	6.24595	4.23134	1.75945	ENSG00000124198:intron	ENSG00000230758	49038356-49038602	-	55931
20	50338669	50339027	359	50338807	4.72575	3.5798	0.71873	intergenic	ENSG00000233077	50310710-50321342	-	-17505
20	51400464	51400701	238	51400575	7.40928	4.11883	2.71399	ENSG00000101096:intron	ENSG00000266761	51452904-51452977	-	52395
20	51585843	51586250	408	51586107	3.77589	3.20345	0.30123	intergenic	ENSG00000101096	51386956-51562831	-	-23215
20	52172600	52172827	228	52172686	4.69815	3.31233	0.69764	ENSG00000020256:intron	ENSG00000020256	52051662-52204308	-	31595
20	52434256	52434704	449	52434587	5.39424	4.00432	1.09512	ENSG00000234948:intron	ENSG00000225785	52451463-52455619	-	21139
20	53259685	53259920	236	53259794	4.31475	3.20475	0.59373	ENSG00000271774:intron;ENSG00000182463:intron	ENSG00000227705	53196228-53208705	-	-51097
20	54004362	54004999	638	54004568	7.3143	4.5382	2.6406	ENSG00000064787:intron	ENSG00000265595	54068407-54068485	-	63805
20	54030634	54030853	220	54030704	4.07388	3.28775	0.47041	ENSG00000064787:intron	ENSG00000265595	54068407-54068485	-	37742
20	54180000	54180299	300	54180161	8.8685	5.46977	4.02102	intergenic	ENSG00000019186	54153448-54173973	-	-6176
20	58907846	58908132	287	58907974	4.16942	3.34931	0.52478	ENSG00000087460:intron	ENSG00000225806	58863527-58888809	-	-19179
20	59723977	59724214	238	59724151	4.383	3.48731	0.59373	ENSG00000087495:intron	ENSG00000238194	59626463-59628289	-	-95806
20	60403458	60403717	260	60403593	7.3143	4.5382	2.6406	ENSG00000228340:intron	ENSG00000229690	60414395-60414872	-	11285
20	61045952	61046222	271	61046156	5.56876	3.96799	1.238	intergenic	ENSG00000229755	61077223-61077816	-	31729
20	62249734	62249963	230	62249924	4.1849	3.24157	0.52478	ENSG00000130703:intron	ENSG00000130703	62231921-62296213	+	17927
20	63077516	63077827	312	63077597	5.3628	3.84033	1.09512	ENSG00000272259:intron	ENSG00000274915	63102141-63102259	+	-24470

21	9112234	9112474	241	9112434	6.45739	2.89139	1.93187	ENSG00000188681:intron	ENSG00000188681	9068360-9129752	-	17398
21	9116665	9116879	215	9116847	4.29236	2.59088	0.59373	ENSG00000188681:intron	ENSG00000188681	9068360-9129752	-	12980
21	9147849	9148063	215	9147900	3.80292	2.3931	0.32309	intergenic	ENSG00000188681	9068360-9129752	-	-18203
21	9166340	9166556	217	9166485	4.03275	2.33536	0.47041	intergenic	ENSG00000188681	9068360-9129752	-	-36695
21	9175332	9175581	250	9175419	5.59646	3.05845	1.26027	intergenic	ENSG00000188681	9068360-9129752	-	-45704
21	9195556	9195848	293	9195692	3.55221	2.34329	0.30123	intergenic	ENSG00000188681	9068360-9129752	-	-65949
21	9112234	9112474	241	9112434	6.45739	2.89139	1.93187	ENSG00000188681:intron	ENSG00000188681	9068360-9129752	-	17398
21	9116665	9116879	215	9116847	4.29236	2.59088	0.59373	ENSG00000188681:intron	ENSG00000188681	9068360-9129752	-	12980
21	9147849	9148063	215	9147900	3.80292	2.3931	0.32309	intergenic	ENSG00000188681	9068360-9129752	-	-18203
21	9166340	9166556	217	9166485	4.03275	2.33536	0.47041	intergenic	ENSG00000188681	9068360-9129752	-	-36695
21	9175332	9175581	250	9175419	5.59646	3.05845	1.26027	intergenic	ENSG00000188681	9068360-9129752	-	-45704
21	9195556	9195848	293	9195692	3.55221	2.34329	0.30123	intergenic	ENSG00000188681	9068360-9129752	-	-65949
21	10457837	10458134	298	10457967	8.50744	3.58385	3.68567	ENSG00000187172:intron	ENSG00000273840	10482737-10605716	+	-24752
21	10460439	10460661	223	10460590	4.94033	2.78888	0.8404	ENSG00000187172:intron	ENSG00000273840	10482737-10605716	+	-22187
21	10627502	10627731	230	10627677	3.34993	2.92244	0.30123	intergenic	ENSG00000276556	10640027-10640338	-	12722
21	10631677	10632058	382	10631909	3.93344	3.19748	0.41301	intergenic	ENSG00000276556	10640027-10640338	-	8471
21	17560866	17561141	276	17561040	4.56295	3.60388	0.59373	ENSG00000154639:intron	ENSG00000199962	17576797-17576906	+	-15794
21	17563539	17563889	351	17563793	4.56295	3.60388	0.59373	ENSG00000154639:intron	ENSG00000199962	17576797-17576906	+	-13083
21	17662964	17663280	317	17663246	4.56295	3.60388	0.59373	intergenic	ENSG00000226956	17659275-17660384	-	-2737
21	17784719	17785045	327	17784804	7.21064	4.47768	2.55784	ENSG00000240770:intron	ENSG00000240770	17763314-17792523	-	7641
21	19148779	19149022	244	19148854	3.02441	2.62206	0.15111	intergenic	ENSG00000229289	19130522-19134423	+	18378
21	23524238	23524567	330	23524372	3.77589	3.20345	0.30123	intergenic	ENSG00000227716	23477640-23490724	+	46762
21	24126747	24127024	278	24126959	3.77589	3.20345	0.30123	intergenic	ENSG00000224018	24155169-24188342	-	61457
21	24151478	24151741	264	24151637	6.26578	4.40475	1.75945	intergenic	ENSG00000224018	24155169-24188342	-	36733
21	24246291	24246542	252	24246406	3.77589	3.20345	0.30123	intergenic	ENSG00000224018	24155169-24188342	-	-58074
21	25457504	25457760	257	25457619	9.09099	5.60604	4.18865	intergenic	ENSG00000185433	25385819-25431701	-	-25930
21	26071969	26072300	332	26072129	5.39424	4.00432	1.09512	ENSG00000142192:intron	ENSG00000224541	26158090-26175824	+	-85956
21	26071969	26072300	332	26072129	5.39424	4.00432	1.09512	ENSG00000142192:intron	ENSG00000224541	26158090-26175824	+	-85956
21	28420292	28420543	252	28420374	4.56295	3.60388	0.59373	intergenic	ENSG00000226935	28539317-28540355	+	-118900
21	30753100	30753365	266	30753236	5.73591	4.07212	1.35894	intergenic	ENSG00000187005	30754829-30755428	-	2196
21	31554503	31554743	241	31554591	16.16702	6.23562	10.6981	ENSG00000156299:intron	ENSG00000237594	31559244-31560487	+	-4621
21	33079056	33079398	343	33079182	18.10627	6.1624	12.54102	intergenic	ENSG00000227757	32913648-33071104	-	-8122
21	33214297	33214647	351	33214450	6.34257	3.83987	1.8313	intergenic	ENSG00000159110	33229900-33265675	+	-15428
21	33215658	33215876	219	33215777	5.86439	3.26245	1.4602	intergenic	ENSG00000159110	33229900-33265675	+	-14133
21	33216086	33216327	242	33216128	4.42449	3.05106	0.59373	intergenic	ENSG00000159110	33229900-33265675	+	-13694
21	33214297	33214647	351	33214450	6.34257	3.83987	1.8313	intergenic	ENSG00000159110	33229900-33265675	+	-15428
21	33215658	33215876	219	33215777	5.86439	3.26245	1.4602	intergenic	ENSG00000159110	33229900-33265675	+	-14133
21	33216086	33216327	242	33216128	4.42449	3.05106	0.59373	intergenic	ENSG00000159110	33229900-33265675	+	-13694
21	33434788	33435029	242	33434969	6.21409	3.77143	1.75458	ENSG00000142188:intron;ENSG00000159128:intron	ENSG00000159128	33402895-33479348	+	32013
21	34649694	34649916	223	34649749	4.69815	3.31233	0.69764	intergenic	ENSG00000159212	34669388-34718227	+	-19583
21	34801680	34801906	227	34801691	3.02441	2.62206	0.15111	intergenic	ENSG00000234380	34745756-34784886	+	56036
21	34865963	34866303	341	34866093	24.00173	8.47995	18.23352	intergenic	ENSG00000234380	34745756-34784886	+	120376
21	34801680	34801906	227	34801691	3.02441	2.62206	0.15111	intergenic	ENSG00000234380	34745756-34784886	+	56036
21	34865963	34866303	341	34866093	24.00173	8.47995	18.23352	intergenic	ENSG00000234380	34745756-34784886	+	120376
21	35187608	35187848	241	35187796	5.05494	3.51889	0.92723	ENSG00000159216:intron	ENSG00000234703	35136637-35139222	+	51090
21	35935212	35935439	228	35935385	3.9293	2.97803	0.41132	ENSG00000159216:intron	ENSG00000234008	35887194-35887807	+	48131
21	36854211	36854416	206	36854331	4.27198	3.17938	0.58762	ENSG00000159267:intron	ENSG00000199806	36851910-36852028	+	2403
21	36870207	36870423	217	36870362	4.00012	3.12743	0.45978	ENSG00000159267:intron	ENSG00000199806	36851910-36852028	+	18404
21	36987135	36987348	214	36987327	3.99877	3.01858	0.45978	ENSG00000207416:Promoter:ENSG00000159267:intron	ENSG00000207416	36986738-36986851	-	-390

21	37336404	37336622	219	37336480	5.4508	3.61666	1.13858	intergenic	ENSG00000157540	37365572-37526358	+	-29059
21	38111651	38111868	218	38111672	5.18253	3.59359	1.02124	ENSG00000184029:intron;ENSG00000157542:intron	ENSG00000157542	37607375-38121345	-	9586
21	39515111	39515539	429	39515242	5.09049	3.41464	0.95198	ENSG00000285815:three_prime_UTR;ENSG00000285815:exon;ENSG00000185437:three_prime_UTR;ENSG00000185437:exon	ENSG00000235012	39525582-39529855	+	-10257
21	40016343	40016565	223	40016477	7.72935	3.47459	3.00722	ENSG00000171587:intron	ENSG00000183036	39867316-39929397	+	149137
21	40400404	40400636	233	40400568	5.01585	3.49609	0.89533	ENSG00000171587:intron	ENSG00000235123	40383082-40385358	+	17437
21	40434493	40435089	597	40434985	4.45315	3.28714	0.59373	ENSG00000171587:intron	ENSG00000235123	40383082-40385358	+	51708
21	40435366	40435686	321	40435649	3.73951	2.86795	0.30123	ENSG00000171587:intron	ENSG00000235123	40383082-40385358	+	52443
21	41622862	41623127	266	41622963	3.88067	2.94972	0.37576	intergenic	ENSG00000223400	41576134-41581319	-	-41675
21	43307066	43307370	305	43307192	23.88818	7.54375	18.1235	intergenic	ENSG00000237864	43322416-43332039	-	24821
21	44093843	44094092	250	44093861	4.3756	3.12867	0.59373	ENSG00000160218:intron	ENSG00000241945	44107289-44131181	+	-13322
21	46240859	46241240	382	46241017	4.91514	3.56573	0.82412	ENSG00000160294:exon;ENSG00000215424:exon	ENSG00000228137	46246889-46247682	+	-5840
21	46550570	46550795	226	46550745	5.40704	3.35205	1.10135	ENSG00000160305:intron	ENSG00000202239	46525617-46525722	+	25065
22	10715026	10715288	263	10715116	6.65428	3.74089	2.08267	intergenic	ENSG00000277248	10736170-10736283	-	21126
22	11325184	11325393	210	11325248	6.06008	4.11811	1.6243	intergenic	ENSG00000276871	11249808-11249959	-	-75329
22	11375875	11376184	310	11376042	4.84177	3.65305	0.77787	intergenic	ENSG00000276871	11249808-11249959	-	-126070
22	11453530	11453847	318	11453821	4.5011	3.09221	0.59373	intergenic	ENSG00000276871	11249808-11249959	-	-203729
22	11834199	11834451	253	11834336	6.51641	4.07877	1.96373	intergenic	ENSG00000276871	11249808-11249959	-	-584365
22	11902167	11902378	212	11902283	6.6448	3.41016	2.07403	intergenic	ENSG00000276871	11249808-11249959	-	-652313
22	11325184	11325393	210	11325248	6.06008	4.11811	1.6243	intergenic	ENSG00000276871	11249808-11249959	-	-75329
22	11375875	11376184	310	11376042	4.84177	3.65305	0.77787	intergenic	ENSG00000276871	11249808-11249959	-	-126070
22	11453530	11453847	318	11453821	4.5011	3.09221	0.59373	intergenic	ENSG00000276871	11249808-11249959	-	-203729
22	11834199	11834451	253	11834336	6.51641	4.07877	1.96373	intergenic	ENSG00000276871	11249808-11249959	-	-584365
22	11902167	11902378	212	11902283	6.6448	3.41016	2.07403	intergenic	ENSG00000276871	11249808-11249959	-	-652313
22	11951307	11951557	251	11951490	4.31475	3.20475	0.59373	intergenic	ENSG00000283023	12602465-12626642	+	-651033
22	12075543	12075809	267	12075618	4.81478	2.9657	0.77787	intergenic	ENSG00000283023	12602465-12626642	+	-526789
22	12115821	12116123	303	12116028	5.93228	3.62292	1.51685	intergenic	ENSG00000283023	12602465-12626642	+	-486493
22	12410354	12410561	208	12410477	6.51641	4.07877	1.96373	intergenic	ENSG00000283023	12602465-12626642	+	-192008
22	12589418	12589740	323	12589559	8.28323	5.11428	3.47693	intergenic	ENSG00000283023	12602465-12626642	+	-12886
22	12639498	12639757	260	12639568	5.75051	3.78743	1.36222	intergenic	ENSG00000283023	12602465-12626642	+	37162
22	12641096	12641341	246	12641255	4.91514	3.56573	0.82412	intergenic	ENSG00000283023	12602465-12626642	+	38753
22	11951307	11951557	251	11951490	4.31475	3.20475	0.59373	intergenic	ENSG00000283023	12602465-12626642	+	-651033
22	12075543	12075809	267	12075618	4.81478	2.9657	0.77787	intergenic	ENSG00000283023	12602465-12626642	+	-526789
22	12115821	12116123	303	12116028	5.93228	3.62292	1.51685	intergenic	ENSG00000283023	12602465-12626642	+	-486493
22	12410354	12410561	208	12410477	6.51641	4.07877	1.96373	intergenic	ENSG00000283023	12602465-12626642	+	-192008
22	12589418	12589740	323	12589559	8.28323	5.11428	3.47693	intergenic	ENSG00000283023	12602465-12626642	+	-12886
22	12639498	12639757	260	12639568	5.75051	3.78743	1.36222	intergenic	ENSG00000283023	12602465-12626642	+	37162
22	12641096	12641341	246	12641255	4.91514	3.56573	0.82412	intergenic	ENSG00000283023	12602465-12626642	+	38753
22	11951307	11951557	251	11951490	4.31475	3.20475	0.59373	intergenic	ENSG00000283023	12602465-12626642	+	-651033
22	12075543	12075809	267	12075618	4.81478	2.9657	0.77787	intergenic	ENSG00000283023	12602465-12626642	+	-526789
22	12115821	12116123	303	12116028	5.93228	3.62292	1.51685	intergenic	ENSG00000283023	12602465-12626642	+	-486493
22	12410354	12410561	208	12410477	6.51641	4.07877	1.96373	intergenic	ENSG00000283023	12602465-12626642	+	-192008
22	12589418	12589740	323	12589559	8.28323	5.11428	3.47693	intergenic	ENSG00000283023	12602465-12626642	+	-12886
22	12639498	12639757	260	12639568	5.75051	3.78743	1.36222	intergenic	ENSG00000283023	12602465-12626642	+	37162
22	12641096	12641341	246	12641255	4.91514	3.56573	0.82412	intergenic	ENSG00000283023	12602465-12626642	+	38753
22	11951307	11951557	251	11951490	4.31475	3.20475	0.59373	intergenic	ENSG00000283023	12602465-12626642	+	-651033

22	12075543	12075809	267	12075618	4.81478	2.9657	0.77787	intergenic	ENSG00000283023	12602465-12626642	+	-526789
22	12115821	12116123	303	12116028	5.93228	3.62292	1.51685	intergenic	ENSG00000283023	12602465-12626642	+	-486493
22	12410354	12410561	208	12410477	6.51641	4.07877	1.96373	intergenic	ENSG00000283023	12602465-12626642	+	-192008
22	12589418	12589740	323	12589559	8.28323	5.11428	3.47693	intergenic	ENSG00000283023	12602465-12626642	+	-12886
22	12639498	12639757	260	12639568	5.75051	3.78743	1.36222	intergenic	ENSG00000283023	12602465-12626642	+	37162
22	12641096	12641341	246	12641255	4.91514	3.56573	0.82412	intergenic	ENSG00000283023	12602465-12626642	+	38753
22	11951307	11951557	251	11951490	4.31475	3.20475	0.59373	intergenic	ENSG00000283023	12602465-12626642	+	-651033
22	12075543	12075809	267	12075618	4.81478	2.9657	0.77787	intergenic	ENSG00000283023	12602465-12626642	+	-526789
22	12115821	12116123	303	12116028	5.93228	3.62292	1.51685	intergenic	ENSG00000283023	12602465-12626642	+	-486493
22	12410354	12410561	208	12410477	6.51641	4.07877	1.96373	intergenic	ENSG00000283023	12602465-12626642	+	-192008
22	12589418	12589740	323	12589559	8.28323	5.11428	3.47693	intergenic	ENSG00000283023	12602465-12626642	+	-12886
22	12639498	12639757	260	12639568	5.75051	3.78743	1.36222	intergenic	ENSG00000283023	12602465-12626642	+	37162
22	12641096	12641341	246	12641255	4.91514	3.56573	0.82412	intergenic	ENSG00000283023	12602465-12626642	+	38753
22	11951307	11951557	251	11951490	4.31475	3.20475	0.59373	intergenic	ENSG00000283023	12602465-12626642	+	-651033
22	12075543	12075809	267	12075618	4.81478	2.9657	0.77787	intergenic	ENSG00000283023	12602465-12626642	+	-526789
22	12115821	12116123	303	12116028	5.93228	3.62292	1.51685	intergenic	ENSG00000283023	12602465-12626642	+	-486493
22	12410354	12410561	208	12410477	6.51641	4.07877	1.96373	intergenic	ENSG00000283023	12602465-12626642	+	-192008
22	12589418	12589740	323	12589559	8.28323	5.11428	3.47693	intergenic	ENSG00000283023	12602465-12626642	+	-12886
22	12639498	12639757	260	12639568	5.75051	3.78743	1.36222	intergenic	ENSG00000283023	12602465-12626642	+	37162
22	12641096	12641341	246	12641255	4.91514	3.56573	0.82412	intergenic	ENSG00000283023	12602465-12626642	+	38753
22	11951307	11951557	251	11951490	4.31475	3.20475	0.59373	intergenic	ENSG00000283023	12602465-12626642	+	-651033
22	12075543	12075809	267	12075618	4.81478	2.9657	0.77787	intergenic	ENSG00000283023	12602465-12626642	+	-526789
22	12115821	12116123	303	12116028	5.93228	3.62292	1.51685	intergenic	ENSG00000283023	12602465-12626642	+	-486493
22	12410354	12410561	208	12410477	6.51641	4.07877	1.96373	intergenic	ENSG00000283023	12602465-12626642	+	-192008
22	12589418	12589740	323	12589559	8.28323	5.11428	3.47693	intergenic	ENSG00000283023	12602465-12626642	+	-12886
22	12639498	12639757	260	12639568	5.75051	3.78743	1.36222	intergenic	ENSG00000283023	12602465-12626642	+	37162
22	12641096	12641341	246	12641255	4.91514	3.56573	0.82412	intergenic	ENSG00000283023	12602465-12626642	+	38753
22	11951307	11951557	251	11951490	4.31475	3.20475	0.59373	intergenic	ENSG00000283023	12602465-12626642	+	-651033
22	12075543	12075809	267	12075618	4.81478	2.9657	0.77787	intergenic	ENSG00000283023	12602465-12626642	+	-526789
22	12115821	12116123	303	12116028	5.93228	3.62292	1.51685	intergenic	ENSG00000283023	12602465-12626642	+	-486493
22	12410354	12410561	208	12410477	6.51641	4.07877	1.96373	intergenic	ENSG00000283023	12602465-12626642	+	-192008
22	12589418	12589740	323	12589559	8.28323	5.11428	3.47693	intergenic	ENSG00000283023	12602465-12626642	+	-12886
22	12639498	12639757	260	12639568	5.75051	3.78743	1.36222	intergenic	ENSG00000283023	12602465-12626642	+	37162
22	12641096	12641341	246	12641255	4.91514	3.56573	0.82412	intergenic	ENSG00000283023	12602465-12626642	+	38753
22	11951307	11951557	251	11951490	4.31475	3.20475	0.59373	intergenic	ENSG00000283023	12602465-12626642	+	-651033
22	12075543	12075809	267	12075618	4.81478	2.9657	0.77787	intergenic	ENSG00000283023	12602465-12626642	+	-526789
22	12115821	12116123	303	12116028	5.93228	3.62292	1.51685	intergenic	ENSG00000283023	12602465-12626642	+	-486493
22	12410354	12410561	208	12410477	6.51641	4.07877	1.96373	intergenic	ENSG00000283023	12602465-12626642	+	-192008
22	12589418	12589740	323	12589559	8.28323	5.11428	3.47693	intergenic	ENSG00000283023	12602465-12626642	+	-12886
22	12639498	12639757	260	12639568	5.75051	3.78743	1.36222	intergenic	ENSG00000283023	12602465-12626642	+	37162
22	12641096	12641341	246	12641255	4.91514	3.56573	0.82412	intergenic	ENSG00000283023	12602465-12626642	+	38753
22	11951307	11951557	251	11951490	4.31475	3.20475	0.59373	intergenic	ENSG00000283023	12602465-12626642	+	-651033
22	12075543	12075809	267	12075618	4.81478	2.9657	0.77787	intergenic	ENSG00000283023	12602465-12626642	+	-526789
22	12115821	12116123	303	12116028	5.93228	3.62292	1.51685	intergenic	ENSG00000283023	12602465-12626642	+	-486493
22	12410354	12410561	208	12410477	6.51641	4.07877	1.96373	intergenic	ENSG00000283023	12602465-12626642	+	-192008
22	12589418	12589740	323	12589559	8.28323	5.11428	3.47693	intergenic	ENSG00000283023	12602465-12626642	+	-12886
22	12639498	12639757	260	12639568	5.75051	3.78743	1.36222	intergenic	ENSG00000283023	12602465-12626642	+	37162
22	12641096	12641341	246	12641255	4.91514	3.56573	0.82412	intergenic	ENSG00000283023	12602465-12626642	+	38753
22	11951307	11951557	251	11951490	4.31475	3.20475	0.59373	intergenic	ENSG00000283023	12602465-12626642	+	-651033
22	12075543	12075809	267	12075618	4.81478	2.9657	0.77787	intergenic	ENSG00000283023	12602465-12626642	+	-526789
22	12115821	12116123	303	12116028	5.93228	3.62292	1.51685	intergenic	ENSG00000283023	12602465-12626642	+	-486493

22	12410354	12410561	208	12410477	6.51641	4.07877	1.96373	intergenic	ENSG00000283023	12602465-12626642	+	-192008
22	12589418	12589740	323	12589559	8.28323	5.11428	3.47693	intergenic	ENSG00000283023	12602465-12626642	+	-12886
22	12639498	12639757	260	12639568	5.75051	3.78743	1.36222	intergenic	ENSG00000283023	12602465-12626642	+	37162
22	12641096	12641341	246	12641255	4.91514	3.56573	0.82412	intergenic	ENSG00000283023	12602465-12626642	+	38753
22	11951307	11951557	251	11951490	4.31475	3.20475	0.59373	intergenic	ENSG00000283023	12602465-12626642	+	-651033
22	12075543	12075809	267	12075618	4.81478	2.9657	0.77787	intergenic	ENSG00000283023	12602465-12626642	+	-526789
22	12115821	12116123	303	12116028	5.93228	3.62292	1.51685	intergenic	ENSG00000283023	12602465-12626642	+	-486493
22	12410354	12410561	208	12410477	6.51641	4.07877	1.96373	intergenic	ENSG00000283023	12602465-12626642	+	-192008
22	12589418	12589740	323	12589559	8.28323	5.11428	3.47693	intergenic	ENSG00000283023	12602465-12626642	+	-12886
22	12639498	12639757	260	12639568	5.75051	3.78743	1.36222	intergenic	ENSG00000283023	12602465-12626642	+	37162
22	12641096	12641341	246	12641255	4.91514	3.56573	0.82412	intergenic	ENSG00000283023	12602465-12626642	+	38753
22	11951307	11951557	251	11951490	4.31475	3.20475	0.59373	intergenic	ENSG00000283023	12602465-12626642	+	-651033
22	12075543	12075809	267	12075618	4.81478	2.9657	0.77787	intergenic	ENSG00000283023	12602465-12626642	+	-526789
22	12115821	12116123	303	12116028	5.93228	3.62292	1.51685	intergenic	ENSG00000283023	12602465-12626642	+	-486493
22	12410354	12410561	208	12410477	6.51641	4.07877	1.96373	intergenic	ENSG00000283023	12602465-12626642	+	-192008
22	12589418	12589740	323	12589559	8.28323	5.11428	3.47693	intergenic	ENSG00000283023	12602465-12626642	+	-12886
22	12639498	12639757	260	12639568	5.75051	3.78743	1.36222	intergenic	ENSG00000283023	12602465-12626642	+	37162
22	12641096	12641341	246	12641255	4.91514	3.56573	0.82412	intergenic	ENSG00000283023	12602465-12626642	+	38753
22	18056895	18057254	360	18057028	8.98232	4.95279	4.10924	intergenic	ENSG00000215193	18077919-18131138	+	-20845
22	19019828	19020208	381	19020161	4.56295	3.60388	0.59373	ENSG00000270393:Promoter:ENSG00000273032:intron	ENSG00000270393	19018042-19018916	-	-1101
22	19606000	19606282	283	19606172	4.42421	3.39052	0.59373	intergenic	ENSG00000230485	19630749-19633574	-	27433
22	19862351	19862630	280	19862406	3.02441	2.62206	0.15111	intergenic	ENSG00000185838	19783223-19854939	-	-7551
22	22451457	22451703	247	22451642	5.39424	4.00432	1.09512	intergenic	ENSG00000253631	22450201-22450652	+	1378
22	24352205	24352469	265	24352372	4.85824	3.66347	0.79046	ENSG00000100014:intron;ENSG00000258555:intron	ENSG00000128271	24417878-24442360	+	-65541
22	24434170	24434431	262	24434312	15.52885	6.53715	10.089	ENSG00000178803:intron;ENSG00000128271:intron;ENSG00000258555:intron	ENSG00000128271	24417878-24442360	+	16422
22	24352205	24352469	265	24352372	4.85824	3.66347	0.79046	ENSG00000100014:intron;ENSG00000258555:intron	ENSG00000128271	24417878-24442360	+	-65541
22	24434170	24434431	262	24434312	15.52885	6.53715	10.089	ENSG00000178803:intron;ENSG00000128271:intron;ENSG00000258555:intron	ENSG00000128271	24417878-24442360	+	16422
22	24576949	24577496	548	24577314	7.17425	4.80518	2.52654	ENSG00000100028:intron	ENSG00000100031	24583749-24629005	+	-6527
22	25793481	25793764	284	25793622	5.39424	4.00432	1.09512	ENSG00000133454:intron	ENSG00000278960	25756317-25756669	-	-36953
22	26081384	26081603	220	26081568	3.90845	3.18146	0.39391	intergenic	ENSG00000232050	26006388-26006532	-	-74961
22	26105588	26105820	233	26105741	4.84177	3.65305	0.77787	intergenic	ENSG00000236464	26161833-26164303	+	-56129
22	26610224	26610686	463	26610453	36.74275	12.63118	30.61533	ENSG00000100122:intron	ENSG00000100122	26599277-26618088	-	7633
22	27738808	27739042	235	27738964	4.82831	3.64454	0.77787	intergenic	ENSG00000226169	27716479-27721677	+	22445
22	29517862	29518093	232	29517944	3.30549	2.89321	0.30123	ENSG00000234208:intron;ENSG00000100296:intron	ENSG00000234208	29513888-29535390	-	17413
22	31167812	31168262	451	31168194	5.39424	4.00432	1.09512	ENSG00000138942:intron	ENSG00000138942	31160182-31207019	+	7854
22	31290726	31290946	221	31290851	7.69969	3.86859	2.97966	ENSG00000228839:Promoter:ENSG00000100100:intron	ENSG00000228839	31292498-31338021	+	-1662
22	32812350	32812582	233	32812480	6.07692	4.28581	1.63695	ENSG00000185666:intron;ENSG00000100234:intron	ENSG00000100234	32801700-32863043	+	10765
22	32838332	32838541	210	32838467	3.6499	2.91341	0.30123	ENSG00000185666:intron;ENSG00000100234:intron	ENSG00000100234	32801700-32863043	+	36736
22	33835794	33836017	224	33835958	3.56795	3.06614	0.30123	ENSG00000133424:intron	ENSG00000273082	33922421-33922766	+	-86516
22	35926225	35926502	278	35926468	3.98411	3.23002	0.45207	ENSG00000100320:intron	ENSG00000237406	35897269-35898404	-	-27959

22	36148604	36148873	270	36148743	7.84387	5.03722	3.11593	ENSG00000128284:intron	ENSG00000279805	36137429-36139350	−	-9388
22	36331464	36331819	356	36331640	15.66006	6.01366	10.21136	ENSG00000100345:intron	ENSG00000278420	36286846-36286907	−	-44734
22	37838272	37838647	376	37838623	3.89605	3.1735	0.38387	ENSG00000100124:intron	ENSG00000284197	37844271-37844371	−	5912
22	37933837	37934060	224	37934002	4.15018	2.90511	0.51719	ENSG00000100139:intron	ENSG00000278948	37943049-37944898	+	-9101
22	38133944	38134221	278	38134142	4.74019	3.58891	0.73063	ENSG00000184381:intron;ENSG00000279080:exon	ENSG00000279080	38130215-38150612	+	3867
22	38153250	38153457	208	38153321	4.53041	3.45699	0.59373	ENSG00000184381:intron	ENSG00000279080	38130215-38150612	+	23138
22	38481693	38481993	301	38481847	3.70522	3.05147	0.30123	ENSG00000100196:intron	ENSG00000100196	38468061-38483447	+	13781
22	38982198	38982482	285	38982353	9.99867	5.32258	5.01318	ENSG00000128383:intron;ENSG00000179750:Promoter	ENSG00000179750	38982346-38992804	+	-6
22	38985018	38985438	421	38985180	33.9636	10.66051	27.9178	ENSG00000128383:intron;ENSG00000179750:intron	ENSG00000179750	38982346-38992804	+	2881
22	38982198	38982482	285	38982353	9.99867	5.32258	5.01318	ENSG00000128383:intron;ENSG00000179750:Promoter	ENSG00000179750	38982346-38992804	+	-6
22	38985018	38985438	421	38985180	33.9636	10.66051	27.9178	ENSG00000128383:intron;ENSG00000179750:intron	ENSG00000179750	38982346-38992804	+	2881
22	40177149	40177401	253	40177297	6.4824	4.21404	1.94623	ENSG00000100354:intron	ENSG00000228599	40106324-40107066	−	-70208
22	40224929	40225192	264	40224973	3.49438	2.91742	0.30123	ENSG00000100354:intron	ENSG00000228599	40106324-40107066	−	-117994
22	40177149	40177401	253	40177297	6.4824	4.21404	1.94623	ENSG00000100354:intron	ENSG00000228599	40106324-40107066	−	-70208
22	40224929	40225192	264	40224973	3.49438	2.91742	0.30123	ENSG00000100354:intron	ENSG00000228599	40106324-40107066	−	-117994
22	42193964	42194190	227	42194032	4.91514	3.56573	0.82412	intergenic	ENSG00000226450	42149885-42155001	−	-39075
22	42511729	42512033	305	42512001	4.85005	3.52617	0.78361	ENSG00000189306:exon;ENSG00000189306:three_prime_UTR;ENSG00000172250:intron	ENSG00000189306	42509967-42519802	−	7921
22	42533376	42533625	250	42533510	7.14655	3.62494	2.51947	intergenic	ENSG00000189306	42509967-42519802	−	-13698
22	43103999	43104303	305	43104173	8.0161	4.5963	3.27031	intergenic	ENSG00000100290	43110747-43129712	+	-6596
22	43222243	43222502	260	43222312	3.3121	2.80227	0.30123	ENSG00000159307:intron	ENSG00000236272	43212673-43213661	+	9699
22	47387919	47388341	423	47388026	3.38496	2.66513	0.30123	intergenic	ENSG00000224715	47345568-47373541	+	42561
22	47593100	47593394	295	47593200	3.54284	3.04957	0.30123	intergenic	ENSG00000205634	47621042-47631569	−	38322
22	48378814	48379036	223	48378943	4.06198	3.28009	0.47041	intergenic	ENSG00000285855	48403461-48415571	+	-24536
22	48832648	48832880	233	48832753	8.13775	4.49748	3.3598	ENSG00000219438:intron	ENSG00000205632	48866769-48898386	+	-34005
22	50152529	50152774	246	50152667	6.51641	4.07877	1.96373	ENSG00000073146:intron	ENSG00000073150	50170730-50180294	+	-18079
3	9462173	9462409	237	9462245	4.1849	3.24157	0.52478	ENSG00000168137:intron	ENSG00000206573	9349688-9398579	−	-63711
3	13332226	13332457	232	13332378	5.13473	3.43925	0.98202	ENSG00000132182:exon	ENSG00000144711	12897219-13283281	−	-49060
3	14468563	14469024	462	14468770	3.20396	2.64557	0.26167	ENSG00000131389:intron	ENSG00000207163	14530981-14531088	−	62295
3	15805917	15806138	222	15806022	6.06008	4.11811	1.6243	ENSG00000206560:intron	ENSG00000206560	15667235-15859771	−	53744
3	15919476	15919884	409	15919744	4.07388	3.28775	0.47041	intergenic	ENSG00000224728	15878046-15879571	+	41633
3	16534703	16534932	230	16534722	3.60613	3.09134	0.30123	ENSG00000233570:Promoter	ENSG00000233570	16536319-16541373	+	-1502
3	18524923	18525183	261	18525024	4.56295	3.60388	0.59373	ENSG00000228956:Promoter	ENSG00000236570	18538672-18539899	+	-13619
3	18572609	18572972	364	18572798	3.03786	2.80302	0.15111	ENSG00000228956:intron	ENSG00000236570	18538672-18539899	+	34118
3	18665200	18665434	235	18665366	3.77589	3.20345	0.30123	ENSG00000228956:intron	ENSG00000236570	18538672-18539899	+	126644
3	18676521	18676790	270	18676616	5.39424	4.00432	1.09512	ENSG00000228956:intron	ENSG00000236570	18538672-18539899	+	137983
3	18524923	18525183	261	18525024	4.56295	3.60388	0.59373	ENSG00000228956:Promoter	ENSG00000236570	18538672-18539899	+	-13619
3	18572609	18572972	364	18572798	3.03786	2.80302	0.15111	ENSG00000228956:intron	ENSG00000236570	18538672-18539899	+	34118
3	18665200	18665434	235	18665366	3.77589	3.20345	0.30123	ENSG00000228956:intron	ENSG00000236570	18538672-18539899	+	126644
3	18676521	18676790	270	18676616	5.39424	4.00432	1.09512	ENSG00000228956:intron	ENSG00000236570	18538672-18539899	+	137983
3	20457954	20458214	261	20458045	5.07624	3.80169	0.93914	ENSG00000231304:intron	ENSG00000206807	20507643-20507756	−	49672
3	24548083	24548365	283	24548102	4.28127	3.42152	0.58762	ENSG00000228791:intron	ENSG00000233544	24555833-24556459	+	-7609
3	27616125	27616417	293	27616281	5.39424	4.00432	1.09512	intergenic	ENSG00000223968	27624201-27624456	−	8185
3	27616909	27617123	215	27616986	5.38044	3.99551	1.09512	intergenic	ENSG00000223968	27624201-27624456	−	7440

3	29005250	29005482	233	29005350	3.20396	2.64557	0.26167	ENSG00000144642:intron;ENSG00000283563:intron	ENSG00000215004	29087395-29088599	+	-82029
3	29686998	29687215	218	29687164	6.89723	4.46128	2.29209	ENSG00000144642:intron;ENSG00000283563:intron	ENSG00000203506	29526250-29642809	-	-44297
3	30353993	30354292	300	30354155	3.03786	2.80302	0.15111	intergenic	ENSG00000281710	30304320-30304536	-	-49606
3	30409881	30410177	297	30409954	4.56295	3.60388	0.59373	intergenic	ENSG00000281710	30304320-30304536	-	-105492
3	30606263	30606761	499	30606641	5.08979	3.09487	0.95129	ENSG00000163513:exon;ENSG00000163513:five_prime_UTR	ENSG00000163513	30606501-30694142	+	10
3	33791906	33792125	220	33792039	4.98096	3.7412	0.87315	intergenic	ENSG00000170248	33798351-33869707	+	-6336
3	36444954	36445244	291	36445081	7.82144	5.02342	3.09404	ENSG00000144681:intron	ENSG00000144681	36380343-36548007	+	64755
3	42012850	42013106	257	42013015	5.53737	3.80352	1.21582	ENSG00000182606:Promoter	ENSG00000182606	42013801-42225889	+	-823
3	43499050	43499262	213	43499143	4.07388	3.28775	0.47041	ENSG00000160746:intron	ENSG00000223916	43484971-43485880	-	-13275
3	43566409	43566709	301	43566531	5.39424	4.00432	1.09512	ENSG00000160746:intron	ENSG00000223916	43484971-43485880	-	-80678
3	43810874	43811295	422	43811117	7.17425	4.80518	2.52654	intergenic	ENSG00000235886	43778960-43779982	-	-31102
3	43814248	43814487	240	43814330	3.77589	3.20345	0.30123	intergenic	ENSG00000235886	43778960-43779982	-	-34385
3	44105003	44105271	269	44105078	11.66864	5.82681	6.52722	intergenic	ENSG00000207954	44114211-44114310	+	-9074
3	45302896	45303165	270	45303142	4.458	3.53587	0.59373	intergenic	ENSG00000202268	45255408-45255620	-	-47410
3	46109354	46109601	248	46109484	9.03881	5.3697	4.15943	intergenic	ENSG00000229515	46142357-46143677	-	34200
3	46386322	46386575	254	46386440	5.93037	4.03949	1.51498	ENSG00000223552:intron	ENSG00000160791	46370853-46376206	+	15595
3	46539130	46539393	264	46539223	8.134	4.66145	3.3598	ENSG00000163827:intron	ENSG00000268324	46557397-46559694	+	-18136
3	51355815	51356056	242	51356011	3.77589	3.20345	0.30123	ENSG00000088538:intron	ENSG00000145050	51385046-51389397	+	-29111
3	52313801	52314051	251	52313942	11.42179	5.90287	6.30811	intergenic	ENSG00000114841	52316318-52400491	+	-2392
3	54700534	54700795	262	54700723	4.1849	3.24157	0.52478	ENSG00000157445:intron	ENSG00000265992	54632121-54639857	-	-60807
3	56047216	56047639	424	56047408	6.26578	4.40475	1.75945	ENSG00000187672:intron	ENSG00000200379	56072103-56072407	+	-24676
3	59655385	59655596	212	59655386	3.34993	2.92244	0.30123	intergenic	ENSG00000277592	59584168-59584445	+	71322
3	59772592	59772834	243	59772674	3.40931	2.96154	0.30123	ENSG00000189283:intron	ENSG00000277592	59584168-59584445	+	188544
3	59832645	59832886	242	59832724	5.39424	4.00432	1.09512	ENSG00000189283:intron	ENSG00000277592	59584168-59584445	+	248597
3	72673119	72673323	205	72673162	6.07692	4.28581	1.63695	intergenic	ENSG00000222838	72691704-72691811	-	18590
3	72731857	72732084	228	72731988	5.39424	4.00432	1.09512	intergenic	ENSG00000222838	72691704-72691811	-	-40159
3	74531587	74531828	242	74531741	5.01585	3.49609	0.89533	intergenic	ENSG00000113805	74262567-74521140	-	-10567
3	74601743	74602083	341	74601833	4.56295	3.60388	0.59373	intergenic	ENSG00000113805	74262567-74521140	-	-80772
3	74647550	74647907	358	74647652	4.56295	3.60388	0.59373	intergenic	ENSG00000113805	74262567-74521140	-	-126588
3	74531587	74531828	242	74531741	5.01585	3.49609	0.89533	intergenic	ENSG00000113805	74262567-74521140	-	-10567
3	74601743	74602083	341	74601833	4.56295	3.60388	0.59373	intergenic	ENSG00000113805	74262567-74521140	-	-80772
3	74647550	74647907	358	74647652	4.56295	3.60388	0.59373	intergenic	ENSG00000113805	74262567-74521140	-	-126588
3	74531587	74531828	242	74531741	5.01585	3.49609	0.89533	intergenic	ENSG00000113805	74262567-74521140	-	-10567
3	74601743	74602083	341	74601833	4.56295	3.60388	0.59373	intergenic	ENSG00000113805	74262567-74521140	-	-80772
3	74647550	74647907	358	74647652	4.56295	3.60388	0.59373	intergenic	ENSG00000113805	74262567-74521140	-	-126588
3	75621872	75622130	259	75622024	10.24382	5.87681	5.23377	ENSG00000172971:intron	ENSG00000172971	75619505-75623849	-	1848
3	76327947	76328289	343	76328084	3.77589	3.20345	0.30123	ENSG00000185008:intron	ENSG00000240809	76434017-76435428	+	-105899
3	76393446	76393963	518	76393667	3.34993	2.92244	0.30123	ENSG00000185008:intron	ENSG00000240809	76434017-76435428	+	-40313
3	89553499	89553759	261	89553503	3.77589	3.20345	0.30123	intergenic	ENSG00000240951	89587885-89588570	-	34941
3	89555508	89555803	296	89555667	6.4824	4.21404	1.94623	intergenic	ENSG00000240951	89587885-89588570	-	32915
3	93470524	93470773	250	93470655	56.22915	3.80316	49.67563	intergenic	ENSG00000251727	93843763-93843862	+	-373115
3	93732500	93732705	206	93732643	5.39424	4.00432	1.09512	intergenic	ENSG00000251727	93843763-93843862	+	-111161
3	97490717	97491007	291	97490958	4.56295	3.60388	0.59373	ENSG00000080224:intron	ENSG00000251172	97481176-97481506	-	-9355
3	97578942	97579225	284	97579173	3.77589	3.20345	0.30123	ENSG00000080224:intron	ENSG00000251172	97481176-97481506	-	-97577
3	97490717	97491007	291	97490958	4.56295	3.60388	0.59373	ENSG00000080224:intron	ENSG00000251172	97481176-97481506	-	-9355
3	97578942	97579225	284	97579173	3.77589	3.20345	0.30123	ENSG00000080224:intron	ENSG00000251172	97481176-97481506	-	-97577
3	101134908	101135161	254	101134947	3.38583	2.94608	0.30123	intergenic	ENSG00000242573	101163093-101163986	+	-28059

3	101518527	101518749	223	101518704	4.93185	3.44723	0.83372	intergenic	ENSG00000175841	101521890-101522979	+	-3252
3	101568390	101568652	263	101568479	6.02088	4.25059	1.59152	intergenic	ENSG00000081154	101574094-101594437	+	-5573
3	101936606	101936917	312	101936744	12.25472	5.36328	7.06105	intergenic	ENSG00000214407	101940858-101997926	+	-4097
3	101942519	101942731	213	101942622	7.38831	4.58155	2.69775	ENSG00000214407:intron	ENSG00000214407	101940858-101997926	+	1766
3	102125345	102125659	315	102125655	3.34993	2.92244	0.30123	ENSG00000170044:intron	ENSG00000170044	102099243-102479841	+	26258
3	102183963	102184333	371	102184193	4.94982	3.72146	0.84841	ENSG00000170044:intron	ENSG00000170044	102099243-102479841	+	84904
3	104519167	104519403	237	104519310	6.25041	4.39506	1.75945	intergenic	ENSG00000243635	104502699-104503266	+	16585
3	106819745	106820078	334	106819876	4.56295	3.60388	0.59373	intergenic	ENSG00000239997	106848670-106849260	+	-28759
3	108100987	108101515	529	108101151	15.93958	7.45562	10.47535	intergenic	ENSG00000196776	108043093-108091862	-	-9388
3	111543726	111544010	285	111543875	4.56295	3.60388	0.59373	intergenic	ENSG00000240787	111570637-111571054	-	27186
3	112676874	112677128	255	112677001	11.85996	5.53143	6.70891	intergenic	ENSG00000242308	112696907-112697706	-	20705
3	119700593	119700918	326	119700768	8.17554	4.86236	3.38452	intergenic	ENSG00000183833	119703021-119767102	+	-2266
3	120282352	120282604	253	120282527	6.49439	4.38367	1.95029	ENSG00000175697:intron	ENSG00000175697	120164644-120285094	-	2616
3	121699939	121700161	223	121699969	5.94748	4.20452	1.52959	ENSG00000173230:intron	ENSG00000180353	121631398-121660927	-	-39122
3	121699939	121700161	223	121699969	5.94748	4.20452	1.52959	ENSG00000173230:intron	ENSG00000180353	121631398-121660927	-	-39122
3	122573339	122573553	215	122573494	5.31697	3.81203	1.09512	ENSG00000163840:three_prime_UTR;ENSG00000163840:exon	ENSG00000173200	122577601-122639047	+	-4155
3	122610067	122610367	301	122610198	9.99238	5.72768	5.00703	ENSG00000173200:intron	ENSG00000173200	122577601-122639047	+	32615
3	123371458	123371685	228	123371603	4.62651	3.51732	0.63659	ENSG00000173175:intron	ENSG00000173175	123282295-123449758	-	78187
3	125053484	125053808	325	125053532	4.31475	3.20475	0.59373	ENSG00000173706:intron	ENSG00000173706	124965709-125055958	-	2312
3	126252736	126252986	251	126252969	3.29424	2.88581	0.30123	intergenic	ENSG00000250934	126266795-126291279	+	-13934
3	126911292	126911769	478	126911623	32.49686	10.84686	26.49206	ENSG00000159685:intron	ENSG00000280053	126973064-126976426	+	-61534
3	130329116	130329378	263	130329298	6.5896	4.44231	2.02209	intergenic	ENSG00000172752	130345515-130484844	+	-16268
3	130388847	130389180	334	130389058	3.77589	3.20345	0.30123	ENSG00000172752:exon	ENSG00000172752	130345515-130484844	+	43498
3	130504740	130505039	300	130504802	4.98096	3.7412	0.87315	intergenic	ENSG00000206384	130560333-130678155	+	-55444
3	130603015	130603271	257	130603171	3.77589	3.20345	0.30123	ENSG00000206384:intron	ENSG00000206384	130560333-130678155	+	42809
3	130835573	130835834	262	130835728	4.47325	3.54574	0.59373	intergenic	ENSG00000232829	130827658-130828376	+	8045
3	130850150	130850394	245	130850282	9.78373	4.69287	4.83465	ENSG00000017260:Promoter	ENSG00000017260	130850594-131016712	+	-322
3	132283471	132283698	228	132283575	4.64049	3.52611	0.64891	ENSG00000196353:intron	ENSG00000196353	131533554-132285410	-	1826
3	133475598	133475905	308	133475799	10.69825	4.53118	5.64748	intergenic	ENSG00000214301	133490823-133491505	+	-15072
3	134620714	134620996	283	134620911	4.64049	3.52611	0.64891	ENSG00000174611:intron	ENSG00000154928	134597800-135260467	+	23054
3	135175157	135175362	206	135175324	4.51948	3.5757	0.59373	ENSG00000154928:intron;ENSG00000240086:intron	ENSG00000240086	135138468-135439888	-	264629
3	135185209	135185479	271	135185287	3.34993	2.92244	0.30123	ENSG00000154928:intron;ENSG00000240086:intron	ENSG00000240086	135138468-135439888	-	254544
3	135233078	135233347	270	135233284	4.36824	3.47775	0.59373	ENSG00000154928:intron;ENSG00000240086:intron	ENSG00000240086	135138468-135439888	-	206676
3	135513999	135514224	226	135514031	4.07388	3.28775	0.47041	intergenic	ENSG00000240086	135138468-135439888	-	-74223
3	135175157	135175362	206	135175324	4.51948	3.5757	0.59373	ENSG00000154928:intron;ENSG00000240086:intron	ENSG00000240086	135138468-135439888	-	264629
3	135185209	135185479	271	135185287	3.34993	2.92244	0.30123	ENSG00000154928:intron;ENSG00000240086:intron	ENSG00000240086	135138468-135439888	-	254544
3	135233078	135233347	270	135233284	4.36824	3.47775	0.59373	ENSG00000154928:intron;ENSG00000240086:intron	ENSG00000240086	135138468-135439888	-	206676
3	135513999	135514224	226	135514031	4.07388	3.28775	0.47041	intergenic	ENSG00000240086	135138468-135439888	-	-74223
3	137194779	137195066	288	137194814	3.34993	2.92244	0.30123	intergenic	ENSG00000249407	136959124-136982196	-	-212726
3	137236420	137236636	217	137236580	4.14216	3.33173	0.51073	intergenic	ENSG00000249407	136959124-136982196	-	-254331
3	138196194	138196488	295	138196426	4.14216	3.33173	0.51073	ENSG00000114098:intron	ENSG00000114098	138187247-138298389	+	9093
3	138406258	138406517	260	138406508	4.84177	3.65305	0.77787	intergenic	ENSG00000158220	138434585-138481686	+	-28198
3	140777228	140777454	227	140777357	5.17447	3.86417	1.0143	intergenic	ENSG00000261826	140865074-140867783	-	90442

3	141634872	141635123	252	141634978	6.14345	4.16882	1.69288	intergenic	ENSG00000242104	141660535-141729405	+	-25538
3	141659435	141659695	261	141659470	3.49438	2.91742	0.30123	ENSG00000242104:Promoter	ENSG00000242104	141660535-141729405	+	-970
3	141724517	141724749	233	141724549	6.26578	4.40475	1.75945	ENSG00000242104:intron	ENSG00000241526	141724424-141724526	+	208
3	143612456	143612829	374	143612479	4.07388	3.28775	0.47041	ENSG00000181804:intron	ENSG00000248374	143620527-143627690	+	-7885
3	143627320	143627734	415	143627562	3.49438	2.91742	0.30123	ENSG00000181804:intron;ENSG00000248374:exon	ENSG00000248374	143620527-143627690	+	6999
3	143689472	143689831	360	143689504	3.77589	3.20345	0.30123	ENSG00000181804:intron	ENSG00000248374	143620527-143627690	+	69124
3	143612456	143612829	374	143612479	4.07388	3.28775	0.47041	ENSG00000181804:intron	ENSG00000248374	143620527-143627690	+	-7885
3	143627320	143627734	415	143627562	3.49438	2.91742	0.30123	ENSG00000181804:intron;ENSG00000248374:exon	ENSG00000248374	143620527-143627690	+	6999
3	143689472	143689831	360	143689504	3.77589	3.20345	0.30123	ENSG00000181804:intron	ENSG00000248374	143620527-143627690	+	69124
3	143831360	143831596	237	143831498	11.02474	6.11671	5.94462	ENSG00000181804:intron	ENSG00000181804	143265221-143848531	-	17053
3	144808236	144808443	208	144808374	4.56295	3.60388	0.59373	intergenic	ENSG00000280397	144442210-144445844	-	-362495
3	146776948	146777275	328	146777112	5.39424	4.00432	1.09512	intergenic	ENSG00000242671	146921922-146925218	+	-144811
3	148298661	148299027	367	148298950	4.84177	3.65305	0.77787	intergenic	ENSG00000242440	148280890-148399956	+	17953
3	150050361	150050596	236	150050532	3.76522	3.1964	0.30123	ENSG00000240477:Promoter;ENSG00000070087:intron;ENSG00000243944:intron	ENSG00000240477	150050728-150051456	+	-250
3	152046263	152046517	255	152046425	3.03786	2.80302	0.15111	intergenic	ENSG00000239941	152118172-152151724	-	105334
3	152135098	152135445	348	152135162	4.56295	3.60388	0.59373	ENSG00000239941:intron	ENSG00000239941	152118172-152151724	-	16453
3	152171169	152171384	216	152171282	3.77589	3.20345	0.30123	intergenic	ENSG00000239941	152118172-152151724	-	-19552
3	152537745	152537980	236	152537928	4.76929	3.60726	0.74587	intergenic	ENSG00000243305	152457758-152496813	-	-41049
3	153399580	153399923	344	153399797	6.78327	4.56203	2.19117	ENSG00000238755:intron	ENSG00000241912	153376830-153377562	+	22921
3	153513290	153513509	220	153513487	4.56295	3.60388	0.59373	ENSG00000238755:intron	ENSG00000207323	153471465-153471571	-	-41828
3	153672190	153672395	206	153672343	3.78699	3.10368	0.30881	ENSG00000238755:intron	ENSG00000200162	153607376-153607479	+	64916
3	153735401	153735743	343	153735424	3.93333	3.08638	0.41301	ENSG00000238755:intron	ENSG00000200162	153607376-153607479	+	128195
3	156368273	156368527	255	156368384	8.17399	4.36112	3.38452	ENSG00000169282:intron	ENSG00000242370	156441156-156446905	-	78505
3	157065268	157065754	487	157065465	4.45692	3.17469	0.59373	intergenic	ENSG00000241544	157081840-157088547	+	-16329
3	157365070	157365320	251	157365169	5.13473	3.43925	0.98202	ENSG00000243176:intron;ENSG00000197415:intron	ENSG00000163661	157436788-157443628	+	-71593
3	157462989	157463228	240	157462996	3.20396	2.64557	0.26167	ENSG00000197415:intron	ENSG00000163661	157436788-157443628	+	26320
3	158286899	158287141	243	158287056	3.02441	2.62206	0.15111	ENSG00000174891:intron	ENSG00000241723	158275808-158276421	-	-10598
3	159418823	159419155	333	159418882	3.40215	2.85906	0.30123	ENSG00000283154:intron	ENSG00000244249	159386755-159387440	-	-31548
3	159455833	159456057	225	159455962	4.46483	3.29412	0.59373	ENSG00000283154:intron	ENSG00000244249	159386755-159387440	-	-68504
3	159974939	159975320	382	159974989	5.24809	3.63214	1.06984	ENSG00000244040:intron	ENSG00000168811	159988749-159996019	+	-13620
3	160207325	160207551	227	160207352	3.81246	2.91013	0.32594	ENSG00000244040:Promoter	ENSG00000244040	159909381-160225299	-	17861
3	161163306	161163652	347	161163522	9.91525	5.47441	4.94	ENSG00000169251:intron	ENSG00000169255	161083882-161105384	-	-58094
3	161474374	161474693	320	161474541	3.01636	2.45817	0.15111	intergenic	ENSG00000182447	161496807-161503942	+	-22274
3	161503295	161503558	264	161503407	3.6499	2.91341	0.30123	ENSG00000182447:exon	ENSG00000182447	161496807-161503942	+	6619
3	161511106	161511347	242	161511201	3.6499	2.91341	0.30123	intergenic	ENSG00000182447	161496807-161503942	+	14419
3	161536991	161537224	234	161537128	4.97105	3.59979	0.86452	intergenic	ENSG00000182447	161496807-161503942	+	40300
3	161474374	161474693	320	161474541	3.01636	2.45817	0.15111	intergenic	ENSG00000182447	161496807-161503942	+	-22274
3	161503295	161503558	264	161503407	3.6499	2.91341	0.30123	ENSG00000182447:exon	ENSG00000182447	161496807-161503942	+	6619
3	161511106	161511347	242	161511201	3.6499	2.91341	0.30123	intergenic	ENSG00000182447	161496807-161503942	+	14419
3	161536991	161537224	234	161537128	4.97105	3.59979	0.86452	intergenic	ENSG00000182447	161496807-161503942	+	40300
3	161474374	161474693	320	161474541	3.01636	2.45817	0.15111	intergenic	ENSG00000182447	161496807-161503942	+	-22274
3	161503295	161503558	264	161503407	3.6499	2.91341	0.30123	ENSG00000182447:exon	ENSG00000182447	161496807-161503942	+	6619
3	161511106	161511347	242	161511201	3.6499	2.91341	0.30123	intergenic	ENSG00000182447	161496807-161503942	+	14419
3	161536991	161537224	234	161537128	4.97105	3.59979	0.86452	intergenic	ENSG00000182447	161496807-161503942	+	40300
3	161866700	161866906	207	161866752	3.38496	2.66513	0.30123	intergenic	ENSG00000240354	161816908-161821908	-	-44894
3	162030162	162030400	239	162030344	3.81246	2.91013	0.32594	intergenic	ENSG00000240354	161816908-161821908	-	-208372

3	162404392	162404856	465	162404647	4.56295	3.60388	0.59373	intergenic	ENSG00000241874	162486540-162486949	-	82325
3	165538825	165539034	210	165538970	3.89605	3.1735	0.38387	ENSG00000244128:intron	ENSG00000114200	165772903-165837472	-	298543
3	165778435	165778667	233	165778511	6.70467	4.51337	2.12517	ENSG00000114200:intron	ENSG00000114200	165772903-165837472	-	58921
3	165856067	165856389	323	165856176	3.49438	2.91742	0.30123	intergenic	ENSG00000114200	165772903-165837472	-	-18755
3	165538825	165539034	210	165538970	3.89605	3.1735	0.38387	ENSG00000244128:intron	ENSG00000114200	165772903-165837472	-	298543
3	165778435	165778667	233	165778511	6.70467	4.51337	2.12517	ENSG00000114200:intron	ENSG00000114200	165772903-165837472	-	58921
3	165856067	165856389	323	165856176	3.49438	2.91742	0.30123	intergenic	ENSG00000114200	165772903-165837472	-	-18755
3	166478479	166478701	223	166478594	6.55036	4.2543	1.99411	intergenic	ENSG00000244321	166569692-166570763	-	92173
3	168085568	168085883	316	168085646	8.98232	4.95279	4.10924	ENSG00000173905:intron	ENSG00000173905	168008676-168095975	-	10250
3	168101350	168101640	291	168101432	3.13241	2.68948	0.2189	intergenic	ENSG00000173905	168008676-168095975	-	-5519
3	168415672	168415881	210	168415703	3.88967	3.0596	0.37913	ENSG00000206120:intron	ENSG00000222357	168475197-168475388	+	-59421
3	168431408	168431624	217	168431421	4.0114	3.13438	0.46103	ENSG00000206120:intron	ENSG00000222357	168475197-168475388	+	-43681
3	169905768	169905988	221	169905923	3.30754	2.7073	0.30123	ENSG00000240128:Promoter	ENSG00000240128	169902979-169904260	-	-1617
3	170133161	170133674	514	170133377	3.9797	2.90741	0.44999	ENSG00000173889:intron	ENSG00000199536	170149844-170149951	+	-16427
3	170571293	170571506	214	170571299	3.6499	2.91341	0.30123	ENSG00000285051:intron;ENSG00000013293:intron;ENSG00000285218:intron	ENSG00000013293	170459583-170586074	-	14675
3	170572907	170573216	310	170573052	9.78373	4.69287	4.83465	ENSG00000285051:intron;ENSG00000013293:intron;ENSG00000285218:intron	ENSG00000013293	170459583-170586074	-	13013
3	170897016	170897336	321	170897209	5.31489	3.67153	1.09512	ENSG00000163577:intron	ENSG00000163577	170888414-170908693	-	11517
3	171754776	171755012	237	171754911	4.0114	3.13438	0.46103	ENSG00000075651:intron	ENSG00000075651	171600404-171810950	-	56056
3	172095189	172095421	233	172095314	8.17399	4.36112	3.38452	ENSG00000075420:intron	ENSG00000075420	172039627-172401665	+	55677
3	172235936	172236222	287	172236127	5.18253	3.59359	1.02124	ENSG00000243398:Promoter:ENSG00000075420:intron	ENSG00000243398	172237325-172237591	+	-1246
3	172242588	172242796	209	172242704	4.2748	3.0719	0.58762	ENSG00000075420:intron	ENSG00000243398	172237325-172237591	+	5366
3	173596705	173597055	351	173596798	5.60156	3.98839	1.26447	ENSG00000169760:intron	ENSG00000237645	173644230-173644711	+	-47350
3	173612458	173612695	238	173612462	3.81246	2.91013	0.32594	ENSG00000169760:intron	ENSG00000237645	173644230-173644711	+	-31654
3	173920478	173920743	266	173920600	10.01593	5.14387	5.02923	ENSG00000169760:intron;ENSG00000228213:exon	ENSG00000228213	173910497-173920796	-	186
3	174241770	174241985	216	174241877	3.20396	2.64557	0.26167	ENSG00000169760:intron	ENSG00000232601	174302943-174303287	-	61410
3	174321702	174322031	330	174321983	3.92092	3.18945	0.4041	intergenic	ENSG00000232601	174302943-174303287	-	-18579
3	174613027	174613275	249	174613141	6.07934	3.97749	1.63735	intergenic	ENSG00000253020	174631822-174632064	+	-18671
3	175150825	175151191	367	175151073	3.20396	2.64557	0.26167	ENSG00000177694:intron	ENSG00000230292	175079306-175115242	-	-35765
3	175177719	175178008	290	175177940	3.45699	2.99296	0.30123	ENSG00000177694:intron	ENSG00000230292	175079306-175115242	-	-62621
3	175150825	175151191	367	175151073	3.20396	2.64557	0.26167	ENSG00000177694:intron	ENSG00000230292	175079306-175115242	-	-35765
3	175177719	175178008	290	175177940	3.45699	2.99296	0.30123	ENSG00000177694:intron	ENSG00000230292	175079306-175115242	-	-62621
3	175194170	175194466	297	175194322	10.60166	5.08797	5.56659	ENSG00000177694:intron	ENSG00000226779	175234860-175271096	-	76778
3	175317078	175317299	222	175317107	3.02441	2.62206	0.15111	ENSG00000177694:intron	ENSG00000226779	175234860-175271096	-	-46092
3	175332524	175332752	229	175332658	5.79352	3.81213	1.40054	ENSG00000177694:intron	ENSG00000264974	175369539-175369621	+	-36901
3	175347008	175347248	241	175347155	7.75292	4.61946	3.02978	ENSG00000177694:intron	ENSG00000264974	175369539-175369621	+	-22411
3	175332524	175332752	229	175332658	5.79352	3.81213	1.40054	ENSG00000177694:intron	ENSG00000264974	175369539-175369621	+	-36901
3	175347008	175347248	241	175347155	7.75292	4.61946	3.02978	ENSG00000177694:intron	ENSG00000264974	175369539-175369621	+	-22411
3	176055285	176055693	409	176055674	4.12863	3.32301	0.50868	intergenic	ENSG00000236357	176068041-176068908	-	13419
3	176157884	176158098	215	176158004	5.52306	3.79499	1.20409	intergenic	ENSG00000238026	176113701-176114537	-	-43453
3	176842333	176842569	237	176842521	5.01585	3.49609	0.89533	ENSG00000232461:intron	ENSG00000232461	176643940-176867838	-	25387
3	177359918	177360163	246	177360034	11.55211	4.89096	6.42066	intergenic	ENSG00000226782	177337627-177349166	-	-10874
3	177527038	177527259	222	177527206	6.12405	4.00354	1.67582	ENSG00000228221:intron	ENSG00000252028	177503302-177503552	-	-23596
3	177589954	177590279	326	177590105	12.96187	5.8878	7.72149	ENSG00000228221:intron	ENSG00000200288	177624281-177624409	-	34293
3	178320402	178320669	268	178320481	6.19969	4.04772	1.74352	ENSG00000197584:intron;ENSG00000223930:intron	ENSG00000197584	178272931-178844429	+	47604

3	178518863	178519406	544	178519295	10.65162	5.29115	5.60305	ENSG00000197584:intron;ENSG00000275163:intron	ENSG00000271131	178493326-178493810	-	-25324
3	179645971	179646439	469	179646067	5.55174	3.81208	1.22965	intergenic	ENSG00000243977	179649493-179650200	-	3995
3	181219355	181219750	396	181219406	5.15664	3.57839	1.00126	ENSG00000242808:intron	ENSG00000206932	181231736-181231843	+	-12184
3	181739407	181739704	298	181739451	3.6499	2.91341	0.30123	ENSG00000242808:intron	ENSG00000278026	181715184-181715293	+	24371
3	181955641	181955876	236	181955799	8.13775	4.49748	3.3598	ENSG00000242512:intron	ENSG00000242512	181952342-182010678	+	3416
3	182325516	182325953	438	182325762	7.58982	3.93998	2.87738	intergenic	ENSG00000242012	182365146-182368256	+	-39412
3	183156828	183157073	246	183156862	3.30754	2.7073	0.30123	ENSG00000078081:intron	ENSG00000078081	183122212-183163839	-	6889
3	183285845	183286077	233	183285923	5.20495	2.89643	1.04033	ENSG00000176597:intron;ENSG00000053524:intron	ENSG00000202502	183258299-183258416	-	-27544
3	183307043	183307411	369	183307113	6.21409	3.77143	1.75458	ENSG00000053524:intron	ENSG00000202502	183258299-183258416	-	-48810
3	183285845	183286077	233	183285923	5.20495	2.89643	1.04033	ENSG00000176597:intron;ENSG00000053524:intron	ENSG00000202502	183258299-183258416	-	-27544
3	183307043	183307411	369	183307113	6.21409	3.77143	1.75458	ENSG00000053524:intron	ENSG00000202502	183258299-183258416	-	-48810
3	183583689	183583948	260	183583878	3.81246	2.91013	0.32594	intergenic	ENSG00000172578	183487530-183555689	-	-28129
3	184646235	184646861	627	184646406	7.04714	4.22304	2.42833	intergenic	ENSG00000177383	184710366-184712002	-	65454
3	185537454	185537723	270	185537512	3.64915	2.8159	0.30123	ENSG00000163898:intron	ENSG00000163898	185506261-185552613	-	15025
3	185547294	185547498	205	185547386	4.67312	2.89995	0.67699	ENSG00000163898:intron	ENSG00000163898	185506261-185552613	-	5217
3	185537454	185537723	270	185537512	3.64915	2.8159	0.30123	ENSG00000163898:intron	ENSG00000163898	185506261-185552613	-	15025
3	185547294	185547498	205	185547386	4.67312	2.89995	0.67699	ENSG00000163898:intron	ENSG00000163898	185506261-185552613	-	5217
3	186278892	186279153	262	186278904	4.31475	3.20475	0.59373	ENSG00000058866:intron	ENSG00000058866	186105667-186362237	-	83215
3	186292474	186292697	224	186292514	4.25006	3.16641	0.57329	ENSG00000058866:intron	ENSG00000058866	186105667-186362237	-	69652
3	186278892	186279153	262	186278904	4.31475	3.20475	0.59373	ENSG00000058866:intron	ENSG00000058866	186105667-186362237	-	83215
3	186292474	186292697	224	186292514	4.25006	3.16641	0.57329	ENSG00000058866:intron	ENSG00000058866	186105667-186362237	-	69652
3	187065311	187065557	247	187065393	5.3206	3.11019	1.09512	ENSG00000073849:intron	ENSG00000163923	187120947-187180908	-	115474
3	187130796	187131052	257	187130884	11.3518	5.86412	6.24499	ENSG00000163923:intron	ENSG00000163923	187120947-187180908	-	49984
3	187065311	187065557	247	187065393	5.3206	3.11019	1.09512	ENSG00000073849:intron	ENSG00000163923	187120947-187180908	-	115474
3	187130796	187131052	257	187130884	11.3518	5.86412	6.24499	ENSG00000163923:intron	ENSG00000163923	187120947-187180908	-	49984
3	188831834	188832043	210	188831860	3.43291	2.87851	0.30123	ENSG00000145012:intron	ENSG00000188001	188947213-189325304	+	-115275
3	188875524	188875783	260	188875620	4.55755	3.47401	0.59373	ENSG00000145012:exon;ENSG00000145012:three_prime_UTR	ENSG00000188001	188947213-189325304	+	-71560
3	189194249	189194479	231	189194410	3.6499	2.91341	0.30123	ENSG00000188001:intron	ENSG00000230115	189238685-189240594	-	46230
3	192893045	192893301	257	192893204	7.69969	3.86859	2.97966	ENSG00000180611:intron	ENSG00000180611	192796814-192918161	-	24988
3	193012077	193012394	318	193012132	5.01585	3.49609	0.89533	intergenic	ENSG00000180611	192796814-192918161	-	-94074
3	192893045	192893301	257	192893204	7.69969	3.86859	2.97966	ENSG00000180611:intron	ENSG00000180611	192796814-192918161	-	24988
3	193012077	193012394	318	193012132	5.01585	3.49609	0.89533	intergenic	ENSG00000180611	192796814-192918161	-	-94074
3	193963381	193963627	247	193963481	5.045	3.38939	0.91868	ENSG00000214146:intron	ENSG00000238043	193980613-194006098	+	-17109
3	194785493	194785717	225	194785512	5.13473	3.43925	0.98202	intergenic	ENSG00000230401	194765237-194768712	-	-16892
3	195269196	195269433	238	195269384	5.09679	3.30224	0.956	ENSG00000173950:intron	ENSG00000173950	195068278-195271167	-	1853
3	195340359	195340596	238	195340581	3.81246	2.91013	0.32594	ENSG00000114331:intron	ENSG00000227433	195349663-195350030	+	-9186
3	196020645	196020902	258	196020837	3.6499	2.91341	0.30123	intergenic	ENSG00000228413	196044694-196045254	+	-23921
3	196274653	196274894	242	196274762	7.40928	4.11883	2.71399	ENSG00000161217:intron;ENSG00000272741:intron	ENSG00000161217	196214221-196287957	-	13184
3	197712355	197712639	285	197712398	4.92533	3.11051	0.829	ENSG00000145016:intron	ENSG00000122068	197737178-197787596	+	-24681
3	198153833	198154163	331	198153837	3.34993	2.92244	0.30123	intergenic	ENSG00000236438	198153286-198222513	+	711
3	198154885	198155155	271	198155005	3.03786	2.80302	0.15111	intergenic	ENSG00000236438	198153286-198222513	+	1733
3	198153833	198154163	331	198153837	3.34993	2.92244	0.30123	intergenic	ENSG00000236438	198153286-198222513	+	711
3	198154885	198155155	271	198155005	3.03786	2.80302	0.15111	intergenic	ENSG00000236438	198153286-198222513	+	1733
4	2746212	2746523	312	2746371	7.84906	4.50457	3.11977	ENSG00000168884:intron	ENSG00000168884	2741647-2756376	-	10009
4	4075193	4075437	245	4075229	4.56295	3.60388	0.59373	ENSG00000284727:intron	ENSG00000243894	4076710-4077491	-	2176

4	4248029	4248278	250	4248261	3.6499	2.91341	0.30123	ENSG00000132406:Promoter	ENSG00000132406	4235541-4248212	-	59
4	4987257	4987852	596	4987465	4.62651	3.51732	0.63659	intergenic	ENSG00000170891	5014585-5019472	-	31918
4	5006563	5006801	239	5006661	4.55072	3.59595	0.59373	intergenic	ENSG00000170891	5014585-5019472	-	12790
4	7058073	7058339	267	7058112	5.11088	3.68528	0.9653	intergenic	ENSG00000109519	7058905-7068197	-	9991
4	9604716	9604986	271	9604758	3.88371	3.16559	0.37576	intergenic	ENSG00000249799	9583941-9584131	-	-20719
4	9841925	9842197	273	9842137	4.56295	3.60388	0.59373	ENSG00000109667:intron	ENSG00000252002	9792750-9792861	-	-49199
4	10234898	10235193	296	10234973	8.1168	5.20561	3.35333	intergenic	ENSG00000271544	10238212-10238235	-	3190
4	10702685	10702901	217	10702782	5.39424	4.00432	1.09512	intergenic	ENSG00000249334	10685002-10697661	+	17790
4	11480602	11480950	349	11480765	5.39424	4.00432	1.09512	intergenic	ENSG00000251152	11469249-11478196	+	11526
4	17336656	17336968	313	17336832	10.54093	5.83399	5.51529	intergenic	ENSG00000206780	17320745-17320882	-	-15929
4	18356278	18356489	212	18356353	4.56295	3.60388	0.59373	intergenic	ENSG00000251048	18488061-18489508	-	133125
4	18378266	18378497	232	18378278	4.1849	3.24157	0.52478	intergenic	ENSG00000251048	18488061-18489508	-	111127
4	18466993	18467325	333	18467210	5.39424	4.00432	1.09512	intergenic	ENSG00000251048	18488061-18489508	-	22349
4	18503802	18504181	380	18504079	3.34993	2.92244	0.30123	intergenic	ENSG00000251048	18488061-18489508	-	-14483
4	18702868	18703126	259	18702951	3.77589	3.20345	0.30123	intergenic	ENSG00000251048	18488061-18489508	-	-213488
4	18934436	18934716	281	18934484	3.49438	2.91742	0.30123	intergenic	ENSG00000279918	19098198-19098740	+	-163622
4	21120066	21120359	294	21120183	8.1168	5.20561	3.35333	ENSG00000185774:intron	ENSG00000250243	21304541-21316485	+	-184329
4	21244088	21244385	298	21244311	3.77589	3.20345	0.30123	ENSG00000185774:intron	ENSG00000250243	21304541-21316485	+	-60305
4	21355827	21356114	288	21356047	3.03786	2.80302	0.15111	ENSG00000185774:intron	ENSG00000250243	21304541-21316485	+	51429
4	25539761	25539980	220	25539928	3.40931	2.96154	0.30123	intergenic	ENSG00000248608	25504996-25506675	+	34874
4	26262741	26262981	241	26262935	3.03786	2.80302	0.15111	ENSG00000168214:intron	ENSG00000168214	26163454-26435131	+	99406
4	27791080	27791289	210	27791158	3.34993	2.92244	0.30123	intergenic	ENSG00000251410	27967722-27985063	-	193879
4	27814028	27814331	304	27814212	8.1168	5.20561	3.35333	intergenic	ENSG00000251410	27967722-27985063	-	170884
4	33973025	33973256	232	33973074	3.88371	3.16559	0.37576	ENSG00000250723:intron;ENSG00000250954:intron	ENSG00000239983	33968173-33968526	+	4967
4	38132321	38132591	271	38132463	8.0161	4.5963	3.27031	ENSG00000065882:intron	ENSG00000249649	38006783-38007105	-	-125350
4	38306693	38306929	237	38306813	5.75051	3.78743	1.36222	intergenic	ENSG00000251642	38286993-38287491	+	19817
4	38752366	38752604	239	38752437	12.35063	6.66347	7.14928	intergenic	ENSG00000222230	38758790-38758892	-	6407
4	38752366	38752604	239	38752437	12.35063	6.66347	7.14928	intergenic	ENSG00000222230	38758790-38758892	-	6407
4	38996081	38996479	399	38996243	7.96683	4.92425	3.22527	ENSG00000121895:intron	ENSG00000121895	38966743-39032922	-	36642
4	39770002	39770298	297	39770094	4.47325	3.54574	0.59373	ENSG00000078140:intron;ENSG00000180610:exon	ENSG00000180610	39770080-39771371	-	1221
4	40769779	40770003	225	40769914	5.39424	4.00432	1.09512	ENSG00000179299:intron	ENSG00000248780	40786109-40786710	+	-16218
4	42167755	42167982	228	42167815	7.57321	4.69036	2.86415	ENSG00000285454:intron	ENSG00000188848	42110937-42152878	-	-14990
4	42437040	42437274	235	42437109	3.6499	2.91341	0.30123	ENSG00000124406:intron	ENSG00000240167	42471809-42472380	+	-34652
4	42529527	42529760	234	42529563	3.88371	3.16559	0.37576	ENSG00000124406:intron	ENSG00000248283	42561456-42562425	-	32782
4	43835962	43836204	243	43836118	6.09579	4.29768	1.6498	intergenic	ENSG00000239679	43863273-43863549	+	-27190
4	48127959	48128461	503	48128170	36.73342	13.71756	30.60637	ENSG00000074966:intron	ENSG00000074966	48066392-48134256	-	6046
4	49490044	49490330	287	49490223	3.7121	2.85213	0.30123	ENSG00000248583:Promoter	ENSG00000248583	49486925-49489554	-	-632
4	49494193	49494396	204	49494246	4.40096	2.37427	0.59373	intergenic	ENSG00000248583	49486925-49489554	-	-4740
4	49709339	49709557	219	49709431	11.88032	6.15899	6.72383	intergenic	ENSG00000249337	49588771-49589529	+	120676
4	49709339	49709557	219	49709431	11.88032	6.15899	6.72383	intergenic	ENSG00000249337	49588771-49589529	+	120676
4	51896055	51896391	337	51896270	4.56295	3.60388	0.59373	ENSG00000109184:intron	ENSG00000272576	51918771-51919381	+	-22548
4	52035526	52035835	310	52035627	4.12863	3.32301	0.50868	ENSG00000163069:intron	ENSG00000163069	52020705-52038482	-	2802
4	52701318	52701687	370	52701568	4.12863	3.32301	0.50868	intergenic	ENSG00000226950	52712403-52720351	+	-10901
4	52716419	52716635	217	52716527	4.88847	3.6826	0.81688	ENSG00000226950:intron	ENSG00000212588	52713248-52713370	+	3278
4	53688247	53688477	231	53688325	7.40928	4.11883	2.71399	ENSG00000249341:intron;ENSG00000282278:intron	ENSG00000072201	53459300-53701405	-	13043
4	54771208	54771633	426	54771332	5.39424	4.00432	1.09512	intergenic	ENSG00000249727	54836160-54845402	-	73982
4	55239393	55239932	540	55239547	5.24168	3.90698	1.06394	intergenic	ENSG00000250812	55219343-55219973	+	20319
4	59590283	59590633	351	59590364	3.77589	3.20345	0.30123	ENSG00000249392:intron	ENSG00000249392	59551141-59630324	-	39866

4	59639265	59639470	206	59639392	3.77589	3.20345	0.30123	intergenic	ENSG00000249392	59551141-59630324	-	-9043
4	64001289	64001538	250	64001423	9.22287	5.47958	4.30742	intergenic	ENSG00000252104	64397693-64397787	-	396374
4	64290151	64290402	252	64290354	3.34993	2.92244	0.30123	ENSG00000205678:intron	ENSG00000252104	64397693-64397787	-	107511
4	64816575	64816988	414	64816840	5.39424	4.00432	1.09512	intergenic	ENSG00000244669	64767129-64767897	-	-48884
4	66014390	66014744	355	66014419	3.34993	2.92244	0.30123	ENSG00000249413:intron;ENSG00000272304:intron	ENSG00000272304	66003280-66016792	+	11286
4	66901720	66902126	407	66901786	4.56295	3.60388	0.59373	intergenic	ENSG00000252890	66897261-66897371	+	4661
4	66911955	66912258	304	66912108	9.07088	5.59371	4.18166	intergenic	ENSG00000252890	66897261-66897371	+	14845
4	72003496	72003703	208	72003596	4.919	3.70193	0.82412	intergenic	ENSG00000056291	72031803-72148067	+	-28204
4	72112566	72112857	292	72112718	8.1168	5.20561	3.35333	ENSG00000056291:intron	ENSG00000056291	72031803-72148067	+	80908
4	72703833	72704102	270	72703964	3.03786	2.80302	0.15111	intergenic	ENSG00000270665	72807266-72808192	+	-103299
4	72809451	72809780	330	72809536	3.49438	2.91742	0.30123	intergenic	ENSG00000270665	72807266-72808192	+	2349
4	75555870	75556146	277	75555968	11.53275	5.55571	6.4016	ENSG00000174792:Promoter	ENSG00000174792	75556047-75565885	+	-39
4	75967312	75967533	222	75967403	5.17447	3.86417	1.0143	ENSG00000198301:intron	ENSG00000245928	75980789-76005942	+	-13367
4	76199682	76199905	224	76199805	6.58155	3.96836	2.01637	ENSG00000138760:intron	ENSG00000189157	76214039-76283780	+	-14246
4	76735354	76735594	241	76735497	3.77589	3.20345	0.30123	ENSG00000138771:intron	ENSG00000233860	76756959-76758474	-	23000
4	76991127	76991457	331	76991335	5.14139	3.84312	0.98688	ENSG00000138758:intron	ENSG00000234036	76958859-76959169	-	-32122
4	77425177	77425538	362	77425534	3.02441	2.62206	0.15111	ENSG00000138764:intron;ENSG00000249036:intron	ENSG00000249036	77394490-77494286	-	68929
4	77923069	77923328	260	77923153	3.02441	2.62206	0.15111	ENSG00000169288:intron	ENSG00000248926	77957999-77958780	-	35582
4	77934869	77935115	247	77935098	5.68188	3.88989	1.31322	ENSG00000169288:intron	ENSG00000248926	77957999-77958780	-	23788
4	80509651	80509970	320	80509783	5.39424	4.00432	1.09512	ENSG00000197826:intron	ENSG00000232327	80386177-80388716	-	-121094
4	81257909	81258187	279	81258018	4.87332	3.67301	0.80353	intergenic	ENSG00000138669	81087369-81215117	-	-42930
4	81550725	81550950	226	81550836	3.77589	3.20345	0.30123	ENSG00000138670:intron	ENSG00000248282	81733384-81734207	-	183370
4	81631954	81632177	224	81632045	6.57074	4.43068	2.0119	ENSG00000138670:intron	ENSG00000248282	81733384-81734207	-	102142
4	81725112	81725414	303	81725246	15.03591	6.29939	9.62995	ENSG00000138670:intron	ENSG00000248282	81733384-81734207	-	8944
4	81550725	81550950	226	81550836	3.77589	3.20345	0.30123	ENSG00000138670:intron	ENSG00000248282	81733384-81734207	-	183370
4	81631954	81632177	224	81632045	6.57074	4.43068	2.0119	ENSG00000138670:intron	ENSG00000248282	81733384-81734207	-	102142
4	81725112	81725414	303	81725246	15.03591	6.29939	9.62995	ENSG00000138670:intron	ENSG00000248282	81733384-81734207	-	8944
4	82905232	82905529	298	82905370	12.86462	6.48314	7.62811	ENSG00000168152:intron	ENSG00000138674	82818660-82901166	-	-4214
4	86863726	86863965	240	86863766	4.07388	3.28775	0.47041	intergenic	ENSG00000243312	86870190-86871404	+	-6345
4	87284477	87284727	251	87284607	4.99664	3.75114	0.88739	intergenic	ENSG00000263981	87300494-87300583	-	15981
4	88119059	88119342	284	88119236	6.26578	4.40475	1.75945	ENSG00000118777:intron	ENSG00000241853	88163578-88163967	+	-44378
4	89222190	89222497	308	89222288	11.05132	6.13235	5.96877	intergenic	ENSG00000185477	89236385-89308010	-	85667
4	89874570	89875034	465	89874852	6.26578	4.40475	1.75945	intergenic	ENSG00000138722	89879531-89954629	+	-4729
4	93602305	93602633	329	93602454	5.39424	4.00432	1.09512	ENSG00000152208:intron	ENSG00000252342	93820170-93820280	+	-217701
4	93822435	93822703	269	93822541	4.56295	3.60388	0.59373	intergenic	ENSG00000252342	93820170-93820280	+	2398
4	93602305	93602633	329	93602454	5.39424	4.00432	1.09512	ENSG00000152208:intron	ENSG00000252342	93820170-93820280	+	-217701
4	93822435	93822703	269	93822541	4.56295	3.60388	0.59373	intergenic	ENSG00000252342	93820170-93820280	+	2398
4	97851049	97851279	231	97851092	4.42776	3.51628	0.59373	ENSG00000163116:intron	ENSG00000250293	97916352-97917401	-	66237
4	99048145	99048415	271	99048357	6.09579	4.29768	1.6498	ENSG00000164024:intron	ENSG00000272777	99067255-99068125	-	19845
4	99054341	99054605	265	99054380	3.77589	3.20345	0.30123	ENSG00000164024:intron	ENSG00000272777	99067255-99068125	-	13652
4	100101157	100101409	253	100101261	7.98084	5.12163	3.23913	ENSG00000245322:intron	ENSG00000248820	100041840-100042104	-	-59178
4	100487632	100488017	386	100487746	3.42114	2.96933	0.30123	ENSG00000164035:intron	ENSG00000250223	100660278-100675113	-	187289
4	101259934	101260237	304	101260089	14.89517	7.13146	9.49496	ENSG00000138814:intron	ENSG00000273882	101240794-101240872	-	-19213
4	101526503	101526867	365	101526784	4.56295	3.60388	0.59373	ENSG00000153064:intron	ENSG00000153064	101411285-102074812	+	115399
4	102469784	102470035	252	102469889	4.56295	3.60388	0.59373	intergenic	ENSG00000251572	102461249-102462152	+	8660
4	103032461	103032816	356	103032656	4.50398	3.56566	0.59373	ENSG00000164038:intron	ENSG00000164037	102885047-103019739	-	-12899
4	105060581	105061031	451	105060802	15.2807	7.87943	9.85742	ENSG00000248373:intron	ENSG00000251473	105102890-105104004	-	43198
4	105077891	105078140	250	105077947	4.57122	3.48259	0.60109	ENSG00000248373:intron	ENSG00000251473	105102890-105104004	-	25989
4	105306312	105306622	311	105306479	10.09471	6.00647	5.09428	intergenic	ENSG00000243383	105293663-105293946	+	12803

4	106158259	106158509	251	106158380	5.56876	3.96799	1.238	ENSG00000145348:intron	ENSG00000249635	106003316-106022478	-	-135905
4	107637896	107638141	246	107637935	3.03786	2.80302	0.15111	ENSG00000138801:intron	ENSG00000138801	107590275-107720452	-	82434
4	107755819	107756178	360	107756130	5.91134	4.18186	1.4976	intergenic	ENSG00000138801	107590275-107720452	-	-35546
4	108118564	108118843	280	108118774	6.4824	4.21404	1.94623	ENSG00000138795:intron	ENSG00000232021	108167524-108256836	+	-48821
4	111064355	111064674	320	111064495	17.81506	8.80949	12.26624	intergenic	ENSG00000251312	111063582-111064227	+	932
4	111268766	111269017	252	111268926	7.082	4.57252	2.45993	intergenic	ENSG00000200963	111331411-111331518	+	-62520
4	111400674	111401035	362	111400780	4.56295	3.60388	0.59373	intergenic	ENSG00000200963	111331411-111331518	+	69443
4	111414782	111415099	318	111415057	3.77589	3.20345	0.30123	intergenic	ENSG00000200963	111331411-111331518	+	83529
4	111435270	111435488	219	111435291	3.77589	3.20345	0.30123	intergenic	ENSG00000200963	111331411-111331518	+	103967
4	111268766	111269017	252	111268926	7.082	4.57252	2.45993	intergenic	ENSG00000200963	111331411-111331518	+	-62520
4	111400674	111401035	362	111400780	4.56295	3.60388	0.59373	intergenic	ENSG00000200963	111331411-111331518	+	69443
4	111414782	111415099	318	111415057	3.77589	3.20345	0.30123	intergenic	ENSG00000200963	111331411-111331518	+	83529
4	111435270	111435488	219	111435291	3.77589	3.20345	0.30123	intergenic	ENSG00000200963	111331411-111331518	+	103967
4	120223973	120224232	260	120224155	5.39424	4.00432	1.09512	ENSG00000250938:intron	ENSG00000225665	120342055-120342647	+	-117953
4	123588660	123588926	267	123588865	5.8578	4.14832	1.45441	intergenic	ENSG00000249464	123650266-123930406	+	-61473
4	123966400	123966669	270	123966553	3.49438	2.91742	0.30123	intergenic	ENSG00000250484	124062461-124062990	+	-95927
4	124102337	124102556	220	124102531	3.34993	2.92244	0.30123	intergenic	ENSG00000250484	124062461-124062990	+	39985
4	124685107	124685343	237	124685165	3.53038	3.04135	0.30123	ENSG00000151458:intron	ENSG00000151458	124664051-124712732	-	27507
4	127216213	127216441	229	127216356	4.84177	3.65305	0.77787	ENSG00000248491:intron	ENSG00000250945	127043429-127077762	-	-138564
4	127229500	127229725	226	127229537	4.56295	3.60388	0.59373	ENSG00000248491:intron	ENSG00000250945	127043429-127077762	-	-151850
4	127235119	127235324	206	127235272	3.34993	2.92244	0.30123	ENSG00000248491:intron	ENSG00000250945	127043429-127077762	-	-157459
4	128452611	128452923	313	128452744	4.1849	3.24157	0.52478	ENSG00000251432:intron	ENSG00000273077	128552589-128553416	-	100649
4	128552575	128552962	388	128552928	4.49742	2.90144	0.59373	ENSG00000273077:intron	ENSG00000273077	128552589-128553416	-	648
4	128452611	128452923	313	128452744	4.1849	3.24157	0.52478	ENSG00000251432:intron	ENSG00000273077	128552589-128553416	-	100649
4	128552575	128552962	388	128552928	4.49742	2.90144	0.59373	ENSG00000273077:intron	ENSG00000273077	128552589-128553416	-	648
4	128653659	128653902	244	128653763	5.39424	4.00432	1.09512	intergenic	ENSG00000248802	128582998-128601407	-	-52373
4	141128209	141128468	260	141128358	4.98096	3.7412	0.87315	ENSG00000170153:intron	ENSG00000170153	140859806-141212877	-	84539
4	141152580	141152952	373	141152727	4.56295	3.60388	0.59373	ENSG00000170153:intron	ENSG00000170153	140859806-141212877	-	60111
4	141128209	141128468	260	141128358	4.98096	3.7412	0.87315	ENSG00000170153:intron	ENSG00000170153	140859806-141212877	-	84539
4	141152580	141152952	373	141152727	4.56295	3.60388	0.59373	ENSG00000170153:intron	ENSG00000170153	140859806-141212877	-	60111
4	142641321	142641589	269	142641561	3.77589	3.20345	0.30123	ENSG00000249806:intron;ENSG00000109452:intron	ENSG00000249806	142566018-142660950	+	75436
4	142880628	142880855	228	142880669	4.22478	3.38503	0.55558	intergenic	ENSG00000109452	142023159-142847432	-	-33309
4	142919928	142920221	294	142920029	3.77589	3.20345	0.30123	intergenic	ENSG00000109452	142023159-142847432	-	-72642
4	143863885	143864119	235	143863998	4.56295	3.60388	0.59373	ENSG00000251600:intron	ENSG00000197465	143870866-143905563	-	41561
4	144502860	144503109	250	144502933	6.15301	4.33368	1.70124	ENSG00000285713:intron;ENSG00000285783:intron	ENSG00000261129	144505899-144509001	+	-2915
4	145455979	145456205	227	145456049	4.07388	3.28775	0.47041	ENSG00000248745:exon	ENSG00000248745	145456004-145456844	+	87
4	148089745	148089952	208	148089853	3.658	3.12558	0.30123	ENSG00000151623:intron	ENSG00000250354	148146470-148208880	+	-56622
4	148163660	148163948	289	148163665	5.11088	3.68528	0.9653	ENSG00000151623:intron;ENSG00000250354:intron	ENSG00000250354	148146470-148208880	+	17333
4	148232011	148232270	260	148232144	3.77589	3.20345	0.30123	ENSG00000151623:intron	ENSG00000250354	148146470-148208880	+	85670
4	150250338	150250609	272	150250421	3.77589	3.20345	0.30123	ENSG00000170390:intron	ENSG00000238721	150209705-150209768	+	40768
4	150288906	150289127	222	150288996	3.77589	3.20345	0.30123	ENSG00000198589:intron	ENSG00000238721	150209705-150209768	+	79311
4	151439233	151439665	433	151439336	4.56295	3.60388	0.59373	ENSG00000164142:intron	ENSG00000164142	151409215-151663632	+	30233
4	151798731	151798959	229	151798882	3.02441	2.62206	0.15111	ENSG00000249184:Promoter	ENSG00000249184	151799499-151801348	+	-654
4	152697000	152697229	230	152697124	7.86063	4.35185	3.13082	intergenic	ENSG00000170006	152618631-152680165	-	-16949
4	152709487	152709859	373	152709743	4.54395	3.46548	0.59373	intergenic	ENSG00000170006	152618631-152680165	-	-29507
4	153859074	153859279	206	153859255	4.22478	3.38503	0.55558	intergenic	ENSG00000145423	153780591-153789120	-	-70056
4	153859074	153859279	206	153859255	4.22478	3.38503	0.55558	intergenic	ENSG00000145423	153780591-153789120	-	-70056
4	158823478	158823835	358	158823669	9.23263	5.09091	4.31623	ENSG00000052795:intron	ENSG00000206703	158794589-158794696	-	-28960

4	163235531	163235814	284	163235759	3.64836	3.01523	0.30123	intergenic	ENSG00000250547	163265574-163266583	-	30911
4	165419939	165420142	204	165420092	3.77589	3.20345	0.30123	ENSG00000109472:intron	ENSG00000251596	165404066-165406350	-	-13690
4	165426873	165427228	356	165427061	4.56295	3.60388	0.59373	ENSG00000109472:intron	ENSG00000251596	165404066-165406350	-	-20700
4	169115283	169115515	233	169115308	4.84177	3.65305	0.77787	ENSG00000154447:intron	ENSG00000214671	169166271-169167117	+	-50872
4	172920997	172921235	239	172921131	5.24168	3.90698	1.06394	ENSG00000174473:intron	ENSG00000241652	173141642-173141938	+	-220526
4	173626034	173626323	290	173626273	4.56295	3.60388	0.59373	ENSG00000237125:intron	ENSG00000213370	173633727-173634371	-	8193
4	173840041	173840285	245	173840061	4.56295	3.60388	0.59373	intergenic	ENSG00000250708	173897251-173929525	+	-57088
4	174348192	174348443	252	174348339	3.77589	3.20345	0.30123	intergenic	ENSG00000249875	174354853-174376445	-	28128
4	174358924	174359162	239	174359036	4.1849	3.24157	0.52478	ENSG00000249875:intron	ENSG00000249875	174354853-174376445	-	17402
4	175446039	175446355	317	175446339	3.34993	2.92244	0.30123	intergenic	ENSG00000249945	175458288-175466697	-	20500
4	176573122	176573424	303	176573311	4.84177	3.65305	0.77787	intergenic	ENSG00000248480	176631391-176633231	-	59958
4	176746091	176746321	231	176746157	4.1849	3.24157	0.52478	ENSG00000150630:intron	ENSG00000150630	176683537-176792727	-	46521
4	179741966	179742250	285	179742025	5.38044	3.99551	1.09512	intergenic	ENSG00000248921	179856852-179857581	+	-114744
4	181752545	181752782	238	181752629	6.36907	4.30669	1.85528	intergenic	ENSG00000251336	181820018-181829973	-	77310
4	181783765	181784010	246	181783840	4.56295	3.60388	0.59373	intergenic	ENSG00000251336	181820018-181829973	-	46086
4	184815517	184815740	224	184815626	6.43235	3.75515	1.9085	ENSG00000251139:intron;ENSG00000151726:intron	ENSG00000251139	184813618-184821300	+	2010
4	185033986	185034292	307	185034171	7.082	4.57252	2.45993	intergenic	ENSG00000187821	185018840-185020804	+	15298
4	185413025	185413274	250	185413054	3.49438	2.91742	0.30123	ENSG00000109775:intron	ENSG00000207231	185415972-185416086	-	2937
4	186147882	186148092	211	186148023	3.03786	2.80302	0.15111	ENSG00000109794:intron	ENSG00000250327	186157895-186158843	+	-9908
4	186162230	186162516	287	186162469	4.30996	3.44006	0.59373	ENSG00000109794:intron	ENSG00000250327	186157895-186158843	+	4477
4	186899369	186899836	468	186899517	5.32771	3.96184	1.09512	ENSG00000249539:Promoter	ENSG00000249539	186900539-186900838	+	-937
4	189425468	189425675	208	189425629	3.34993	2.92244	0.30123	intergenic	ENSG00000205100	189472964-189475192	+	-47393
4	189553381	189553648	268	189553523	5.39424	4.00432	1.09512	intergenic	ENSG00000205100	189472964-189475192	+	80550
4	189631229	189631553	325	189631408	11.1261	6.40691	6.0347	intergenic	ENSG00000250739	189659604-189661486	+	-28213
4	190111107	190111426	320	190111305	4.2748	3.0719	0.58762	intergenic	ENSG00000280457	190091004-190092279	+	20262
5	4585151	4585449	299	4585271	5.39424	4.00432	1.09512	ENSG00000248973:intron	ENSG00000251371	4640730-4648254	+	-55430
5	6829467	6829752	286	6829613	11.18335	6.21019	6.08588	ENSG00000251365:intron;ENSG00000283420:Promoter	ENSG00000283420	6827852-6827921	-	-1688
5	7672719	7672992	274	7672858	4.33895	3.45881	0.59373	ENSG00000078295:intron	ENSG00000250761	7707801-7749766	-	76911
5	10765598	10765952	355	10765719	4.04884	3.27164	0.47041	ENSG00000272324:intron	ENSG00000112977	10679229-10761272	-	-4502
5	11050513	11050732	220	11050586	6.26578	4.40475	1.75945	ENSG00000169862:intron	ENSG00000207312	11027199-11027307	+	23423
5	11052510	11052800	291	11052607	6.49439	4.38367	1.95029	ENSG00000169862:intron	ENSG00000207312	11027199-11027307	+	25455
5	11068669	11068930	262	11068880	3.76522	3.1964	0.30123	ENSG00000169862:intron	ENSG00000207312	11027199-11027307	+	41600
5	11082650	11082866	217	11082746	3.77589	3.20345	0.30123	ENSG00000169862:exon	ENSG00000207312	11027199-11027307	+	55558
5	13624285	13624508	224	13624333	5.75307	4.08283	1.36357	intergenic	ENSG00000243744	13637918-13638381	-	13985
5	14613471	14613813	343	14613770	4.07388	3.28775	0.47041	intergenic	ENSG00000248791	14620019-14621313	+	-6377
5	14809725	14810008	284	14809826	8.99866	4.4694	4.12107	ENSG00000154122:intron	ENSG00000249485	14797124-14798400	-	-11466
5	14918442	14918697	256	14918455	3.40931	2.96154	0.30123	intergenic	ENSG00000250662	14877666-14877919	-	-40650
5	18698305	18698531	227	18698467	3.03786	2.80302	0.15111	intergenic	ENSG00000248693	18704432-18746173	-	47755
5	18746409	18746692	284	18746496	7.17425	4.80518	2.52654	ENSG00000248693:Promoter	ENSG00000248693	18704432-18746173	-	-377
5	24893028	24893449	422	24893188	6.26578	4.40475	1.75945	intergenic	ENSG00000250524	24881942-24885461	-	-7777
5	27252949	27253314	366	27253076	25.71253	10.82179	19.88726	intergenic	ENSG00000113100	26880599-27121150	-	-131981
5	27277506	27277715	210	27277603	4.56295	3.60388	0.59373	intergenic	ENSG00000113100	26880599-27121150	-	-156460
5	29078716	29079228	513	29079032	6.26578	4.40475	1.75945	intergenic	ENSG00000252601	29070495-29070610	+	8476
5	32542642	32542907	266	32542786	13.56604	6.40949	8.26891	ENSG00000113387:intron	ENSG00000113387	32531632-32604079	+	11142
5	33135154	33135385	232	33135282	4.919	3.70193	0.82412	ENSG00000250697:intron	ENSG00000240376	33162178-33162807	+	-26909
5	34959363	34959666	304	34959540	15.49818	8.00863	10.06014	intergenic	ENSG00000168724	34929558-34958964	+	29956
5	36310120	36310336	217	36310154	3.03786	2.80302	0.15111	intergenic	ENSG00000164188	36248433-36302114	-	-8113
5	37465481	37465833	353	37465554	4.56295	3.60388	0.59373	ENSG00000082068:intron	ENSG00000248216	37505687-37506857	-	41200
5	39077040	39077268	229	39077168	6.78327	4.56203	2.19117	intergenic	ENSG00000164327	38937918-39074408	-	-2745

5	39234592	39234924	333	39234748	4.07388	3.28775	0.47041	ENSG00000082074:intron	ENSG00000082074	39105254-39274528	-	39770
5	39691870	39692260	391	39692167	3.58061	3.07449	0.30123	intergenic	ENSG00000250492	39718983-39721513	-	29448
5	40000774	40001105	332	40000954	4.56295	3.60388	0.59373	ENSG00000250048:intron	ENSG00000249668	40066650-40067200	+	-65711
5	41761846	41762113	268	41761969	4.07388	3.28775	0.47041	ENSG00000083720:intron	ENSG00000248668	41870029-41872241	+	-108050
5	41835711	41835963	253	41835791	6.26578	4.40475	1.75945	ENSG00000083720:intron	ENSG00000248668	41870029-41872241	+	-34192
5	42259594	42259870	277	42259821	3.77589	3.20345	0.30123	intergenic	ENSG00000260786	42188265-42191523	+	71466
5	42343592	42343831	240	42343637	5.2587	3.91783	1.07467	intergenic	ENSG00000112964	42423776-42721878	+	-80065
5	42950685	42950948	264	42950868	8.17399	4.36112	3.38452	intergenic	ENSG00000250711	42972623-42973092	+	-21807
5	50183378	50183622	245	50183503	3.77589	3.20345	0.30123	intergenic	ENSG00000170571	50396191-50443248	-	259748
5	50183378	50183622	245	50183503	3.77589	3.20345	0.30123	intergenic	ENSG00000170571	50396191-50443248	-	259748
5	52179930	52180159	230	52180016	3.63193	3.10837	0.30123	intergenic	ENSG00000244621	52282566-52282859	+	-102522
5	54996648	54996928	281	54996801	6.26578	4.40475	1.75945	ENSG00000164283:intron	ENSG00000164283	54977863-55022671	-	25883
5	55262248	55262463	216	55262355	3.03786	2.80302	0.15111	ENSG00000067248:intron;ENSG00000251307:intron	ENSG00000251307	55233933-55295201	+	28422
5	56148481	56148732	252	56148645	7.21064	4.47768	2.55784	ENSG00000164512:intron;ENSG00000223003:Promoter	ENSG00000223003	56146738-56146846	-	-1760
5	56328908	56329239	332	56329093	17.58419	7.57485	12.04595	intergenic	ENSG00000249697	56313904-56321397	-	-7676
5	58527884	58528106	223	58527993	3.02441	2.62206	0.15111	intergenic	ENSG00000265699	58530042-58530109	-	2114
5	58648232	58648607	376	58648407	6.26578	4.40475	1.75945	ENSG00000152932:intron	ENSG00000152932	58582220-58859394	+	66199
5	59233693	59234010	318	59233818	5.48809	3.9179	1.17181	ENSG00000113448:intron	ENSG00000276214	59314109-59314800	+	-80258
5	59413049	59413280	232	59413144	3.03786	2.80302	0.15111	ENSG00000113448:intron	ENSG00000276214	59314109-59314800	+	99055
5	59512120	59512436	317	59512299	3.77589	3.20345	0.30123	ENSG00000113448:intron	ENSG00000251306	59528860-59529251	+	-16582
5	59562967	59563246	280	59563148	6.87919	4.45046	2.28002	ENSG00000113448:intron	ENSG00000251306	59528860-59529251	+	34246
5	62678647	62678901	255	62678895	3.29424	2.88581	0.30123	intergenic	ENSG00000217416	62776876-62777263	-	98489
5	62681429	62681798	370	62681443	3.03786	2.80302	0.15111	intergenic	ENSG00000217416	62776876-62777263	-	95650
5	66149262	66149618	357	66149397	4.64049	3.52611	0.64891	ENSG00000153914:intron	ENSG00000253744	66144155-66144795	-	-4644
5	66730747	66731162	416	66731066	4.07388	3.28775	0.47041	ENSG00000069020:intron	ENSG00000249057	66662330-66662988	+	68624
5	66829857	66830127	271	66829998	4.31475	3.20475	0.59373	ENSG00000069020:intron	ENSG00000249057	66662330-66662988	+	167661
5	66730747	66731162	416	66731066	4.07388	3.28775	0.47041	ENSG00000069020:intron	ENSG00000249057	66662330-66662988	+	68624
5	66829857	66830127	271	66829998	4.31475	3.20475	0.59373	ENSG00000069020:intron	ENSG00000249057	66662330-66662988	+	167661
5	66840745	66841053	309	66840934	13.07138	7.09148	7.82275	ENSG00000069020:intron	ENSG00000229666	67001382-67003953	-	163054
5	67006608	67006837	230	67006730	6.00239	4.23898	1.57533	ENSG00000069020:intron	ENSG00000229666	67001382-67003953	-	-2769
5	66840745	66841053	309	66840934	13.07138	7.09148	7.82275	ENSG00000069020:intron	ENSG00000229666	67001382-67003953	-	163054
5	67006608	67006837	230	67006730	6.00239	4.23898	1.57533	ENSG00000069020:intron	ENSG00000229666	67001382-67003953	-	-2769
5	67206948	67207238	291	67207040	11.3238	6.29327	6.21788	intergenic	ENSG00000134061	67179612-67196799	-	-10293
5	68434240	68434565	326	68434394	11.51664	5.74538	6.39216	ENSG00000248884:exon	ENSG00000248884	68430426-68434481	-	79
5	71653595	71653989	395	71653927	4.07388	3.28775	0.47041	ENSG00000131844:intron	ENSG00000164326	71719162-71721048	+	-65370
5	72444113	72444331	219	72444223	4.35972	3.35026	0.59373	ENSG00000178175:exon;ENSG00000261269:Promoter	ENSG00000261269	72439902-72442387	-	-1834
5	73464025	73464242	218	73464114	4.56295	3.60388	0.59373	ENSG00000251324:intron	ENSG00000251324	73454186-73472896	-	8763
5	73485345	73485786	442	73485412	4.56295	3.60388	0.59373	intergenic	ENSG00000251324	73454186-73472896	-	-12669
5	74327065	74327316	252	74327178	12.20111	6.5755	7.01985	ENSG00000251041:intron;ENSG00000249343:intron	ENSG00000249343	74322485-74328297	+	4705
5	74363201	74363485	285	74363308	4.56295	3.60388	0.59373	intergenic	ENSG00000250446	74327994-74334766	-	-28576
5	75475244	75475513	270	75475485	4.16942	3.34931	0.52478	ENSG00000113163:intron	ENSG00000122008	75511755-75601144	+	-36377
5	78743547	78743824	278	78743643	5.24168	3.90698	1.06394	intergenic	ENSG00000254310	78753595-78753873	+	-9910
5	79085587	79085949	363	79085867	4.1849	3.24157	0.52478	ENSG00000132840:intron;ENSG00000132837:intron	ENSG00000132840	79069716-79089466	+	16051
5	82403773	82404076	304	82403905	4.31475	3.20475	0.59373	intergenic	ENSG00000205464	82279461-82386977	+	124463
5	83653751	83653995	245	83653890	8.26347	5.10236	3.46049	ENSG00000145681:intron	ENSG00000253073	83685865-83686018	+	-31992
5	84063088	84063539	452	84063290	3.77589	3.20345	0.30123	ENSG00000164176:intron	ENSG00000252861	84196882-84196989	+	-133569

5	87121409	87121627	219	87121567	4.74019	3.58891	0.73063	ENSG00000249061:intron	ENSG00000265919	87114878-87114954	-	-6563
5	89313729	89314122	394	89313960	15.566	7.77404	10.12097	ENSG00000248309:intron	ENSG00000250831	89466536-89470039	-	156114
5	89930717	89931010	294	89930876	8.134	4.66145	3.3598	intergenic	ENSG00000249265	89944909-89947573	+	-14046
5	90038092	90038350	259	90038251	4.07388	3.28775	0.47041	intergenic	ENSG00000264342	90016620-90016720	-	-21500
5	91280532	91281161	630	91280692	10.75085	5.53547	5.69375	ENSG00000234292:exon;ENSG00000248323:intron	ENSG00000234292	91280096-91281142	-	296
5	91311760	91312014	255	91311852	7.51885	4.83789	2.81499	ENSG00000248323:intron	ENSG00000248323	91054833-91314547	-	2660
5	92581467	92581738	272	92581594	3.77589	3.20345	0.30123	ENSG00000249776:intron	ENSG00000249169	92654847-92679141	+	-73245
5	92607279	92607561	283	92607299	3.77589	3.20345	0.30123	ENSG00000249776:intron	ENSG00000249169	92654847-92679141	+	-47427
5	92908026	92908320	295	92908152	13.86989	6.8073	8.5517	ENSG00000248528:intron	ENSG00000249958	92889386-92890840	-	-17332
5	94850701	94850942	242	94850863	4.56295	3.60388	0.59373	ENSG00000175471:intron	ENSG00000243806	94825960-94826694	-	-24127
5	95707778	95708157	380	95708120	3.63193	3.10837	0.30123	ENSG00000250240:intron	ENSG00000164292	95713521-95824383	+	-5554
5	96657125	96657408	284	96657274	11.66864	5.82681	6.52722	ENSG00000153113:intron	ENSG00000249180	96741078-96742698	-	85432
5	99027346	99027640	295	99027465	5.56876	3.96799	1.238	intergenic	ENSG00000241597	98954393-98954972	+	73099
5	99048252	99048457	206	99048291	4.56295	3.60388	0.59373	intergenic	ENSG00000241597	98954393-98954972	+	93961
5	99156262	99156673	412	99156333	4.56295	3.60388	0.59373	intergenic	ENSG00000241597	98954393-98954972	+	202074
5	100935458	100935782	325	100935604	4.07388	3.28775	0.47041	intergenic	ENSG00000113532	100806934-100903266	-	-32353
5	101680417	101680800	384	101680611	4.56295	3.60388	0.59373	intergenic	ENSG00000277506	101581829-101582128	+	98779
5	101925604	101926071	468	101925912	5.39424	4.00432	1.09512	intergenic	ENSG00000249495	101976295-101976798	+	-50458
5	102714953	102715176	224	102715052	4.56295	3.60388	0.59373	intergenic	ENSG00000145730	102753980-103031105	+	-38916
5	102826965	102827293	329	102827232	4.56295	3.60388	0.59373	intergenic	ENSG00000145730	102753980-103031105	+	73148
5	109318389	109318718	330	109318682	4.07388	3.28775	0.47041	ENSG00000249476:intron	ENSG00000249476	109237119-109326369	-	7816
5	115068885	115069121	237	115068974	10.55294	5.62938	5.52637	intergenic	ENSG00000250242	115087891-115088475	-	19472
5	115478353	115478596	244	115478567	4.03577	3.26323	0.47041	intergenic	ENSG00000249944	115489633-115489931	+	-11159
5	120457897	120458184	288	120458087	6.26578	4.40475	1.75945	intergenic	ENSG00000184838	120464277-120687332	+	-6237
5	120533486	120533744	259	120533528	3.03786	2.80302	0.15111	ENSG00000184838:intron	ENSG00000184838	120464277-120687332	+	69337
5	121625329	121625675	347	121625378	4.56295	3.60388	0.59373	intergenic	ENSG00000213661	121616246-121616807	+	9255
5	124512571	124512864	294	124512644	4.56295	3.60388	0.59373	ENSG00000248296:intron	ENSG00000248296	124492774-124536348	-	23631
5	124569273	124569503	231	124569341	5.39424	4.00432	1.09512	intergenic	ENSG00000248296	124492774-124536348	-	-33039
5	124583618	124584054	437	124583929	16.50093	7.50584	11.01119	intergenic	ENSG00000248296	124492774-124536348	-	-47487
5	124887418	124887628	211	124887503	6.26578	4.40475	1.75945	intergenic	ENSG00000249261	124868971-124869914	-	-17608
5	124902337	124902611	275	124902502	4.84177	3.65305	0.77787	ENSG00000260192:intron	ENSG00000249261	124868971-124869914	-	-32559
5	124952253	124952480	228	124952335	5.59518	3.83801	1.25935	ENSG00000260192:intron	ENSG00000249261	124868971-124869914	-	-82452
5	124967913	124968118	206	124968087	3.03786	2.80302	0.15111	ENSG00000260192:intron	ENSG00000249261	124868971-124869914	-	-98101
5	124887418	124887628	211	124887503	6.26578	4.40475	1.75945	intergenic	ENSG00000249261	124868971-124869914	-	-17608
5	124902337	124902611	275	124902502	4.84177	3.65305	0.77787	ENSG00000260192:intron	ENSG00000249261	124868971-124869914	-	-32559
5	124952253	124952480	228	124952335	5.59518	3.83801	1.25935	ENSG00000260192:intron	ENSG00000249261	124868971-124869914	-	-82452
5	124967913	124968118	206	124968087	3.03786	2.80302	0.15111	ENSG00000260192:intron	ENSG00000249261	124868971-124869914	-	-98101
5	124978578	124978821	244	124978786	3.658	3.12558	0.30123	ENSG00000260192:intron	ENSG00000250194	125074664-125074980	+	-95965
5	125245447	125245801	355	125245729	3.77589	3.20345	0.30123	ENSG00000260192:intron	ENSG00000250721	125265527-125265651	+	-19903
5	125961168	125961489	322	125961364	11.18335	6.21019	6.08588	intergenic	ENSG00000241888	125966776-125968085	-	6757
5	127677574	127677938	365	127677875	3.49438	2.91742	0.30123	intergenic	ENSG00000248799	127651692-127664029	-	-13726
5	130324090	130324384	295	130324229	6.51641	4.07877	1.96373	intergenic	ENSG00000252514	130386620-130386680	-	62443
5	131854312	131854551	240	131854351	3.53038	3.04135	0.30123	ENSG00000239642:intron;ENSG00000281938:intron	ENSG00000279584	131797414-131797929	+	57017
5	132020219	132020518	300	132020356	13.56604	6.40949	8.26891	intergenic	ENSG00000164398	131949972-132012243	-	-8125
5	135446752	135447012	261	135446977	5.6497	4.01836	1.29426	ENSG00000255833:three_prime_UTR;ENSG00000251380:exon;ENSG00000255833:exon	ENSG00000251380	135444213-135447348	-	466
5	137009378	137009688	311	137009559	6.26578	4.40475	1.75945	ENSG00000152377:intron	ENSG00000222285	136969199-136969306	-	-40226

5	137112912	137113254	343	137113221	3.34993	2.92244	0.30123	ENSG00000152377:intron	ENSG00000222285	136969199-136969306	-	-143776
5	137305078	137305443	366	137305215	4.56295	3.60388	0.59373	ENSG00000152377:intron	ENSG00000152377	136975297-137598379	-	293119
5	138340708	138340990	283	138340875	4.90328	3.55851	0.82412	ENSG00000120709:intron	ENSG00000158402	138285264-138338355	-	-2493
5	140461766	140462181	416	140461938	3.77589	3.20345	0.30123	ENSG00000131503:intron;ENSG00000254996:intron	ENSG00000254996	140401907-140549569	+	60066
5	140711046	140711408	363	140711163	14.24642	6.77521	8.90296	ENSG00000199990:Promoter	ENSG00000199990	140711274-140711373	+	-47
5	141259217	141259466	250	141259224	3.77589	3.20345	0.30123	ENSG00000277795:Promoter	ENSG00000277795	141259234-141261364	+	107
5	141864902	141865251	350	141865100	9.79137	5.02659	4.83571	ENSG00000156453:exon	ENSG00000156453	141853110-141879246	-	14170
5	141942503	141942779	277	141942593	3.6499	2.91341	0.30123	intergenic	ENSG00000271871	141952418-141953375	+	-9777
5	142191476	142191693	218	142191520	4.96535	3.7313	0.86205	intergenic	ENSG00000280047	142165766-142168387	+	25818
5	142223700	142223959	260	142223830	10.64217	4.64231	5.60051	intergenic	ENSG00000280047	142165766-142168387	+	58063
5	142191476	142191693	218	142191520	4.96535	3.7313	0.86205	intergenic	ENSG00000280047	142165766-142168387	+	25818
5	142223700	142223959	260	142223830	10.64217	4.64231	5.60051	intergenic	ENSG00000280047	142165766-142168387	+	58063
5	142191476	142191693	218	142191520	4.96535	3.7313	0.86205	intergenic	ENSG00000280047	142165766-142168387	+	25818
5	142223700	142223959	260	142223830	10.64217	4.64231	5.60051	intergenic	ENSG00000280047	142165766-142168387	+	58063
5	143009083	143009390	308	143009251	13.86989	6.8073	8.5517	ENSG00000145819:intron	ENSG00000285366	142946568-142947105	-	-62131
5	143052550	143052767	218	143052671	4.33895	3.45881	0.59373	ENSG00000145819:intron	ENSG00000285366	142946568-142947105	-	-105553
5	143009083	143009390	308	143009251	13.86989	6.8073	8.5517	ENSG00000145819:intron	ENSG00000285366	142946568-142947105	-	-62131
5	143052550	143052767	218	143052671	4.33895	3.45881	0.59373	ENSG00000145819:intron	ENSG00000285366	142946568-142947105	-	-105553
5	143262431	143262713	283	143262546	8.17554	4.86236	3.38452	intergenic	ENSG00000231579	143332604-143333239	+	-70032
5	143627271	143627513	243	143627318	4.56295	3.60388	0.59373	ENSG00000249881:intron	ENSG00000249881	143605627-143828772	+	21764
5	143733146	143733374	229	143733316	3.58061	3.07449	0.30123	ENSG00000249881:intron	ENSG00000225869	143752089-143752704	-	19444
5	144867861	144868163	303	144868013	4.42776	3.51628	0.59373	intergenic	ENSG00000249229	145001852-145005789	-	137777
5	145056934	145057180	247	145056985	7.17425	4.80518	2.52654	intergenic	ENSG00000249229	145001852-145005789	-	-51267
5	145086287	145086515	229	145086413	4.07388	3.28775	0.47041	intergenic	ENSG00000249229	145001852-145005789	-	-80611
5	144867861	144868163	303	144868013	4.42776	3.51628	0.59373	intergenic	ENSG00000249229	145001852-145005789	-	137777
5	145056934	145057180	247	145056985	7.17425	4.80518	2.52654	intergenic	ENSG00000249229	145001852-145005789	-	-51267
5	145086287	145086515	229	145086413	4.07388	3.28775	0.47041	intergenic	ENSG00000249229	145001852-145005789	-	-80611
5	146721577	146722064	488	146721662	5.39424	4.00432	1.09512	ENSG00000156475:intron	ENSG00000248568	146706380-146707600	-	-14220
5	147508089	147508303	215	147508160	5.56876	3.96799	1.238	ENSG00000113657:intron	ENSG00000113657	147390810-147510056	-	1860
5	147509198	147509579	382	147509225	3.88371	3.16559	0.37576	ENSG00000113657:intron	ENSG00000113657	147390810-147510056	-	668
5	147932609	147932862	254	147932729	4.1849	3.24157	0.52478	intergenic	ENSG00000250346	147922178-147923421	+	10557
5	148091047	148091321	275	148091122	5.73591	4.07212	1.35894	ENSG00000133710:intron	ENSG00000133710	148025682-148137289	+	65501
5	148228567	148228800	234	148228583	4.07388	3.28775	0.47041	ENSG00000248109:intron	ENSG00000248109	148221649-148243580	+	7034
5	148834753	148835163	411	148834891	5.3937	3.85943	1.09512	intergenic	ENSG00000169252	148825244-148828687	+	9713
5	149254918	149255189	272	149255106	3.6499	2.91341	0.30123	ENSG00000248647:intron;ENSG00000173210:intron;ENSG00000253406:intron	ENSG00000157510	149271870-149341802	+	-16817
5	150349541	150349772	232	150349660	3.92092	3.18945	0.4041	intergenic	ENSG00000070814	150357628-150400308	+	-7972
5	152382555	152382778	224	152382742	4.56295	3.60388	0.59373	intergenic	ENSG00000132911	152391531-152433368	-	50702
5	152453237	152453605	369	152453539	3.34993	2.92244	0.30123	intergenic	ENSG00000132911	152391531-152433368	-	-20052
5	153118148	153118729	582	153118553	6.26578	4.40475	1.75945	ENSG00000249484:intron	ENSG00000249484	152618964-153223543	-	105105
5	153253870	153254078	209	153253973	3.03786	2.80302	0.15111	intergenic	ENSG00000249484	152618964-153223543	-	-30430
5	153352684	153352894	211	153352769	5.87556	4.15944	1.46959	intergenic	ENSG00000249484	152618964-153223543	-	-129245
5	155933637	155933898	262	155933800	3.77589	3.20345	0.30123	ENSG00000170624:intron	ENSG00000170624	155870343-156767788	+	63424
5	156092065	156092340	276	156092218	3.77589	3.20345	0.30123	ENSG00000170624:intron	ENSG00000170624	155870343-156767788	+	221859
5	159021343	159021602	260	159021455	5.31032	3.95075	1.09512	ENSG00000164330:intron	ENSG00000253456	158985805-158987199	+	35667
5	159773308	159773646	339	159773477	29.47108	9.71099	23.55325	ENSG00000253311:intron;ENSG00000249738:intron	ENSG00000170214	159865079-159972544	+	-91602
5	159826438	159826738	301	159826608	14.83338	6.62465	9.44274	ENSG00000253311:intron	ENSG00000170214	159865079-159972544	+	-38491

5	159773308	159773646	339	159773477	29.47108	9.71099	23.55325	ENSG00000253311:intron;ENSG00000249738:intron	ENSG00000170214	159865079-159972544	+	-91602
5	159826438	159826738	301	159826608	14.83338	6.62465	9.44274	ENSG00000253311:intron	ENSG00000170214	159865079-159972544	+	-38491
5	162320276	162320521	246	162320446	5.39424	4.00432	1.09512	intergenic	ENSG00000201474	162477890-162477993	+	-157492
5	163679134	163679365	232	163679244	4.56295	3.60388	0.59373	intergenic	ENSG00000224012	163709209-163709514	-	30265
5	166904710	166904934	225	166904829	4.56295	3.60388	0.59373	intergenic	ENSG00000254130	166905221-166926370	-	21548
5	166943885	166944131	247	166943966	3.77589	3.20345	0.30123	intergenic	ENSG00000254130	166905221-166926370	-	-17637
5	166987701	166988014	314	166987832	3.77589	3.20345	0.30123	intergenic	ENSG00000254130	166905221-166926370	-	-61487
5	168116054	168116293	240	168116106	4.07388	3.28775	0.47041	ENSG00000253925:intron;ENSG00000145934:intron	ENSG00000279739	168128855-168130422	+	-12682
5	169063035	169063266	232	169063118	4.84177	3.65305	0.77787	ENSG00000184347:intron	ENSG00000248222	169013226-169037998	+	49924
5	171231421	171231690	270	171231558	5.39424	4.00432	1.09512	ENSG00000204764:intron	ENSG00000253213	171251865-171252982	+	-20310
5	174473581	174473922	342	174473771	6.26578	4.40475	1.75945	ENSG00000249306:intron	ENSG00000213376	174513304-174514307	+	-39553
5	177454619	177454847	229	177454709	7.44723	3.75648	2.74965	ENSG00000131188:intron	ENSG00000246334	177438502-177447699	-	-7033
5	179158546	179158858	313	179158591	3.93344	3.19748	0.41301	ENSG00000087116:intron	ENSG00000253144	179082679-179083194	-	-75507
5	179392125	179392469	345	179392269	3.77589	3.20345	0.30123	intergenic	ENSG00000253617	179385818-179385992	-	-6304
6	154265	154674	410	154486	5.74381	3.92704	1.36222	intergenic	ENSG00000217929	131909-145083	-	-9386
6	1797102	1797450	349	1797411	4.06198	3.28009	0.47041	ENSG00000112699:intron	ENSG00000054598	1609971-1613897	+	187304
6	1831027	1831299	273	1831084	3.49438	2.91742	0.30123	ENSG00000112699:intron	ENSG00000054598	1609971-1613897	+	221191
6	4166159	4166414	256	4166171	4.06198	3.28009	0.47041	intergenic	ENSG00000237716	4186623-4188109	-	21823
6	4682593	4682823	231	4682721	4.1849	3.24157	0.52478	intergenic	ENSG00000217385	4702462-4703779	+	-19754
6	5425510	5425885	376	5425560	4.48857	3.55567	0.59373	ENSG00000145982:intron	ENSG00000269985	5451682-5458075	-	32378
6	5588649	5588887	239	5588876	3.76336	3.08858	0.30123	ENSG00000145982:intron	ENSG00000218574	5609226-5610195	-	21427
6	6124960	6125220	261	6125064	4.55072	3.59595	0.59373	intergenic	ENSG00000283616	6169333-6169409	+	-44243
6	6138486	6138915	430	6138606	6.26578	4.40475	1.75945	intergenic	ENSG00000283616	6169333-6169409	+	-30633
6	6681181	6681523	343	6681345	4.81345	3.63515	0.77787	ENSG00000261211:exon	ENSG00000261211	6680308-6683633	-	2281
6	6733713	6733990	278	6733935	4.62651	3.51732	0.63659	ENSG00000226281:intron	ENSG00000226281	6692743-6739030	-	5179
6	7360493	7360774	282	7360604	4.07388	3.28775	0.47041	ENSG00000164304:intron	ENSG00000124783	7268305-7347446	-	-13187
6	7963217	7963639	423	7963461	46.3087	16.17968	39.98943	ENSG00000259040:intron	ENSG00000219294	7986536-7988192	+	-23108
6	12017954	12018202	249	12018067	3.37418	2.93841	0.30123	ENSG00000095951:intron	ENSG00000095951	12008761-12164999	+	9316
6	12051796	12052090	295	12051944	6.8972	4.14041	2.29209	ENSG00000095951:intron	ENSG00000095951	12008761-12164999	+	43181
6	15138347	15138672	326	15138524	4.56295	3.60388	0.59373	intergenic	ENSG00000242989	15112967-15113221	+	25542
6	16731701	16731961	261	16731821	8.45558	5.02538	3.63629	ENSG00000124788:intron	ENSG00000229931	16761137-16762652	+	-29306
6	17394110	17394372	263	17394257	4.91809	3.10693	0.82412	ENSG00000112186:intron	ENSG00000112186	17393215-17557792	+	1025
6	19474717	19474976	260	19474827	3.77589	3.20345	0.30123	ENSG00000228412:intron	ENSG00000201523	19438282-19438418	+	36564
6	19494700	19495385	686	19495154	5.39424	4.00432	1.09512	ENSG00000228412:intron	ENSG00000201523	19438282-19438418	+	56760
6	20162422	20162637	216	20162594	5.56876	3.96799	1.238	ENSG00000172197:intron	ENSG00000227803	20212086-20317739	+	-49557
6	20638364	20638614	251	20638441	5.24168	3.90698	1.06394	ENSG00000145996:intron	ENSG00000225832	20722327-20723655	-	85166
6	21217525	21217796	272	21217741	4.84177	3.65305	0.77787	ENSG00000145996:intron	ENSG00000227089	21354410-21355296	-	137636
6	24244697	24244918	222	24244836	4.62651	3.51732	0.63659	ENSG00000146038:intron	ENSG00000251830	24166272-24166374	+	78535
6	24976733	24976951	219	24976837	5.6497	4.01836	1.29426	ENSG00000214975:exon;ENSG00000111913:intron	ENSG00000214975	24976418-24976982	+	423
6	25250887	25251136	250	25251034	8.17554	4.86236	3.38452	ENSG00000168405:intron	ENSG00000271108	25248262-25248779	+	2749
6	25315561	25315856	296	25315756	3.76336	3.08858	0.30123	ENSG00000079691:intron;ENSG00000168405:intron	ENSG00000207286	25287431-25287533	-	-28175
6	25587179	25587394	216	25587264	3.17405	2.80686	0.2494	ENSG00000079691:intron	ENSG00000079689	25652200-25701783	+	-64914
6	26553734	26554094	361	26553952	6.33349	4.28489	1.823	intergenic	ENSG00000284607	26569323-26574698	+	-15409
6	28084642	28084869	228	28084851	3.69765	3.15177	0.30123	ENSG00000197279:intron	ENSG00000272009	28078791-28081130	-	-3625
6	28151551	28151875	325	28151644	3.77589	3.20345	0.30123	ENSG00000198315:intron	ENSG00000198315	28141909-28159472	+	9803
6	29507254	29507500	247	29507499	3.02441	2.62206	0.15111	ENSG00000229274:exon	ENSG00000229274	29497508-29510556	+	9868

6	30259788	30260086	299	30259956	4.70801	3.44018	0.70461	ENSG00000270604:intron;ENSG00000243753:exon	ENSG00000243753	30259583-30293014	+	353
6	31034687	31035026	340	31034800	4.56295	3.60388	0.59373	ENSG00000261272:exon	ENSG00000228789	31053449-31059890	+	-18593
6	31198002	31198323	322	31198103	3.6499	2.91341	0.30123	ENSG00000206344:intron;ENSG00000272501:Promoter	ENSG00000272501	31195199-31198037	-	-125
6	31734639	31734920	282	31734817	8.04467	4.29601	3.29781	ENSG00000213719:intron	ENSG00000213722	31727037-31730617	-	-4162
6	31777034	31777374	341	31777169	5.96733	3.52084	1.54405	ENSG00000204396:exon;ENSG00000204396:five_prime_UTR	ENSG00000204396	31765589-31777294	-	90
6	32299655	32299934	280	32299693	4.56295	3.60388	0.59373	ENSG00000204296:intron;ENSG00000225914:intron	ENSG00000237285	32325218-32326178	+	-25424
6	32832360	32832628	269	32832420	5.15789	3.85362	1.00126	ENSG00000204267:exon;ENSG00000250264:exon	ENSG00000204267	32821832-32838780	-	6286
6	34224089	34224334	246	34224192	4.84975	2.89864	0.78358	intergenic	ENSG00000214810	34219438-34220066	+	4773
6	34485015	34485298	284	34485153	8.95304	4.76203	4.09227	ENSG00000124507:intron	ENSG00000124507	34466060-34535231	+	19096
6	34811226	34811538	313	34811447	6.86122	4.43968	2.2645	ENSG00000065060:intron	ENSG00000206717	34821444-34821542	+	-10062
6	35777321	35777544	224	35777460	5.66809	4.02982	1.31021	ENSG00000196748:exon	ENSG00000196748	35776593-35779552	+	839
6	35982150	35982387	238	35982347	4.35972	3.35026	0.59373	ENSG00000112053:intron	ENSG00000271304	35989514-35991374	-	9106
6	36336582	36336802	221	36336683	5.56876	3.96799	1.238	ENSG00000189325:intron	ENSG00000189325	36315756-36336885	-	193
6	36975311	36975574	264	36975383	6.06008	4.11811	1.6243	ENSG00000137409:intron	ENSG00000137409	36968140-36986298	-	10856
6	37024670	37024952	283	37024811	11.66864	5.82681	6.52722	ENSG00000146192:intron	ENSG00000146192	37005645-37029070	+	19165
6	37048748	37048990	243	37048847	11.73403	6.07685	6.5894	intergenic	ENSG00000226976	37044859-37045189	+	4009
6	37471720	37471946	227	37471773	3.51799	3.03318	0.30123	ENSG00000137200:intron	ENSG00000198937	37482919-37499922	-	28089
6	39530194	39530432	239	39530319	8.06337	4.79751	3.31375	ENSG00000164627:intron	ENSG00000231276	39553810-39554943	+	-23497
6	40258357	40258589	233	40258529	5.75307	4.08283	1.36357	intergenic	ENSG00000227131	40271565-40276237	-	17764
6	41938966	41939192	227	41939087	4.17432	3.23501	0.52478	ENSG00000112576:intron	ENSG00000112578	41921187-41933046	+	17891
6	42514673	42514933	261	42514825	7.17425	4.80518	2.52654	intergenic	ENSG00000237107	42499709-42500022	+	15093
6	42630939	42631241	303	42630992	4.04884	3.27164	0.47041	ENSG00000024048:intron	ENSG00000206848	42664161-42664268	+	-33071
6	43767882	43768097	216	43767939	1.80642	1.97206	0.08775	intergenic	ENSG00000112715	43770183-43786487	+	-2194
6	43798958	43799445	488	43799096	6.51641	4.07877	1.96373	intergenic	ENSG00000283573	43803192-43842625	+	-3991
6	45709937	45710156	220	45710008	7.11644	3.84111	2.49147	intergenic	ENSG00000261080	45573345-45576770	-	-133276
6	45830326	45830846	521	45830717	5.75051	3.78743	1.36222	intergenic	ENSG00000212468	46018744-46018851	+	-188158
6	46853947	46854217	271	46854113	4.56295	3.60388	0.59373	ENSG00000069122:intron	ENSG00000225730	46903470-46909078	+	-49388
6	47499166	47499400	235	47499286	6.26578	4.40475	1.75945	ENSG00000198087:intron	ENSG00000199762	47489066-47489170	+	10216
6	51660848	51661130	283	51660966	5.6497	4.01836	1.29426	ENSG00000170927:intron	ENSG00000228689	51599722-51623428	+	61266
6	51780997	51781218	222	51781099	3.77589	3.20345	0.30123	ENSG00000170927:intron	ENSG00000228689	51599722-51623428	+	181385
6	52227633	52227877	245	52227739	8.85591	4.88357	4.00947	intergenic	ENSG00000112116	52236680-52244537	-	16782
6	52390076	52390732	657	52390599	7.84387	5.03722	3.11593	ENSG00000170915:intron;ENSG00000096093:intron	ENSG00000096093	52362122-52529886	+	28281
6	52897987	52898265	279	52898127	6.15301	4.33368	1.70124	ENSG00000174156:intron	ENSG00000174156	52896638-52909685	-	11559
6	61494632	61494915	284	61494640	4.07388	3.28775	0.47041	intergenic	ENSG00000255633	61574327-61574402	+	-79554
6	61538970	61539188	219	61539057	4.70801	3.44018	0.70461	intergenic	ENSG00000255633	61574327-61574402	+	-35248
6	64154155	64154433	279	64154242	4.56295	3.60388	0.59373	ENSG00000188107:intron	ENSG00000232120	64377794-64412779	+	-223500
6	69467207	69467414	208	69467300	3.03786	2.80302	0.15111	intergenic	ENSG00000219085	69705286-69706160	-	238850
6	69490731	69491062	332	69490992	4.56295	3.60388	0.59373	intergenic	ENSG00000219085	69705286-69706160	-	215264
6	69763862	69764115	254	69763993	4.56295	3.60388	0.59373	ENSG00000168216:intron	ENSG00000219881	69745870-69746851	+	18118
6	71788617	71788871	255	71788683	4.07388	3.28775	0.47041	intergenic	ENSG00000079841	71886702-72403143	+	-97958
6	80503793	80504040	248	80503913	4.56295	3.60388	0.59373	intergenic	ENSG00000181705	80499195-80499392	-	-4524
6	81927771	81928030	260	81927939	4.84177	3.65305	0.77787	ENSG00000226453:intron	ENSG00000226453	81845184-81933480	-	5580
6	82410327	82410549	223	82410480	5.22474	3.89619	1.05478	intergenic	ENSG00000146242	82363205-82370828	+	47232
6	82450987	82451203	217	82451111	3.77589	3.20345	0.30123	intergenic	ENSG00000146242	82363205-82370828	+	87889

6	83507872	83508087	216	83507955	3.18471	2.81386	0.2494	intergenic	ENSG00000146250	83512537-83525704	+	-4558
6	83532368	83532584	217	83532497	3.03786	2.80302	0.15111	intergenic	ENSG00000146250	83512537-83525704	+	19938
6	84090279	84090518	240	84090483	3.77589	3.20345	0.30123	ENSG00000135324:three_prime_UTR;ENSG00000135324:exon	ENSG00000135324	84033755-84090881	+	56643
6	84561083	84561293	211	84561257	4.56295	3.60388	0.59373	intergenic	ENSG00000231776	84421027-84556152	-	-5035
6	84575341	84575688	348	84575535	7.34679	4.23292	2.66603	intergenic	ENSG00000231776	84421027-84556152	-	-19362
6	85490202	85490468	267	85490336	5.24168	3.90698	1.06394	ENSG00000135318:intron	ENSG00000280232	85498440-85499058	-	8723
6	85524425	85524780	356	85524752	3.98411	3.23002	0.45207	ENSG00000135317:intron	ENSG00000280232	85498440-85499058	-	-25544
6	87032730	87033045	316	87032897	3.77589	3.20345	0.30123	intergenic	ENSG00000217169	87000044-87000291	-	-32596
6	88348158	88348650	493	88348461	5.38044	3.99551	1.09512	ENSG00000234426:intron	ENSG00000220267	88275881-88276989	-	-71414
6	88650295	88650560	266	88650399	12.20111	6.5755	7.01985	ENSG00000111880:intron	ENSG00000222145	88714241-88714430	-	64003
6	89298070	89298304	235	89298165	8.95304	4.76203	4.09227	ENSG00000111886:intron	ENSG00000220418	89301782-89303114	+	-3595
6	89691105	89691325	221	89691146	3.23478	2.75364	0.28314	ENSG00000112159:intron	ENSG00000228124	89673468-89675783	+	17746
6	90369012	90369351	340	90369196	20.26942	7.93672	14.62399	intergenic	ENSG00000260271	90320442-90362632	+	48739
6	90479099	90479518	420	90479371	4.76929	3.60726	0.74587	intergenic	ENSG00000135341	90513572-90587045	-	107737
6	90892272	90892525	254	90892313	3.77589	3.20345	0.30123	intergenic	ENSG00000135341	90513572-90587045	-	-305353
6	92709951	92710370	420	92710226	13.26553	7.20777	7.98923	intergenic	ENSG00000220908	92723002-92723829	-	13669
6	98561244	98561486	243	98561331	3.77589	3.20345	0.30123	intergenic	ENSG00000187472	98780256-98780505	-	219140
6	98777834	98778081	248	98777960	11.46477	6.37695	6.34551	intergenic	ENSG00000187472	98780256-98780505	-	2548
6	99263691	99263909	219	99263857	4.49014	3.43176	0.59373	intergenic	ENSG00000146267	99271168-99350062	-	86262
6	99606476	99607016	541	99606660	4.10177	3.30571	0.49129	ENSG00000112238:Promoter	ENSG00000112238	99606729-99615578	+	16
6	105646401	105646814	414	105646632	4.29558	3.43077	0.59373	intergenic	ENSG00000237567	105612666-105632196	-	-14411
6	105757530	105758087	558	105757656	15.53994	6.99217	10.09573	ENSG00000284999:intron	ENSG00000219088	105666325-105667998	+	91483
6	105787267	105787503	237	105787351	6.6688	3.87655	2.09177	ENSG00000284999:intron	ENSG00000284999	105679377-105893041	-	105656
6	106106412	106106628	217	106106549	6.31582	4.27407	1.80607	ENSG00000057657:intron;ENSG00000057663:intron	ENSG00000269919	106100139-106100593	+	6380
6	106145738	106145988	251	106145892	6.20936	3.52525	1.75105	ENSG00000057663:intron	ENSG00000269919	106100139-106100593	+	45723
6	106106412	106106628	217	106106549	6.31582	4.27407	1.80607	ENSG00000057657:intron;ENSG00000057663:intron	ENSG00000269919	106100139-106100593	+	6380
6	106145738	106145988	251	106145892	6.20936	3.52525	1.75105	ENSG00000057663:intron	ENSG00000269919	106100139-106100593	+	45723
6	107052134	107052394	261	107052298	5.24168	3.90698	1.06394	intergenic	ENSG00000130349	107028212-107051342	+	24051
6	107658009	107658282	274	107658141	8.17554	4.86236	3.38452	ENSG00000112320:intron	ENSG00000280135	107697298-107700218	-	42073
6	108169610	108169947	338	108169781	3.20396	2.64557	0.26167	ENSG00000112333:intron	ENSG00000112333	108166057-108188809	+	3721
6	108564265	108564583	319	108564412	16.73013	7.13482	11.23059	ENSG00000118689:intron	ENSG00000118689	108559834-108684774	+	4589
6	108755190	108755699	510	108755643	5.45233	3.75292	1.13858	ENSG00000203801:intron	ENSG00000203801	108751653-108769942	+	3791
6	109015381	109015604	224	109015523	4.1849	3.24157	0.52478	ENSG00000080546:intron	ENSG00000271730	108998481-108999125	+	17011
6	109204034	109204295	262	109204060	3.49438	2.91742	0.30123	ENSG00000203799:intron	ENSG00000203799	109165830-109355063	+	38334
6	113632425	113632635	211	113632489	4.31475	3.20475	0.59373	ENSG00000230943:intron	ENSG00000230943	113616926-113650090	-	17560
6	113690739	113690985	247	113690861	9.09099	5.60604	4.18865	intergenic	ENSG00000230943	113616926-113650090	-	-40771
6	113991751	113992017	267	113991826	4.32442	3.44941	0.59373	ENSG00000196591:intron;ENSG00000228624:intron	ENSG00000196591	113933027-114011308	-	19424
6	114162505	114162767	263	114162613	7.17425	4.80518	2.52654	ENSG00000249853:intron;ENSG00000228624:intron	ENSG00000212333	114220680-114220786	-	58150
6	114169365	114169694	330	114169438	4.56295	3.60388	0.59373	ENSG00000249853:intron;ENSG00000228624:intron	ENSG00000212333	114220680-114220786	-	51257
6	114162505	114162767	263	114162613	7.17425	4.80518	2.52654	ENSG00000249853:intron;ENSG00000228624:intron	ENSG00000212333	114220680-114220786	-	58150
6	114169365	114169694	330	114169438	4.56295	3.60388	0.59373	ENSG00000249853:intron;ENSG00000228624:intron	ENSG00000212333	114220680-114220786	-	51257

6	117545222	117545472	251	117545408	5.73591	4.07212	1.35894	ENSG00000164465:intron;ENSG00000282218:intron	ENSG00000282218	117318210-117573571	-	28224
6	117692230	117692530	301	117692390	5.34519	3.973	1.09512	ENSG00000153989:intron	ENSG00000153989	117675501-117710640	+	16878
6	117730267	117730477	211	117730381	3.03786	2.80302	0.15111	intergenic	ENSG00000153989	117675501-117710640	+	54870
6	119147382	119147725	344	119147601	15.38923	7.67141	9.96025	ENSG00000253194:intron;ENSG00000111879:intron	ENSG00000111879	118959762-119149387	-	1834
6	128271257	128271513	257	128271285	4.31475	3.20475	0.59373	ENSG00000152894:intron	ENSG00000224733	128500526-128501176	+	-229141
6	129490525	129491263	739	129491120	18.62188	8.98605	13.04335	ENSG00000196569:intron	ENSG00000233351	129479614-129481410	-	-9483
6	129684045	129684321	277	129684106	6.51641	4.07877	1.96373	ENSG00000146376:intron	ENSG00000146376	129576131-129710225	-	26042
6	129686919	129687207	289	129687082	7.96683	4.92425	3.22527	ENSG00000146376:intron	ENSG00000146376	129576131-129710225	-	23162
6	129684045	129684321	277	129684106	6.51641	4.07877	1.96373	ENSG00000146376:intron	ENSG00000146376	129576131-129710225	-	26042
6	129686919	129687207	289	129687082	7.96683	4.92425	3.22527	ENSG00000146376:intron	ENSG00000146376	129576131-129710225	-	23162
6	129816577	129816825	249	129816763	3.49438	2.91742	0.30123	intergenic	ENSG00000219302	129819636-129820170	-	3469
6	130517411	130517696	286	130517570	10.34847	5.72262	5.33114	intergenic	ENSG00000202438	130573995-130574112	-	56559
6	130588271	130588547	277	130588421	4.56295	3.60388	0.59373	intergenic	ENSG00000202438	130573995-130574112	-	-14296
6	130517411	130517696	286	130517570	10.34847	5.72262	5.33114	intergenic	ENSG00000202438	130573995-130574112	-	56559
6	130588271	130588547	277	130588421	4.56295	3.60388	0.59373	intergenic	ENSG00000202438	130573995-130574112	-	-14296
6	133270248	133270791	544	133270454	4.84177	3.65305	0.77787	ENSG00000112319:intron	ENSG00000112319	133240597-133532120	+	29922
6	134236052	134236303	252	134236223	8.18881	4.69185	3.39457	ENSG00000118515:intron	ENSG00000200058	134257034-134257143	+	-20857
6	134247176	134247402	227	134247257	5.37821	3.84986	1.09512	ENSG00000118515:intron	ENSG00000200058	134257034-134257143	+	-9745
6	134236052	134236303	252	134236223	8.18881	4.69185	3.39457	ENSG00000118515:intron	ENSG00000200058	134257034-134257143	+	-20857
6	134247176	134247402	227	134247257	5.37821	3.84986	1.09512	ENSG00000118515:intron	ENSG00000200058	134257034-134257143	+	-9745
6	134464952	134465342	391	134465114	3.56683	2.68164	0.30123	ENSG00000236700:intron;ENSG00000231971:intron	ENSG00000236700	134437715-134504581	+	27431
6	137811440	137811682	243	137811528	5.13473	3.43925	0.98202	intergenic	ENSG00000234956	137730169-137792835	-	-18725
6	137866868	137867087	220	137866975	6.79868	3.38039	2.20551	ENSG00000118503:Promoter:ENSG00000237499:intron	ENSG00000118503	137867187-137883312	+	-210
6	139188498	139188856	359	139188750	5.75051	3.78743	1.36222	ENSG00000231329:intron;ENSG00000272446:intron	ENSG00000231329	139144203-139239653	-	50976
6	139764706	139764994	289	139764873	7.3143	4.5382	2.6406	ENSG00000231426:intron	ENSG00000231426	139677638-139860476	+	87211
6	140198955	140199311	357	140199192	4.56295	3.60388	0.59373	intergenic	ENSG00000263514	140205251-140205326	+	-6118
6	140329951	140330191	241	140330080	4.458	3.53587	0.59373	intergenic	ENSG00000263514	140205251-140205326	+	124819
6	140385547	140385804	258	140385640	4.56295	3.60388	0.59373	intergenic	ENSG00000263514	140205251-140205326	+	180424
6	140744951	140745297	347	140745120	17.55853	7.81859	12.02348	ENSG00000285875:intron	ENSG00000217684	140761528-140762262	+	-16404
6	140774284	140774520	237	140774408	11.01631	5.89057	5.93639	ENSG00000285875:intron	ENSG00000217684	140761528-140762262	+	12873
6	140744951	140745297	347	140745120	17.55853	7.81859	12.02348	ENSG00000285875:intron	ENSG00000217684	140761528-140762262	+	-16404
6	140774284	140774520	237	140774408	11.01631	5.89057	5.93639	ENSG00000285875:intron	ENSG00000217684	140761528-140762262	+	12873
6	158207480	158207755	276	158207619	13.26553	7.20777	7.98923	intergenic	ENSG00000130338	158232235-158511828	+	-24618
6	158869802	158870080	279	158869920	9.06429	5.18652	4.17525	ENSG00000203711:Promoter	ENSG00000203711	158869938-158919105	+	2
6	159979206	159979490	285	159979345	6.26578	4.40475	1.75945	ENSG00000197081:intron	ENSG00000197081	159969098-160113507	+	10249
6	161446740	161447037	298	161446911	5.39424	4.00432	1.09512	ENSG00000185345:intron	ENSG00000026652	161129978-161274061	-	-172827
7	831798	832047	250	831926	8.134	4.66145	3.3598	ENSG00000164828:intron	ENSG00000164828	816614-896435	+	15308
7	1104356	1104574	219	1104541	5.40925	3.86905	1.10135	ENSG00000146540:intron	ENSG00000164850	1082207-1093815	+	22257
7	1406753	1407015	263	1406943	5.66875	3.88202	1.31047	intergenic	ENSG00000164877	1428464-1459502	-	52618
7	2723109	2723360	252	2723170	4.26704	3.41232	0.58491	ENSG00000174945:intron	ENSG00000174945	2679521-2775500	+	43713
7	2757963	2758247	285	2758062	8.8685	5.46977	4.02102	ENSG00000174945:intron;ENSG00000146535:intron	ENSG00000231357	2835620-2836022	+	-77515
7	2946531	2946766	236	2946686	4.48857	3.55567	0.59373	ENSG00000237286:exon;ENSG00000198286:intron	ENSG00000237286	2944034-2947091	+	2614
7	3422348	3422567	220	3422490	5.39424	4.00432	1.09512	ENSG00000146555:intron	ENSG00000224593	3337888-3338601	+	84569
7	3482163	3482440	278	3482354	4.1849	3.24157	0.52478	ENSG00000146555:intron	ENSG00000224593	3337888-3338601	+	144413

7	6990586	6990789	204	6990616	5.0924	3.81196	0.95207	intergenic	ENSG00000242731	6931683-6939603	-	-51084
7	7945128	7945348	221	7945263	6.47492	3.91084	1.94623	ENSG00000283549:intron;ENSG00000219545:intron	ENSG00000234141	7949852-7950709	-	5471
7	13849985	13850419	435	13850294	10.79066	5.97945	5.72727	intergenic	ENSG00000224330	13854354-13859025	-	8823
7	14687276	14687559	284	14687433	9.59377	5.70253	4.65976	ENSG00000136267:intron	ENSG00000226323	14814130-14816565	+	-126713
7	20960579	20960846	268	20960759	4.31475	3.20475	0.59373	ENSG00000232790:intron;ENSG00000285592:intron	ENSG00000285592	20959390-21028813	-	68101
7	21095009	21095333	325	21095218	4.56295	3.60388	0.59373	intergenic	ENSG00000285592	20959390-21028813	-	-66357
7	20960579	20960846	268	20960759	4.31475	3.20475	0.59373	ENSG00000232790:intron;ENSG00000285592:intron	ENSG00000285592	20959390-21028813	-	68101
7	21095009	21095333	325	21095218	4.56295	3.60388	0.59373	intergenic	ENSG00000285592	20959390-21028813	-	-66357
7	21667819	21668046	228	21667883	4.919	3.70193	0.82412	ENSG00000105877:intron	ENSG00000105877	21543214-21901839	+	124718
7	24036547	24036781	235	24036602	5.2079	3.88546	1.04111	intergenic	ENSG00000252041	24132704-24132837	+	-96040
7	24290205	24290552	348	24290218	4.07388	3.28775	0.47041	ENSG00000122585:intron	ENSG00000122585	24284162-24291865	+	6216
7	24383504	24383728	225	24383598	3.03786	2.80302	0.15111	intergenic	ENSG00000122585	24284162-24291865	+	99453
7	24821154	24821727	574	24821539	10.76521	5.96458	5.70713	ENSG00000070882:intron	ENSG00000105928	24698350-24758113	-	-63327
7	25031016	25031261	246	25031152	5.50544	3.52	1.18795	intergenic	ENSG00000070882	24796538-24981634	-	-49504
7	25125445	25125702	258	25125544	8.99866	4.4694	4.12107	ENSG00000172115:Promoter	ENSG00000172115	25120090-25125361	-	-212
7	25964524	25964775	252	25964666	9.69963	5.35224	4.76095	intergenic	ENSG00000199085	25949918-25949986	-	-14663
7	26613722	26613981	260	26613828	5.17447	3.86417	1.0143	intergenic	ENSG00000222004	26637870-26647305	+	-24019
7	26666706	26666934	229	26666802	5.39424	4.00432	1.09512	intergenic	ENSG00000222004	26637870-26647305	+	28949
7	26744662	26744894	233	26744801	4.56295	3.60388	0.59373	ENSG00000005020:intron	ENSG00000222004	26637870-26647305	+	106907
7	28207415	28207723	309	28207589	6.49439	4.38367	1.95029	ENSG00000234336:intron	ENSG00000153814	27830572-28180743	-	-26825
7	30778781	30779020	240	30778930	4.10177	3.30571	0.49129	ENSG00000106125:intron;ENSG00000254959:intron	ENSG00000106125	30771416-30892387	+	7484
7	30984412	30984667	256	30984611	5.39424	4.00432	1.09512	intergenic	ENSG00000106128	30938668-30993254	+	45871
7	34375209	34375432	224	34375340	3.53832	2.94528	0.30123	ENSG00000197085:intron	ENSG00000202431	34297495-34297598	-	-77722
7	36119717	36119999	283	36119819	6.79012	4.3971	2.19778	intergenic	ENSG00000228815	36109023-36110872	-	-8985
7	36966900	36967117	218	36967054	4.07388	3.28775	0.47041	ENSG00000155849:intron	ENSG00000224101	36997795-37013630	+	-30787
7	37032321	37032682	362	37032411	3.24979	2.85659	0.29383	ENSG00000155849:intron	ENSG00000224101	36997795-37013630	+	34706
7	37709996	37710240	245	37710201	5.31032	3.95075	1.09512	ENSG00000187037:intron;ENSG00000086289:intron	ENSG00000086289	37683842-37951941	+	26275
7	38694944	38695150	207	38694990	4.56295	3.60388	0.59373	intergenic	ENSG00000164556	38685345-38687037	-	-8009
7	38798575	38798926	352	38798684	5.39424	4.00432	1.09512	ENSG00000006715:intron	ENSG00000164556	38685345-38687037	-	-111713
7	39105651	39106031	381	39105787	4.07388	3.28775	0.47041	ENSG00000106536:intron	ENSG00000233854	38984242-39013551	-	-92289
7	42168829	42169051	223	42168921	3.77589	3.20345	0.30123	ENSG00000106571:intron	ENSG00000213721	42113364-42113620	+	55575
7	42551017	42551236	220	42551172	3.77589	3.20345	0.30123	intergenic	ENSG00000238284	42661725-42706447	-	155321
7	42704536	42704855	320	42704731	4.42776	3.51628	0.59373	ENSG00000238284:intron	ENSG00000238284	42661725-42706447	-	1752
7	42951787	42952042	256	42951900	3.77589	3.20345	0.30123	intergenic	ENSG00000106588	42916856-42932223	-	-19691
7	43072409	43072688	280	43072576	4.1849	3.24157	0.52478	ENSG00000232006:intron	ENSG00000201933	43096731-43096844	+	-24183
7	43318794	43319200	407	43319016	4.56295	3.60388	0.59373	ENSG00000002746:intron	ENSG00000213717	43274158-43274582	+	44838
7	43322811	43323034	224	43322924	5.6497	4.01836	1.29426	ENSG00000002746:intron	ENSG00000213717	43274158-43274582	+	48764
7	44639263	44639494	232	44639416	11.87429	5.03049	6.72258	ENSG00000105953:intron	ENSG00000105953	44606571-44709066	+	32807
7	47475240	47475464	225	47475334	5.55463	3.31788	1.22984	ENSG00000136205:intron	ENSG00000136205	47275153-47582558	-	107206
7	47669576	47669814	239	47669686	13.87404	4.9901	8.55472	intergenic	ENSG00000221845	47655243-47661648	+	14451
7	48699582	48699925	344	48699874	4.56295	3.60388	0.59373	intergenic	ENSG00000285536	48708326-48711582	+	-8573
7	49349963	49350538	576	49350409	4.56295	3.60388	0.59373	intergenic	ENSG00000270763	49258492-49259846	-	-90404
7	49488645	49488853	209	49488803	5.39424	4.00432	1.09512	intergenic	ENSG00000270763	49258492-49259846	-	-228902
7	51997699	51998180	482	51997888	3.36259	2.93078	0.30123	intergenic	ENSG00000233960	52165234-52192913	-	194974
7	52124916	52125123	208	52125038	3.03786	2.80302	0.15111	intergenic	ENSG00000233960	52165234-52192913	-	67894
7	52714718	52714944	227	52714886	3.77589	3.20345	0.30123	intergenic	ENSG00000234304	52891836-52897807	-	182976

7	52741138	52741360	223	52741140	3.02441	2.62206	0.15111	intergenic	ENSG00000234304	52891836-52897807	-	156558
7	52714718	52714944	227	52714886	3.77589	3.20345	0.30123	intergenic	ENSG00000234304	52891836-52897807	-	182976
7	52741138	52741360	223	52741140	3.02441	2.62206	0.15111	intergenic	ENSG00000234304	52891836-52897807	-	156558
7	55120824	55121124	301	55121047	4.56295	3.60388	0.59373	ENSG00000146648:intron	ENSG00000224057	55179749-55188934	-	67960
7	55127170	55127412	243	55127236	4.84177	3.65305	0.77787	ENSG00000146648:intron	ENSG00000224057	55179749-55188934	-	61643
7	55533423	55533626	204	55533473	4.92533	3.11051	0.829	ENSG00000154978:intron	ENSG00000154978	55436055-55572988	-	39464
7	55534327	55534734	408	55534503	25.86516	7.9306	20.03412	ENSG00000154978:intron	ENSG00000154978	55436055-55572988	-	38458
7	55945205	55945448	244	55945324	4.76929	3.60726	0.74587	ENSG00000249773:intron	ENSG00000146729	55951792-56000181	+	-6466
7	61850843	61851082	240	61850972	7.19325	4.46755	2.54492	intergenic	ENSG00000233918	62275360-62275678	-	424716
7	61957177	61957401	225	61957265	5.39424	4.00432	1.09512	intergenic	ENSG00000233918	62275360-62275678	-	318389
7	62402757	62402998	242	62402974	4.31475	3.20475	0.59373	intergenic	ENSG00000233918	62275360-62275678	-	-127199
7	61850843	61851082	240	61850972	7.19325	4.46755	2.54492	intergenic	ENSG00000233918	62275360-62275678	-	424716
7	61957177	61957401	225	61957265	5.39424	4.00432	1.09512	intergenic	ENSG00000233918	62275360-62275678	-	318389
7	62402757	62402998	242	62402974	4.31475	3.20475	0.59373	intergenic	ENSG00000233918	62275360-62275678	-	-127199
7	61850843	61851082	240	61850972	7.19325	4.46755	2.54492	intergenic	ENSG00000233918	62275360-62275678	-	424716
7	61957177	61957401	225	61957265	5.39424	4.00432	1.09512	intergenic	ENSG00000233918	62275360-62275678	-	318389
7	62402757	62402998	242	62402974	4.31475	3.20475	0.59373	intergenic	ENSG00000233918	62275360-62275678	-	-127199
7	64522366	64522629	264	64522561	3.77589	3.20345	0.30123	ENSG00000173041:intron	ENSG00000228653	64500824-64501729	+	21673
7	65385885	65386126	242	65386081	5.56876	3.96799	1.238	ENSG00000146757:intron	ENSG00000146757	65373798-65401135	+	12207
7	66293401	66293775	375	66293546	5.39424	4.00432	1.09512	ENSG00000169902:intron	ENSG00000252126	66344302-66344414	+	-50714
7	67794312	67794576	265	67794370	4.56295	3.60388	0.59373	intergenic	ENSG00000223948	67769628-67770468	-	-23975
7	69192840	69193079	240	69193043	4.56295	3.60388	0.59373	intergenic	ENSG00000236839	69187832-69189318	-	-3641
7	73851511	73851790	280	73851673	13.56604	6.40949	8.26891	intergenic	ENSG00000165171	73834589-73842535	-	-9115
7	76898658	76898898	241	76898717	8.1168	5.20561	3.35333	ENSG00000285666:intron	ENSG00000231183	76902490-76919191	+	-3712
7	77820255	77820582	328	77820407	3.27191	2.87113	0.30123	ENSG00000006576:intron	ENSG00000006576	77798791-77957503	+	21627
7	78071182	78071385	204	78071236	3.77589	3.20345	0.30123	ENSG00000187391:intron	ENSG00000281008	78134078-78134851	+	-62795
7	78113994	78114205	212	78114158	4.07388	3.28775	0.47041	ENSG00000187391:intron	ENSG00000281008	78134078-78134851	+	-19979
7	78142228	78142610	383	78142264	3.34993	2.92244	0.30123	ENSG00000187391:intron	ENSG00000281008	78134078-78134851	+	8340
7	78496154	78496372	219	78496266	3.49438	2.91742	0.30123	ENSG00000187391:intron	ENSG00000226230	78486529-78487028	+	9733
7	81257269	81257562	294	81257315	5.39424	4.00432	1.09512	intergenic	ENSG00000226671	81191397-81192009	+	66018
7	84058822	84059125	304	84058953	5.01585	3.49609	0.89533	intergenic	ENSG00000230244	83663418-83664625	+	395555
7	90551771	90552039	269	90551836	5.19114	3.87478	1.02732	intergenic	ENSG00000223969	90590618-90597353	-	45448
7	91446430	91446635	206	91446551	4.51948	3.5757	0.59373	ENSG00000235450:intron;ENSG00000243144:intron	ENSG00000235450	91380777-91556848	-	110316
7	91545406	91545830	425	91545519	4.56295	3.60388	0.59373	ENSG00000235450:intron	ENSG00000235450	91380777-91556848	-	11230
7	91546559	91546765	207	91546623	5.39424	4.00432	1.09512	ENSG00000235450:intron	ENSG00000235450	91380777-91556848	-	10186
7	92633365	92633665	301	92633512	6.51641	4.07877	1.96373	ENSG00000105810:intron	ENSG00000234545	92560792-92590394	-	-43120
7	93773148	93773351	204	93773289	3.77589	3.20345	0.30123	ENSG00000127928:intron	ENSG00000225898	93777838-93778514	+	-4589
7	93784576	93784803	228	93784685	4.91514	3.56573	0.82412	ENSG00000127928:intron	ENSG00000225898	93777838-93778514	+	6851
7	94007681	94007956	276	94007702	3.88371	3.16559	0.37576	ENSG00000236861:intron	ENSG00000105829	93962761-94004382	-	-3436
7	94620716	94621005	290	94620872	11.66864	5.82681	6.52722	ENSG00000127990:intron	ENSG00000242265	94656324-94669695	+	-35464
7	97459946	97460358	413	97460088	3.77589	3.20345	0.30123	intergenic	ENSG00000226046	97437517-97437896	+	22634
7	99876388	99876658	271	99876516	4.11517	3.31434	0.49781	ENSG00000244623:exon	ENSG00000244623	99875986-99877057	-	534
7	100344854	100345160	307	100344998	15.03591	6.29939	9.62995	ENSG00000242294:intron;ENSG00000272752:intron	ENSG00000078319	100320991-100341908	-	-3098
7	101138540	101138782	243	101138636	8.3074	5.12886	3.50022	ENSG00000106366:three_prime_UTR;ENSG00000106366:exon	ENSG00000106366	101127088-101139266	+	11572
7	101739826	101740073	248	101739949	11.53275	5.55571	6.4016	intergenic	ENSG00000257923	101815903-102283958	+	-75954
7	103519545	103519847	303	103519762	5.6497	4.01836	1.29426	ENSG00000189056:intron	ENSG00000222386	103484207-103484539	-	-35156
7	106346712	106346933	222	106346790	10.20694	5.24451	5.19796	intergenic	ENSG00000243438	106315224-106315743	+	31598

7	107585410	107585853	444	107585813	3.20396	2.64557	0.26167	ENSG00000075790:intron	ENSG00000272854	107579556-107580057	-	-5574
7	107728418	107728632	215	107728575	5.01585	3.49609	0.89533	intergenic	ENSG00000105879	107743696-107761667	+	-15171
7	109169823	109170056	234	109170032	4.19696	3.36708	0.53407	intergenic	ENSG00000230941	109322319-109326315	-	156376
7	109205958	109206202	245	109206059	4.00012	3.12743	0.45978	intergenic	ENSG00000230941	109322319-109326315	-	120235
7	109563242	109563448	207	109563400	3.90845	3.18146	0.39391	ENSG00000234273:intron	ENSG00000234273	109521980-109597166	-	33821
7	109613121	109613353	233	109613267	5.6497	4.01836	1.29426	intergenic	ENSG00000234273	109521980-109597166	-	-16070
7	109893154	109893537	384	109893379	6.31582	4.27407	1.80607	intergenic	ENSG00000237064	109959217-109960184	-	66839
7	110273491	110273763	273	110273536	6.47779	4.37345	1.94623	intergenic	ENSG00000278424	110215890-110216380	+	57736
7	110919164	110919466	303	110919294	12.13524	6.07965	6.96558	ENSG00000184903:intron	ENSG00000173114	111091005-111125454	+	-171690
7	111054276	111054572	297	111054333	3.69375	3.04415	0.30123	ENSG00000184903:intron	ENSG00000173114	111091005-111125454	+	-36581
7	110919164	110919466	303	110919294	12.13524	6.07965	6.96558	ENSG00000184903:intron	ENSG00000173114	111091005-111125454	+	-171690
7	111054276	111054572	297	111054333	3.69375	3.04415	0.30123	ENSG00000184903:intron	ENSG00000173114	111091005-111125454	+	-36581
7	111953639	111953855	217	111953811	4.57196	3.35828	0.60151	ENSG00000128512:intron;ENSG00000222346:Promoter	ENSG00000222346	111953652-111953765	-	18
7	112016575	112016846	272	112016684	5.68188	3.88989	1.31322	ENSG00000128512:intron	ENSG00000234358	111971221-111971988	-	-44722
7	112902003	112902210	208	112902050	4.45692	3.17469	0.59373	ENSG00000164603:intron	ENSG00000164603	112819146-112939916	-	37810
7	112911062	112911296	235	112911144	4.00012	3.12743	0.45978	ENSG00000164603:intron	ENSG00000164603	112819146-112939916	-	28737
7	117267469	117267681	213	117267618	3.6499	2.91341	0.30123	intergenic	ENSG00000230785	117262917-117264888	-	-2686
7	118901961	118902222	262	118902149	4.07388	3.28775	0.47041	intergenic	ENSG00000229982	118880102-118881288	-	-20803
7	119974945	119975321	377	119975134	3.64836	3.01523	0.30123	intergenic	ENSG00000271739	120005975-120006101	-	30968
7	120233186	120233390	205	120233324	3.56683	2.68164	0.30123	intergenic	ENSG00000184408	120273667-120750331	+	-40379
7	120288644	120288856	213	120288707	5.56876	3.96799	1.238	ENSG00000184408:intron	ENSG00000184408	120273667-120750331	+	15082
7	120404588	120405223	636	120405181	4.1849	3.24157	0.52478	ENSG00000184408:intron	ENSG00000184408	120273667-120750331	+	131238
7	121138390	121138620	231	121138467	3.95704	2.57553	0.43404	ENSG00000106034:intron	ENSG00000212628	121083699-121083813	-	-54691
7	121174440	121174723	284	121174631	7.3143	4.5382	2.6406	ENSG00000106034:intron	ENSG00000207090	121194947-121195057	-	20476
7	121175553	121175847	295	121175762	3.6812	2.9324	0.30123	ENSG00000106034:intron	ENSG00000207090	121194947-121195057	-	19357
7	121278744	121279075	332	121278887	9.5279	4.89046	4.5986	ENSG00000106034:intron	ENSG00000002745	121325366-121341104	+	-46457
7	121478668	121478881	214	121478739	3.94435	3.09315	0.42361	intergenic	ENSG00000234985	121440833-121441261	-	-37513
7	121530441	121530801	361	121530675	5.84361	3.7038	1.44168	intergenic	ENSG00000234985	121440833-121441261	-	-89359
7	121544788	121545134	347	121544944	11.17905	5.37582	6.08588	intergenic	ENSG00000234985	121440833-121441261	-	-103699
7	121478668	121478881	214	121478739	3.94435	3.09315	0.42361	intergenic	ENSG00000234985	121440833-121441261	-	-37513
7	121530441	121530801	361	121530675	5.84361	3.7038	1.44168	intergenic	ENSG00000234985	121440833-121441261	-	-89359
7	121544788	121545134	347	121544944	11.17905	5.37582	6.08588	intergenic	ENSG00000234985	121440833-121441261	-	-103699
7	121983604	121983811	208	121983751	4.82376	3.51022	0.77787	ENSG00000106278:exon	ENSG00000252003	122081719-122081781	+	-98012
7	122053098	122053316	219	122053298	3.81246	2.91013	0.32594	ENSG00000106278:intron	ENSG00000252003	122081719-122081781	+	-28512
7	122081030	122081261	232	122081111	4.1849	3.24157	0.52478	ENSG00000252003:Promoter:ENSG00000008311:intron	ENSG00000252003	122081719-122081781	+	-574
7	122377590	122377970	381	122377854	4.68284	3.42499	0.68513	ENSG00000081803:intron;ENSG00000240499:intron	ENSG00000240499	122328468-122440388	+	49311
7	122397677	122398109	433	122398007	4.8107	3.50229	0.77787	ENSG00000081803:intron;ENSG00000240499:intron	ENSG00000240499	122328468-122440388	+	69424
7	122454950	122455160	211	122455099	3.88371	3.16559	0.37576	ENSG00000081803:intron	ENSG00000240499	122328468-122440388	+	126586
7	122377590	122377970	381	122377854	4.68284	3.42499	0.68513	ENSG00000081803:intron;ENSG00000240499:intron	ENSG00000240499	122328468-122440388	+	49311
7	122397677	122398109	433	122398007	4.8107	3.50229	0.77787	ENSG00000081803:intron;ENSG00000240499:intron	ENSG00000240499	122328468-122440388	+	69424
7	122454950	122455160	211	122455099	3.88371	3.16559	0.37576	ENSG00000081803:intron	ENSG00000240499	122328468-122440388	+	126586
7	122638799	122639026	228	122638968	4.11506	3.19834	0.49781	ENSG00000081803:intron	ENSG00000225795	122676579-122676811	+	-37667
7	124270527	124270759	233	124270739	3.90052	3.06626	0.38783	ENSG00000242593:intron	ENSG00000243574	124274678-124287544	-	16901
7	125578414	125578645	232	125578573	3.10079	2.75883	0.19252	intergenic	ENSG00000227869	125431346-125466401	+	147183
7	126093592	126093845	254	126093804	4.383	3.48731	0.59373	intergenic	ENSG00000197462	125917870-125933832	+	175848

7	130960324	130960606	283	130960511	5.40704	3.35205	1.10135	ENSG00000231721:intron;ENSG00000285106:intron	ENSG00000273319	130936463-130939661	+	24001
7	133645462	133646022	561	133645481	3.6499	2.91341	0.30123	ENSG00000131558:intron	ENSG00000235967	133727367-133727734	−	81992
7	134174819	134175079	261	134174915	8.31326	4.76113	3.50375	ENSG00000155530:intron	ENSG00000155530	134127298-134264591	+	47650
7	137070962	137071216	255	137071050	3.20396	2.64557	0.26167	ENSG00000234352:intron	ENSG00000224469	137164042-137164612	+	-92953
7	137078796	137079018	223	137078907	5.88285	4.01078	1.4765	ENSG00000234352:intron	ENSG00000224469	137164042-137164612	+	-85135
7	137337701	137338016	316	137337933	4.58409	3.36557	0.61075	ENSG00000105894:intron	ENSG00000105894	137227340-137343865	−	6007
7	137751443	137751710	268	137751619	6.13439	3.86438	1.6842	ENSG00000157680:intron	ENSG00000230383	137721984-137722846	+	29592
7	139916471	139916838	368	139916615	36.33923	9.8649	30.22827	ENSG00000059377:intron	ENSG00000064393	139561569-139777778	−	-138876
7	140876706	140877068	363	140876897	10.5753	5.64189	5.54838	ENSG00000157764:intron	ENSG00000271932	140884071-140884178	+	-7184
7	140927483	140927699	217	140927509	3.36027	2.73883	0.30123	intergenic	ENSG00000157764	140719326-140924928	−	-2662
7	141557959	141558214	256	141558083	9.68134	5.14987	4.74279	ENSG00000006530:intron	ENSG00000261570	141529202-141551304	−	-6782
7	141661142	141661456	315	141661411	4.14279	3.10309	0.51135	ENSG00000257093:exon;ENSG00000257093:three_prime_UTR	ENSG00000270157	141662921-141663846	−	2547
7	142188218	142188482	265	142188337	9.13305	4.25545	4.22677	ENSG00000257743:intron	ENSG00000240268	142240739-142247067	−	58717
7	142555705	142555967	263	142555746	4.2748	3.0719	0.58762	intergenic	ENSG00000276597	142554835-142555318	+	1000
7	142556727	142557004	278	142556835	5.26629	3.39198	1.07888	intergenic	ENSG00000276597	142554835-142555318	+	2030
7	142555705	142555967	263	142555746	4.2748	3.0719	0.58762	intergenic	ENSG00000276597	142554835-142555318	+	1000
7	142556727	142557004	278	142556835	5.26629	3.39198	1.07888	intergenic	ENSG00000276597	142554835-142555318	+	2030
7	142808827	142809051	225	142808932	4.11463	2.71871	0.49781	intergenic	ENSG00000237254	142812585-142813399	−	4460
7	144496113	144496368	256	144496243	4.14216	3.33173	0.51073	ENSG00000196511:intron	ENSG00000221507	144451680-144451817	+	44560
7	144512081	144512357	277	144512286	5.55248	3.95787	1.22965	ENSG00000196511:intron	ENSG00000221507	144451680-144451817	+	60538
7	144496113	144496368	256	144496243	4.14216	3.33173	0.51073	ENSG00000196511:intron	ENSG00000221507	144451680-144451817	+	44560
7	144512081	144512357	277	144512286	5.55248	3.95787	1.22965	ENSG00000196511:intron	ENSG00000221507	144451680-144451817	+	60538
7	145520623	145520918	296	145520787	7.60321	4.88949	2.89017	intergenic	ENSG00000231839	145583196-145584817	+	-62426
7	145523020	145523294	275	145523102	5.58512	3.97816	1.25272	intergenic	ENSG00000231839	145583196-145584817	+	-60039
7	145520623	145520918	296	145520787	7.60321	4.88949	2.89017	intergenic	ENSG00000231839	145583196-145584817	+	-62426
7	145523020	145523294	275	145523102	5.58512	3.97816	1.25272	intergenic	ENSG00000231839	145583196-145584817	+	-60039
7	145684129	145684360	232	145684315	4.30911	3.31873	0.59373	intergenic	ENSG00000230746	145675881-145681369	−	-2875
7	145794768	145795097	330	145795036	4.1849	3.24157	0.52478	intergenic	ENSG00000230746	145675881-145681369	−	-113563
7	145684129	145684360	232	145684315	4.30911	3.31873	0.59373	intergenic	ENSG00000230746	145675881-145681369	−	-2875
7	145794768	145795097	330	145795036	4.1849	3.24157	0.52478	intergenic	ENSG00000230746	145675881-145681369	−	-113563
7	146804224	146804548	325	146804355	6.75501	4.37612	2.16881	ENSG00000174469:intron	ENSG00000236795	147080933-147097609	−	293223
7	147005468	147005690	223	147005565	4.69706	3.56173	0.69764	ENSG00000174469:intron	ENSG00000236795	147080933-147097609	−	92030
7	147087932	147088156	225	147087964	4.49014	3.43176	0.59373	ENSG00000236795:intron;ENSG00000174469:intron	ENSG00000236795	147080933-147097609	−	9565
7	146804224	146804548	325	146804355	6.75501	4.37612	2.16881	ENSG00000174469:intron	ENSG00000236795	147080933-147097609	−	293223
7	147005468	147005690	223	147005565	4.69706	3.56173	0.69764	ENSG00000174469:intron	ENSG00000236795	147080933-147097609	−	92030
7	147087932	147088156	225	147087964	4.49014	3.43176	0.59373	ENSG00000236795:intron;ENSG00000174469:intron	ENSG00000236795	147080933-147097609	−	9565
7	147310878	147311136	259	147310971	5.97849	4.06862	1.55475	ENSG00000174469:intron	ENSG00000221442	147378016-147378121	−	67114
7	147630016	147630235	220	147630108	5.39672	3.71993	1.09738	ENSG00000174469:intron	ENSG00000230190	147671710-147673143	+	-41585
7	147766163	147766395	233	147766352	5.61808	3.99867	1.27988	ENSG00000174469:intron	ENSG00000270634	147704293-147704575	−	-61703
7	148564663	148564909	247	148564797	10.60166	5.08797	5.56659	ENSG00000283648:intron	ENSG00000283648	148543676-148572177	−	7391
7	149318175	149318391	217	149318292	5.45233	3.75292	1.13858	intergenic	ENSG00000231397	149321864-149322092	−	3809
7	149873750	149874146	397	149873810	5.2792	3.39885	1.08834	ENSG00000171130:exon;ENSG00000204934:intron;ENSG00000171130:five_prime_UTR	ENSG00000171130	149872967-149891204	+	980

7	150367938	150368236	299	150368078	19.84129	6.50196	14.21805	ENSG00000284041:Promoter;ENSG00000214022:Promoter;ENSG00000240449:intron	ENSG00000214022	150368188-150374044	+	-101
7	152193701	152194221	521	152194173	4.39825	3.3743	0.59373	ENSG00000055609:intron	ENSG00000213188	152128878-152129686	+	65082
7	152244677	152244881	205	152244754	8.2953	4.7511	3.48851	ENSG00000055609:intron	ENSG00000213188	152128878-152129686	+	115900
7	152193701	152194221	521	152194173	4.39825	3.3743	0.59373	ENSG00000055609:intron	ENSG00000213188	152128878-152129686	+	65082
7	152244677	152244881	205	152244754	8.2953	4.7511	3.48851	ENSG00000055609:intron	ENSG00000213188	152128878-152129686	+	115900
7	153070299	153070548	250	153070359	4.5035	3.44013	0.59373	intergenic	ENSG00000224104	153144312-153144588	-	74165
7	153412287	153412797	511	153412403	4.45692	3.17469	0.59373	ENSG00000234722:intron	ENSG00000234722	153355364-153413981	-	1439
7	153866047	153866261	215	153866112	4.45692	3.17469	0.59373	intergenic	ENSG00000130226	153887096-154894285	+	-20942
7	154488601	154489020	420	154488699	3.02441	2.62206	0.15111	ENSG00000130226:intron	ENSG00000236408	154838387-154865483	-	376673
7	154608671	154609130	460	154609039	4.07388	3.28775	0.47041	ENSG00000130226:intron	ENSG00000236408	154838387-154865483	-	256583
7	154652698	154652908	211	154652822	3.53038	3.04135	0.30123	ENSG00000130226:intron	ENSG00000236408	154838387-154865483	-	212680
7	154784464	154784810	347	154784655	4.23916	3.15996	0.56297	ENSG00000130226:intron	ENSG00000236408	154838387-154865483	-	80846
7	154488601	154489020	420	154488699	3.02441	2.62206	0.15111	ENSG00000130226:intron	ENSG00000236408	154838387-154865483	-	376673
7	154608671	154609130	460	154609039	4.07388	3.28775	0.47041	ENSG00000130226:intron	ENSG00000236408	154838387-154865483	-	256583
7	154652698	154652908	211	154652822	3.53038	3.04135	0.30123	ENSG00000130226:intron	ENSG00000236408	154838387-154865483	-	212680
7	154784464	154784810	347	154784655	4.23916	3.15996	0.56297	ENSG00000130226:intron	ENSG00000236408	154838387-154865483	-	80846
7	154488601	154489020	420	154488699	3.02441	2.62206	0.15111	ENSG00000130226:intron	ENSG00000236408	154838387-154865483	-	376673
7	154608671	154609130	460	154609039	4.07388	3.28775	0.47041	ENSG00000130226:intron	ENSG00000236408	154838387-154865483	-	256583
7	154652698	154652908	211	154652822	3.53038	3.04135	0.30123	ENSG00000130226:intron	ENSG00000236408	154838387-154865483	-	212680
7	154784464	154784810	347	154784655	4.23916	3.15996	0.56297	ENSG00000130226:intron	ENSG00000236408	154838387-154865483	-	80846
7	157368687	157368922	236	157368877	4.15047	3.22024	0.51719	ENSG00000105993:intron	ENSG00000105993	157335380-157417439	+	33424
7	158283619	158283839	221	158283669	3.64136	2.90822	0.30123	ENSG00000155093:intron	ENSG00000207637	158532717-158532813	-	249084
7	158381324	158381553	230	158381449	5.58064	3.82933	1.24914	ENSG00000155093:intron	ENSG00000207637	158532717-158532813	-	151375
7	158405687	158406007	321	158405870	4.22261	3.26496	0.55558	ENSG00000155093:intron	ENSG00000207637	158532717-158532813	-	126966
7	158283619	158283839	221	158283669	3.64136	2.90822	0.30123	ENSG00000155093:intron	ENSG00000207637	158532717-158532813	-	249084
7	158381324	158381553	230	158381449	5.58064	3.82933	1.24914	ENSG00000155093:intron	ENSG00000207637	158532717-158532813	-	151375
7	158405687	158406007	321	158405870	4.22261	3.26496	0.55558	ENSG00000155093:intron	ENSG00000207637	158532717-158532813	-	126966
7	159246545	159246862	318	159246801	3.88967	3.0596	0.37913	intergenic	ENSG00000229435	159231434-159233377	+	15269
8	354285	354538	254	354476	5.39424	4.00432	1.09512	intergenic	ENSG00000249868	311132-331026	-	-23385
8	574612	574884	273	574719	4.56295	3.60388	0.59373	intergenic	ENSG00000180190	489791-545781	-	-28966
8	3575841	3576077	237	3575880	6.26578	4.40475	1.75945	ENSG00000183117:intron	ENSG00000222546	3700491-3700628	+	-124532
8	3949552	3949825	274	3949647	3.03786	2.80302	0.15111	ENSG00000183117:intron	ENSG00000222546	3700491-3700628	+	249197
8	5815337	5815566	230	5815363	4.76929	3.60726	0.74587	intergenic	ENSG00000253571	5848696-5849692	+	-33245
8	6109671	6109960	290	6109763	5.39424	4.00432	1.09512	ENSG00000253880:intron	ENSG00000253189	6036432-6036974	-	-72841
8	9087893	9088179	287	9088015	8.92565	5.30243	4.07488	ENSG00000104626:intron	ENSG00000197334	9091239-9091508	+	-3203
8	9101475	9101772	298	9101750	3.77589	3.20345	0.30123	ENSG00000104626:intron	ENSG00000197334	9091239-9091508	+	10384
8	9125331	9125570	240	9125425	5.68188	3.88989	1.31322	intergenic	ENSG00000254340	9141423-9145435	+	-15973
8	10232664	10232881	218	10232672	3.77589	3.20345	0.30123	ENSG00000175806:intron	ENSG00000285606	10275498-10279737	-	46965
8	10248826	10249147	322	10248944	7.17425	4.80518	2.52654	ENSG00000175806:intron	ENSG00000285606	10275498-10279737	-	30751
8	13041502	13041774	273	13041530	4.1849	3.24157	0.52478	intergenic	ENSG00000206996	13044349-13044456	+	-2711
8	14144980	14145356	377	14145068	4.56295	3.60388	0.59373	ENSG00000185053:intron	ENSG00000254575	14161296-14165359	+	-16128
8	16478764	16479023	260	16478891	4.14216	3.33173	0.51073	ENSG00000038945:intron	ENSG00000038945	16107877-16567490	-	88597
8	17826784	17827143	360	17826987	4.919	3.70193	0.82412	ENSG00000253944:intron	ENSG00000253671	17808940-17820868	+	18023
8	19908763	19908986	224	19908794	3.32816	2.90812	0.30123	ENSG00000175445:intron	ENSG00000175445	19901716-19967259	+	7158
8	23439529	23439929	401	23439646	3.77589	3.20345	0.30123	ENSG00000197217:intron	ENSG00000134013	23297188-23425328	-	-14400
8	25108574	25108905	332	25108663	4.1849	3.24157	0.52478	intergenic	ENSG00000253670	25141393-25141968	-	33229
8	27575272	27575498	227	27575405	4.81345	3.63515	0.77787	ENSG00000234770:intron	ENSG00000234770	27560273-27589073	+	15111

8	27614632	27614985	354	27614808	22.91595	7.35032	17.18397	ENSG00000120885:five_prime_UTR;ENSG00000120885:exon	ENSG00000120885	27596916-27615031	-	223
8	28341163	28341411	249	28341303	9.02537	5.16418	4.14689	ENSG00000168081:intron	ENSG00000253690	28250062-28339255	-	-2031
8	29555275	29555592	318	29555419	9.34519	5.55289	4.42343	ENSG00000253182:intron	ENSG00000253182	29548170-29564341	+	7263
8	29614624	29614846	223	29614775	7.34679	4.23292	2.66603	intergenic	ENSG00000243844	29632090-29632644	+	-17355
8	29665079	29665468	390	29665249	12.43382	5.82026	7.22732	intergenic	ENSG00000243844	29632090-29632644	+	33183
8	29753432	29753723	292	29753541	9.85502	5.24413	4.88402	ENSG00000253490:intron	ENSG00000253490	29748308-29798492	+	5269
8	30317525	30317756	232	30317552	5.78764	4.10443	1.39623	intergenic	ENSG00000253346	30331960-30332277	-	14637
8	30655983	30656221	239	30656114	12.70978	6.39668	7.48239	ENSG00000197265:intron	ENSG00000197265	30578317-30658251	-	2149
8	30880117	30880349	233	30880234	4.45692	3.17469	0.59373	ENSG00000133863:intron	ENSG00000133863	30831543-30913003	-	32770
8	31100622	31100899	278	31100772	5.39424	4.00432	1.09512	ENSG00000165392:intron	ENSG00000253579	31131538-31131802	-	31042
8	31107166	31107469	304	31107386	4.28127	3.42152	0.58762	ENSG00000165392:intron	ENSG00000253579	31131538-31131802	-	24485
8	31100622	31100899	278	31100772	5.39424	4.00432	1.09512	ENSG00000165392:intron	ENSG00000253579	31131538-31131802	-	31042
8	31107166	31107469	304	31107386	4.28127	3.42152	0.58762	ENSG00000165392:intron	ENSG00000253579	31131538-31131802	-	24485
8	34906865	34907100	236	34906996	5.39424	4.00432	1.09512	intergenic	ENSG00000243181	34874158-34874633	+	32824
8	35011691	35011948	258	35011876	4.07388	3.28775	0.47041	intergenic	ENSG00000243181	34874158-34874633	+	137661
8	37144679	37144996	318	37144897	7.37304	4.74897	2.68538	intergenic	ENSG00000253650	37095149-37095390	+	49688
8	37433510	37433907	398	37433884	4.56295	3.60388	0.59373	intergenic	ENSG00000253746	37405438-37406724	-	-26984
8	38658570	38658815	246	38658778	4.91514	3.56573	0.82412	intergenic	ENSG00000254321	38700625-38704855	-	46163
8	39725292	39725582	291	39725387	3.77589	3.20345	0.30123	ENSG00000168619:intron	ENSG00000104755	39743734-39838289	-	112852
8	42203361	42203597	237	42203547	5.2271	3.75666	1.05667	ENSG00000104368:intron	ENSG00000104368	42175232-42207724	-	4245
8	42321522	42321788	267	42321599	4.82831	3.64454	0.77787	ENSG00000104365:intron	ENSG00000070501	42338453-42371808	+	-16798
8	42856971	42857231	261	42857077	7.25542	4.67748	2.59922	ENSG00000120925:intron	ENSG00000131931	42836673-42843325	-	-13775
8	46288688	46288929	242	46288818	6.26578	4.40475	1.75945	intergenic	ENSG00000276685	46028803-46028892	-	-259916
8	47954898	47955113	216	47955023	4.96535	3.7313	0.86205	ENSG00000253729:intron	ENSG00000253729	47773107-47960183	-	5178
8	50271610	50271847	238	50271701	3.77589	3.20345	0.30123	ENSG00000147481:intron	ENSG00000253849	50381014-50382275	+	-109286
8	50294636	50294940	305	50294805	7.17425	4.80518	2.52654	ENSG00000147481:intron	ENSG00000253849	50381014-50382275	+	-86226
8	51869952	51870312	361	51870098	4.56295	3.60388	0.59373	ENSG00000168300:intron	ENSG00000253475	51895956-51896374	-	26242
8	54663903	54664221	319	54664140	5.39424	4.00432	1.09512	ENSG00000104237:intron	ENSG00000253114	54696397-54697127	+	-32335
8	58446067	58446410	344	58446069	3.49438	2.91742	0.30123	ENSG00000215114:intron	ENSG00000250031	58424587-58426685	-	-19553
8	59339099	59339328	230	59339116	4.07388	3.28775	0.47041	intergenic	ENSG00000201763	59455884-59456010	+	-116671
8	59345698	59345906	209	59345896	3.77589	3.20345	0.30123	intergenic	ENSG00000201763	59455884-59456010	+	-110082
8	60051576	60051812	237	60051700	6.51641	4.07877	1.96373	ENSG00000254775:intron	ENSG00000254775	60049586-60074715	+	2107
8	60780820	60781143	324	60780964	9.85502	5.24413	4.88402	ENSG00000171316:intron	ENSG00000254432	60808734-60809606	-	28625
8	60848226	60848460	235	60848368	7.66756	4.92891	2.95201	ENSG00000171316:intron	ENSG00000254432	60808734-60809606	-	-38736
8	60780820	60781143	324	60780964	9.85502	5.24413	4.88402	ENSG00000171316:intron	ENSG00000254432	60808734-60809606	-	28625
8	60848226	60848460	235	60848368	7.66756	4.92891	2.95201	ENSG00000171316:intron	ENSG00000254432	60808734-60809606	-	-38736
8	61399223	61399443	221	61399287	3.34993	2.92244	0.30123	intergenic	ENSG00000253711	61300163-61301069	-	-98263
8	62915376	62915837	462	62915670	4.56295	3.60388	0.59373	ENSG00000185942:intron	ENSG00000253431	62863081-62864181	-	-51425
8	63620247	63620495	249	63620396	4.07388	3.28775	0.47041	intergenic	ENSG00000252358	63609353-63609600	-	-10770
8	69475141	69475378	238	69475329	5.06016	3.79147	0.9304	ENSG00000137573:intron	ENSG00000137573	69466623-69660915	+	8636
8	69536957	69537163	207	69537040	7.37304	4.74897	2.68538	ENSG00000137573:intron	ENSG00000137573	69466623-69660915	+	70436
8	69678748	69678986	239	69678937	3.6499	2.91341	0.30123	ENSG00000137571:intron	ENSG00000222889	69700223-69700495	+	-21356
8	70013580	70013804	225	70013695	4.93185	3.44723	0.83372	intergenic	ENSG00000253721	69987414-69987697	-	-25994
8	70015437	70015669	233	70015551	9.06429	5.18652	4.17525	intergenic	ENSG00000253721	69987414-69987697	-	-27855
8	71253656	71253895	240	71253789	7.58982	3.93998	2.87738	ENSG00000104313:intron	ENSG00000254031	71155456-71204223	+	98319
8	71263941	71264162	222	71264076	5.07624	3.80169	0.93914	ENSG00000104313:intron	ENSG00000254031	71155456-71204223	+	108595
8	71253656	71253895	240	71253789	7.58982	3.93998	2.87738	ENSG00000104313:intron	ENSG00000254031	71155456-71204223	+	98319
8	71263941	71264162	222	71264076	5.07624	3.80169	0.93914	ENSG00000104313:intron	ENSG00000254031	71155456-71204223	+	108595
8	72000384	72000625	242	72000538	5.73591	4.07212	1.35894	ENSG00000235531:intron	ENSG00000104321	72019916-72075617	-	75113

8	72080837	72081082	246	72080930	3.34993	2.92244	0.30123	ENSG00000235531:intron	ENSG00000104321	72019916-72075617	-	-5342
8	72102974	72103193	220	72103112	3.03786	2.80302	0.15111	ENSG00000235531:intron	ENSG00000104321	72019916-72075617	-	-27466
8	72823114	72823394	281	72823229	10.98447	6.32119	5.90745	ENSG00000182674:intron	ENSG00000253784	72874858-72881740	-	58486
8	74131539	74132001	463	74131832	4.84177	3.65305	0.77787	intergenic	ENSG00000253115	74103515-74106744	+	28254
8	78348267	78348601	335	78348488	4.56295	3.60388	0.59373	intergenic	ENSG00000252935	78398514-78398622	-	50188
8	78386433	78386647	215	78386516	3.77589	3.20345	0.30123	intergenic	ENSG00000252935	78398514-78398622	-	12082
8	78387073	78387315	243	78387158	9.43096	5.20133	4.50508	intergenic	ENSG00000252935	78398514-78398622	-	11428
8	78348267	78348601	335	78348488	4.56295	3.60388	0.59373	intergenic	ENSG00000252935	78398514-78398622	-	50188
8	78386433	78386647	215	78386516	3.77589	3.20345	0.30123	intergenic	ENSG00000252935	78398514-78398622	-	12082
8	78387073	78387315	243	78387158	9.43096	5.20133	4.50508	intergenic	ENSG00000252935	78398514-78398622	-	11428
8	78548356	78548632	277	78548580	4.56295	3.60388	0.59373	ENSG00000254266:intron;ENSG00000171033:intron	ENSG00000254266	78426102-78558503	-	10009
8	80957676	80958020	345	80957873	3.77589	3.20345	0.30123	intergenic	ENSG00000254060	81036963-81040831	+	-79115
8	81025620	81025844	225	81025639	5.22474	3.89619	1.05478	ENSG00000076641:intron	ENSG00000254060	81036963-81040831	+	-11231
8	102653888	102654118	231	102653961	5.75051	3.78743	1.36222	ENSG00000155090:intron	ENSG00000155090	102648778-102655902	-	1899
8	116649685	116650034	350	116649897	4.60755	3.1497	0.62762	ENSG00000147677:intron	ENSG00000147679	116766502-116849463	+	-116643
8	120567319	120567620	302	120567572	3.29424	2.88581	0.30123	ENSG00000172164:intron	ENSG00000250290	120635557-120638204	-	70735
8	120586552	120586864	313	120586725	4.07388	3.28775	0.47041	ENSG00000172164:intron	ENSG00000250290	120635557-120638204	-	51496
8	120567319	120567620	302	120567572	3.29424	2.88581	0.30123	ENSG00000172164:intron	ENSG00000250290	120635557-120638204	-	70735
8	120586552	120586864	313	120586725	4.07388	3.28775	0.47041	ENSG00000172164:intron	ENSG00000250290	120635557-120638204	-	51496
8	121909781	121909999	219	121909951	4.2388	3.39408	0.56297	ENSG00000248690:intron	ENSG00000253814	122091479-122091609	+	-181589
8	125110905	125111110	206	125111025	6.58155	3.96836	2.01637	ENSG00000156831:intron	ENSG00000164961	125024259-125091840	-	-19167
8	125130550	125130759	210	125130746	3.95865	3.21367	0.43404	ENSG00000156831:intron	ENSG00000164961	125024259-125091840	-	-38814
8	125151753	125151994	242	125151883	4.45692	3.17469	0.59373	ENSG00000156831:intron	ENSG00000164961	125024259-125091840	-	-60033
8	125352850	125353141	292	125352946	8.10122	4.81936	3.34921	ENSG00000156831:intron;ENSG00000254431:Promoter	ENSG00000254431	125348195-125351416	-	-1579
8	125360324	125360564	241	125360420	4.813	3.37843	0.77787	ENSG00000156831:intron	ENSG00000254431	125348195-125351416	-	-9027
8	125352850	125353141	292	125352946	8.10122	4.81936	3.34921	ENSG00000156831:intron;ENSG00000254431:Promoter	ENSG00000254431	125348195-125351416	-	-1579
8	125360324	125360564	241	125360420	4.813	3.37843	0.77787	ENSG00000156831:intron	ENSG00000254431	125348195-125351416	-	-9027
8	125637561	125637803	243	125637671	11.78444	5.68533	6.63496	intergenic	ENSG00000253470	125749054-125750241	-	112559
8	125644469	125644765	297	125644605	11.53275	5.55571	6.4016	intergenic	ENSG00000253470	125749054-125750241	-	105624
8	125698793	125699118	326	125698937	16.28881	7.39114	10.81485	intergenic	ENSG00000253470	125749054-125750241	-	51286
8	125769228	125769635	408	125769274	3.02441	2.62206	0.15111	intergenic	ENSG00000253470	125749054-125750241	-	-19190
8	125637561	125637803	243	125637671	11.78444	5.68533	6.63496	intergenic	ENSG00000253470	125749054-125750241	-	112559
8	125644469	125644765	297	125644605	11.53275	5.55571	6.4016	intergenic	ENSG00000253470	125749054-125750241	-	105624
8	125698793	125699118	326	125698937	16.28881	7.39114	10.81485	intergenic	ENSG00000253470	125749054-125750241	-	51286
8	125769228	125769635	408	125769274	3.02441	2.62206	0.15111	intergenic	ENSG00000253470	125749054-125750241	-	-19190
8	125637561	125637803	243	125637671	11.78444	5.68533	6.63496	intergenic	ENSG00000253470	125749054-125750241	-	112559
8	125644469	125644765	297	125644605	11.53275	5.55571	6.4016	intergenic	ENSG00000253470	125749054-125750241	-	105624
8	125698793	125699118	326	125698937	16.28881	7.39114	10.81485	intergenic	ENSG00000253470	125749054-125750241	-	51286
8	125769228	125769635	408	125769274	3.02441	2.62206	0.15111	intergenic	ENSG00000253470	125749054-125750241	-	-19190
8	125637561	125637803	243	125637671	11.78444	5.68533	6.63496	intergenic	ENSG00000253470	125749054-125750241	-	112559
8	125644469	125644765	297	125644605	11.53275	5.55571	6.4016	intergenic	ENSG00000253470	125749054-125750241	-	105624
8	125698793	125699118	326	125698937	16.28881	7.39114	10.81485	intergenic	ENSG00000253470	125749054-125750241	-	51286
8	125769228	125769635	408	125769274	3.02441	2.62206	0.15111	intergenic	ENSG00000253470	125749054-125750241	-	-19190
8	125942245	125942505	261	125942339	5.74381	3.92704	1.36222	ENSG00000245164:intron	ENSG00000245164	125922307-125951249	-	8874
8	126006616	126006892	277	126006653	4.1849	3.24157	0.52478	intergenic	ENSG00000254227	125998993-126001001	-	-5752
8	126966190	126966545	356	126966408	4.30996	3.44006	0.59373	ENSG00000253438:intron	ENSG00000282961	127079873-127092600	+	-113506
8	127970996	127971270	275	127971173	5.89863	4.02031	1.48878	ENSG00000249859:intron	ENSG00000221771	127960632-127960695	+	10500
8	128222012	128222226	215	128222129	3.73002	2.77027	0.30123	intergenic	ENSG00000201782	128220503-128220803	+	1615

8	128240405	128240739	335	128240549	15.24834	6.61394	9.82777	intergenic	ENSG00000201782	128220503-128220803	+	20068
8	128657428	128657738	311	128657580	9.85502	5.24413	4.88402	intergenic	ENSG00000254275	128405268-128564679	−	-92903
8	128824485	128824688	204	128824495	3.23478	2.75364	0.28314	intergenic	ENSG00000254275	128405268-128564679	−	-259907
8	128903842	128904052	211	128903976	4.65453	3.53495	0.66139	intergenic	ENSG00000254275	128405268-128564679	−	-339267
8	129048609	129048846	238	129048711	6.49439	4.38367	1.95029	ENSG00000229140:intron	ENSG00000253225	129250966-129251138	+	-202239
8	129206203	129206482	280	129206349	4.99664	3.75114	0.88739	ENSG00000229140:intron	ENSG00000253225	129250966-129251138	+	-44624
8	130174493	130174740	248	130174622	7.07338	4.39791	2.45329	ENSG00000153317:intron	ENSG00000212342	130162971-130163127	+	11645
8	130444688	130444937	250	130444815	7.53075	4.33168	2.82489	ENSG00000153317:Promoter	ENSG00000153317	130052103-130443660	−	-1152
8	131366491	131366707	217	131366654	6.03946	4.26226	1.60751	intergenic	ENSG00000253507	131308544-131317632	+	58054
8	131846599	131847277	679	131846975	3.49438	2.91742	0.30123	intergenic	ENSG00000132294	131904087-132013642	+	-57149
8	132276470	132276713	244	132276580	9.33092	5.14553	4.41089	ENSG00000184156:intron	ENSG00000132297	132061485-132111159	−	-165432
8	133730906	133731211	306	133731027	4.18632	3.24245	0.52565	intergenic	ENSG00000253970	133754810-133755450	+	-23752
8	134946096	134946334	239	134946176	4.72575	3.5798	0.71873	intergenic	ENSG00000244413	134978826-134979279	−	33064
8	135069474	135069701	228	135069632	4.55072	3.59595	0.59373	intergenic	ENSG00000244413	134978826-134979279	−	-90308
8	135574783	135575065	283	135574904	4.11517	3.31434	0.49781	ENSG00000131773:intron	ENSG00000253915	135625184-135625981	−	51057
8	136248494	136248700	207	136248646	4.47684	3.42343	0.59373	intergenic	ENSG00000285683	136237385-136241532	−	-7064
8	136333637	136333924	288	136333731	7.38831	4.58155	2.69775	intergenic	ENSG00000285683	136237385-136241532	−	-92248
8	138210105	138210440	336	138210238	5.96569	4.21594	1.54281	ENSG00000147724:intron	ENSG00000253288	137809443-138083570	−	-126702
8	138402658	138402918	261	138402843	4.91514	3.56573	0.82412	ENSG00000147724:intron	ENSG00000147724	138130022-138496822	−	94034
8	138453792	138453999	208	138453947	4.56295	3.60388	0.59373	ENSG00000147724:intron	ENSG00000147724	138130022-138496822	−	42927
8	139021888	139022148	261	139022035	5.48809	3.9179	1.17181	intergenic	ENSG00000253432	139096304-139102830	−	80812
8	140591427	140591688	262	140591615	5.65806	3.7345	1.30082	ENSG00000123908:intron;ENSG00000280326:Promoter	ENSG00000280326	140591683-140593201	+	-126
8	143110727	143111200	474	143110778	4.06198	3.28009	0.47041	intergenic	ENSG00000261667	143080456-143083001	+	30507
8	143936480	143936730	251	143936596	15.01379	5.73717	9.61104	ENSG00000178209:intron	ENSG00000207574	143945190-143945279	−	8674
9	2305651	2306237	587	2305818	4.56295	3.60388	0.59373	intergenic	ENSG00000264615	2228960-2229241	−	-76702
9	4121802	4122046	245	4121963	5.6497	4.01836	1.29426	ENSG00000107249:intron	ENSG00000230001	4070905-4071884	−	-50039
9	4153329	4153606	278	4153467	3.49438	2.91742	0.30123	ENSG00000107249:intron	ENSG00000230001	4070905-4071884	−	-81583
9	5768501	5768737	237	5768553	8.134	4.66145	3.3598	ENSG00000107036:intron;ENSG00000099219:intron	ENSG00000225408	5719020-5720244	−	-48374
9	7727110	7727435	326	7727298	3.6499	2.91341	0.30123	intergenic	ENSG00000273056	7786104-7786688	−	59416
9	7756098	7756305	208	7756295	3.37418	2.93841	0.30123	intergenic	ENSG00000273056	7786104-7786688	−	30487
9	11757689	11757951	263	11757782	4.56295	3.60388	0.59373	ENSG00000285784:intron	ENSG00000275130	11618495-11618621	−	-139198
9	30075543	30075913	371	30075743	14.37081	7.6082	9.00841	intergenic	ENSG00000230097	29824741-29826711	−	-249016
9	33988497	33988782	286	33988613	10.79066	5.97945	5.72727	ENSG00000137073:intron	ENSG00000226801	33968070-33968518	−	-20121
9	37409287	37409649	363	37409480	24.61963	9.03149	18.83339	intergenic	ENSG00000137106	37422665-37436990	+	-13197
9	37596983	37597323	341	37597145	4.87332	3.67301	0.80353	ENSG00000255872:intron	ENSG00000175768	37582645-37592642	−	-4510
9	37804992	37805257	266	37805046	4.5035	3.44013	0.59373	ENSG00000255872:intron;ENSG00000122741:intron	ENSG00000107371	37766977-37801437	−	-3687
9	41220450	41220763	314	41220622	3.9797	2.90741	0.44999	intergenic	ENSG00000276029	41233754-41233835	+	-13148
9	41303305	41303707	403	41303376	4.84177	3.65305	0.77787	intergenic	ENSG00000274680	41292886-41294125	+	10619
9	41303305	41303707	403	41303376	4.84177	3.65305	0.77787	intergenic	ENSG00000274680	41292886-41294125	+	10619
9	63821321	63821554	234	63821417	5.80381	3.06316	1.41073	intergenic	ENSG00000266017	63819573-63819654	+	1864
9	69346842	69347057	216	69346939	4.31475	3.20475	0.59373	ENSG00000135063:intron	ENSG00000135063	69324566-69392558	+	22383
9	71814349	71814555	207	71814513	3.18471	2.81386	0.2494	intergenic	ENSG00000135048	71683365-71816690	−	2238
9	71858780	71859084	305	71858898	6.26578	4.40475	1.75945	intergenic	ENSG00000135048	71683365-71816690	−	-42241
9	72294561	72295028	468	72294850	4.56295	3.60388	0.59373	intergenic	ENSG00000252428	72299719-72299838	−	5044
9	72889099	72889368	270	72889275	6.49439	4.38367	1.95029	intergenic	ENSG00000236849	72871727-72874109	−	-15124
9	73149496	73149784	289	73149666	9.09099	5.60604	4.18865	intergenic	ENSG00000135046	73151756-73170393	+	-2116
9	73279360	73279569	210	73279528	3.77589	3.20345	0.30123	intergenic	ENSG00000135046	73151756-73170393	+	127708
9	73716752	73716986	235	73716865	6.49439	4.38367	1.95029	intergenic	ENSG00000212454	73750971-73751077	−	34208

9	73772767	73773002	236	73772827	4.56295	3.60388	0.59373	intergenic	ENSG00000212454	73750971-73751077	-	-21807
9	73785612	73786013	402	73785844	3.34993	2.92244	0.30123	intergenic	ENSG00000212454	73750971-73751077	-	-34735
9	75175743	75176009	267	75175903	6.49439	4.38367	1.95029	intergenic	ENSG00000200041	75181519-75181621	-	5745
9	76596244	76596478	235	76596357	6.49439	4.38367	1.95029	ENSG00000187210:intron	ENSG00000219149	76549281-76549771	-	-46589
9	77273556	77273779	224	77273716	5.34519	3.973	1.09512	ENSG00000197969:intron	ENSG00000232998	77176759-77178180	-	-95487
9	78464564	78464881	318	78464829	4.42776	3.51628	0.59373	intergenic	ENSG00000270627	78589157-78589539	+	-124435
9	78560636	78561006	371	78560792	3.03786	2.80302	0.15111	intergenic	ENSG00000270627	78589157-78589539	+	-28336
9	78570667	78570874	208	78570737	3.03786	2.80302	0.15111	intergenic	ENSG00000270627	78589157-78589539	+	-18387
9	78636480	78636783	304	78636627	4.56295	3.60388	0.59373	intergenic	ENSG00000270627	78589157-78589539	+	47474
9	88589557	88589869	313	88589681	3.56683	2.68164	0.30123	intergenic	ENSG00000130045	88534032-88584274	+	55680
9	94107189	94107490	302	94107284	4.56295	3.60388	0.59373	ENSG00000158079:intron	ENSG00000269946	94166288-94200627	+	-58949
9	94698039	94698354	316	94698088	4.458	3.53587	0.59373	intergenic	ENSG00000148120	94726700-95087218	+	-28504
9	94702455	94702661	207	94702551	5.27581	3.92874	1.08581	intergenic	ENSG00000148120	94726700-95087218	+	-24142
9	95028237	95028588	352	95028426	5.14139	3.84312	0.98688	ENSG00000148120:intron	ENSG00000274115	95065349-95065446	+	-36937
9	97704124	97704416	293	97704210	3.49438	2.91742	0.30123	ENSG00000236130:intron	ENSG00000214417	97698921-97700734	+	5348
9	99927149	99927442	294	99927189	4.56295	3.60388	0.59373	ENSG00000254571:intron;ENSG00000136874:intron	ENSG00000254571	99915076-99929896	-	2601
9	103150239	103150537	299	103150458	4.56295	3.60388	0.59373	ENSG00000225564:intron	ENSG00000229830	103065415-103065598	+	84972
9	104961288	104961562	275	104961415	12.53113	6.77006	7.32038	intergenic	ENSG00000230013	104990795-104991781	-	30356
9	106463832	106464062	231	106464015	4.44284	3.52605	0.59373	ENSG00000234323:intron	ENSG00000229297	106584141-106591952	-	128005
9	108906918	108907209	292	108907107	5.6497	4.01836	1.29426	ENSG00000070061:intron	ENSG00000070061	108867516-108934116	-	27053
9	110808745	110809003	259	110808883	8.134	4.66145	3.3598	intergenic	ENSG00000223918	110727455-110727679	-	-81194
9	112696997	112697247	251	112697187	3.49438	2.91742	0.30123	ENSG00000148153:intron	ENSG00000148153	112683925-112718236	-	21114
9	112721768	112721976	209	112721888	3.67095	3.02962	0.30123	intergenic	ENSG00000148153	112683925-112718236	-	-3635
9	113311115	113311413	299	113311157	3.02441	2.62206	0.15111	intergenic	ENSG00000165188	113297092-113303376	-	-7887
9	113579001	113579251	251	113579180	8.60945	4.92743	3.78062	ENSG00000237073:intron;ENSG00000138835:intron	ENSG00000237073	113570189-113590019	-	10893
9	114267662	114268104	443	114267823	4.84177	3.65305	0.77787	ENSG00000196739:intron	ENSG00000229314	114323055-114326475	+	-55172
9	114385454	114385776	323	114385626	9.34358	4.96849	4.42228	ENSG00000106948:intron	ENSG00000106948	114334155-114394405	-	8790
9	114447444	114447662	219	114447641	4.76929	3.60726	0.74587	ENSG00000095397:intron	ENSG00000271214	114491563-114491741	+	-44010
9	114485006	114485347	342	114485161	15.75907	7.35618	10.3066	ENSG00000095397:intron	ENSG00000271214	114491563-114491741	+	-6387
9	114447444	114447662	219	114447641	4.76929	3.60726	0.74587	ENSG00000095397:intron	ENSG00000271214	114491563-114491741	+	-44010
9	114485006	114485347	342	114485161	15.75907	7.35618	10.3066	ENSG00000095397:intron	ENSG00000271214	114491563-114491741	+	-6387
9	114696876	114697465	590	114697336	7.37304	4.74897	2.68538	ENSG00000230284:Promoter	ENSG00000230284	114695921-114696315	-	-855
9	114696876	114697465	590	114697336	7.37304	4.74897	2.68538	ENSG00000230284:Promoter	ENSG00000230284	114695921-114696315	-	-855
9	118723312	118723542	231	118723518	5.39424	4.00432	1.09512	intergenic	ENSG00000235201	118740562-118745312	+	-17135
9	120173232	120173451	220	120173357	7.91183	5.07907	3.17539	intergenic	ENSG00000207814	120244978-120245050	-	71709
9	120404878	120405084	207	120404951	3.02441	2.62206	0.15111	ENSG00000136861:intron	ENSG00000207814	120244978-120245050	-	-159930
9	120584099	120584351	253	120584200	9.65073	4.95373	4.71281	intergenic	ENSG00000136861	120388868-120580170	-	-4054
9	120892748	120892998	251	120892861	7.36237	4.39861	2.67971	ENSG00000119403:intron	ENSG00000119403	120855651-120894896	-	2023
9	123284920	123285168	249	123284965	4.15576	3.3405	0.52015	intergenic	ENSG00000165209	123109499-123268576	-	-16467
9	124218754	124219016	263	124218876	9.77126	4.84568	4.82479	intergenic	ENSG00000119408	124257605-124353307	+	-38720
9	125478485	125478785	301	125478592	6.17229	4.34582	1.71923	ENSG00000119487:intron	ENSG00000243845	125512901-125513207	-	34572
9	125707751	125708043	293	125707776	4.32168	3.32656	0.59373	ENSG00000119487:Promoter	ENSG00000119487	125437392-125707234	-	-662
9	127005413	127005659	247	127005516	6.00061	3.79022	1.57469	ENSG00000136828:intron	ENSG00000136828	126914773-127223166	+	90762
9	129053718	129054115	398	129053992	3.3168	2.90065	0.30123	ENSG00000148343:intron	ENSG00000148343	129036620-129072082	+	17296
9	131622096	131622330	235	131622286	4.84177	3.65305	0.77787	ENSG00000107263:intron	ENSG00000283526	131545513-131558620	+	76699
9	133225653	133225896	244	133225810	3.63193	3.10837	0.30123	ENSG00000119440:intron	ENSG00000119440	133224904-133228591	-	2817
9	136882569	136882916	348	136882719	25.82664	9.62988	19.99835	intergenic	ENSG00000127191	136881911-136926607	+	831
X	2155320	2155706	387	2155587	5.39424	4.00432	1.09512	intergenic	ENSG00000223571	2334294-2336410	-	180897

X	2855459	2855726	268	2855666	5.39424	4.00432	1.09512	ENSG00000235483:Promoter :ENSG00000056998:intron	ENSG00000235483	2852739-2853760	-	-1832
X	9829601	9829876	276	9829711	9.09099	5.60604	4.18865	ENSG00000146950:intron	ENSG00000215319	9818238-9819469	-	-10269
X	13653051	13653317	267	13653128	6.89723	4.46128	2.29209	intergenic	ENSG00000176896	13653188-13681964	+	-4
X	16958520	16958785	266	16958678	4.07388	3.28775	0.47041	ENSG00000169891:intron	ENSG00000169891	16946690-17153280	+	11962
X	17773784	17773999	216	17773906	4.00982	3.24654	0.45978	intergenic	ENSG00000227873	17769004-17769398	-	-4493
X	19431570	19431792	223	19431644	3.77589	3.20345	0.30123	ENSG00000180815:intron	ENSG00000202144	19376773-19376875	+	54907
X	19839867	19840106	240	19839943	4.1849	3.24157	0.52478	ENSG00000147010:intron	ENSG00000147010	19533974-19887601	-	47615
X	19847090	19847441	352	19847145	4.56295	3.60388	0.59373	ENSG00000147010:intron	ENSG00000147010	19533974-19887601	-	40336
X	20516226	20516549	324	20516340	7.37304	4.74897	2.68538	intergenic	ENSG00000252978	20452107-20452431	+	64280
X	21926849	21927188	340	21926974	10.09471	6.00647	5.09428	intergenic	ENSG00000271286	21939460-21940160	+	-12442
X	29443797	29444230	434	29443924	3.34993	2.92244	0.30123	ENSG00000169306:intron	ENSG00000227393	29369000-29369845	-	-74168
X	39855781	39856038	258	39855863	6.26578	4.40475	1.75945	intergenic	ENSG00000261435	39837535-39848358	-	-7551
X	45758895	45759352	458	45759220	7.17425	4.80518	2.52654	ENSG00000270069:intron	ENSG00000269902	45764771-45765299	-	6176
X	49079865	49080087	223	49079948	7.87837	4.69115	3.14537	ENSG00000196998:intron	ENSG00000206936	49082027-49082165	+	-2051
X	65596316	65596633	318	65596475	16.31852	7.93598	10.84035	ENSG00000147065:intron	ENSG00000147065	65588376-65741931	+	8098
X	71628060	71628514	455	71628132	4.07388	3.28775	0.47041	intergenic	ENSG00000186810	71615915-71618517	-	-9769
X	72018511	72018736	226	72018646	7.49188	4.02246	2.78905	ENSG00000204131:intron	ENSG00000196933	72044544-72044892	+	-25921
X	78065036	78065340	305	78065155	8.00408	5.13597	3.2614	ENSG00000102144:intron	ENSG00000187325	78129747-78139706	-	74518
X	94499331	94499583	253	94499470	5.36277	3.98422	1.09512	intergenic	ENSG00000232293	94636662-94639570	-	140113
X	95814678	95814892	215	95814754	3.77589	3.20345	0.30123	intergenic	ENSG00000270583	95720242-95721144	+	94542
X	95827263	95827598	336	95827543	4.07388	3.28775	0.47041	intergenic	ENSG00000270583	95720242-95721144	+	107188
X	108159099	108159315	217	108159110	4.08844	3.29712	0.48089	ENSG00000197565:intron	ENSG00000101844	108091667-108154671	+	67539
X	109271029	109271356	328	109271207	4.91514	3.56573	0.82412	intergenic	ENSG00000215000	109341812-109342697	+	-70620
X	112225677	112225962	286	112225826	4.84177	3.65305	0.77787	intergenic	ENSG00000271464	112319951-112320417	-	94598
X	112276336	112276718	383	112276413	4.07388	3.28775	0.47041	intergenic	ENSG00000271464	112319951-112320417	-	43890
X	116797134	116797386	253	116797276	4.56295	3.60388	0.59373	intergenic	ENSG00000224217	116748529-116748877	-	-48382
X	118664862	118665202	341	118665000	3.77589	3.20345	0.30123	ENSG00000147251:intron	ENSG00000230399	118632374-118634218	-	-30813
X	118678615	118678929	315	118678907	3.34993	2.92244	0.30123	ENSG00000147251:intron	ENSG00000230399	118632374-118634218	-	-44553
X	118679658	118679968	311	118679805	4.56295	3.60388	0.59373	ENSG00000147251:intron	ENSG00000230399	118632374-118634218	-	-45594
X	118664862	118665202	341	118665000	3.77589	3.20345	0.30123	ENSG00000147251:intron	ENSG00000230399	118632374-118634218	-	-30813
X	118678615	118678929	315	118678907	3.34993	2.92244	0.30123	ENSG00000147251:intron	ENSG00000230399	118632374-118634218	-	-44553
X	118679658	118679968	311	118679805	4.56295	3.60388	0.59373	ENSG00000147251:intron	ENSG00000230399	118632374-118634218	-	-45594
X	118664862	118665202	341	118665000	3.77589	3.20345	0.30123	ENSG00000147251:intron	ENSG00000230399	118632374-118634218	-	-30813
X	118678615	118678929	315	118678907	3.34993	2.92244	0.30123	ENSG00000147251:intron	ENSG00000230399	118632374-118634218	-	-44553
X	118679658	118679968	311	118679805	4.56295	3.60388	0.59373	ENSG00000147251:intron	ENSG00000230399	118632374-118634218	-	-45594
X	119514744	119515043	300	119514778	5.92936	4.19316	1.51421	intergenic	ENSG00000224281	119466033-119469098	-	-45795
X	120401736	120401985	250	120401829	4.56295	3.60388	0.59373	intergenic	ENSG00000101892	120362084-120383249	+	39776
X	122783326	122783618	293	122783361	4.84177	3.65305	0.77787	intergenic	ENSG00000212321	122840425-122840645	+	-56953
X	124064087	124064314	228	124064190	4.56295	3.60388	0.59373	ENSG00000101972:intron	ENSG00000232412	123959232-123961341	-	-102859
X	124169184	124169409	226	124169286	5.15789	3.85362	1.00126	ENSG00000101972:intron	ENSG00000237426	124173000-124174459	-	5163
X	135532029	135532318	290	135532137	6.00239	4.23898	1.57533	ENSG00000165359:intron	ENSG00000225235	135520082-135520674	-	-11499
X	135741517	135741782	266	135741640	3.93344	3.19748	0.41301	intergenic	ENSG00000233861	135730732-135732197	-	-9452
X	136477029	136477233	205	136477195	4.06198	3.28009	0.47041	intergenic	ENSG00000102239	136487886-136493780	+	-10755
X	139415940	139416385	446	139415996	3.77589	3.20345	0.30123	intergenic	ENSG00000232549	139446890-139447676	+	-30728
X	139738652	139738875	224	139738777	6.88374	4.62434	2.28385	ENSG00000101974:intron	ENSG00000101977	139581769-139708227	-	-30536
X	139979252	139979480	229	139979411	5.82254	4.12626	1.42733	intergenic	ENSG00000203933	139955724-139965520	-	-13845
X	142039875	142040299	425	142039975	4.56295	3.60388	0.59373	intergenic	ENSG00000236205	142147861-142148866	-	108779
X	142158929	142159146	218	142159007	3.03786	2.80302	0.15111	intergenic	ENSG00000236205	142147861-142148866	-	-10171
X	148014664	148014903	240	148014800	4.56295	3.60388	0.59373	ENSG00000176988:intron	ENSG00000201285	148008099-148008215	-	-6568
X	148247244	148247553	310	148247534	3.49438	2.91742	0.30123	intergenic	ENSG00000233467	148198013-148198878	-	-48520

X	151968797	151969102	306	151969053	3.69765	3.15177	0.30123	ENSG00000102287:intron	ENSG00000102287	151953123-151974680	-	5731
Y	56763398	56763606	209	56763473	9.09099	5.60604	4.18865	intergenic	ENSG00000235857	56855243-56855488	+	-91741
Y	56822614	56822836	223	56822731	3.49438	2.91742	0.30123	intergenic	ENSG00000235857	56855243-56855488	+	-32518
Y	56763398	56763606	209	56763473	9.09099	5.60604	4.18865	intergenic	ENSG00000235857	56855243-56855488	+	-91741
Y	56822614	56822836	223	56822731	3.49438	2.91742	0.30123	intergenic	ENSG00000235857	56855243-56855488	+	-32518
