# Supplementary material for: JunB is a key regulator of multiple myeloma bone marrow angiogenesis
Source: Leukemia. 2021 May 18;35(12):3509–25. doi: 10.1038/s41375-021-01271-9 (PMC8632680; doi:10.1038/s41375-021-01271-9)
Supplement: Supplementary file 3 — Supplementary Table 5 (Unique JunB binding targets identified by ChIP-seq.) [file 41375_2021_1271_MOESM3_ESM.pdf]

Ensembl Gene ID	Gene ID	Tax ID	Gene Symbol	Description
ENSG00000144452	26154	H. sapiens	ABCA12	ATP binding cassette subfamily A member 12
ENSG00000153093	55289	H. sapiens	ACOXL	acyl-CoA oxidase like
ENSG00000183760	390928	H. sapiens	ACP7	acid phosphatase 7, tartrate resistant (putative)
ENSG00000164398	23305	H. sapiens	ACSL6	acyl-CoA synthetase long chain family member 6
ENSG00000170634	98	H. sapiens	ACYP2	acylphosphatase 2
ENSG00000196839	100	H. sapiens	ADA	adenosine deaminase
ENSG00000267511	100422407	H. sapiens	ADAD1P2	adenosine deaminase domain containing 1 pseudogene 2
ENSG00000104755	2515	H. sapiens	ADAM2	ADAM metallopeptidase domain 2
ENSG00000114948	8745	H. sapiens	ADAM23	ADAM metallopeptidase domain 23
ENSG00000218052	642935	H. sapiens	ADAMTS7P4	ADAMTS7 pseudogene 4
ENSG00000173175	111	H. sapiens	ADCY5	adenylate cyclase 5
ENSG00000128271	135	H. sapiens	ADORA2A	adenosine A2a receptor
ENSG00000170425	136	H. sapiens	ADORA2B	adenosine A2b receptor
ENSG00000170214	147	H. sapiens	ADRA1B	adrenoceptor alpha 1B
ENSG00000169252	154	H. sapiens	ADRB2	adrenoceptor beta 2
ENSG00000157510	134265	H. sapiens	AFAP1L1	actin filament associated protein 1 like 1
ENSG00000230114	101929721	H. sapiens	AGBL4-AS1	AGBL4 antisense RNA 1
ENSG00000026652	56895	H. sapiens	AGPAT4	1-acylglycerol-3-phosphate O-acyltransferase 4
ENSG00000233955	402093	H. sapiens	AHCYP3	adenosylhomocysteinase pseudogene 3
ENSG00000124942	79026	H. sapiens	AHNAK	AHNAK nucleoprotein
ENSG00000163568	9447	H. sapiens	AIM2	absent in melanoma 2
ENSG00000256614	100132086	H. sapiens	AK6P1	adenylate kinase 6 pseudogene 1
ENSG00000106948	80709	H. sapiens	AKNA	AT-hook transcription factor
ENSG00000166025	154810	H. sapiens	AMOTL1	angiomotin like 1
ENSG00000174945	155185	H. sapiens	AMZ1	archaelysin family metallopeptidase 1
ENSG00000151687	150709	H. sapiens	ANKAR	ankyrin and armadillo repeat containing
ENSG00000254996	404734	H. sapiens	ANKHD1-EIF4EBP3	ANKHD1-EIF4EBP3 readthrough
ENSG00000206560	23243	H. sapiens	ANKRD28	ankyrin repeat domain 28
ENSG00000135976	375248	H. sapiens	ANKRD36	ankyrin repeat domain 36
ENSG00000151458	57182	H. sapiens	ANKRD50	ankyrin repeat domain 50
ENSG00000135046	301	H. sapiens	ANXA1	annexin A1
ENSG00000122359	311	H. sapiens	ANXA11	annexin A11
ENSG00000148120	84909	H. sapiens	AOPEP	aminopeptidase O (putative)
ENSG00000134817	187	H. sapiens	APLNR	apelin receptor

ENSG00000179750	9582	H. sapiens	APOBEC3B	apolipoprotein B mRNA editing enzyme catalytic subunit 3B
ENSG00000146376	93663	H. sapiens	ARHGAP18	Rho GTPase activating protein 18
ENSG00000215769	109286553	H. sapiens	ARHGAP27P1-BPTFP1-KPNA2P3	ARHGAP27P1-BPTFP1-KPNA2P3 readthrough, transcribed pseudogene
ENSG00000224891	107985793	H. sapiens	ARL14EPP1	ARL14EP pseudogene 1
ENSG00000114098	25852	H. sapiens	ARMC8	armadillo repeat containing 8
ENSG00000153317	50807	H. sapiens	ASAP1	ArfGAP with SH3 domain, ankyrin repeat and PH domain 1
ENSG00000168010	89849	H. sapiens	ATG16L2	autophagy related 16 like 2
ENSG00000101844	115201	H. sapiens	ATG4A	autophagy related 4A cysteine peptidase
ENSG00000101892	23439	H. sapiens	ATP1B4	ATPase Na+/K+ transporting family member beta 4
ENSG00000271614	338758	H. sapiens	ATP2B1-AS1	ATP2B1 antisense RNA 1
ENSG00000058668	493	H. sapiens	ATP2B4	ATPase plasma membrane Ca2+ transporting 4
ENSG00000017260	27032	H. sapiens	ATP2C1	ATPase secretory pathway Ca2+ transporting 1
ENSG00000186009	496	H. sapiens	ATP4B	ATPase H+/K+ transporting subunit beta
ENSG00000214025	100422514	H. sapiens	ATP5PBP4	ATP synthase peripheral stalk-membrane subunit b pseudogene 4
ENSG00000205464	92270	H. sapiens	ATP6AP1L	ATPase H+ transporting accessory protein 1 like
ENSG00000171130	155066	H. sapiens	ATP6V0E2	ATPase H+ transporting V0 subunit e2
ENSG00000169255	8706	H. sapiens	B3GALNT1	beta-1,3-N-acetylgalactosaminyltransferase 1 (globoside blood group)
ENSG00000162630	8707	H. sapiens	B3GALT2	beta-1,3-galactosyltransferase 2
ENSG00000135454	2583	H. sapiens	B4GALNT1	beta-1,4-N-acetyl-galactosaminyltransferase 1
ENSG00000153064	55024	H. sapiens	BANK1	B cell scaffold protein with ankyrin repeats 1
ENSG00000105327	27113	H. sapiens	BBC3	BCL2 binding component 3
ENSG00000140463	585	H. sapiens	BBS4	Bardet-Biedl syndrome 4
ENSG00000060982	586	H. sapiens	BCAT1	branched chain amino acid transaminase 1
ENSG00000114200	590	H. sapiens	BCHE	butyrylcholinesterase

ENSG00000116128	607	H. sapiens	BCL9	BCL9 transcription coactivator
ENSG00000188848	389206	H. sapiens	BEND4	BEN domain containing 4
ENSG00000105829	10282	H. sapiens	BET1	Bet1 golgi vesicular membrane trafficking protein
ENSG00000132840	23743	H. sapiens	BHMT2	betaine--homocysteine S-methyltransferase 2
ENSG00000100290	638	H. sapiens	BIK	BCL2 interacting killer
ENSG00000110934	51411	H. sapiens	BIN2	bridging integrator 2
ENSG00000104081	90427	H. sapiens	BMF	Bcl2 modifying factor
ENSG00000164603	154743	H. sapiens	BMT2	base methyltransferase of 25S rRNA 2 homolog
ENSG00000269055	106481693	H. sapiens	BNIP3P18	BCL2 interacting protein 3 pseudogene 18
ENSG00000269431	100421710	H. sapiens	BNIP3P8	BCL2 interacting protein 3 pseudogene 8
ENSG00000189325	389384	H. sapiens	BNIP5	BCL2 interacting protein 5
ENSG00000157764	673	H. sapiens	BRAF	B-Raf proto-oncogene, serine/threonine kinase
ENSG00000102239	680	H. sapiens	BRS3	bombesin receptor subtype 3
ENSG00000262609	100132641	H. sapiens	BTF3P14	basic transcription factor 3 pseudogene 14
ENSG00000112578	705	H. sapiens	BYSL	bystin like
ENSG00000143633	128061	H. sapiens	C1orf131	chromosome 1 open reading frame 131
ENSG00000240770	246312	H. sapiens	C21orf91-OT1	C21orf91 overlapping transcript 1
ENSG00000171860	719	H. sapiens	C3AR1	complement C3a receptor 1
ENSG00000203711	100130967	H. sapiens	C6orf99	chromosome 6 putative open reading frame 99
ENSG00000221845	401335	H. sapiens	C7orf65	chromosome 7 putative open reading frame 65
ENSG00000141837	773	H. sapiens	CACNA1A	calcium voltage-gated channel subunit alpha1 A
ENSG00000182389	785	H. sapiens	CACNB4	calcium voltage-gated channel auxiliary subunit beta 4
ENSG00000183128	119395	H. sapiens	CALHM3	calcium homeostasis modulator 3
ENSG00000240809	401076	H. sapiens	CAP1P1	CAP1 pseudogene 1
ENSG00000112186	10486	H. sapiens	CAP2	cyclase associated actin cytoskeleton regulatory protein 2
ENSG00000162909	824	H. sapiens	CAPN2	calpain 2
ENSG00000110619	833	H. sapiens	CARS1	cysteinyl-tRNA synthetase 1
ENSG00000164326	9607	H. sapiens	CARTPT	CART prepropeptide
ENSG00000257859	101929110	H. sapiens	CASC18	cancer susceptibility 18

ENSG00000067955	865	H. sapiens	CBFB	core-binding factor subunit beta
ENSG00000105879	79872	H. sapiens	CBLL1	Cbl proto-oncogene like 1
ENSG00000203799	221262	H. sapiens	CCDC162P	coiled-coil domain containing 162, pseudogene
ENSG00000198937	154467	H. sapiens	CCDC167	coiled-coil domain containing 167
ENSG00000108702	6346	H. sapiens	CCL1	C-C motif chemokine ligand 1
ENSG00000160791	1234	H. sapiens	CCR5	C-C motif chemokine receptor 5
ENSG00000249958	100288772	H. sapiens	CCT7P2	chaperonin containing TCP1 subunit 7 pseudogene 2
ENSG00000134061	4064	H. sapiens	CD180	CD180 molecule
ENSG00000158477	909	H. sapiens	CD1A	CD1a molecule
ENSG00000186074	146722	H. sapiens	CD300LF	CD300 molecule like family member f
ENSG00000026508	960	H. sapiens	CD44	CD44 molecule (Indian blood group)
ENSG00000196776	961	H. sapiens	CD47	CD47 molecule
ENSG00000196352	1604	H. sapiens	CD55	CD55 molecule (Cromer blood group)
ENSG00000158402	995	H. sapiens	CDC25C	cell division cycle 25C
ENSG00000226289	100420558	H. sapiens	CDCA4P3	cell division cycle associated 4 pseudogene 3
ENSG00000113100	1007	H. sapiens	CDH9	cadherin 9
ENSG00000136861	55755	H. sapiens	CDK5RAP2	CDK5 regulatory subunit associated protein 2
ENSG00000135048	23670	H. sapiens	CEMIP2	cell migration inducing hyaluronidase 2
ENSG00000226982	1061	H. sapiens	CENPCP1	centromere protein C pseudogene 1
ENSG00000188452	375298	H. sapiens	CERKL	ceramide kinase like
ENSG00000172292	253782	H. sapiens	CERS6	ceramide synthase 6
ENSG00000188596	144535	H. sapiens	CFAP54	cilia and flagella associated protein 54
ENSG00000183833	89876	H. sapiens	CFAP91	cilia and flagella associated protein 91
ENSG00000177200	80205	H. sapiens	CHD9	chromodomain helicase DNA binding protein 9
ENSG00000128656	1123	H. sapiens	CHN1	chimerin 1
ENSG00000182022	51363	H. sapiens	CHST15	carbohydrate sulfotransferase 15
ENSG00000131873	22856	H. sapiens	CHSY1	chondroitin sulfate synthase 1
ENSG00000179583	4261	H. sapiens	CIITA	class II major histocompatibility complex transactivator
ENSG00000159212	54102	H. sapiens	CLIC6	chloride intracellular channel 6
ENSG00000165959	79789	H. sapiens	CLMN	calmin

ENSG00000196748	389383	H. sapiens	CLPSL2	colipase like 2
ENSG00000120885	1191	H. sapiens	CLU	clusterin
ENSG00000130176	1264	H. sapiens	CNN1	calponin 1
ENSG00000119946	26507	H. sapiens	CNNM1	cyclin and CBS domain divalent metal cation transport mediator 1
ENSG00000113805	5067	H. sapiens	CNTN3	contactin 3
ENSG00000236795	101928700	H. sapiens	CNTNAP2-AS1	CNTNAP2 antisense RNA 1
ENSG00000172752	256076	H. sapiens	COL6A5	collagen type VI alpha 5 chain
ENSG00000206384	131873	H. sapiens	COL6A6	collagen type VI alpha 6 chain
ENSG00000198756	23127	H. sapiens	COLGALT2	collagen beta(1-O)galactosyltransferase 2
ENSG00000236041	106481684	H. sapiens	COX6CP18	cytochrome c oxidase subunit 6C pseudogene 18
ENSG00000196353	131034	H. sapiens	CPNE4	copine 4
ENSG00000117322	1380	H. sapiens	CR2	complement C3d receptor 2
ENSG00000143536	49860	H. sapiens	CRNN	cornulin
ENSG00000132693	1401	H. sapiens	CRP	C-reactive protein
ENSG00000109943	56253	H. sapiens	CRTAM	cytotoxic and regulatory T cell molecule
ENSG00000100122	1414	H. sapiens	CRYBB1	crystallin beta B1
ENSG00000178662	80034	H. sapiens	CSRNP3	cysteine and serine rich nuclear protein 3
ENSG00000230013	107987106	H. sapiens	CT70	cancer/testis associated transcript 70
ENSG00000163599	1493	H. sapiens	CTLA4	cytotoxic T-lymphocyte associated protein 4
ENSG00000198730	9646	H. sapiens	CTR9	CTR9 homolog, Paf1/RNA polymerase II complex component
ENSG00000109861	1075	H. sapiens	CTSC	cathepsin C
ENSG00000107611	8029	H. sapiens	CUBN	cubilin
ENSG00000257923	1523	H. sapiens	CUX1	cut like homeobox 1
ENSG00000163510	57703	H. sapiens	CWC22	CWC22 spliceosome associated protein homolog
ENSG00000152404	143884	H. sapiens	CWF19L2	CWF19 like cell cycle control factor 2
ENSG00000180871	3579	H. sapiens	CXCR2	C-X-C motif chemokine receptor 2
ENSG00000186810	2833	H. sapiens	CXCR3	C-X-C motif chemokine receptor 3
ENSG00000203933	347487	H. sapiens	CXorf66	chromosome X open reading frame 66
ENSG00000172115	54205	H. sapiens	CYCS	cytochrome c, somatic
ENSG00000019186	1591	H. sapiens	CYP24A1	cytochrome P450 family 24 subfamily A member 1
ENSG00000197838	1553	H. sapiens	CYP2A13	cytochrome P450 family 2 subfamily A member 13

ENSG00000226450	1568	H. sapiens	CYP2D8P	ccytochrome P450 family 2 subfamily D member 8, pseudogene
ENSG00000186104	120227	H. sapiens	CYP2R1	cytochrome P450 family 2 subfamily R member 1
ENSG00000186204	66002	H. sapiens	CYP4F12	cytochrome P450 family 4 subfamily F member 12
ENSG00000273330	100422235	H. sapiens	CYP4F36P	cytochrome P450 family 4 subfamily F member 36, pseudogene
ENSG00000271286	100418871	H. sapiens	CYTH1P1	CYTH1 pseudogene 1
ENSG00000170891	54360	H. sapiens	CYTL1	cytokine like 1
ENSG00000226950	57291	H. sapiens	DANCR	differentiation antagonizing non-protein coding RNA
ENSG00000112977	1611	H. sapiens	DAP	death associated protein
ENSG00000251380	140947	H. sapiens	DCANP1	dendritic cell associated nuclear protein
ENSG00000213722	23564	H. sapiens	DDAH2	dimethylarginine dimethylaminohydrolase 2
ENSG00000270763	100533714	H. sapiens	DDX43P2	DEAD-box helicase 43 pseudogene 2
ENSG00000160570	162989	H. sapiens	DEDD2	death effector domain containing 2
ENSG00000121644	51029	H. sapiens	DESI2	desumoylating isopeptidase 2
ENSG00000058866	1608	H. sapiens	DGKG	diacylglycerol kinase gamma
ENSG00000067596	1659	H. sapiens	DHX8	DEAH-box helicase 8
ENSG00000143006	63948	H. sapiens	DMRTB1	DMRT like family B with proline rich C-terminal 1
ENSG00000114841	25981	H. sapiens	DNAH1	dynein axonemal heavy chain 1
ENSG00000105877	8701	H. sapiens	DNAH11	dynein axonemal heavy chain 11
ENSG00000105993	10049	H. sapiens	DNAJB6	DnaJ heat shock protein family (Hsp40) member B6
ENSG00000168724	134218	H. sapiens	DNAJC21	DnaJ heat shock protein family (Hsp40) member C21
ENSG00000130226	1804	H. sapiens	DPP6	dipeptidyl peptidase like 6
ENSG00000231839	100420073	H. sapiens	DPY19L4P2	DPY19L4 pseudogene 2
ENSG00000113657	1809	H. sapiens	DPYSL3	dihydropyrimidinase like 3
ENSG00000149295	1813	H. sapiens	DRD2	dopamine receptor D2
ENSG00000235123	100506492	H. sapiens	DSCAM-AS1	DSCAM antisense RNA 1
ENSG00000079393	51207	H. sapiens	DUSP13	dual specificity phosphatase 13
ENSG00000184545	1850	H. sapiens	DUSP8	dual specificity phosphatase 8
ENSG00000280457	728410	H. sapiens	DUX4L2	double homeobox 4 like 2 (pseudogene)

ENSG00000248820	100288885	H. sapiens	DYNLL1P6	dynein light chain LC8-type 1 pseudogene 6
ENSG00000157540	1859	H. sapiens	DYRK1A	dual specificity tyrosine phosphorylation regulated kinase 1A
ENSG00000231105	100506801	H. sapiens	ECE1-AS1	ECE1 antisense RNA 1
ENSG00000236991	101927983	H. sapiens	EDRF1-AS1	EDRF1 antisense RNA 1
ENSG00000096093	114327	H. sapiens	EFHC1	EF-hand domain containing 1
ENSG00000132294	23167	H. sapiens	EFR3A	EFR3 homolog A
ENSG00000224057	100507500	H. sapiens	EGFR-AS1	EGFR antisense RNA 1
ENSG00000013016	30845	H. sapiens	EHD3	EH domain containing 3
ENSG00000236357	100129423	H. sapiens	EI24P1	EI24 pseudogene 1
ENSG00000234028	101928403	H. sapiens	EIF2AK3-DT	EIF2AK3 divergent transcript
ENSG00000277998	100462836	H. sapiens	EIF3EP3	eukaryotic translation initiation factor 3 subunit E pseudogene 3
ENSG00000237064	442720	H. sapiens	EIF3IP1	eukaryotic translation initiation factor 3 subunit I pseudogene 1
ENSG00000233544	100129045	H. sapiens	EIF3KP2	eukaryotic translation initiation factor 3 subunit K pseudogene 2
ENSG00000163577	56648	H. sapiens	EIF5A2	eukaryotic translation initiation factor 5A2
ENSG00000111145	2004	H. sapiens	ELK3	ETS transcription factor ELK3
ENSG00000224101	100861514	H. sapiens	ELMO1-AS1	ELMO1 antisense RNA 1
ENSG00000070061	8518	H. sapiens	ELP1	elongator complex protein 1
ENSG00000170571	133418	H. sapiens	EMB	embigin
ENSG00000086289	54749	H. sapiens	EPDR1	ependymin related 1
ENSG00000154928	2047	H. sapiens	EPHB1	EPH receptor B1
ENSG00000083782	1833	H. sapiens	EPYC	epiphycan
ENSG00000175595	2072	H. sapiens	ERCC4	ERCC excision repair 4, endonuclease catalytic subunit
ENSG00000178607	2081	H. sapiens	ERN1	endoplasmic reticulum to nucleus signaling 1
ENSG00000164283	11082	H. sapiens	ESM1	endothelial cell specific molecule 1
ENSG00000265992	790952	H. sapiens	ESRG	embryonic stem cell related
ENSG00000158220	83850	H. sapiens	ESYT3	extended synaptotagmin 3
ENSG00000143971	54465	H. sapiens	ETAA1	ETAA1 activator of ATR kinase
ENSG00000107371	51010	H. sapiens	EXOSC3	exosome component 3
ENSG00000112319	2070	H. sapiens	EYA4	EYA transcriptional coactivator and phosphatase 4

ENSG00000163586	2168	H. sapiens	FABP1	fatty acid binding protein 1
ENSG00000234545	257415	H. sapiens	FAM133B	family with sequence similarity 133 member B
ENSG00000147724	51059	H. sapiens	FAM135B	family with sequence similarity 135 member B
ENSG00000236438	728262	H. sapiens	FAM157A	family with sequence similarity 157 member A
ENSG00000164142	729830	H. sapiens	FAM160A1	family with sequence similarity 160 member A1
ENSG00000243062	148753	H. sapiens	FAM163A	family with sequence similarity 163 member A
ENSG00000164556	340286	H. sapiens	FAM183BP	acyloxyacyl hydrolase (neutrophil)
ENSG00000111879	79632	H. sapiens	FAM184A	family with sequence similarity 184 member A
ENSG00000135063	9413	H. sapiens	FAM189A2	family with sequence similarity 189 member A2
ENSG00000189157	100129583	H. sapiens	FAM47E	family with sequence similarity 47 member E
ENSG00000180219	196472	H. sapiens	FAM71C	family with sequence similarity 71 member C
ENSG00000203780	92565	H. sapiens	FANK1	fibronectin type III and ankyrin repeat domains 1
ENSG00000146267	84553	H. sapiens	FAXC	failed axon connections homolog, metaxin like GST domain containing
ENSG00000239997	100128733	H. sapiens	FCF1P3	FCF1 pseudogene 3
ENSG00000146192	221472	H. sapiens	FGD2	FYVE, RhoGEF and PH domain containing 2
ENSG00000231426	100132735	H. sapiens	FILNC1	FOXO induced long non-coding RNA 1
ENSG00000229515	391533	H. sapiens	FLT1P1	FLT1 pseudogene 1
ENSG00000075420	64778	H. sapiens	FNDC3B	fibronectin type III domain containing 3B
ENSG00000075426	2355	H. sapiens	FOSL2	FOS like 2, AP-1 transcription factor subunit
ENSG00000054598	2296	H. sapiens	FOXC1	forkhead box C1
ENSG00000118689	2309	H. sapiens	FOXO3	forkhead box O3
ENSG00000179772	2307	H. sapiens	FOXS1	forkhead box S1
ENSG00000282826	100289097	H. sapiens	FRG1CP	FSHD region gene 1 family member C, pseudogene
ENSG00000151474	55691	H. sapiens	FRMD4A	FERM domain containing 4A
ENSG00000087086	2512	H. sapiens	FTL	ferritin light chain
ENSG00000082074	2533	H. sapiens	FYB1	FYN binding protein 1
ENSG00000122068	84248	H. sapiens	FYTTD1	forty-two-three domain containing 1
ENSG00000123689	50486	H. sapiens	G0S2	G0/G1 switch 2
ENSG00000235651	100422338	H. sapiens	G3BP1P1	G3BP1 pseudogene 1

ENSG00000102287	2564	H. sapiens	GABRE	gamma-aminobutyric acid type A receptor subunit epsilon
ENSG00000143641	2590	H. sapiens	GALNT2	polypeptide N-acetylgalactosaminyltransferase 2
ENSG00000115339	2591	H. sapiens	GALNT3	polypeptide N-acetylgalactosaminyltransferase 3
ENSG00000117228	2633	H. sapiens	GBP1	guanylate binding protein 1
ENSG00000115263	2641	H. sapiens	GCG	glucagon
ENSG00000100031	2678	H. sapiens	GGT1	gamma-glutamyltransferase 1
ENSG00000112964	2690	H. sapiens	GHR	growth hormone receptor
ENSG00000106128	2692	H. sapiens	GHRHR	growth hormone releasing hormone receptor
ENSG00000254765	2754	H. sapiens	GLULP2	glutamate-ammonia ligase pseudogene 2
ENSG00000185838	54584	H. sapiens	GNB1L	G protein subunit beta 1 like
ENSG00000173905	27333	H. sapiens	GOLIM4	golgi integral membrane protein 4
ENSG00000166734	113201	H. sapiens	GOLM2	golgi membrane protein 2
ENSG00000236520	100873972	H. sapiens	GPC6-AS1	GPC6 antisense RNA 1
ENSG00000164850	2852	H. sapiens	GPER1	G protein-coupled estrogen receptor 1
ENSG00000175697	165829	H. sapiens	GPR156	G protein-coupled receptor 156
ENSG00000143147	23432	H. sapiens	GPR161	G protein-coupled receptor 161
ENSG00000185477	285513	H. sapiens	GPRIN3	GPRIN family member 3
ENSG00000141738	2886	H. sapiens	GRB7	growth factor receptor bound protein 7
ENSG00000137106	9380	H. sapiens	GRHPR	glyoxylate and hydroxypyruvate reductase
ENSG00000168959	2915	H. sapiens	GRM5	glutamate metabotropic receptor 5
ENSG00000109519	80273	H. sapiens	GRPEL1	GrpE like 1, mitochondrial
ENSG00000105928	1687	H. sapiens	GSDME	gasdermin E
ENSG00000174156	2940	H. sapiens	GSTA3	glutathione S-transferase alpha 3
ENSG00000232829	344813	H. sapiens	GSTO3P	glutathione S-transferase omega 3, pseudogene
ENSG00000197265	2961	H. sapiens	GTF2E2	general transcription factor IIE subunit 2
ENSG00000077235	2975	H. sapiens	GTF3C1	general transcription factor IIIC subunit 1
ENSG00000124196	149699	H. sapiens	GTSF1L	gametocyte specific factor 1 like

ENSG00000197465	2996	H. sapiens	GYPE	glycophorin E (MNS blood group)
ENSG00000243977	115482685	H. sapiens	H3P13	H3 histone pseudogene 13
ENSG00000251596	3031	H. sapiens	HADHAP1	HADHA pseudogene 1
ENSG00000228789	285834	H. sapiens	HCG22	HLA complex group 22
ENSG00000180353	3059	H. sapiens	HCLS1	hematopoietic cell-specific Lyn substrate 1
ENSG00000233012	100419489	H. sapiens	HDAC1P2	histone deacetylase 1 pseudogene 2
ENSG00000196591	3066	H. sapiens	HDAC2	histone deacetylase 2
ENSG00000173706	57493	H. sapiens	HEG1	heart development protein with EGF like domains 1
ENSG00000187821	391723	H. sapiens	HELT	helt bHLH transcription factor
ENSG00000261418	8918	H. sapiens	HERC2P6	hect domain and RLD 2 pseudogene 6
ENSG00000132297	10086	H. sapiens	HHLA1	HERV-H LTR-associating 1
ENSG00000064393	28996	H. sapiens	HIPK2	homeodomain interacting protein kinase 2
ENSG00000095951	3096	H. sapiens	HIVEP1	HIVEP zinc finger 1
ENSG00000243753	3139	H. sapiens	HLA-L	major histocompatibility complex, class I, L (pseudogene)
ENSG00000228808	100873890	H. sapiens	HMGB3P4	high mobility group box 3 pseudogene 4
ENSG00000213305	100421794	H. sapiens	HNRNPCP6	heterogeneous nuclear ribonucleoprotein C pseudogene 6
ENSG00000228653	100127907	H. sapiens	HNRNPCP7	heterogeneous nuclear ribonucleoprotein C pseudogene 7
ENSG00000120094	3211	H. sapiens	HOXB1	homeobox B1
ENSG00000182601	9951	H. sapiens	HS3ST4	heparan sulfate-glucosamine 3-sulfotransferase 4
ENSG00000157423	54768	H. sapiens	HYDIN	HYDIN axonemal central pair apparatus protein
ENSG00000108622	3384	H. sapiens	ICAM2	intercellular adhesion molecule 2
ENSG00000163565	3428	H. sapiens	IFI16	interferon gamma inducible protein 16
ENSG00000159110	3455	H. sapiens	IFNAR2	interferon alpha and beta receptor subunit 2
ENSG00000159128	3460	H. sapiens	IFNGR2	interferon gamma receptor 2
ENSG00000109083	90410	H. sapiens	IFT20	intraflagellar transport 20
ENSG00000017427	3479	H. sapiens	IGF1	insulin like growth factor 1
ENSG00000197081	3482	H. sapiens	IGF2R	insulin like growth factor 2 receptor

ENSG00000204866	147920	H. sapiens	IGFL2	IGF like family member 2
ENSG00000211890	3494	H. sapiens	IGHA2	immunoglobulin heavy constant alpha 2 (A2m marker)
ENSG00000136634	3586	H. sapiens	IL10	interleukin 10
ENSG00000110324	3587	H. sapiens	IL10RA	interleukin 10 receptor subunit alpha
ENSG00000168811	3592	H. sapiens	IL12A	interleukin 12A
ENSG00000244040	101928376	H. sapiens	IL12A-AS1	IL12A antisense RNA 1
ENSG00000081985	3595	H. sapiens	IL12RB2	interleukin 12 receptor subunit beta 2
ENSG00000112116	112744	H. sapiens	IL17F	interleukin 17F
ENSG00000110944	51561	H. sapiens	IL23A	interleukin 23 subunit alpha
ENSG00000134460	3559	H. sapiens	IL2RA	interleukin 2 receptor subunit alpha
ENSG00000148153	58493	H. sapiens	INIP	INTS3 and NABP interacting protein
ENSG00000109452	8821	H. sapiens	INPP4B	inositol polyphosphate-4-phosphatase type II B
ENSG00000198825	22876	H. sapiens	INPP5F	inositol polyphosphate-5-phosphatase F
ENSG00000173404	3642	H. sapiens	INSM1	INSM transcriptional repressor 1
ENSG00000225235	100874118	H. sapiens	INTS6L-AS1	INTS6L antisense RNA 1
ENSG00000250492	285634	H. sapiens	INTS6P1	integrator complex subunit 6 pseudogene 1
ENSG00000231871	100873949	H. sapiens	IPO9-AS1	IPO9 antisense RNA 1
ENSG00000144711	9922	H. sapiens	IQSEC1	IQ motif and Sec7 domain ArfGEF 1
ENSG00000072952	10335	H. sapiens	IRAG1	inositol 1,4,5-triphosphate receptor associated 1
ENSG00000136003	23479	H. sapiens	ISCU	iron-sulfur cluster assembly enzyme
ENSG00000172183	3669	H. sapiens	ISG20	interferon stimulated exonuclease gene 20
ENSG00000169896	3684	H. sapiens	ITGAM	integrin subunit alpha M
ENSG00000143772	3707	H. sapiens	ITPKB	inositol-trisphosphate 3-kinase B
ENSG00000116679	10625	H. sapiens	IVNS1ABP	influenza virus NS1A binding protein
ENSG00000153814	221895	H. sapiens	JAZF1	JAZF zinc finger 1
ENSG00000235012	729056	H. sapiens	JCADP1	JCAD pseudogene 1
ENSG00000177606	3725	H. sapiens	JUN	Jun proto-oncogene, AP-1 transcription factor subunit
ENSG00000271108	100420841	H. sapiens	KATNBL1P5	katanin regulatory subunit B1 like 1 pseudogene 5
ENSG00000242370	100874084	H. sapiens	KCNAB1-AS1	KCNAB1 antisense RNA 1

ENSG00000129159	3746	H. sapiens	KCNC1	potassium voltage-gated channel subfamily C member 1
ENSG00000184408	3751	H. sapiens	KCND2	potassium voltage-gated channel subfamily D member 2
ENSG00000187486	3767	H. sapiens	KCNJ11	potassium inwardly rectifying channel subfamily J member 11
ENSG00000157542	3763	H. sapiens	KCNJ6	potassium inwardly rectifying channel subfamily J member 6
ENSG00000173338	10089	H. sapiens	KCNK7	potassium two pore domain channel subfamily K member 7
ENSG00000197584	10242	H. sapiens	KCNMB2	potassium calcium-activated channel subfamily M regulatory beta subunit 2
ENSG00000170745	3790	H. sapiens	KCNS3	potassium voltage-gated channel modifier subfamily S member 3
ENSG00000100196	11015	H. sapiens	KDELR3	KDEL endoplasmic reticulum protein retention receptor 3
ENSG00000132510	23135	H. sapiens	KDM6B	lysine demethylase 6B
ENSG00000138182	9585	H. sapiens	KIF20B	kinesin family member 20B
ENSG00000155090	7071	H. sapiens	KLF10	Kruppel like factor 10
ENSG00000067082	1316	H. sapiens	KLF6	Kruppel like factor 6
ENSG00000172578	89857	H. sapiens	KLHL6	kelch like family member 6
ENSG00000214417	392371	H. sapiens	KRT18P13	keratin 18 pseudogene 13
ENSG00000240128	151825	H. sapiens	KRT18P43	keratin 18 pseudogene 43
ENSG00000265480	284085	H. sapiens	KRT18P55	keratin 18 pseudogene 55
ENSG00000249668	106479027	H. sapiens	KRT18P56	keratin 18 pseudogene 56
ENSG00000215208	100418829	H. sapiens	KRT18P60	keratin 18 pseudogene 60
ENSG00000187005	337977	H. sapiens	KRTAP21-1	keratin associated protein 21-1
ENSG00000115919	8942	H. sapiens	KYNU	kynureninase
ENSG00000264707	101927150	H. sapiens	L3MBTL4-AS1	L3MBTL4 antisense RNA 1
ENSG00000078081	27074	H. sapiens	LAMP3	lysosomal associated membrane protein 3
ENSG00000243438	106479034	H. sapiens	LARP1BP2	LARP1B pseudogene 2
ENSG00000271588	106480287	H. sapiens	LARP7P1	LARP7 pseudogene 1
ENSG00000119440	286310	H. sapiens	LCN1P1	lipocalin 1 pseudogene 1
ENSG00000232021	641518	H. sapiens	LEF1-AS1	LEF1 antisense RNA 1
ENSG00000185433	54072	H. sapiens	LINC00158	long intergenic non-protein coding RNA 158
ENSG00000226935	118421	H. sapiens	LINC00161	long intergenic non-protein coding RNA 161
ENSG00000203801	387111	H. sapiens	LINC00222	long intergenic non-protein coding RNA 222

ENSG00000259974	140828	H. sapiens	LINC00261	long intergenic non-protein coding RNA 261
ENSG00000235665	339788	H. sapiens	LINC00298	long intergenic non-protein coding RNA 298
ENSG00000179676	221241	H. sapiens	LINC00305	long intergenic non-protein coding RNA 305
ENSG00000260388	100861549	H. sapiens	LINC00562	long intergenic non-protein coding RNA 562
ENSG00000196243	439916	H. sapiens	LINC00615	long intergenic non-protein coding RNA 615
ENSG00000233746	200261	H. sapiens	LINC00656	long intergenic non-protein coding RNA 656
ENSG00000233570	100996597	H. sapiens	LINC00690	long intergenic non-protein coding RNA 690
ENSG00000245164	100130231	H. sapiens	LINC00861	long intergenic non-protein coding RNA 861
ENSG00000205634	400932	H. sapiens	LINC00898	long intergenic non-protein coding RNA 898
ENSG00000236700	154092	H. sapiens	LINC01010	long intergenic non-protein coding RNA 1010
ENSG00000230426	104169671	H. sapiens	LINC01036	long intergenic non-protein coding RNA 1036
ENSG00000249464	285419	H. sapiens	LINC01091	long intergenic non-protein coding RNA 1091
ENSG00000242512	100996490	H. sapiens	LINC01206	long intergenic non-protein coding RNA 1206
ENSG00000250223	100874275	H. sapiens	LINC01216	long intergenic non-protein coding RNA 1216
ENSG00000250739	101928971	H. sapiens	LINC01262	long intergenic non-protein coding RNA 1262
ENSG00000233077	101927586	H. sapiens	LINC01271	long intergenic non-protein coding RNA 1271
ENSG00000234722	103724390	H. sapiens	LINC01287	long intergenic non-protein coding RNA 1287
ENSG00000205632	100128946	H. sapiens	LINC01310	long intergenic non-protein coding RNA 1310
ENSG00000249343	101929082	H. sapiens	LINC01333	long intergenic non-protein coding RNA 1333
ENSG00000228309	101929093	H. sapiens	LINC01350	long intergenic non-protein coding RNA 1350
ENSG00000251324	104355294	H. sapiens	LINC01386	long intergenic non-protein coding RNA 1386
ENSG00000234380	100506385	H. sapiens	LINC01426	long intergenic non-protein coding RNA 1426
ENSG00000238284	101928795	H. sapiens	LINC01448	long intergenic non-protein coding RNA 1448
ENSG00000249484	101927134	H. sapiens	LINC01470	long intergenic non-protein coding RNA 1470
ENSG00000236849	101927258	H. sapiens	LINC01474	long intergenic non-protein coding RNA 1474

ENSG00000231776	105377880	H. sapiens	LINC01611	long intergenic non-protein coding RNA 1611
ENSG00000257242	256021	H. sapiens	LINC01619	long intergenic non-protein coding RNA 1619
ENSG00000228397	101928043	H. sapiens	LINC01635	long intergenic non-protein coding RNA 1635
ENSG00000227940	105372897	H. sapiens	LINC01696	long intergenic non-protein coding RNA 1696
ENSG00000230324	339568	H. sapiens	LINC01734	long intergenic non-protein coding RNA 1734
ENSG00000236720	101928778	H. sapiens	LINC01741	long intergenic non-protein coding RNA 1741
ENSG00000235054	284661	H. sapiens	LINC01777	long intergenic non-protein coding RNA 1777
ENSG00000231420	105373683	H. sapiens	LINC01817	long intergenic non-protein coding RNA 1817
ENSG00000236914	107984773	H. sapiens	LINC01852	long intergenic non-protein coding RNA 1852
ENSG00000254130	102557615	H. sapiens	LINC01947	long intergenic non-protein coding RNA 1947
ENSG00000230401	105374293	H. sapiens	LINC01972	long intergenic non-protein coding RNA 1972
ENSG00000204283	400624	H. sapiens	LINC01973	long intergenic non-protein coding RNA 1973
ENSG00000242671	101927866	H. sapiens	LINC02010	long intergenic non-protein coding RNA 2010
ENSG00000241544	105374177	H. sapiens	LINC02029	long intergenic non-protein coding RNA 2029
ENSG00000267452	105371828	H. sapiens	LINC02073	long intergenic non-protein coding RNA 2073
ENSG00000267065	101928514	H. sapiens	LINC02080	long intergenic non-protein coding RNA 2080
ENSG00000214407	152225	H. sapiens	LINC02085	long intergenic non-protein coding RNA 2085
ENSG00000272575	105369564	H. sapiens	LINC02098	long intergenic non-protein coding RNA 2098
ENSG00000253490	101929450	H. sapiens	LINC02099	long intergenic non-protein coding RNA 2099
ENSG00000259847	101927650	H. sapiens	LINC02126	long intergenic non-protein coding RNA 2126
ENSG00000260264	105371142	H. sapiens	LINC02194	long intergenic non-protein coding RNA 2194
ENSG00000250708	101928478	H. sapiens	LINC02269	long intergenic non-protein coding RNA 2269
ENSG00000260423	101930452	H. sapiens	LINC02367	long intergenic non-protein coding RNA 2367
ENSG00000257784	101927038	H. sapiens	LINC02400	long intergenic non-protein coding RNA 2400
ENSG00000203585	100507175	H. sapiens	LINC02408	long intergenic non-protein coding RNA 2408

ENSG00000246363	728084	H. sapiens	LINC02458	long intergenic non-protein coding RNA 2458
ENSG00000234956	100507406	H. sapiens	LINC02539	long intergenic non-protein coding RNA 2539
ENSG00000230943	101927686	H. sapiens	LINC02541	long intergenic non-protein coding RNA 2541
ENSG00000256422	102723895	H. sapiens	LINC02552	long intergenic non-protein coding RNA 2552
ENSG00000261780	100505817	H. sapiens	LINC02582	long intergenic non-protein coding RNA 2582
ENSG00000187600	101805491	H. sapiens	LINC02583	long intergenic non-protein coding RNA 2583
ENSG00000226251	101929541	H. sapiens	LINC02608	long intergenic non-protein coding RNA 2608
ENSG00000224788	101928298	H. sapiens	LINC02670	long intergenic non-protein coding RNA 2670
ENSG00000273409	101929497	H. sapiens	LINC02712	long intergenic non-protein coding RNA 2712
ENSG00000256684	105369443	H. sapiens	LINC02737	long intergenic non-protein coding RNA 2737
ENSG00000254968	101928823	H. sapiens	LINC02763	long intergenic non-protein coding RNA 2763
ENSG00000229274	105375009	H. sapiens	LINC02829	long intergenic non-protein coding RNA 2829
ENSG00000285536	105375273	H. sapiens	LINC02838	long intergenic non-protein coding RNA 2838
ENSG00000236436	101928271	H. sapiens	LINC02850	long intergenic non-protein coding RNA 2850
ENSG00000222004	285941	H. sapiens	LINC02860	long intergenic non-protein coding RNA 2860
ENSG00000166035	3990	H. sapiens	LIPC	lipase C, hepatic type
ENSG00000163898	200879	H. sapiens	LIPH	lipase H
ENSG00000143013	8543	H. sapiens	LMO4	LIM domain only 4
ENSG00000162761	4009	H. sapiens	LMX1A	LIM homeobox transcription factor 1 alpha
ENSG00000237396	101929685	H. sapiens	LNCNEF	lncRNA neighboring enhancer of FOXA2
ENSG00000072201	84708	H. sapiens	LNX1	ligand of numb-protein X 1
ENSG00000250812	100128865	H. sapiens	LOC100128865	methyltransferase like 5 pseudogene
ENSG00000238026	100131216	H. sapiens	LOC100131216	transmembrane protein 38B pseudogene
ENSG00000231357	100131264	H. sapiens	LOC100131264	prefoldin subunit 4 pseudogene
ENSG00000258262	100132126	H. sapiens	LOC100132126	mitochondrial carrier 2 pseudogene
ENSG00000261522	100133920	H. sapiens	LOC100133920	methylenetetrahydrofolate dehydrogenase (NADP+ dependent) 1 like pseudogene
ENSG00000251473	100288146	H. sapiens	LOC100288146	nucleoporin Nup43-like

ENSG00000228729	100418874	H. sapiens	LOC100418874	cathepsin B pseudogene
ENSG00000223905	100418965	H. sapiens	LOC100418965	peroxiredoxin 5 pseudogene
ENSG00000236159	100419562	H. sapiens	LOC100419562	ribosomal protein L13 pseudogene
ENSG00000235951	100419685	H. sapiens	LOC100419685	zinc finger protein 555 pseudogene
ENSG00000232327	100419739	H. sapiens	LOC100419739	zinc finger protein 341 pseudogene
ENSG00000258134	100420186	H. sapiens	LOC100420186	WW domain binding protein 2 pseudogene
ENSG00000226671	100420647	H. sapiens	LOC100420647	voltage dependent anion channel 3 pseudogene
ENSG00000254629	100420800	H. sapiens	LOC100420800	suppressor of cytokine signaling 6 pseudogene
ENSG00000270583	100420872	H. sapiens	LOC100420872	ribosome binding factor A (putative) pseudogene
ENSG00000264050	100421028	H. sapiens	LOC100421028	gap junction protein alpha 4 pseudogene
ENSG00000230785	100421782	H. sapiens	LOC100421782	heterogeneous nuclear ribonucleoprotein A1 like 2 pseudogene
ENSG00000236468	100421840	H. sapiens	LOC100421840	eukaryotic translation elongation factor 1 alpha 2 pseudogene
ENSG00000248791	100422687	H. sapiens	LOC100422687	pleckstrin homology domain containing A1 pseudogene
ENSG00000223522	100505716	H. sapiens	LOC100505716	uncharacterized LOC100505716
ENSG00000255521	100507144	H. sapiens	LOC100507144	uncharacterized LOC100507144
ENSG00000236205	100533669	H. sapiens	LOC100533669	MAGE family member A10 pseudogene
ENSG00000257550	100652999	H. sapiens	LOC100652999	uncharacterized LOC100652999
ENSG00000237176	100996696	H. sapiens	LOC100996696	polyadenylate-binding protein 4 pseudogene
ENSG00000257855	101059974	H. sapiens	LOC101059974	p53 and DNA damage-regulated protein 1 pseudogene
ENSG00000258133	101060021	H. sapiens	LOC101060021	ADP-ribosylation factor 1 pseudogene
ENSG00000231252	101926964	H. sapiens	LOC101926964	uncharacterized LOC101926964
ENSG00000238276	101927760	H. sapiens	LOC101927760	uncharacterized LOC101927760
ENSG00000269985	101927972	H. sapiens	LOC101927972	uncharacterized LOC101927972
ENSG00000232412	101928402	H. sapiens	LOC101928402	uncharacterized LOC101928402
ENSG00000258274	101928731	H. sapiens	LOC101928731	uncharacterized LOC101928731

ENSG00000263063	101929552	H. sapiens	LOC101929552	uncharacterized LOC101929552
ENSG00000249635	101929577	H. sapiens	LOC101929577	uncharacterized LOC101929577
ENSG00000253671	101930275	H. sapiens	LOC101930275	uncharacterized LOC101930275
ENSG00000231964	102724323	H. sapiens	LOC102724323	uncharacterized LOC102724323
ENSG00000268650	102725254	H. sapiens	LOC102725254	uncharacterized LOC102725254
ENSG00000285280	105371664	H. sapiens	LOC105371664	uncharacterized LOC105371664
ENSG00000284607	105374988	H. sapiens	LOC105374988	uncharacterized LOC105374988
ENSG00000227131	105375050	H. sapiens	LOC105375050	uncharacterized LOC105375050
ENSG00000230190	105375556	H. sapiens	LOC105375556	uncharacterized LOC105375556
ENSG00000254775	105375861	H. sapiens	LOC105375861	uncharacterized LOC105375861
ENSG00000249184	105377488	H. sapiens	LOC105377488	uncharacterized LOC105377488
ENSG00000259392	107984761	H. sapiens	LOC107984761	microtubule-associated proteins 1A/1B light chain 3 beta 2-like
ENSG00000276871	110467520	H. sapiens	LOC110467520	RNA, 5.8S ribosomal pseudogene
ENSG00000249944	112267949	H. sapiens	LOC112267949	COX assembly mitochondrial protein homolog pseudogene
ENSG00000250547	133332	H. sapiens	LOC133332	mitochondrial ribosomal protein S5 pseudogene
ENSG00000237594	150051	H. sapiens	LOC150051	uncharacterized LOC150051
ENSG00000249476	285638	H. sapiens	LOC285638	uncharacterized LOC285638
ENSG00000261070	338694	H. sapiens	LOC338694	uncharacterized LOC338694
ENSG00000224715	339685	H. sapiens	LOC339685	uncharacterized LOC339685
ENSG00000253571	392180	H. sapiens	LOC392180	transcriptional adaptor 1 pseudogene
ENSG00000262587	554206	H. sapiens	LOC554206	leucine carboxyl methyltransferase 1 pseudogene
ENSG00000248608	645433	H. sapiens	LOC645433	inositol-3-phosphate synthase 1 pseudogene
ENSG00000254471	646112	H. sapiens	LOC646112	origin recognition complex subunit 3 pseudogene
ENSG00000225884	729254	H. sapiens	LOC729254	hCG2045843
ENSG00000240477	730021	H. sapiens	LOC730021	eukaryotic translation initiation factor 3 subunit J pseudogene
ENSG00000134013	4017	H. sapiens	LOXL2	lysyl oxidase like 2
ENSG00000175445	4023	H. sapiens	LPL	lipoprotein lipase

ENSG00000155530	136332	H. sapiens	LRGUK	leucine rich repeats and guanylate kinase domain containing
ENSG00000268324	83598	H. sapiens	LRRC2-AS1	LRRC2 antisense RNA 1
ENSG00000173114	54674	H. sapiens	LRRN3	leucine rich repeat neuronal 3
ENSG00000248323	100505994	H. sapiens	LUCAT1	lung cancer associated transcript 1
ENSG00000261667	101928108	H. sapiens	LY6L	lymphocyte antigen 6 family member L
ENSG00000177383	64110	H. sapiens	MAGEF1	MAGE family member F1
ENSG00000144063	7851	H. sapiens	MALL	mal, T cell differentiation protein like
ENSG00000145050	7873	H. sapiens	MANF	mesencephalic astrocyte derived neurotrophic factor
ENSG00000034152	5606	H. sapiens	MAP2K3	mitogen-activated protein kinase kinase 3
ENSG00000135341	6885	H. sapiens	MAP3K7	mitogen-activated protein kinase kinase kinase 7
ENSG00000071054	9448	H. sapiens	MAP4K4	mitogen-activated protein kinase kinase kinase kinase 4
ENSG00000259438	112543478	H. sapiens	MAPK6-DT	MAPK6 divergent transcript
ENSG00000119487	79109	H. sapiens	MAPKAP1	MAPK associated protein 1
ENSG00000166974	10982	H. sapiens	MAPRE2	microtubule associated protein RP/EB family member 2
ENSG00000248109	105378220	H. sapiens	MARCOL	MARCO like
ENSG00000228815	100631260	H. sapiens	MARK2P13	microtubule affinity regulating kinase 2 pseudogene 13
ENSG00000271642	100421550	H. sapiens	MARK3P1	microtubule affinity regulating kinase 3 pseudogene 1
ENSG00000180611	151963	H. sapiens	MB21D2	Mab-21 domain containing 2
ENSG00000143797	129642	H. sapiens	MBOAT2	membrane bound O-acyltransferase domain containing 2
ENSG00000101977	4168	H. sapiens	MCF2	MCF.2 cell line derived transforming sequence
ENSG00000143384	4170	H. sapiens	MCL1	MCL1 apoptosis regulator, BCL2 family member
ENSG00000082212	4200	H. sapiens	ME2	malic enzyme 2
ENSG00000172878	254042	H. sapiens	METAP1D	methionyl aminopeptidase type 1D, mitochondrial
ENSG00000165171	155368	H. sapiens	METTL27	methyltransferase like 27
ENSG00000197614	8076	H. sapiens	MFAP5	microfibril associated protein 5
ENSG00000164877	79778	H. sapiens	MICALL2	MICAL like 2

ENSG00000148343	84895	H. sapiens	MIGA2	mitoguardin 2
ENSG00000106125	84182	H. sapiens	MINDY4	MINDY lysine 48 deubiquitinase 4
ENSG00000221771	100302161	H. sapiens	MIR1205	microRNA 1205
ENSG00000283429	100422872	H. sapiens	MIR1244-3	microRNA 1244-3
ENSG00000283203	100302142	H. sapiens	MIR1246	microRNA 1246
ENSG00000251862	100616437	H. sapiens	MIR1343	microRNA 1343
ENSG00000207954	406929	H. sapiens	MIR138-1	microRNA 138-1
ENSG00000207814	406939	H. sapiens	MIR147A	microRNA 147a
ENSG00000199085	406940	H. sapiens	MIR148A	microRNA 148a
ENSG00000283218	100302131	H. sapiens	MIR302F	microRNA 302f
ENSG00000264358	100422947	H. sapiens	MIR3122	microRNA 3122
ENSG00000266761	100422889	H. sapiens	MIR3194	microRNA 3194
ENSG00000199104	442911	H. sapiens	MIR346	microRNA 346
ENSG00000264342	100500825	H. sapiens	MIR3660	microRNA 3660
ENSG00000263514	100500879	H. sapiens	MIR3668	microRNA 3668
ENSG00000266458	100422852	H. sapiens	MIR4259	microRNA 4259
ENSG00000265172	100422996	H. sapiens	MIR4262	microRNA 4262
ENSG00000265965	100423027	H. sapiens	MIR4266	microRNA 4266
ENSG00000283420	100422999	H. sapiens	MIR4278	microRNA 4278
ENSG00000265919	100422887	H. sapiens	MIR4280	microRNA 4280
ENSG00000263890	100422924	H. sapiens	MIR4303	microRNA 4303
ENSG00000265957	100422829	H. sapiens	MIR4319	microRNA 4319
ENSG00000283343	100422865	H. sapiens	MIR4320	microRNA 4320
ENSG00000264371	100616365	H. sapiens	MIR4425	microRNA 4425
ENSG00000266705	100616213	H. sapiens	MIR4437	microRNA 4437
ENSG00000276029	100616184	H. sapiens	MIR4477A	microRNA 4477a
ENSG00000266017	100616194	H. sapiens	MIR4477B	microRNA 4477b
ENSG00000283699	100616320	H. sapiens	MIR4481	microRNA 4481
ENSG00000264200	100616457	H. sapiens	MIR4693	microRNA 4693
ENSG00000266593	100616369	H. sapiens	MIR4713	microRNA 4713
ENSG00000265595	100616225	H. sapiens	MIR4756	microRNA 4756
ENSG00000264974	100616395	H. sapiens	MIR4789	microRNA 4789
ENSG00000265699	100616339	H. sapiens	MIR548AE2	microRNA 548ae-2
ENSG00000212036	102465854	H. sapiens	MIR548BA	microRNA 548ba
ENSG00000221442	100313895	H. sapiens	MIR548F4	microRNA 548f-4
ENSG00000263381	100847089	H. sapiens	MIR5584	microRNA 5584
ENSG00000283616	100847034	H. sapiens	MIR5683	microRNA 5683
ENSG00000266099	100847031	H. sapiens	MIR5700	microRNA 5700
ENSG00000263981	100847027	H. sapiens	MIR5705	microRNA 5705
ENSG00000207637	693180	H. sapiens	MIR595	microRNA 595
ENSG00000274115	102466518	H. sapiens	MIR6081	microRNA 6081
ENSG00000276176	102466104	H. sapiens	MIR6090	microRNA 6090
ENSG00000207622	693204	H. sapiens	MIR619	microRNA 619
ENSG00000207965	693214	H. sapiens	MIR629	microRNA 629
ENSG00000284250	102466864	H. sapiens	MIR6516	microRNA 6516
ENSG00000284197	724028	H. sapiens	MIR658	microRNA 658
ENSG00000207574	724031	H. sapiens	MIR661	microRNA 661
ENSG00000275101	102466983	H. sapiens	MIR6766	microRNA 6766
ENSG00000274969	102465462	H. sapiens	MIR6771	microRNA 6771
ENSG00000278420	102465491	H. sapiens	MIR6819	microRNA 6819

ENSG00000283789	102465836	H. sapiens	MIR7846	microRNA 7846
ENSG00000273882	102465868	H. sapiens	MIR8066	microRNA 8066
ENSG00000216192	100126320	H. sapiens	MIR920	microRNA 920
ENSG00000011143	54903	H. sapiens	MKS1	MKS transition zone complex subunit 1
ENSG00000137674	9313	H. sapiens	MMP20	matrix metallopeptidase 20
ENSG00000138722	22915	H. sapiens	MMRN1	multimerin 1
ENSG00000163563	4332	H. sapiens	MNDA	myeloid cell nuclear differentiation antigen
ENSG00000240268	100289017	H. sapiens	MOXD2P	monooxygenase DBH like 2, pseudogene
ENSG00000135324	112609	H. sapiens	MRAP2	melanocortin 2 receptor accessory protein 2
ENSG00000183695	117194	H. sapiens	MRGPRX2	MAS related GPR family member X2
ENSG00000132313	51318	H. sapiens	MRPL35	mitochondrial ribosomal protein L35
ENSG00000175581	51642	H. sapiens	MRPL48	mitochondrial ribosomal protein L48
ENSG00000253814	347704	H. sapiens	MRPS36P3	mitochondrial ribosomal protein S36 pseudogene 3
ENSG00000214782	728588	H. sapiens	MS4A18	membrane spanning 4-domains A18
ENSG00000283601	116435280	H. sapiens	MS4A19P	membrane spanning 4-domains A19, pseudogene
ENSG00000151379	343930	H. sapiens	MSGN1	mesogenin 1
ENSG00000147065	4478	H. sapiens	MSN	moesin
ENSG00000038945	4481	H. sapiens	MSR1	macrophage scavenger receptor 1
ENSG00000125148	4502	H. sapiens	MT2A	metallothionein 2A
ENSG00000137409	23787	H. sapiens	MTCH1	mitochondrial carrier 1
ENSG00000228898	107075173	H. sapiens	MTCO1P43	MT-CO1 pseudogene 43
ENSG00000236007	107075175	H. sapiens	MTCO1P46	MT-CO1 pseudogene 46
ENSG00000229690	140909	H. sapiens	MTCO2P1	MT-CO2 pseudogene 1
ENSG00000240951	107075266	H. sapiens	MTCO2P6	MT-CO2 pseudogene 6
ENSG00000248282	109729154	H. sapiens	MTCYBP44	MT-CYB pseudogene 44
ENSG00000217169	106480237	H. sapiens	MTHFD2P2	methylenetetrahydrofolate dehydrogenase (NADP+ dependent) 2, methenyltetrahydrofolate cyclohydrolase pseudogene 2
ENSG00000014914	10903	H. sapiens	MTMR11	myotubularin related protein 11
ENSG00000253970	100873179	H. sapiens	MTND2P7	MT-ND2 pseudogene 7
ENSG00000130349	51250	H. sapiens	MTRES1	mitochondrial transcription rescue factor 1
ENSG00000255823	100463486	H. sapiens	MTRNR2L8	MT-RNR2 like 8
ENSG00000236242	100885782	H. sapiens	MYO16-AS1	MYO16 antisense RNA 1
ENSG00000230292	100862679	H. sapiens	NAALADL2-AS3	NAALADL2 antisense RNA 3

ENSG00000196531	4666	H. sapiens	NACA	nascent polypeptide associated complex subunit alpha
ENSG00000249229	100132712	H. sapiens	NAMPTP2	nicotinamide phosphoribosyltransferase pseudogene 2
ENSG00000121152	23397	H. sapiens	NCAPH	non-SMC condensin I complex subunit H
ENSG00000166579	81565	H. sapiens	NDEL1	nudE neurodevelopment protein 1 like 1
ENSG00000237406	266625	H. sapiens	NDUFA9P1	NADH:ubiquinone oxidoreductase subunit A9 pseudogene 1
ENSG00000251306	100132036	H. sapiens	NDUFB4P2	NADH:ubiquinone oxidoreductase subunit B4 pseudogene 2
ENSG00000178127	4729	H. sapiens	NDUFV2	NADH:ubiquinone oxidoreductase core subunit V2
ENSG00000245532	283131	H. sapiens	NEAT1	nuclear paraspeckle assembly transcript 1
ENSG00000119408	10783	H. sapiens	NEK6	NIMA related kinase 6
ENSG00000117691	29937	H. sapiens	NENF	neudesin neurotrophic factor
ENSG00000101096	4773	H. sapiens	NFATC2	nuclear factor of activated T cells 2
ENSG00000237853	645030	H. sapiens	NFIA-AS1	NFIA antisense RNA 1
ENSG00000146729	2631	H. sapiens	NIPSNAP2	nipsnap homolog 2
ENSG00000228213	100874010	H. sapiens	NLGN1-AS1	NLGN1 antisense RNA 1
ENSG00000179873	204801	H. sapiens	NLRP11	NLR family pyrin domain containing 11
ENSG00000132911	56923	H. sapiens	NMUR2	neuromedin U receptor 2
ENSG00000198929	9722	H. sapiens	NOS1AP	nitric oxide synthase 1 adaptor protein
ENSG00000185823	23742	H. sapiens	NPAP1	nuclear pore associated protein 1
ENSG00000056291	10886	H. sapiens	NPFFR2	neuropeptide FF receptor 2
ENSG00000214285	594857	H. sapiens	NPS	neuropeptide S
ENSG00000122585	4852	H. sapiens	NPY	neuropeptide Y
ENSG00000112333	7101	H. sapiens	NR2E1	nuclear receptor subfamily 2 group E member 1
ENSG00000154146	4900	H. sapiens	NRGN	neurogranin
ENSG00000118257	8828	H. sapiens	NRP2	neuropilin 2
ENSG00000103274	4682	H. sapiens	NUBP1	nucleotide binding protein 1
ENSG00000163002	129401	H. sapiens	NUP35	nucleoporin 35
ENSG00000153989	116150	H. sapiens	NUS1	NUS1 dehydrodolichyl diphosphate synthase subunit
ENSG00000130045	158046	H. sapiens	NXNL2	nucleoredoxin like 2

ENSG00000137634	54827	H. sapiens	NXPE4	neurexophilin and PC-esterase domain family member 4
ENSG00000174792	152816	H. sapiens	ODAPH	odontogenesis associated phosphoprotein
ENSG00000105953	4967	H. sapiens	OGDH	oxoglutarate dehydrogenase
ENSG00000180409	81487	H. sapiens	OR10AA1P	olfactory receptor family 10 subfamily AA member 1 pseudogene
ENSG00000251686	343409	H. sapiens	OR10J8P	olfactory receptor family 10 subfamily J member 8 pseudogene
ENSG00000198967	128368	H. sapiens	OR10Z1	olfactory receptor family 10 subfamily Z member 1
ENSG00000127780	8388	H. sapiens	OR1E2	olfactory receptor family 1 subfamily E member 2
ENSG00000244623	81392	H. sapiens	OR2AE1	olfactory receptor family 2 subfamily AE member 1
ENSG00000181903	219432	H. sapiens	OR4C6	olfactory receptor family 4 subfamily C member 6
ENSG00000167332	81285	H. sapiens	OR51E2	olfactory receptor family 51 subfamily E member 2
ENSG00000172324	219981	H. sapiens	OR5A2	olfactory receptor family 5 subfamily A member 2
ENSG00000181518	338662	H. sapiens	OR8D4	olfactory receptor family 8 subfamily D member 4
ENSG00000229314	5004	H. sapiens	ORM1	orosomucoid 1
ENSG00000130703	9885	H. sapiens	OSBPL2	oxysterol binding protein like 2
ENSG00000070882	26031	H. sapiens	OSBPL3	oxysterol binding protein like 3
ENSG00000256285	106480232	H. sapiens	OSBPL9P5	oxysterol binding protein like 9 pseudogene 5
ENSG00000182447	131149	H. sapiens	OTOL1	otolin 1
ENSG00000187950	341350	H. sapiens	OVCH1	ovochymase 1
ENSG00000248668	100874002	H. sapiens	OXCT1-AS1	OXCT1 antisense RNA 1
ENSG00000101104	80336	H. sapiens	PABPC1L	poly(A) binding protein cytoplasmic 1 like
ENSG00000124507	29993	H. sapiens	PACSIN1	protein kinase C and casein kinase substrate in neurons 1
ENSG00000145730	5066	H. sapiens	PAM	peptidylglycine alpha-amidating monooxygenase
ENSG00000073150	56666	H. sapiens	PANX2	pannexin 2
ENSG00000138801	9061	H. sapiens	PAPSS1	3'-phosphoadenosine 5'-phosphosulfate synthase 1
ENSG00000116117	117583	H. sapiens	PARD3B	par-3 family cell polarity regulator beta
ENSG00000173200	165631	H. sapiens	PARP15	poly(ADP-ribose) polymerase family member 15

ENSG00000265369	728606	H. sapiens	PCAT18	prostate cancer associated transcript 18
ENSG00000156453	5097	H. sapiens	PCDH1	protocadherin 1
ENSG00000081154	57092	H. sapiens	PCNP	PEST proteolytic signal containing nuclear protein
ENSG00000135749	80003	H. sapiens	PCNX2	pecanex 2
ENSG00000183036	5121	H. sapiens	PCP4	Purkinje cell protein 4
ENSG00000161217	5130	H. sapiens	PCYT1A	phosphate cytidylyltransferase 1, choline, alpha
ENSG00000229739	102724919	H. sapiens	PDC-AS1	PDC antisense RNA 1
ENSG00000105185	9141	H. sapiens	PDCD5	programmed cell death 5
ENSG00000170248	10015	H. sapiens	PDCD6IP	programmed cell death 6 interacting protein
ENSG00000065989	5141	H. sapiens	PDE4A	phosphodiesterase 4A
ENSG00000184588	5142	H. sapiens	PDE4B	phosphodiesterase 4B
ENSG00000227466	101927139	H. sapiens	PDE4B-AS1	PDE4B antisense RNA 1
ENSG00000110435	8050	H. sapiens	PDHX	pyruvate dehydrogenase complex component X
ENSG00000162493	10630	H. sapiens	PDPN	podoplanin
ENSG00000242265	23089	H. sapiens	PEG10	paternally expressed 10
ENSG00000215193	55670	H. sapiens	PEX26	peroxisomal biogenesis factor 26
ENSG00000170525	5209	H. sapiens	PFKFB3	6-phosphofructo-2-kinase/fructose-2,6-biphosphatase 3
ENSG00000197121	80055	H. sapiens	PGAP1	post-GPI attachment to proteins inositol deacylase 1
ENSG00000238194	100506384	H. sapiens	PHACTR3-AS1	PHACTR3 antisense RNA 1
ENSG00000119403	26147	H. sapiens	PHF19	PHD finger protein 19
ENSG00000006576	57157	H. sapiens	PHTF2	putative homeodomain transcription factor 2
ENSG00000228839	101929760	H. sapiens	PIK3IP1-DT	PIK3IP1 divergent transcript
ENSG00000254266	101927003	H. sapiens	PKIA-AS1	PKIA antisense RNA 1
ENSG00000143627	5313	H. sapiens	PKLR	pyruvate kinase L/R
ENSG00000057294	5318	H. sapiens	PKP2	plakophilin 2
ENSG00000117215	26279	H. sapiens	PLA2G2D	phospholipase A2 group IID
ENSG00000104368	5327	H. sapiens	PLAT	plasminogen activator, tissue type
ENSG00000101333	5332	H. sapiens	PLCB4	phospholipase C beta 4
ENSG00000115896	5334	H. sapiens	PLCL1	phospholipase C like 1 (inactive)
ENSG00000075651	5337	H. sapiens	PLD1	phospholipase D1
ENSG00000140464	5371	H. sapiens	PML	PML nuclear body scaffold
ENSG00000078319	5379	H. sapiens	PMS2P1	PMS1 homolog 2, mismatch repair system component pseudogene 1
ENSG00000070501	5423	H. sapiens	POLB	DNA polymerase beta
ENSG00000122008	51426	H. sapiens	POLK	DNA polymerase kappa

ENSG00000167390	651452	H. sapiens	POM121L3P	POM121 transmembrane nucleoporin like 3, pseudogene
ENSG00000279261	100130249	H. sapiens	PP2672	uncharacterized LOC100130249
ENSG00000138621	60490	H. sapiens	PPCDC	phosphopantothenoylcysteine decarboxylase
ENSG00000110841	8496	H. sapiens	PPFIBP1	PPFIA binding protein 1
ENSG00000271131	111082968	H. sapiens	PPIAP75	peptidylprolyl isomerase A pseudogene 75
ENSG00000250484	111082983	H. sapiens	PPIAP76	peptidylprolyl isomerase A pseudogene 76
ENSG00000224986	5512	H. sapiens	PPP1R8P1	protein phosphatase 1 regulatory subunit 8 pseudogene 1
ENSG00000137509	5547	H. sapiens	PRCP	prolylcarboxypeptidase
ENSG00000112238	59336	H. sapiens	PRDM13	PR/SET domain 13
ENSG00000253729	5591	H. sapiens	PRKDC	protein kinase, DNA-activated, catalytic subunit
ENSG00000138669	5593	H. sapiens	PRKG2	protein kinase cGMP-dependent 2
ENSG00000282961	101867536	H. sapiens	PRNCR1	prostate cancer associated non-coding RNA 1
ENSG00000180259	149830	H. sapiens	PRNT	prion locus lncRNA, testis expressed
ENSG00000264685	100422496	H. sapiens	PRPF19P1	PRPF19 pseudogene 1
ENSG00000226847	100861436	H. sapiens	PRPS1P1	phosphoribosyl pyrophosphate synthetase 1 pseudogene 1
ENSG00000184838	51334	H. sapiens	PRR16	proline rich 16
ENSG00000283526	642515	H. sapiens	PRRT1B	proline rich transmembrane protein 1B
ENSG00000146250	167681	H. sapiens	PRSS35	serine protease 35
ENSG00000106588	5683	H. sapiens	PSMA2	proteasome 20S subunit alpha 2
ENSG00000217385	442153	H. sapiens	PSMC1P11	proteasome 26S subunit, ATPase 1 pseudogene 11
ENSG00000105894	5764	H. sapiens	PTN	pleiotrophin
ENSG00000173482	5797	H. sapiens	PTPRM	protein tyrosine phosphatase receptor type M
ENSG00000163661	5806	H. sapiens	PTX3	pentraxin 3
ENSG00000241945	5822	H. sapiens	PWP2	PWP2 small subunit processome component
ENSG00000163564	149628	H. sapiens	PYHIN1	pyrin and HIN domain family member 1
ENSG00000041353	5874	H. sapiens	RAB27B	RAB27B, member RAS oncogene family
ENSG00000152932	115827	H. sapiens	RAB3C	RAB3C, member RAS oncogene family

ENSG00000188060	115273	H. sapiens	RAB42	RAB42, member RAS oncogene family
ENSG00000236101	100874390	H. sapiens	RAC1P7	Rac family small GTPase 1 pseudogene 7
ENSG00000236570	131185	H. sapiens	RAD23BP1	RAD23B pseudogene 1
ENSG00000136828	9649	H. sapiens	RALGPS1	Ral GEF with PH domain and SH3 binding motif 1
ENSG00000164188	202151	H. sapiens	RANBP3L	RAN binding protein 3 like
ENSG00000173166	65059	H. sapiens	RAPH1	Ras association (RalGDS/AF-6) and pleckstrin homology domains 1
ENSG00000131759	5914	H. sapiens	RARA	retinoic acid receptor alpha
ENSG00000101773	5932	H. sapiens	RBBP8	RB binding protein 8, endonuclease
ENSG00000230399	100420552	H. sapiens	RBBP8P1	RBBP8 pseudogene 1
ENSG00000168214	3516	H. sapiens	RBPJ	recombination signal binding protein for immunoglobulin kappa J region
ENSG00000263818	100131347	H. sapiens	RDM1P5	RDM1 pseudogene 5
ENSG00000214022	29803	H. sapiens	REPIN1	replication initiator 1
ENSG00000169891	9185	H. sapiens	REPS2	RALBP1 associated Eps domain containing 2
ENSG00000132005	5989	H. sapiens	RFX1	regulatory factor X1
ENSG00000111783	5992	H. sapiens	RFX4	regulatory factor X4
ENSG00000150681	64407	H. sapiens	RGS18	regulator of G protein signaling 18
ENSG00000129667	79651	H. sapiens	RHBDF2	rhomboid 5 homolog 2
ENSG00000263961	440712	H. sapiens	RHEX	regulator of hemoglobinization and erythroid cell expansion
ENSG00000164292	22836	H. sapiens	RHOBTB3	Rho related BTB domain containing 3
ENSG00000164327	253260	H. sapiens	RICTOR	RPTOR independent companion of MTOR complex 2
ENSG00000079841	22999	H. sapiens	RIMS1	regulating synaptic membrane exocytosis 1
ENSG00000200047	106479146	H. sapiens	RN7SKP115	RN7SK pseudogene 115
ENSG00000252358	106480875	H. sapiens	RN7SKP135	RN7SK pseudogene 135
ENSG00000222704	106479177	H. sapiens	RN7SKP182	RN7SK pseudogene 182
ENSG00000252978	106480889	H. sapiens	RN7SKP183	RN7SK pseudogene 183
ENSG00000252396	106481816	H. sapiens	RN7SKP195	RN7SK pseudogene 195
ENSG00000201782	106480903	H. sapiens	RN7SKP226	RN7SK pseudogene 226
ENSG00000222616	106480841	H. sapiens	RN7SKP27	RN7SK pseudogene 27
ENSG00000222889	106480457	H. sapiens	RN7SKP29	RN7SK pseudogene 29
ENSG00000253073	106480924	H. sapiens	RN7SKP295	RN7SK pseudogene 295
ENSG00000253020	106479113	H. sapiens	RN7SKP40	RN7SK pseudogene 40
ENSG00000252028	106479118	H. sapiens	RN7SKP52	RN7SK pseudogene 52

ENSG00000223269	106480849	H. sapiens	RN7SKP53	RN7SK pseudogene 53
ENSG00000243541	106480949	H. sapiens	RN7SL104P	RNA, 7SL, cytoplasmic 104, pseudogene
ENSG00000243546	106480950	H. sapiens	RN7SL108P	RNA, 7SL, cytoplasmic 108, pseudogene
ENSG00000240474	106480488	H. sapiens	RN7SL116P	RNA, 7SL, cytoplasmic 116, pseudogene
ENSG00000243398	106480959	H. sapiens	RN7SL141P	RNA, 7SL, cytoplasmic 141, pseudogene
ENSG00000239679	106480493	H. sapiens	RN7SL193P	RNA, 7SL, cytoplasmic 193, pseudogene
ENSG00000264916	106479306	H. sapiens	RN7SL230P	RNA, 7SL, cytoplasmic 230, pseudogene
ENSG00000241652	106479314	H. sapiens	RN7SL253P	RNA, 7SL, cytoplasmic 253, pseudogene
ENSG00000242121	106481833	H. sapiens	RN7SL267P	RNA, 7SL, cytoplasmic 267, pseudogene
ENSG00000243845	106479235	H. sapiens	RN7SL30P	RNA, 7SL, cytoplasmic 30, pseudogene
ENSG00000242989	106479345	H. sapiens	RN7SL332P	RNA, 7SL, cytoplasmic 332, pseudogene
ENSG00000242482	106480506	H. sapiens	RN7SL392P	RNA, 7SL, cytoplasmic 392, pseudogene
ENSG00000239396	106481036	H. sapiens	RN7SL414P	RNA, 7SL, cytoplasmic 414, pseudogene
ENSG00000241229	106480510	H. sapiens	RN7SL443P	RNA, 7SL, cytoplasmic 443, pseudogene
ENSG00000243871	106481055	H. sapiens	RN7SL487P	RNA, 7SL, cytoplasmic 487, pseudogene
ENSG00000242985	106479243	H. sapiens	RN7SL50P	RNA, 7SL, cytoplasmic 50, pseudogene
ENSG00000243075	106479412	H. sapiens	RN7SL519P	RNA, 7SL, cytoplasmic 519, pseudogene
ENSG00000264615	106481851	H. sapiens	RN7SL592P	RNA, 7SL, cytoplasmic 592, pseudogene
ENSG00000243420	106479490	H. sapiens	RN7SL734P	RNA, 7SL, cytoplasmic 734, pseudogene
ENSG00000277506	106479513	H. sapiens	RN7SL802P	RNA, 7SL, cytoplasmic 802, pseudogene
ENSG00000263595	106479522	H. sapiens	RN7SL823P	RNA, 7SL, cytoplasmic 823, pseudogene
ENSG00000243383	106479256	H. sapiens	RN7SL89P	RNA, 7SL, cytoplasmic 89, pseudogene
ENSG00000223318	100873381	H. sapiens	RNA5SP111	RNA, 5S ribosomal pseudogene 111
ENSG00000222838	100873404	H. sapiens	RNA5SP136	RNA, 5S ribosomal pseudogene 136
ENSG00000202502	100873417	H. sapiens	RNA5SP151	RNA, 5S ribosomal pseudogene 151
ENSG00000252002	100873420	H. sapiens	RNA5SP154	RNA, 5S ribosomal pseudogene 154

ENSG00000222230	100873424	H. sapiens	RNA5SP158	RNA, 5S ribosomal pseudogene 158
ENSG00000252342	100873429	H. sapiens	RNA5SP164	RNA, 5S ribosomal pseudogene 164
ENSG00000223003	100873446	H. sapiens	RNA5SP184	RNA, 5S ribosomal pseudogene 184
ENSG00000222285	100873455	H. sapiens	RNA5SP193	RNA, 5S ribosomal pseudogene 193
ENSG00000201523	100873466	H. sapiens	RNA5SP205	RNA, 5S ribosomal pseudogene 205
ENSG00000212333	100873473	H. sapiens	RNA5SP213	RNA, 5S ribosomal pseudogene 213
ENSG00000200058	100873478	H. sapiens	RNA5SP218	RNA, 5S ribosomal pseudogene 218
ENSG00000252041	100873486	H. sapiens	RNA5SP228	RNA, 5S ribosomal pseudogene 228
ENSG00000212308	100873278	H. sapiens	RNA5SP23	RNA, 5S ribosomal pseudogene 23
ENSG00000222346	100873495	H. sapiens	RNA5SP237	RNA, 5S ribosomal pseudogene 237
ENSG00000212628	100873499	H. sapiens	RNA5SP241	RNA, 5S ribosomal pseudogene 241
ENSG00000222546	100873507	H. sapiens	RNA5SP251	RNA, 5S ribosomal pseudogene 251
ENSG00000201763	100873520	H. sapiens	RNA5SP267	RNA, 5S ribosomal pseudogene 267
ENSG00000252428	100873538	H. sapiens	RNA5SP285	RNA, 5S ribosomal pseudogene 285
ENSG00000212454	100873539	H. sapiens	RNA5SP286	RNA, 5S ribosomal pseudogene 286
ENSG00000252496	100873365	H. sapiens	RNA5SP33	RNA, 5S ribosomal pseudogene 33
ENSG00000200687	106479007	H. sapiens	RNA5SP335	RNA, 5S ribosomal pseudogene 335
ENSG00000201059	100873599	H. sapiens	RNA5SP336	RNA, 5S ribosomal pseudogene 336
ENSG00000202290	100873369	H. sapiens	RNA5SP37	RNA, 5S ribosomal pseudogene 37
ENSG00000252260	100873705	H. sapiens	RNA5SP461	RNA, 5S ribosomal pseudogene 461
ENSG00000199806	100873733	H. sapiens	RNA5SP491	RNA, 5S ribosomal pseudogene 491
ENSG00000201285	111644143	H. sapiens	RNA5SP524	RNA, 5S ribosomal pseudogene 524
ENSG00000170153	57484	H. sapiens	RNF150	ring finger protein 150
ENSG00000165188	138065	H. sapiens	RNF183	ring finger protein 183
ENSG00000138942	91445	H. sapiens	RNF185	ring finger protein 185
ENSG00000271739	106480154	H. sapiens	RNU1-29P	RNA, U1 small nuclear 29, pseudogene
ENSG00000207056	106481604	H. sapiens	RNU1-8P	RNA, U1 small nuclear 8, pseudogene

ENSG00000201616	106480179	H. sapiens	RNU1-91P	RNA, U1 small nuclear 91, pseudogene
ENSG00000222357	106481640	H. sapiens	RNU2-20P	RNA, U2 small nuclear 20, pseudogene
ENSG00000251994	106481642	H. sapiens	RNU2-27P	RNA, U2 small nuclear 27, pseudogene
ENSG00000252066	106480220	H. sapiens	RNU2-53P	RNA, U2 small nuclear 53, pseudogene
ENSG00000202429	106479580	H. sapiens	RNU4-48P	RNA, U4 small nuclear 48, pseudogene
ENSG00000206936	106481188	H. sapiens	RNU4-52P	RNA, U4 small nuclear 52, pseudogene
ENSG00000223152	106479599	H. sapiens	RNU4-88P	RNA, U4 small nuclear 88, pseudogene
ENSG00000264229	100151683	H. sapiens	RNU4ATAC	RNA, U4atac small nuclear (U12-dependent splicing)
ENSG00000199377	26828	H. sapiens	RNU5F-1	RNA, U5F small nuclear 1
ENSG00000200637	100873866	H. sapiens	RNU5F-3P	RNA, U5F small nuclear 3, pseudogene
ENSG00000252414	106481208	H. sapiens	RNU6-100P	RNA, U6 small nuclear 100, pseudogene
ENSG00000238680	106481508	H. sapiens	RNU6-1037P	RNA, U6 small nuclear 1037, pseudogene
ENSG00000207267	106480636	H. sapiens	RNU6-1081P	RNA, U6 small nuclear 1081, pseudogene
ENSG00000252935	106481565	H. sapiens	RNU6-1220P	RNA, U6 small nuclear 1220, pseudogene
ENSG00000252994	106480104	H. sapiens	RNU6-1231P	RNA, U6 small nuclear 1231, pseudogene
ENSG00000206618	106480117	H. sapiens	RNU6-1264P	RNA, U6 small nuclear 1264, pseudogene
ENSG00000252151	106481579	H. sapiens	RNU6-1265P	RNA, U6 small nuclear 1265, pseudogene
ENSG00000206703	106481217	H. sapiens	RNU6-128P	RNA, U6 small nuclear 128, pseudogene
ENSG00000202206	106481229	H. sapiens	RNU6-169P	RNA, U6 small nuclear 169, pseudogene
ENSG00000206866	106481880	H. sapiens	RNU6-187P	RNA, U6 small nuclear 187, pseudogene
ENSG00000252104	106479651	H. sapiens	RNU6-191P	RNA, U6 small nuclear 191, pseudogene
ENSG00000252556	106479680	H. sapiens	RNU6-256P	RNA, U6 small nuclear 256, pseudogene
ENSG00000200963	106479695	H. sapiens	RNU6-289P	RNA, U6 small nuclear 289, pseudogene
ENSG00000252126	106480569	H. sapiens	RNU6-313P	RNA, U6 small nuclear 313, pseudogene
ENSG00000199536	106479707	H. sapiens	RNU6-315P	RNA, U6 small nuclear 315, pseudogene
ENSG00000202239	106481303	H. sapiens	RNU6-396P	RNA, U6 small nuclear 396, pseudogene

ENSG00000206762	106481310	H. sapiens	RNU6-418P	RNA, U6 small nuclear 418, pseudogene
ENSG00000207312	106479755	H. sapiens	RNU6-429P	RNA, U6 small nuclear 429, pseudogene
ENSG00000200086	106481315	H. sapiens	RNU6-433P	RNA, U6 small nuclear 433, pseudogene
ENSG00000202431	106479760	H. sapiens	RNU6-438P	RNA, U6 small nuclear 438, pseudogene
ENSG00000252861	106479764	H. sapiens	RNU6-448P	RNA, U6 small nuclear 448, pseudogene
ENSG00000207200	100873759	H. sapiens	RNU6-45P	RNA, U6 small nuclear 45, pseudogene
ENSG00000206970	106481329	H. sapiens	RNU6-474P	RNA, U6 small nuclear 474, pseudogene
ENSG00000251727	106479782	H. sapiens	RNU6-488P	RNA, U6 small nuclear 488, pseudogene
ENSG00000206932	106478914	H. sapiens	RNU6-4P	RNA, U6 small nuclear 4, pseudogene
ENSG00000207090	106479795	H. sapiens	RNU6-517P	RNA, U6 small nuclear 517, pseudogene
ENSG00000200146	106481353	H. sapiens	RNU6-544P	RNA, U6 small nuclear 544, pseudogene
ENSG00000252688	106481364	H. sapiens	RNU6-579P	RNA, U6 small nuclear 579, pseudogene
ENSG00000206999	106479841	H. sapiens	RNU6-622P	RNA, U6 small nuclear 622, pseudogene
ENSG00000200563	106479850	H. sapiens	RNU6-640P	RNA, U6 small nuclear 640, pseudogene
ENSG00000207225	106479871	H. sapiens	RNU6-692P	RNA, U6 small nuclear 692, pseudogene
ENSG00000252890	106479875	H. sapiens	RNU6-699P	RNA, U6 small nuclear 699, pseudogene
ENSG00000202074	106481405	H. sapiens	RNU6-715P	RNA, U6 small nuclear 715, pseudogene
ENSG00000212468	106481418	H. sapiens	RNU6-754P	RNA, U6 small nuclear 754, pseudogene
ENSG00000207414	106481775	H. sapiens	RNU6-768P	RNA, U6 small nuclear 768, pseudogene
ENSG00000206807	106480611	H. sapiens	RNU6-815P	RNA, U6 small nuclear 815, pseudogene
ENSG00000206996	106481447	H. sapiens	RNU6-842P	RNA, U6 small nuclear 842, pseudogene
ENSG00000207183	106480614	H. sapiens	RNU6-843P	RNA, U6 small nuclear 843, pseudogene
ENSG00000199448	106479940	H. sapiens	RNU6-845P	RNA, U6 small nuclear 845, pseudogene
ENSG00000251757	106479941	H. sapiens	RNU6-848P	RNA, U6 small nuclear 848, pseudogene
ENSG00000271932	106481203	H. sapiens	RNU6-85P	RNA, U6 small nuclear 85, pseudogene

ENSG00000206848	106480618	H. sapiens	RNU6-890P	RNA, U6 small nuclear 890, pseudogene
ENSG00000207323	106479965	H. sapiens	RNU6-901P	RNA, U6 small nuclear 901, pseudogene
ENSG00000207163	106479967	H. sapiens	RNU6-905P	RNA, U6 small nuclear 905, pseudogene
ENSG00000199872	106481477	H. sapiens	RNU6-942P	RNA, U6 small nuclear 942, pseudogene
ENSG00000221507	106479560	H. sapiens	RNU6ATAC40P	RNA, U6atac small nuclear 40, pseudogene
ENSG00000252222	100147761	H. sapiens	RNU7-13P	RNA, U7 small nuclear 13 pseudogene
ENSG00000238452	106479079	H. sapiens	RNU7-144P	RNA, U7 small nuclear 144 pseudogene
ENSG00000252003	106481802	H. sapiens	RNU7-154P	RNA, U7 small nuclear 154 pseudogene
ENSG00000238950	106479090	H. sapiens	RNU7-173P	RNA, U7 small nuclear 173 pseudogene
ENSG00000238721	106480834	H. sapiens	RNU7-194P	RNA, U7 small nuclear 194 pseudogene
ENSG00000252514	100151649	H. sapiens	RNU7-53P	RNA, U7 small nuclear 53 pseudogene
ENSG00000243844	646909	H. sapiens	RPL17P33	ribosomal protein L17 pseudogene 33
ENSG00000213717	100271285	H. sapiens	RPL18AP10	ribosomal protein L18a pseudogene 10
ENSG00000223916	731642	H. sapiens	RPL18AP9	ribosomal protein L18a pseudogene
ENSG00000213661	100129374	H. sapiens	RPL18P3	ribosomal protein L18 pseudogene 3
ENSG00000234985	392979	H. sapiens	RPL18P4	ribosomal protein L18 pseudogene 4
ENSG00000224469	100270944	H. sapiens	RPL18P5	ribosomal protein L18 pseudogene 5
ENSG00000224019	100271161	H. sapiens	RPL21P32	ribosomal protein L21 pseudogene 32
ENSG00000224593	100271181	H. sapiens	RPL21P72	ribosomal protein L21 pseudogene 72
ENSG00000243181	100133273	H. sapiens	RPL21P80	ribosomal protein L21 pseudogene 80
ENSG00000243071	653079	H. sapiens	RPL21P98	ribosomal protein L21 pseudogene 98
ENSG00000235990	645962	H. sapiens	RPL23AP20	ribosomal protein L23a pseudogene 20
ENSG00000244413	645809	H. sapiens	RPL23AP56	ribosomal protein L23a pseudogene 56
ENSG00000243964	440027	H. sapiens	RPL23AP65	ribosomal protein L23a pseudogene 65
ENSG00000232751	730070	H. sapiens	RPL26P11	ribosomal protein L26 pseudogene 11

ENSG00000225713	100270975	H. sapiens	RPL30P1	ribosomal protein L30 pseudogene 1
ENSG00000230837	140753	H. sapiens	RPL31P2	ribosomal protein L31 pseudogene 2
ENSG00000227433	100270989	H. sapiens	RPL31P22	ribosomal protein L31 pseudogene 22
ENSG00000241853	100271194	H. sapiens	RPL31P24	ribosomal protein L31 pseudogene 24
ENSG00000239983	727792	H. sapiens	RPL31P31	ribosomal protein L31 pseudogene 31
ENSG00000243164	645654	H. sapiens	RPL31P48	ribosomal protein L31 pseudogene 48
ENSG00000231333	100270998	H. sapiens	RPL34P6	ribosomal protein L34 pseudogene 6
ENSG00000163923	116832	H. sapiens	RPL39L	ribosomal protein L39 like
ENSG00000256338	22970	H. sapiens	RPL41P2	ribosomal protein L41 pseudogene 2
ENSG00000214671	100270907	H. sapiens	RPL6P12	ribosomal protein L6 pseudogene 12
ENSG00000230383	646483	H. sapiens	RPL6P19	ribosomal protein L6 pseudogene 19
ENSG00000234358	100271215	H. sapiens	RPL7AP42	ribosomal protein L7a pseudogene 42
ENSG00000242248	100271537	H. sapiens	RPL7AP57	ribosomal protein L7a pseudogene 57
ENSG00000234253	130728	H. sapiens	RPL7P13	ribosomal protein L7 pseudogene 13
ENSG00000243806	100270852	H. sapiens	RPL7P18	ribosomal protein L7 pseudogene 18
ENSG00000228599	646912	H. sapiens	RPL7P52	ribosomal protein L7 pseudogene 52
ENSG00000238069	100271047	H. sapiens	RPLP0P7	ribosomal protein lateral stalk subunit P0 pseudogene 7
ENSG00000227560	100271607	H. sapiens	RPS15AP30	ribosomal protein S15a pseudogene 30
ENSG00000244621	100271072	H. sapiens	RPS17P11	ribosomal protein S17 pseudogene 11
ENSG00000223918	100271236	H. sapiens	RPS21P5	ribosomal protein S21 pseudogene 5
ENSG00000233946	100271123	H. sapiens	RPS29P4	ribosomal protein S29 pseudogene 4
ENSG00000244249	100270859	H. sapiens	RPS2P19	ribosomal protein S2 pseudogene 19
ENSG00000243894	727951	H. sapiens	RPS3AP16	RPS3A pseudogene 16
ENSG00000217684	100271253	H. sapiens	RPS3AP24	RPS3A pseudogene 24
ENSG00000243101	645957	H. sapiens	RPS3P7	ribosomal protein S3 pseudogene 7
ENSG00000230871	100130313	H. sapiens	RPS6P23	ribosomal protein S6 pseudogene 23

ENSG00000244669	100130347	H. sapiens	RPS6P5	ribosomal protein S6 pseudogene 5
ENSG00000213326	644315	H. sapiens	RPS7P11	ribosomal protein S7 pseudogene 11
ENSG00000240167	100270870	H. sapiens	RPS7P7	ribosomal protein S7 pseudogene 7
ENSG00000240376	728553	H. sapiens	RPS8P8	ribosomal protein S8 pseudogene 8
ENSG00000241597	100133054	H. sapiens	RPS9P3	ribosomal protein S9 pseudogene 3
ENSG00000267524	390857	H. sapiens	RPSAP57	ribosomal protein SA pseudogene 57
ENSG00000250327	105377595	H. sapiens	RPSAP70	ribosomal protein SA pseudogene 70
ENSG00000189306	27341	H. sapiens	RRP7A	ribosomal RNA processing 7 homolog A
ENSG00000182010	219790	H. sapiens	RTKN2	rhotekin 2
ENSG00000176225	25914	H. sapiens	RTTN	rotatin
ENSG00000163191	6282	H. sapiens	S100A11	S100 calcium binding protein A11
ENSG00000280515	104548973	H. sapiens	SALRNA2	senescence associated long non-coding RNA 2
ENSG00000079689	10590	H. sapiens	SCGN	secretagogin, EF-hand calcium binding protein
ENSG00000236107	101929680	H. sapiens	SCN1A-AS1	SCN1A and SCN9A antisense RNA 1
ENSG00000168447	6338	H. sapiens	SCNN1B	sodium channel epithelial 1 subunit beta
ENSG00000236272	105373051	H. sapiens	SCUBE1-AS2	SCUBE1 antisense RNA 2
ENSG00000245928	101928809	H. sapiens	SDAD1-AS1	SDAD1 antisense RNA 1
ENSG00000139410	113675	H. sapiens	SDSL	serine dehydratase like
ENSG00000138674	22872	H. sapiens	SEC31A	SEC31 homolog A, COPII coat complex component
ENSG00000254703	100507392	H. sapiens	SENCR	smooth muscle and endothelial cell enriched migration/differentiation-associated lncRNA
ENSG00000263718	105371907	H. sapiens	SEPTIN9-DT	SEPTIN9 divergent transcript
ENSG00000106366	5054	H. sapiens	SERPINE1	serpin family E member 1
ENSG00000149212	143686	H. sapiens	SESN3	sestrin 3
ENSG00000145423	6423	H. sapiens	SFRP2	secreted frizzled related protein 2
ENSG00000163069	6443	H. sapiens	SGCB	sarcoglycan beta
ENSG00000170624	6444	H. sapiens	SGCD	sarcoglycan delta
ENSG00000147010	30011	H. sapiens	SH3KBP1	SH3 domain containing kinase binding protein 1
ENSG00000162105	22941	H. sapiens	SHANK2	SH3 and multiple ankyrin repeat domains 2
ENSG00000089012	55423	H. sapiens	SIRPG	signal regulatory protein gamma

ENSG00000162241	283130	H. sapiens	SLC25A45	solute carrier family 25 member 45
ENSG00000224281	100303728	H. sapiens	SLC25A5-AS1	SLC25A5 antisense RNA 1
ENSG00000116704	23169	H. sapiens	SLC35D1	solute carrier family 35 member D1
ENSG00000134955	219855	H. sapiens	SLC37A2	solute carrier family 37 member 2
ENSG00000129353	57153	H. sapiens	SLC44A2	solute carrier family 44 member 2
ENSG00000013293	57709	H. sapiens	SLC7A14	solute carrier family 7 member 14
ENSG00000181804	285195	H. sapiens	SLC9A9	solute carrier family 9 member A9
ENSG00000164037	150159	H. sapiens	SLC9B1	solute carrier family 9 member B1
ENSG00000234855	100505540	H. sapiens	SLIT1-AS1	SLIT1 antisense RNA 1
ENSG00000095932	284422	H. sapiens	SMIM24	small integral membrane protein 24
ENSG00000230758	101290500	H. sapiens	SNAP23P1	synaptosome associated protein 23 pseudogene 1
ENSG00000184602	8303	H. sapiens	SNN	stannin
ENSG00000212588	677810	H. sapiens	SNORA26	small nucleolar RNA, H/ACA box 26
ENSG00000206780	109616994	H. sapiens	SNORA75B	small nucleolar RNA, H/ACA box 75B
ENSG00000207093	100033420	H. sapiens	SNORD116-8	small nucleolar RNA, C/D box 116-8
ENSG00000172845	6670	H. sapiens	SP3	Sp3 transcription factor
ENSG00000153498	122258	H. sapiens	SPACA7	sperm acrosome associated 7
ENSG00000118363	9789	H. sapiens	SPCS2	signal peptidase complex subunit 2
ENSG00000072195	10290	H. sapiens	SPEG	striated muscle enriched protein kinase
ENSG00000133710	11005	H. sapiens	SPINK5	serine peptidase inhibitor Kazal type 5
ENSG00000152377	6695	H. sapiens	SPOCK1	SPARC (osteonectin), cwcv and kazal like domains proteoglycan 1
ENSG00000107742	9806	H. sapiens	SPOCK2	SPARC (osteonectin), cwcv and kazal like domains proteoglycan 2
ENSG00000136158	10253	H. sapiens	SPRY2	sprouty RTK signaling antagonist 2
ENSG00000253431	100419416	H. sapiens	SRPK2P	SRSF protein kinase 2 pseudogene
ENSG00000124783	6745	H. sapiens	SSR1	signal sequence receptor subunit 1
ENSG00000115525	8869	H. sapiens	ST3GAL5	ST3 beta-galactoside alpha-2,3-sialyltransferase 5

ENSG00000113532	7903	H. sapiens	ST8SIA4	ST8 alpha-N-acetyl-neuraminide alpha-2,8-sialyltransferase 4
ENSG00000144681	6769	H. sapiens	STAC	SH3 and cysteine rich domain
ENSG00000198648	27347	H. sapiens	STK39	serine/threonine kinase 39
ENSG00000196182	83931	H. sapiens	STK40	serine/threonine kinase 40
ENSG00000165209	55342	H. sapiens	STRBP	spermatid perinuclear RNA binding protein
ENSG00000113387	10923	H. sapiens	SUB1	SUB1 regulator of transcription
ENSG00000137573	23213	H. sapiens	SULF1	sulfatase 1
ENSG00000253579	106480679	H. sapiens	SUMO2P16	SUMO2 pseudogene 16
ENSG00000164828	23353	H. sapiens	SUN1	Sad1 and UNC84 domain containing 1
ENSG00000132872	6860	H. sapiens	SYT4	synaptotagmin 4
ENSG00000187325	51616	H. sapiens	TAF9B	TATA-box binding protein associated factor 9b
ENSG00000136560	10010	H. sapiens	TANK	TRAF family member associated NFKB activator
ENSG00000204267	6891	H. sapiens	TAP2	transporter 2, ATP binding cassette subfamily B member
ENSG00000121075	9496	H. sapiens	TBX4	T-box transcription factor 4
ENSG00000176896	170082	H. sapiens	TCEANC	transcription elongation factor A N-terminal and central domain containing
ENSG00000070814	6949	H. sapiens	TCOF1	treacle ribosome biogenesis factor 1
ENSG00000256849	400013	H. sapiens	TCP1P3	t-complex 1 pseudogene 3
ENSG00000218819	100129278	H. sapiens	TDRD15	tudor domain containing 15
ENSG00000180190	157695	H. sapiens	TDRP	testis development related protein
ENSG00000188681	100132288	H. sapiens	TEKT4P2	tektin 4 pseudogene 2
ENSG00000251336	105377573	H. sapiens	TEMN3-AS1	TENM3 antisense transcript 1
ENSG00000133863	56154	H. sapiens	TEX15	testis expressed 15, meiosis and synapsis associated
ENSG00000281453	103611157	H. sapiens	TGFB2-OT1	TGFB2 overlapping transcript 1
ENSG00000163513	7048	H. sapiens	TGFBR2	transforming growth factor beta receptor 2
ENSG00000131931	55145	H. sapiens	THAP1	THAP domain containing 1
ENSG00000144115	55258	H. sapiens	THNSL2	threonine synthase like 2
ENSG00000206573	440944	H. sapiens	THUMPD3-AS1	THUMPD3 antisense RNA 1
ENSG00000111602	8914	H. sapiens	TIMELESS	timeless circadian regulator
ENSG00000100234	7078	H. sapiens	TIMP3	TIMP metallopeptidase inhibitor 3

ENSG00000057704	57458	H. sapiens	TMCC3	transmembrane and coiled-coil domain family 3
ENSG00000143183	54499	H. sapiens	TMCO1	transmembrane and coiled-coil domains 1
ENSG00000166069	145942	H. sapiens	TMCO5A	transmembrane and coiled-coil domains 5A
ENSG00000166292	55273	H. sapiens	TMEM100	transmembrane protein 100
ENSG00000132406	85013	H. sapiens	TMEM128	transmembrane protein 128
ENSG00000170006	201799	H. sapiens	TMEM154	transmembrane protein 154
ENSG00000121895	80008	H. sapiens	TMEM156	transmembrane protein 156
ENSG00000134490	85019	H. sapiens	TMEM241	transmembrane protein 241
ENSG00000171729	55092	H. sapiens	TMEM51	transmembrane protein 51
ENSG00000133687	83857	H. sapiens	TMTC1	transmembrane O-mannosyltransferase targeting cadherins 1
ENSG00000179104	160335	H. sapiens	TMTC2	transmembrane O-mannosyltransferase targeting cadherins 2
ENSG00000118503	7128	H. sapiens	TNFAIP3	TNF alpha induced protein 3
ENSG00000120949	943	H. sapiens	TNFRSF8	TNF receptor superfamily member 8
ENSG00000168884	79155	H. sapiens	TNIP2	TNFAIP3 interacting protein 2
ENSG00000136205	64759	H. sapiens	TNS3	tensin 3
ENSG00000241874	100287573	H. sapiens	TOMM22P6	TOMM22 pseudogene 6
ENSG00000175768	401505	H. sapiens	TOMM5	translocase of outer mitochondrial membrane 5
ENSG00000175274	9537	H. sapiens	TP53I11	tumor protein p53 inducible protein 11
ENSG00000146242	7162	H. sapiens	TPBG	trophoblast glycoprotein
ENSG00000188001	285386	H. sapiens	TPRG1	tumor protein p63 regulated 1
ENSG00000230115	100874027	H. sapiens	TPRG1-AS2	TPRG1 antisense RNA 2
ENSG00000127191	7186	H. sapiens	TRAF2	TNF receptor associated factor 2
ENSG00000182606	22906	H. sapiens	TRAK1	trafficking kinesin protein 1
ENSG00000276597	28580	H. sapiens	TRBV11-3	T cell receptor beta variable 11-3
ENSG00000237254	28557	H. sapiens	TRBV30	T cell receptor beta variable 30
ENSG00000185880	140691	H. sapiens	TRIM69	tripartite motif containing 69
ENSG00000104321	8989	H. sapiens	TRPA1	transient receptor potential cation channel subfamily A member 1
ENSG00000167723	162514	H. sapiens	TRPV3	transient receptor potential cation channel subfamily V member 3

ENSG00000068724	57217	H. sapiens	TTC7A	tetratricopeptide repeat domain 7A
ENSG00000214391	399942	H. sapiens	TUBAP2	tubulin alpha pseudogene 2
ENSG00000220418	100288513	H. sapiens	TUBB3P1	tubulin beta 3 class III pseudogene 1
ENSG00000126216	10426	H. sapiens	TUBGCP3	tubulin gamma complex associated protein 3
ENSG00000130338	56995	H. sapiens	TULP4	TUB like protein 4
ENSG00000074966	7294	H. sapiens	TXK	TXK tyrosine kinase
ENSG00000077498	7299	H. sapiens	TYR	tyrosinase
ENSG00000235995	100287279	H. sapiens	UBE2V1P6	UBE2V1 pseudogene 6
ENSG00000151116	55293	H. sapiens	UEVLD	UEV and lactate/malate dehyrogenase domains
ENSG00000107731	219699	H. sapiens	UNC5B	unc-5 netrin receptor B
ENSG00000162607	7398	H. sapiens	USP1	ubiquitin specific peptidase 1
ENSG00000120800	27340	H. sapiens	UTP20	UTP20 small subunit processome component
ENSG00000147679	84294	H. sapiens	UTP23	UTP23 small subunit processome component
ENSG00000230489	100873946	H. sapiens	VAV3-AS1	VAV3 antisense RNA 1
ENSG00000112715	7422	H. sapiens	VEGFA	vascular endothelial growth factor A
ENSG00000150630	7424	H. sapiens	VEGFC	vascular endothelial growth factor C
ENSG00000026025	7431	H. sapiens	VIM	vimentin
ENSG00000154978	81552	H. sapiens	VOPP1	VOPP1 WW domain binding protein
ENSG00000048707	55187	H. sapiens	VPS13D	vacuolar protein sorting 13 homolog D
ENSG00000163032	7447	H. sapiens	VSNL1	visinin like 1
ENSG00000214376	387804	H. sapiens	VSTM5	V-set and transmembrane domain containing 5
ENSG00000199990	56664	H. sapiens	VTRNA1-1	vault RNA 1-1
ENSG00000204396	80737	H. sapiens	VWA7	von Willebrand factor A domain containing 7
ENSG00000110799	7450	H. sapiens	VWF	von Willebrand factor
ENSG00000226210	100288778	H. sapiens	WASH8P	WAS protein family homolog 8, pseudogene
ENSG00000164961	9897	H. sapiens	WASHC5	WASH complex subunit 5
ENSG00000091157	23335	H. sapiens	WDR7	WD repeat domain 7
ENSG00000166483	7465	H. sapiens	WEE1	WEE1 G2 checkpoint kinase
ENSG00000002745	51384	H. sapiens	WNT16	Wnt family member 16
ENSG00000158156	55113	H. sapiens	XKR8	XK related 8
ENSG00000173950	152002	H. sapiens	XXYLT1	xyloside xylosyltransferase 1
ENSG00000213188	100131012	H. sapiens	YBX1P4	Y-box binding protein 1 pseudogene 4
ENSG00000236564	100130391	H. sapiens	YWHAQP5	YWHAQ pseudogene 5
ENSG00000109906	7704	H. sapiens	ZBTB16	zinc finger and BTB domain containing 16

ENSG00000156599	25921	H. sapiens	ZDHHC5	zinc finger DHHC-type palmitoyltransferase 5
ENSG00000128016	7538	H. sapiens	ZFP36	ZFP36 ring finger protein
ENSG00000020256	55734	H. sapiens	ZFP64	ZFP64 zinc finger protein
ENSG00000198315	7745	H. sapiens	ZKSCAN8	zinc finger with KRAB and SCAN domains 8
ENSG00000166261	7753	H. sapiens	ZNF202	zinc finger protein 202
ENSG00000010244	7756	H. sapiens	ZNF207	zinc finger protein 207
ENSG00000162664	284695	H. sapiens	ZNF326	zinc finger protein 326
ENSG00000269235	101669766	H. sapiens	ZNF350-AS1	ZNF350 antisense RNA 1
ENSG00000219665	101928464	H. sapiens	ZNF433-AS1	ZNF433 and ZNF878 antisense RNA 1
ENSG00000267871	105372476	H. sapiens	ZNF460-AS1	ZNF460 antisense RNA 1
ENSG00000144026	84874	H. sapiens	ZNF514	zinc finger protein 514
ENSG00000172000	80032	H. sapiens	ZNF556	zinc finger protein 556
ENSG00000130544	79230	H. sapiens	ZNF557	zinc finger protein 557
ENSG00000146757	168374	H. sapiens	ZNF92	zinc finger protein 92
ENSG00000170044	131368	H. sapiens	ZPLD1	zona pellucida like domain containing 1

GroupID	Category	Term	Description	LogP	Log (q-value)	InTerm_InList
1_Summary	GO Biological Processes	GO:0001525	angiogenesis	-6.64804	-2.302	39/611
1_Member	GO Biological Processes	GO:0001525	angiogenesis	-6.64804	-2.302	39/611
1_Member	GO Biological Processes	GO:0048514	blood vessel morphogenesis	-5.87672	-2.052	41/707
1_Member	GO Biological Processes	GO:0001568	blood vessel development	-5.37222	-2.052	43/794
1_Member	GO Biological Processes	GO:1901342	regulation of vasculature development	-4.81377	-1.672	28/446
1_Member	GO Biological Processes	GO:0045765	regulation of angiogenesis	-4.26255	-1.379	25/404
1_Member	GO Biological Processes	GO:0071363	cellular response to growth factor stimulus	-2.69214	-0.692	32/719
1_Member	GO Biological Processes	GO:0070848	response to growth factor	-2.6891	-0.692	33/749
1_Member	GO Biological Processes	GO:0001935	endothelial cell proliferation	-2.54881	-0.647	13/207
1_Member	GO Biological Processes	GO:1901343	negative regulation of vasculature development	-2.54881	-0.647	13/207
1_Member	GO Biological Processes	GO:1904018	positive regulation of vasculature development	-2.49395	-0.616	14/235
1_Member	GO Biological Processes	GO:0016525	negative regulation of	-2.37758	-0.547	12/192
1_Member	GO Biological Processes	GO:2000181	negative regulation of blood vessel morphogenesis	-2.34187	-0.523	12/194
1_Member	GO Biological Processes	GO:0045766	positive regulation of	-2.10745	-0.416	12/208
1_Member	GO Biological Processes	GO:0050673	epithelial cell proliferation	-2.02103	-0.381	21/465
2_Summary	GO Biological Processes	GO:0003013	circulatory system process	-5.83748	-2.052	35/559
2_Member	GO Biological Processes	GO:0003013	circulatory system process	-5.83748	-2.052	35/559
2_Member	GO Biological Processes	GO:0008015	blood circulation	-5.60138	-2.052	34/548
2_Member	GO Biological Processes	GO:0006939	smooth muscle contraction	-3.29275	-0.968	10/109
2_Member	GO Biological Processes	GO:0044057	regulation of system process	-2.74264	-0.725	29/626
2_Member	GO Biological Processes	GO:0003012	muscle system process	-2.69559	-0.692	23/460
2_Member	GO Biological Processes	GO:0006936	muscle contraction	-2.59022	-0.657	19/359
2_Member	GO Biological Processes	GO:0090257	regulation of muscle system	-2.56413	-0.648	15/256
2_Member	GO Biological Processes	GO:0006937	regulation of muscle contraction	-2.27834	-0.482	11/173
2_Member	GO Biological Processes	GO:0003018	vascular process in circulatory system	-2.24154	-0.465	11/175
2_Member	GO Biological Processes	GO:0006940	regulation of smooth muscle contraction	-2.19174	-0.457	6/65
3_Summary	GO Biological Processes	GO:1901385	regulation of voltage-gated calcium channel activity	-5.5351	-2.052	8/36
3_Member	GO Biological Processes	GO:1901385	regulation of voltage-gated calcium channel activity	-5.5351	-2.052	8/36
3_Member	GO Biological Processes	GO:0043269	regulation of ion transport	-3.08662	-0.877	33/708
3_Member	GO Biological Processes	GO:0034765	regulation of ion transmembrane transport	-3.01398	-0.850	25/489
3_Member	GO Biological Processes	GO:0010959	regulation of metal ion transport	-2.8138	-0.753	21/396
3_Member	GO Biological Processes	GO:1901019	regulation of calcium ion transmembrane transporter [illegible]	-2.68441	-0.692	8/89
3_Member	GO Biological Processes	GO:0034762	regulation of transmembrane transport	-2.65264	-0.690	27/577
3_Member	GO Biological Processes	GO:2001259	positive regulation of cation channel activity	-2.63943	-0.686	7/71
3_Member	GO Biological Processes	GO:2001257	regulation of cation channel activity	-2.56524	-0.648	12/182
3_Member	GO Biological Processes	GO:0070588	calcium ion transmembrane	-2.55987	-0.648	17/308
3_Member	GO Biological Processes	GO:0098662	inorganic cation transmembrane transport	-2.52884	-0.634	33/767
3_Member	GO Biological Processes	GO:0043270	positive regulation of ion	-2.52851	-0.634	16/284
3_Member	GO Biological Processes	GO:0006816	calcium ion transport	-2.43922	-0.574	21/426
3_Member	GO Biological Processes	GO:1901387	positive regulation of voltage-gated calcium channel activity	-2.40004	-0.552	3/13
3_Member	GO Biological Processes	GO:1904062	regulation of cation transmembrane transport	-2.36691	-0.540	18/349
3_Member	GO Biological Processes	GO:1903169	regulation of calcium ion transmembrane transport	-2.23891	-0.465	10/151
3_Member	GO Biological Processes	GO:0032414	positive regulation of ion transmembrane transporter [illegible]	-2.21787	-0.461	8/106

3_Member	GO Biological Processes	GO:0070838	divalent metal ion transport	-2.21391	-0.461	22/475
3_Member	GO Biological Processes	GO:0051924	regulation of calcium ion	-2.21364	-0.461	14/253
3_Member	GO Biological Processes	GO:0072511	divalent inorganic cation	-2.15278	-0.431	22/481
3_Member	GO Biological Processes	GO:0034764	positive regulation of transmembrane transport	-2.07605	-0.402	12/210
3_Member	GO Biological Processes	GO:0034767	positive regulation of ion transmembrane transport	-2.04992	-0.396	10/161
3_Member	GO Biological Processes	GO:0032412	regulation of ion transmembrane transporter activity	-2.04478	-0.396	14/265
3_Member	GO Biological Processes	GO:0032411	positive regulation of transporter activity	-2.03339	-0.390	8/114
4_Summary	GO Biological Processes	GO:0019932	second-messenger-mediated	-5.51467	-2.052	30/456
4_Member	GO Biological Processes	GO:0019932	second-messenger-mediated	-5.51467	-2.052	30/456
4_Member	GO Biological Processes	GO:0019933	cAMP-mediated signaling	-3.6795	-1.146	15/199
4_Member	GO Biological Processes	GO:0019935	cyclic-nucleotide-mediated	-3.61792	-1.129	16/224
4_Member	GO Biological Processes	GO:0007189	adenylate cyclase-activating G protein-coupled receptor signaling pathway	-3.3672	-0.999	12/147
4_Member	GO Biological Processes	GO:0045744	negative regulation of G protein-coupled receptor signaling	-2.55313	-0.647	6/55
4_Member	GO Biological Processes	GO:0071880	adenylate cyclase-activating adrenergic receptor signaling pathway	-2.26669	-0.475	4/28
4_Member	GO Biological Processes	GO:0007187	G protein-coupled receptor signaling pathway, coupled to cyclic nucleotide second	-2.072	-0.400	14/263
4_Member	GO Biological Processes	GO:0071875	adrenergic receptor signaling pathway	-2.05715	-0.398	4/32
5_Summary	GO Biological Processes	GO:0035924	cellular response to vascular endothelial growth factor	-5.4854	-2.052	11/75
5_Member	GO Biological Processes	GO:0035924	cellular response to vascular endothelial growth factor	-5.4854	-2.052	11/75
5_Member	GO Biological Processes	GO:0038084	vascular endothelial growth factor signaling pathway	-3.50803	-1.070	7/51
5_Member	GO Biological Processes	GO:0001945	lymph vessel development	-3.33425	-0.978	5/26
5_Member	GO Biological Processes	GO:0036303	lymph vessel morphogenesis	-2.82213	-0.753	4/20
5_Member	Canonical Pathways	M137	PID VEGF VEGFR PATHWAY	-2.75225	-0.727	3/10
5_Member	GO Biological Processes	GO:0001946	lymphangiogenesis	-2.21499	-0.461	3/15
6_Summary	GO Biological Processes	GO:0043065	positive regulation of apoptotic process	-5.39929	-2.052	39/687
6_Member	GO Biological Processes	GO:0043065	positive regulation of apoptotic process	-5.39929	-2.052	39/687
6_Member	GO Biological Processes	GO:0097190	apoptotic signaling pathway	-5.36961	-2.052	36/612
6_Member	GO Biological Processes	GO:0043068	positive regulation of programmed cell death	-5.267	-2.000	39/696
6_Member	GO Biological Processes	GO:0010942	positive regulation of cell death	-4.5461	-1.550	39/749
6_Member	GO Biological Processes	GO:2001233	regulation of apoptotic signaling pathway	-4.53412	-1.550	26/413
6_Member	GO Biological Processes	GO:2001235	positive regulation of apoptotic signaling pathway	-4.08337	-1.341	15/183
6_Member	GO Biological Processes	GO:2001236	regulation of extrinsic apoptotic signaling pathway	-3.52733	-1.084	13/162
6_Member	GO Biological Processes	GO:0097191	extrinsic apoptotic signaling pathway	-3.01939	-0.852	15/230
6_Member	GO Biological Processes	GO:2001238	positive regulation of extrinsic apoptotic signaling pathway	-2.67852	-0.692	6/52
6_Member	GO Biological Processes	GO:2001237	negative regulation of extrinsic apoptotic signaling pathway	-2.19373	-0.457	8/107
6_Member	GO Biological Processes	GO:2001234	negative regulation of apoptotic signaling pathway	-2.12178	-0.422	13/233
7_Summary	GO Biological Processes	GO:0046635	positive regulation of alpha-beta T cell activation	-5.35729	-2.052	10/63
7_Member	GO Biological Processes	GO:0046635	positive regulation of alpha-beta T cell activation	-5.35729	-2.052	10/63

7_Member	GO Biological Processes	GO:0046634	regulation of alpha-beta T cell activation	-5.13672	-1.934	12/97
7_Member	GO Biological Processes	GO:0050865	regulation of cell activation	-5.13459	-1.934	37/653
7_Member	GO Biological Processes	GO:0002694	regulation of leukocyte	-4.65037	-1.581	34/608
7_Member	GO Biological Processes	GO:0046638	positive regulation of alpha-beta T cell differentiation	-4.62653	-1.581	8/47
7_Member	GO Biological Processes	GO:1903708	positive regulation of	-4.55782	-1.550	17/207
7_Member	GO Biological Processes	GO:0045580	regulation of T cell	-4.46251	-1.531	14/150
7_Member	GO Biological Processes	GO:0050867	positive regulation of cell activation	-4.3941	-1.495	26/421
7_Member	GO Biological Processes	GO:0002696	positive regulation of leukocyte activation	-4.2281	-1.359	25/406
7_Member	GO Biological Processes	GO:0045619	regulation of lymphocyte differentiation	-4.13744	-1.341	15/181
7_Member	GO Biological Processes	GO:1902107	positive regulation of leukocyte differentiation	-4.12519	-1.341	14/161
7_Member	GO Biological Processes	GO:0051249	regulation of lymphocyte	-4.1075	-1.341	29/515
7_Member	GO Biological Processes	GO:0045621	positive regulation of lymphocyte differentiation	-4.08092	-1.341	11/105
7_Member	GO Biological Processes	GO:0046631	alpha-beta T cell activation	-3.97674	-1.264	13/146
7_Member	GO Biological Processes	GO:0045582	positive regulation of T cell differentiation	-3.89559	-1.193	10/92
7_Member	GO Biological Processes	GO:0050863	regulation of T cell activation	-3.80744	-1.184	21/332
7_Member	GO Biological Processes	GO:0070661	leukocyte proliferation	-3.69437	-1.146	20/314
7_Member	GO Biological Processes	GO:0046649	lymphocyte activation	-3.67568	-1.146	37/767
7_Member	GO Biological Processes	GO:1902105	regulation of leukocyte differentiation	-3.65634	-1.137	19/292
7_Member	GO Biological Processes	GO:0046637	regulation of alpha-beta T cell differentiation	-3.64443	-1.137	8/64
7_Member	GO Biological Processes	GO:0030217	T cell differentiation	-3.49404	-1.063	17/253
7_Member	GO Biological Processes	GO:0022409	positive regulation of cell-cell adhesion	-3.4507	-1.029	18/279
7_Member	GO Biological Processes	GO:1903037	regulation of leukocyte cell-cell adhesion	-3.43125	-1.016	20/329
7_Member	GO Biological Processes	GO:1903039	positive regulation of leukocyte cell-cell adhesion	-3.38577	-1.001	16/235
7_Member	GO Biological Processes	GO:0051251	positive regulation of lymphocyte activation	-3.38356	-1.001	21/357
7_Member	GO Biological Processes	GO:0030155	regulation of cell adhesion	-3.37368	-1.001	35/738
7_Member	GO Biological Processes	GO:0050870	positive regulation of T cell activation	-3.342	-0.978	15/214
7_Member	GO Biological Processes	GO:0032943	mononuclear cell proliferation	-3.30487	-0.968	18/287
7_Member	GO Biological Processes	GO:0070663	regulation of leukocyte proliferation	-3.28567	-0.965	16/240
7_Member	GO Biological Processes	GO:0007159	leukocyte cell-cell adhesion	-3.2736	-0.960	21/364
7_Member	GO Biological Processes	GO:1903706	regulation of hemopoiesis	-3.21906	-0.944	26/500
7_Member	GO Biological Processes	GO:0030098	lymphocyte differentiation	-3.21235	-0.944	21/368
7_Member	GO Biological Processes	GO:0032944	regulation of mononuclear cell proliferation	-3.19644	-0.933	15/221
7_Member	GO Biological Processes	GO:0045785	positive regulation of cell	-3.10385	-0.882	23/428
7_Member	GO Biological Processes	GO:0042110	T cell activation	-3.07661	-0.876	25/484
7_Member	GO Biological Processes	GO:0002521	leukocyte differentiation	-3.07646	-0.876	27/539
7_Member	GO Biological Processes	GO:0022407	regulation of cell-cell adhesion	-2.93147	-0.810	23/441
7_Member	GO Biological Processes	GO:0046651	lymphocyte proliferation	-2.93108	-0.810	17/284
7_Member	GO Biological Processes	GO:0046632	alpha-beta T cell differentiation	-2.77993	-0.732	9/106
7_Member	GO Biological Processes	GO:0050670	regulation of lymphocyte proliferation	-2.77423	-0.729	14/219
7_Member	GO Biological Processes	GO:2000516	positive regulation of CD4-positive, alpha-beta T cell [illegible]	-2.66783	-0.692	5/36
7_Member	GO Biological Processes	GO:0035710	CD4-positive, alpha-beta T cell activation	-2.45071	-0.583	8/97
7_Member	GO Biological Processes	GO:0042129	regulation of T cell	-2.39281	-0.548	11/167
7_Member	GO Biological Processes	GO:0042098	T cell proliferation	-2.30675	-0.498	12/196

7_Member	GO Biological Processes	GO:2000514	regulation of CD4-positive, alpha-beta T cell activation	-2.25793	-0.473	6/63
7_Member	GO Biological Processes	GO:0050671	positive regulation of lymphocyte proliferation	-2.06382	-0.398	9/136
7_Member	GO Biological Processes	GO:0032946	positive regulation of mononuclear cell proliferation	-2.04399	-0.396	9/137
8_Summary	GO Biological Processes	GO:0001819	positive regulation of cytokine production	-4.94481	-1.775	29/463
8_Member	GO Biological Processes	GO:0001819	positive regulation of cytokine production	-4.94481	-1.775	29/463
8_Member	GO Biological Processes	GO:0032652	regulation of interleukin-1 production	-4.49646	-1.541	12/112
8_Member	GO Biological Processes	GO:0032612	interleukin-1 production	-4.12932	-1.341	12/122
8_Member	GO Biological Processes	GO:0035456	response to interferon-beta	-3.84152	-1.184	6/32
8_Member	GO Biological Processes	GO:0035458	cellular response to interferon-	-3.59771	-1.117	5/23
8_Member	GO Biological Processes	GO:0032732	positive regulation of interleukin-1 production	-3.07125	-0.876	7/60
8_Member	GO Biological Processes	GO:0032651	regulation of interleukin-1 beta production	-3.02102	-0.852	9/98
8_Member	GO Biological Processes	GO:0032611	interleukin-1 beta production	-2.75156	-0.727	9/107
8_Member	GO Biological Processes	GO:0032731	positive regulation of interleukin-1 beta production	-2.63572	-0.684	6/53
8_Member	GO Biological Processes	GO:0031349	positive regulation of defense response	-2.56408	-0.648	25/528
9_Summary	GO Biological Processes	GO:0018209	peptidyl-serine modification	-4.70196	-1.586	23/333
9_Member	GO Biological Processes	GO:0018209	peptidyl-serine modification	-4.70196	-1.586	23/333
9_Member	GO Biological Processes	GO:0018105	peptidyl-serine phosphorylation	-3.76798	-1.178	20/310
9_Member	GO Biological Processes	GO:0033135	regulation of peptidyl-serine phosphorylation	-3.42133	-1.016	12/145
9_Member	GO Biological Processes	GO:0033138	positive regulation of peptidyl-serine phosphorylation	-2.69591	-0.692	9/109
9_Member	GO Biological Processes	GO:0018210	peptidyl-threonine modification	-2.27482	-0.480	9/126
10_Summary	GO Biological Processes	GO:0007610	behavior	-4.63589	-1.581	34/609
10_Member	GO Biological Processes	GO:0007610	behavior	-4.63589	-1.581	34/609
10_Member	GO Biological Processes	GO:0007612	learning	-2.36173	-0.539	10/145
10_Member	GO Biological Processes	GO:0050806	positive regulation of synaptic transmission	-2.29699	-0.492	11/172
10_Member	GO Biological Processes	GO:0042391	regulation of membrane potential	-2.2481	-0.467	21/443
10_Member	GO Biological Processes	GO:0060291	long-term synaptic potentiation	-2.14864	-0.430	7/87
10_Member	GO Biological Processes	GO:0050804	modulation of chemical synaptic transmission	-2.13184	-0.425	21/454
10_Member	GO Biological Processes	GO:0099177	regulation of trans-synaptic signaling	-2.12154	-0.422	21/455
10_Member	GO Biological Processes	GO:1900271	regulation of long-term synaptic potentiation	-2.11991	-0.422	5/48
10_Member	GO Biological Processes	GO:0007611	learning or memory	-2.11351	-0.419	14/260
11_Summary	GO Biological Processes	GO:0032663	regulation of interleukin-2 production	-4.48955	-1.541	8/49
11_Member	GO Biological Processes	GO:0032663	regulation of interleukin-2 production	-4.48955	-1.541	8/49
11_Member	GO Biological Processes	GO:0032623	interleukin-2 production	-4.17548	-1.341	8/54
11_Member	GO Biological Processes	GO:0032743	positive regulation of interleukin-2 production	-2.3857	-0.547	4/26
11_Member	Canonical Pathways	M128	PID TNF PATHWAY	-2.19838	-0.457	5/46
12_Summary	GO Biological Processes	GO:0008285	negative regulation of cell proliferation	-4.40192	-1.495	40/788
12_Member	GO Biological Processes	GO:0008285	negative regulation of cell proliferation	-4.40192	-1.495	40/788
13_Summary	GO Biological Processes	GO:0007163	establishment or maintenance of cell polarity	-4.17524	-1.341	17/222
13_Member	GO Biological Processes	GO:0007163	establishment or maintenance of cell polarity	-4.17524	-1.341	17/222
13_Member	GO Biological Processes	GO:0030010	establishment of cell polarity	-4.06872	-1.341	13/143

14_Summary	Reactome Gene Sets	R-HSA-8877330	RUNX1 and FOXP3 control the development of regulatory T lymphocytes (Tregs)	-4.09635	-1.341	4/10
14_Member	Reactome Gene Sets	R-HSA-8877330	RUNX1 and FOXP3 control the development of regulatory T lymphocytes (Tregs)	-4.09635	-1.341	4/10
14_Member	GO Biological Processes	GO:0002683	negative regulation of immune system process	-4.06449	-1.341	28/492
14_Member	GO Biological Processes	GO:0002695	negative regulation of leukocyte activation	-3.00407	-0.845	13/184
14_Member	GO Biological Processes	GO:0050869	negative regulation of B cell activation	-2.9035	-0.800	5/32
14_Member	GO Biological Processes	GO:0070664	negative regulation of leukocyte proliferation	-2.77956	-0.732	8/86
14_Member	GO Biological Processes	GO:0045066	regulatory T cell differentiation	-2.72361	-0.712	5/35
14_Member	GO Biological Processes	GO:0051250	negative regulation of lymphocyte activation	-2.66374	-0.692	11/154
14_Member	GO Biological Processes	GO:0050866	negative regulation of cell activation	-2.54881	-0.647	13/207
14_Member	GO Biological Processes	GO:0032945	negative regulation of mononuclear cell proliferation	-2.34694	-0.527	7/80
14_Member	GO Biological Processes	GO:0007162	negative regulation of cell	-2.28598	-0.486	16/301
14_Member	GO Biological Processes	GO:0045589	regulation of regulatory T cell differentiation	-2.05715	-0.398	4/32
14_Member	GO Biological Processes	GO:0030889	negative regulation of B cell proliferation	-2.05704	-0.398	3/17
15_Summary	GO Biological Processes	GO:0060541	respiratory system development	-4.08711	-1.341	16/204
15_Member	GO Biological Processes	GO:0060541	respiratory system development	-4.08711	-1.341	16/204
15_Member	GO Biological Processes	GO:0030324	lung development	-3.6884	-1.146	14/177
15_Member	GO Biological Processes	GO:0030323	respiratory tube development	-3.58796	-1.117	14/181
16_Summary	GO Biological Processes	GO:0080135	regulation of cellular response to stress	-3.86751	-1.184	38/778
16_Member	GO Biological Processes	GO:0080135	regulation of cellular response to stress	-3.86751	-1.184	38/778
16_Member	GO Biological Processes	GO:2001020	regulation of response to DNA damage stimulus	-3.57444	-1.109	16/226
16_Member	GO Biological Processes	GO:0008630	intrinsic apoptotic signaling pathway in response to DNA damage	-3.49021	-1.063	10/103
16_Member	GO Biological Processes	GO:0097193	intrinsic apoptotic signaling pathway	-3.25185	-0.960	18/290
16_Member	GO Biological Processes	GO:2001242	regulation of intrinsic apoptotic signaling pathway	-2.41251	-0.552	11/166
16_Member	GO Biological Processes	GO:2001021	negative regulation of response to DNA damage stimulus	-2.25915	-0.473	7/83
16_Member	GO Biological Processes	GO:0042771	intrinsic apoptotic signaling pathway in response to DNA damage by p53 class mediator	-2.23926	-0.465	5/45
16_Member	GO Biological Processes	GO:1902230	negative regulation of intrinsic apoptotic signaling pathway in response to DNA damage	-2.10639	-0.416	4/31
17_Summary	GO Biological Processes	GO:0001836	release of cytochrome c from mitochondria	-3.84339	-1.184	8/60
17_Member	GO Biological Processes	GO:0001836	release of cytochrome c from mitochondria	-3.84339	-1.184	8/60
17_Member	GO Biological Processes	GO:0090199	regulation of release of cytochrome c from mitochondria	-3.6184	-1.129	7/49
17_Member	GO Biological Processes	GO:0090200	positive regulation of release of cytochrome c from mitochondria	-3.17829	-0.925	5/28
17_Member	GO Biological Processes	GO:1905477	positive regulation of protein localization to membrane	-2.25262	-0.469	9/127
18_Summary	GO Biological Processes	GO:0048511	rhythmic process	-3.84313	-1.184	20/306
18_Member	GO Biological Processes	GO:0048511	rhythmic process	-3.84313	-1.184	20/306
18_Member	GO Biological Processes	GO:0042749	regulation of circadian sleep/wake cycle	-3.69493	-1.146	5/22

18_Member	GO Biological Processes	GO:0022410	circadian sleep/wake cycle	-3.41779	-1.016	5/25
18_Member	GO Biological Processes	GO:0007623	circadian rhythm	-3.25795	-0.960	15/218
18_Member	GO Biological Processes	GO:0042745	circadian sleep/wake cycle	-3.17829	-0.925	5/28
18_Member	GO Biological Processes	GO:0045187	regulation of circadian sleep/wake cycle, sleep	-3.00399	-0.845	4/18
18_Member	GO Biological Processes	GO:0048512	circadian behavior	-2.81349	-0.753	6/49
18_Member	GO Biological Processes	GO:0030431	sleep	-2.7814	-0.732	5/34
18_Member	GO Biological Processes	GO:0007622	rhythmic behavior	-2.76736	-0.727	6/50
18_Member	GO Biological Processes	GO:0050802	circadian sleep/wake cycle, sleep	-2.66088	-0.692	4/22
18_Member	GO Biological Processes	GO:0042752	regulation of circadian rhythm	-2.36626	-0.540	9/122
18_Member	GO Biological Processes	GO:0048520	positive regulation of behavior	-2.32486	-0.509	4/27
18_Member	GO Biological Processes	GO:0051932	synaptic transmission, GABAergic	-2.11991	-0.422	5/48
18_Member	GO Biological Processes	GO:0042753	positive regulation of circadian rhythm	-2.05704	-0.398	3/17
18_Member	GO Biological Processes	GO:0050795	regulation of behavior	-2.00846	-0.377	6/71
19_Summary	GO Biological Processes	GO:0050708	regulation of protein secretion	-3.82981	-1.184	24/405
19_Member	GO Biological Processes	GO:0050708	regulation of protein secretion	-3.82981	-1.184	24/405
19_Member	GO Biological Processes	GO:0002791	regulation of peptide secretion	-3.77369	-1.178	25/434
19_Member	GO Biological Processes	GO:1903532	positive regulation of secretion by cell	-3.21864	-0.944	20/342
19_Member	GO Biological Processes	GO:0051223	regulation of protein transport	-3.11496	-0.889	31/648
19_Member	GO Biological Processes	GO:0090087	regulation of peptide transport	-3.08897	-0.877	32/679
19_Member	GO Biological Processes	GO:0051046	regulation of secretion	-2.92871	-0.810	34/753
19_Member	GO Biological Processes	GO:0070201	regulation of establishment of protein localization	-2.92458	-0.809	32/695
19_Member	GO Biological Processes	GO:0051047	positive regulation of secretion	-2.74925	-0.727	20/374
19_Member	GO Biological Processes	GO:0009306	protein secretion	-2.68437	-0.692	25/517
19_Member	GO Biological Processes	GO:1903530	regulation of secretion by cell	-2.66827	-0.692	31/692
19_Member	GO Biological Processes	GO:0002793	positive regulation of peptide secretion	-2.6127	-0.671	14/228
19_Member	GO Biological Processes	GO:0002790	peptide secretion	-2.57141	-0.648	26/556
19_Member	GO Biological Processes	GO:0050714	positive regulation of protein secretion	-2.54881	-0.647	13/207
19_Member	GO Biological Processes	GO:0002792	negative regulation of peptide secretion	-2.38981	-0.547	9/121
19_Member	GO Biological Processes	GO:0051222	positive regulation of protein transport	-2.17062	-0.444	18/365
19_Member	GO Biological Processes	GO:0051048	negative regulation of secretion	-2.09169	-0.408	12/209
19_Member	GO Biological Processes	GO:1904951	positive regulation of establishment of protein [illegible]	-2.06177	-0.398	19/403
19_Member	GO Biological Processes	GO:0050709	negative regulation of protein secretion	-2.01165	-0.377	8/115
20_Summary	GO Biological Processes	GO:0120179	adherens junction disassembly	-3.78959	-1.184	3/5
20_Member	GO Biological Processes	GO:0120179	adherens junction disassembly	-3.78959	-1.184	3/5
20_Member	GO Biological Processes	GO:0120180	cell-substrate adherens junction disassembly	-3.78959	-1.184	3/5
20_Member	GO Biological Processes	GO:0120181	focal adhesion disassembly	-3.78959	-1.184	3/5
20_Member	GO Biological Processes	GO:0120182	regulation of focal adhesion disassembly	-3.78959	-1.184	3/5
20_Member	GO Biological Processes	GO:0120183	positive regulation of focal adhesion disassembly	-3.78959	-1.184	3/5
20_Member	GO Biological Processes	GO:0051549	positive regulation of keratinocyte migration	-2.50567	-0.623	3/12
20_Member	GO Biological Processes	GO:0051547	regulation of keratinocyte	-2.30361	-0.498	3/14
20_Member	GO Biological Processes	GO:1903393	positive regulation of adherens junction organization	-2.10639	-0.416	4/31
