# Supplementary material for: JunB is a key regulator of multiple myeloma bone marrow angiogenesis
Source: Leukemia. 2021 May 18;35(12):3509–25. doi: 10.1038/s41375-021-01271-9 (PMC8632680; doi:10.1038/s41375-021-01271-9)
Supplement: Supplementary file 4 — Supplementary Table 6 (KEGG and GO enrichment analysis of JunBand its target AFs, and of each MCODE cluster in Supplementary Fig. 11B-D.) [file 41375_2021_1271_MOESM4_ESM.pdf]

GroupID	Category	Term	Description	LogP	Log (q-value)	InTerm_InList	Symbols
1_Summary	Reactome Gene Sets	R-HSA-9006934	Signaling by Receptor Tyrosine Kinases	-6.04391	-2.075	4/506	IGF1,JUNB,VEGFA,VEGFB
1_Member	Reactome Gene Sets	R-HSA-9006934	Signaling by Receptor Tyrosine Kinases	-6.04391	-2.075	4/506	IGF1,JUNB,VEGFA,VEGFB
1_Member	GO Biological Processes	GO:0002576	platelet degranulation	-5.85232	-2.075	3/128	IGF1,VEGFA,VEGFB
1_Member	Reactome Gene Sets	R-HSA-114608	Platelet degranulation	-5.84212	-2.075	3/129	IGF1,VEGFA,VEGFB
1_Member	Reactome Gene Sets	R-HSA-76005	Response to elevated platelet cytosolic Ca2+	-5.79233	-2.075	3/134	IGF1,VEGFA,VEGFB
1_Member	KEGG Pathway	hsa04510	Focal adhesion	-5.27562	-1.731	3/199	IGF1,VEGFA,VEGFB
1_Member	GO Biological Processes	GO:0050679	positive regulation of epithelial cell proliferation	-5.20548	-1.731	3/210	IGF1,VEGFA,VEGFB
1_Member	KEGG Pathway	hsa04015	Rap1 signaling pathway	-5.20548	-1.731	3/210	IGF1,VEGFA,VEGFB
1_Member	KEGG Pathway	hsa04014	Ras signaling pathway	-5.10404	-1.688	3/227	IGF1,VEGFA,VEGFB
1_Member	Reactome Gene Sets	R-HSA-76002	Platelet activation, signaling and aggregation	-4.91243	-1.548	3/263	IGF1,VEGFA,VEGFB
1_Member	KEGG Pathway	hsa04151	PI3K-Akt signaling pathway	-4.5712	-1.286	3/342	IGF1,VEGFA,VEGFB
1_Member	Canonical Pathways	M5883	NABA SECRETED FACTORS	-4.56364	-1.286	3/344	IGF1,VEGFA,VEGFB
1_Member	KEGG Pathway	hsa05200	Pathways in cancer	-4.38441	-1.208	3/395	IGF1,VEGFA,VEGFB
1_Member	GO Biological Processes	GO:0050678	regulation of epithelial cell proliferation	-4.38113	-1.208	3/396	IGF1,VEGFA,VEGFB

1_Member	GO Biological Processes	GO:0050673	epithelial cell proliferation	-4.20135	-1.058	3/455	IGF1,VEGFA,VEGFB
1_Member	GO Biological Processes	GO:0030335	positive regulation of cell migration	-3.93313	-0.818	3/560	IGF1,VEGFA,VEGFB
1_Member	GO Biological Processes	GO:2000147	positive regulation of cell motility	-3.87901	-0.804	3/584	IGF1,VEGFA,VEGFB
1_Member	GO Biological Processes	GO:0007507	heart development	-3.86802	-0.804	3/589	IGF1,VEGFA,VEGFB
1_Member	GO Biological Processes	GO:0051272	positive regulation of cellular component movement	-3.84203	-0.802	3/601	IGF1,VEGFA,VEGFB
1_Member	GO Biological Processes	GO:0040017	positive regulation of locomotion	-3.81866	-0.801	3/612	IGF1,VEGFA,VEGFB
1_Member	Reactome Gene Sets	R-HSA-109582	Hemostasis	-3.79778	-0.801	3/622	IGF1,VEGFA,VEGFB
1_Member	GO Biological Processes	GO:0007169	transmembrane receptor protein tyrosine kinase signaling pathway	-3.57956	-0.675	3/737	IGF1,VEGFA,VEGFB
1_Member	Canonical Pathways	M5885	NABA MATRISOME ASSOCIATED	-3.55197	-0.664	3/753	IGF1,VEGFA,VEGFB
1_Member	GO Biological Processes	GO:0045055	regulated exocytosis	-3.4807	-0.639	3/796	IGF1,VEGFA,VEGFB
2_Summary	GO Biological Processes	GO:0001701	in utero embryonic development	-4.41763	-1.208	3/385	IGF1,JUNB,VEGFA
2_Member	GO Biological Processes	GO:0001701	in utero embryonic development	-4.41763	-1.208	3/385	IGF1,JUNB,VEGFA
2_Member	GO Biological Processes	GO:0043009	chordate embryonic development	-3.74903	-0.772	3/646	IGF1,JUNB,VEGFA
2_Member	GO Biological Processes	GO:0009792	embryo development ending in birth or egg hatching	-3.71366	-0.756	3/664	IGF1,JUNB,VEGFA
2_Member	GO Biological Processes	GO:0003006	developmental process involved in reproduction	-3.67736	-0.743	3/683	IGF1,JUNB,VEGFA

3_Summary	GO Biological Processes	GO:0048514	blood vessel morphogenesis	-3.66425	-0.743	3/690	JUNB,VEGFA,VEGFB
3_Member	GO Biological Processes	GO:0048514	blood vessel morphogenesis	-3.66425	-0.743	3/690	JUNB,VEGFA,VEGFB
3_Member	GO Biological Processes	GO:0001568	blood vessel development	-3.5117	-0.655	3/777	JUNB,VEGFA,VEGFB
4_Summary	GO Biological Processes	GO:1901137	carbohydrate derivative biosynthetic process	-3.51666	-0.655	3/774	IGF1,PIGF,VEGFB
4_Member	GO Biological Processes	GO:1901137	carbohydrate derivative biosynthetic process	-3.51666	-0.655	3/774	IGF1,PIGF,VEGFB

MCODE Cluster 1

GO	Category	Description	LogP	#GeneInGO	#GeneInGOAndHitList	Hits	Log(q-value)
GO:0007204	GO Biological Processes	positive regulation of cytosolic calcium ion concentration	-17.642	322	10	ADCY5|APLNR|C3AR1|CCR5|DRD2|CXCR3|GPER1|CXCR2|CCL1|NMU	-13.9947
GO:0030595	GO Biological Processes	leukocyte chemotaxis	-9.5587	233	6	ANXA1|C3AR1|CCR5|CXCR3|CXCR2|CCL1	-6.46772
ko04062	KEGG Pathway	Chemokine signaling pathway	-8.07621	182	5	ADCY5|CCR5|CXCR3|CXCR2|CCL1	-5.03665
GO:1904018	GO Biological Processes	positive regulation of vasculature development	-7.52058	235	5	APLNR|ANXA1|C3AR1|GPER1|CXCR2	-4.55454

MCODE Cluster 2

GO	Category	Description	LogP	#GeneInGO	#GeneInGOAndHitList	Hits	Log(q-value)
GO:0002576	GO Biological Processes	platelet degranulation	-8.25479	129	5	CLU|IGF1|ORM1|VEGFA|MMRN1	-4.42738
GO:0045785	GO Biological Processes	positive regulation of cell adhesion	-5.66554	428	5	IGF1|IL2RA|IL10|ITPKB|VEGFA	-2.40973
GO:0019932	GO Biological Processes	second-messenger-mediated signaling	-5.53096	456	5	ADRA1B|GCG|GRM5|IGF1|VEGFA	-2.3608
GO:0045860	GO Biological Processes	positive regulation of protein kinase activity	-5.16941	541	5	CLU|GCG|GRM5|IGF1|VEGFA	-2.1019

MCODE Cluster 3

GO	Category	Description	LogP	#GeneInGO	#GeneInGOAndHitList	Hits	Log(q-value)
ko04060	KEGG Pathway	Cytokine-cytokine receptor interaction	-7.73058	270	6	IFNAR2|IFNGR2|IL10RA|VEGFC|IL23A|IL17F	-3.44565
ko04630	KEGG Pathway	Jak-STAT signaling pathway	-7.25953	156	5	IFNAR2|IFNGR2|IL10RA|MCL1|IL23A	-3.44565
hsa05200	KEGG Pathway	Pathways in cancer	-5.80108	575	6	IFNAR2|IFNGR2|JUN|VEGFC|IL23A|CYCS	-2.73358
GO:0032103	GO Biological Processes	positive regulation of response to external stimulus	-5.5922	339	5	ADORA2B|SERPINE1|VEGFC|IL23A|IL17F	-2.56477
GO:0002696	GO Biological Processes	positive regulation of leukocyte activation	-5.21065	406	5	ADORA2B|CTLA4|CD55|ITGAM|IL23A	-2.30799
GO:0050900	GO Biological Processes	leukocyte migration	-4.69975	518	5	CD44|ITGAM|SERPINE1|VEGFC|IL23A	-1.9098
