# Supplementary material for: JunB is a key regulator of multiple myeloma bone marrow angiogenesis
Source: Leukemia. 2021 May 18;35(12):3509–25. doi: 10.1038/s41375-021-01271-9 (PMC8632680; doi:10.1038/s41375-021-01271-9)
Supplement: Supplementary file 5 — Supplementary Table 7 (JASPAR analysis of putative JunB binding sites in the peak sequences fromChIP-seq results related to Fig. 4E.) [file 41375_2021_1271_MOESM5_ESM.pdf]

VEGF

Matrix ID	Name	Score	Relative score	Sequence ID	Start	End	Strand	Predicted sequence
MA1132.1	JUN::JUNB	4.12107	0.759722736	NC_000006.12:43767882-43768097	51	60	+	GTTGGCTTAT
MA0490.1	JUNB	-2.49437	0.757619687	NC_000006.12:43767882-43768097	87	97	+	GAGTGACTGGT
MA1137.1	FOSL1::JUNB	4.81772	0.755958332	NC_000006.12:43767882-43768097	159	171	+	TTTTTACTAAGAC
MA1137.1	FOSL1::JUNB	4.26797	0.743178438	NC_000006.12:43767882-43768097	14	26	+	TTGTAACACACCT
MA0490.1	JUNB	-3.65814	0.74257083	NC_000006.12:43767882-43768097	85	95	-	CAGTCACTCCG
MA1135.1	FOSB::JUNB	1.33875	0.736292029	NC_000006.12:43767882-43768097	160	169	+	TTTTACTAAG
MA0490.1	JUNB	-4.40179	0.732954473	NC_000006.12:43767882-43768097	16	26	-	AGGTGTGTTAC
MA0490.2	JUNB	1.69019	0.72790234	NC_000006.12:43767882-43768097	159	171	+	TTTTTACTAAGAC
MA1140.1	JUNB(var.2)	3.98047	0.726449566	NC_000006.12:43767882-43768097	11	20	+	TTGTTGTAAC
MA1132.1	JUN::JUNB	2.64066	0.72630277	NC_000006.12:43767882-43768097	15	24	+	TGTAACACAC
MA1140.1	JUNB(var.2)	3.91472	0.724830867	NC_000006.12:43767882-43768097	11	20	-	GTTACAACAA
MA1137.1	FOSL1::JUNB	3.38836	0.722730438	NC_000006.12:43767882-43768097	97	109	+	TGATGGCTATCCC
MA1132.1	JUN::JUNB	2.4561	0.72213633	NC_000006.12:43767882-43768097	204	213	+	GCTGAAGAAG
MA1134.1	FOS::JUNB	0.769282	0.721136515	NC_000006.12:43767882-43768097	14	25	-	GGTGTGTTACAA
MA0490.1	JUNB	-5.46936	0.719149526	NC_000006.12:43767882-43768097	14	24	+	TTGTAACACAC
MA1138.1	FOSL2::JUNB	0.293743	0.717062413	NC_000006.12:43767882-43768097	160	169	+	TTTTACTAAG
MA1135.1	FOSB::JUNB	0.280769	0.716993946	NC_000006.12:43767882-43768097	204	213	+	GCTGAAGAAG
MA1132.1	JUN::JUNB	2.2162	0.716720518	NC_000006.12:43767882-43768097	98	107	+	GATGGCTATC
MA1134.1	FOS::JUNB	0.490004	0.716251231	NC_000006.12:43767882-43768097	159	170	-	TCTTAGTAAAAA
MA0490.1	JUNB	-5.81269	0.714709922	NC_000006.12:43767882-43768097	161	171	-	GTCTTAGTAAA
MA1135.1	FOSB::JUNB	0.136294	0.714358668	NC_000006.12:43767882-43768097	161	170	-	TCTTAGTAAA
MA0490.2	JUNB	0.82096	0.712518057	NC_000006.12:43767882-43768097	87	99	+	GAGTGACTGGTGA
MA1137.1	FOSL1::JUNB	2.91884	0.711815616	NC_000006.12:43767882-43768097	203	215	+	GGCTGAAGAAGGC
MA1134.1	FOS::JUNB	0.22851	0.711677016	NC_000006.12:43767882-43768097	160	171	+	TTTTACTAAGAC
MA1138.1	FOSL2::JUNB	-0.191085	0.708244331	NC_000006.12:43767882-43768097	204	213	+	GCTGAAGAAG
MA1135.1	FOSB::JUNB	-0.205884	0.708117169	NC_000006.12:43767882-43768097	88	97	+	AGTGACTGGT
MA1132.1	JUN::JUNB	1.83192	0.708045513	NC_000006.12:43767882-43768097	188	197	+	GTTGGCTCTT
MA1134.1	FOS::JUNB	0.0207567	0.708042872	NC_000006.12:43767882-43768097	87	98	-	CACCAGTCACTC
MA1138.1	FOSL2::JUNB	-0.224047	0.707644815	NC_000006.12:43767882-43768097	88	97	+	AGTGACTGGT
MA1137.1	FOSL1::JUNB	2.70908	0.706939423	NC_000006.12:43767882-43768097	14	26	-	AGGTGTGTTACAA
MA1135.1	FOSB::JUNB	-0.273292	0.706887625	NC_000006.12:43767882-43768097	15	24	+	TGTAACACAC
MA1132.1	JUN::JUNB	1.69831	0.705029297	NC_000006.12:43767882-43768097	160	169	+	TTTTACTAAG

MA1132.1	JUN::JUNB	1.67511	0.704505499	NC_000006.12:43767882-43768097	161	170	-	TCTTAGTAAA
MA1132.1	JUN::JUNB	1.66457	0.704267675	NC_000006.12:43767882-43768097	16	25	-	GGTGTGTTAC
MA1135.1	FOSB::JUNB	-0.485494	0.703016966	NC_000006.12:43767882-43768097	16	25	-	GGTGTGTTAC
MA1133.1	JUN::JUNB(var.2)	2.86455	0.70297279	NC_000006.12:43767882-43768097	10	21	-	TGTTACAACAAC
MA1132.1	JUN::JUNB	1.59708	0.702744104	NC_000006.12:43767882-43768097	88	97	+	AGTGACTGGT
MA1139.1	FOSL2::JUNB(var.2)	1.14209	0.702423396	NC_000006.12:43767882-43768097	10	21	+	GTTGTTGTAACA
MA1137.1	FOSL1::JUNB	2.49899	0.702055587	NC_000006.12:43767882-43768097	87	99	+	GAGTGACTGGTGA
MA1139.1	FOSL2::JUNB(var.2)	1.11118	0.701884428	NC_000006.12:43767882-43768097	10	21	-	TGTTACAACAAC
MA1138.1	FOSL2::JUNB	-0.587508	0.701034143	NC_000006.12:43767882-43768097	161	170	-	TCTTAGTAAA

IGF1

Matrix ID	Name	Score	Relative score	Sequence ID	Start	End	Strand	Predicted sequence
MA0490.1	JUNB	15.5159	0.990513609	NC_000012.12:c102475036-102474456	350	360	-	GAATGACTCAT
MA0490.2	JUNB	16.1308	0.983483241	NC_000012.12:c102475036-102474456	348	360	-	GAATGACTCATGC
MA1135.1	FOSB::JUNB	14.6617	0.979309151	NC_000012.12:c102475036-102474456	431	440	+	GATGACTCAC
MA1132.1	JUN::JUNB	13.785	0.977884343	NC_000012.12:c102475036-102474456	350	359	-	AATGACTCAT
MA1132.1	JUN::JUNB	13.7757	0.977674607	NC_000012.12:c102475036-102474456	431	440	+	GATGACTCAC
MA1138.1	FOSL2::JUNB	14.6138	0.977516485	NC_000012.12:c102475036-102474456	431	440	+	GATGACTCAC
MA0490.1	JUNB	14.4184	0.976322622	NC_000012.12:c102475036-102474456	430	440	+	TGATGACTCAC
MA1138.1	FOSL2::JUNB	14.4653	0.974816291	NC_000012.12:c102475036-102474456	350	359	-	AATGACTCAT
MA1134.1	FOS::JUNB	15.112	0.972028594	NC_000012.12:c102475036-102474456	430	441	-	TGTGAGTCATCA
MA1137.1	FOSL1::JUNB	14.0502	0.97058286	NC_000012.12:c102475036-102474456	430	442	+	TGATGACTCACAC
MA0490.1	JUNB	13.772	0.967963608	NC_000012.12:c102475036-102474456	432	442	-	GTGTGAGTCAT
MA1135.1	FOSB::JUNB	14.0125	0.967466994	NC_000012.12:c102475036-102474456	350	359	-	AATGACTCAT
MA1134.1	FOS::JUNB	14.6496	0.96393931	NC_000012.12:c102475036-102474456	349	360	+	CATGAGTCATTC
MA1135.1	FOSB::JUNB	13.6637	0.961105733	NC_000012.12:c102475036-102474456	349	358	+	CATGAGTCAT
MA1138.1	FOSL2::JUNB	13.6374	0.959758718	NC_000012.12:c102475036-102474456	349	358	+	CATGAGTCAT
MA1138.1	FOSL2::JUNB	13.4001	0.955442043	NC_000012.12:c102475036-102474456	432	441	-	TGTGAGTCAT
MA1137.1	FOSL1::JUNB	13.397	0.955398946	NC_000012.12:c102475036-102474456	348	360	-	GAATGACTCATGC
MA1134.1	FOS::JUNB	14.1487	0.955176447	NC_000012.12:c102475036-102474456	431	442	+	GATGACTCACAC
MA1135.1	FOSB::JUNB	13.3316	0.955047952	NC_000012.12:c102475036-102474456	432	441	-	TGTGAGTCAT
MA0490.1	JUNB	12.6449	0.953388907	NC_000012.12:c102475036-102474456	348	358	+	GCATGAGTCAT
MA1134.1	FOS::JUNB	13.6703	0.946808337	NC_000012.12:c102475036-102474456	348	359	-	AATGACTCATGC
MA0490.2	JUNB	13.8661	0.943401031	NC_000012.12:c102475036-102474456	430	442	+	TGATGACTCACAC
MA0490.2	JUNB	13.5107	0.937110364	NC_000012.12:c102475036-102474456	348	360	+	GCATGAGTCATTC
MA1137.1	FOSL1::JUNB	11.3462	0.907724676	NC_000012.12:c102475036-102474456	430	442	-	GTGTGAGTCATCA
MA1137.1	FOSL1::JUNB	11.1925	0.90415025	NC_000012.12:c102475036-102474456	348	360	+	GCATGAGTCATTC
MA1132.1	JUN::JUNB	10.3886	0.901212783	NC_000012.12:c102475036-102474456	349	358	+	CATGAGTCAT
MA0490.2	JUNB	11.3725	0.899266707	NC_000012.12:c102475036-102474456	430	442	-	GTGTGAGTCATCA
MA1132.1	JUN::JUNB	9.68411	0.885307795	NC_000012.12:c102475036-102474456	432	441	-	TGTGAGTCAT
MA1136.1	FOSB::JUNB(var.2)	8.80432	0.849059269	NC_000012.12:c102475036-102474456	59	68	-	GTGATATCAT
MA1139.1	FOSL2::JUNB(var.2)	9.40732	0.846550511	NC_000012.12:c102475036-102474456	58	69	-	GGTGATATCATT
MA1139.1	FOSL2::JUNB(var.2)	9.38453	0.846153039	NC_000012.12:c102475036-102474456	58	69	+	AATGATATCACC
MA1133.1	JUN::JUNB(var.2)	9.50516	0.84088166	NC_000012.12:c102475036-102474456	58	69	-	GGTGATATCATT

MA1136.1	FOSB::JUNB(var.2)	8.07561	0.834339888	NC_000012.12:c102475036-102474456	59	68	+	ATGATATCAC
MA1140.1	JUNB(var.2)	7.78452	0.820099296	NC_000012.12:c102475036-102474456	59	68	-	GTGATATCAT
MA1140.1	JUNB(var.2)	7.76371	0.819587007	NC_000012.12:c102475036-102474456	59	68	+	ATGATATCAC
MA0490.1	JUNB	2.17992	0.818063922	NC_000012.12:c102475036-102474456	203	213	+	CAATGATTCCT
MA0490.1	JUNB	2.00434	0.815793425	NC_000012.12:c102475036-102474456	551	561	+	AGATCAGTAAG
MA0490.1	JUNB	1.98583	0.815554121	NC_000012.12:c102475036-102474456	96	106	-	AAGTGATTCTG
MA1132.1	JUN::JUNB	6.3186	0.809331765	NC_000012.12:c102475036-102474456	99	108	+	AATCACTTAT
MA1132.1	JUN::JUNB	5.92385	0.800420495	NC_000012.12:c102475036-102474456	223	232	+	GATCACTTAA

FOXO3

Matrix ID	Name	Score	Relative score	Sequence ID	Start	End	Strand	Predicted sequence
MA1135.1	FOSB::JUNB	13.107	0.950950624	NC_000006.12:108564265-108564583	8	17	+	TATGACTAAT
MA1138.1	FOSL2::JUNB	13.0539	0.949145638	NC_000006.12:108564265-108564583	8	17	+	TATGACTAAT
MA1137.1	FOSL1::JUNB	13.0888	0.948233114	NC_000006.12:108564265-108564583	7	19	+	TTATGACTAATAG
MA1132.1	JUN::JUNB	12.0539	0.938806509	NC_000006.12:108564265-108564583	8	17	+	TATGACTAAT
MA1134.1	FOS::JUNB	13.1929	0.938456991	NC_000006.12:108564265-108564583	7	18	-	TATTAGTCATAA
MA1137.1	FOSL1::JUNB	12.6169	0.937262978	NC_000006.12:108564265-108564583	135	147	-	AAATGACTAATCA
MA0490.1	JUNB	11.177	0.934406264	NC_000006.12:108564265-108564583	137	147	-	AAATGACTAAT
MA1138.1	FOSL2::JUNB	12.2116	0.933825402	NC_000006.12:108564265-108564583	137	146	-	AATGACTAAT
MA1132.1	JUN::JUNB	11.7034	0.930893962	NC_000006.12:108564265-108564583	137	146	-	AATGACTAAT
MA1135.1	FOSB::JUNB	11.9762	0.930323634	NC_000006.12:108564265-108564583	137	146	-	AATGACTAAT
MA0490.2	JUNB	12.9257	0.926755716	NC_000006.12:108564265-108564583	135	147	-	AAATGACTAATCA
MA0490.1	JUNB	10.3058	0.923141389	NC_000006.12:108564265-108564583	7	17	+	TTATGACTAAT
MA1134.1	FOS::JUNB	12.1755	0.920661514	NC_000006.12:108564265-108564583	136	147	+	GATTAGTCATTT
MA0490.2	JUNB	12.3463	0.916501751	NC_000006.12:108564265-108564583	7	19	+	TTATGACTAATAG
MA1135.1	FOSB::JUNB	9.66741	0.888210754	NC_000006.12:108564265-108564583	136	145	+	GATTAGTCAT
MA1138.1	FOSL2::JUNB	9.40618	0.882800287	NC_000006.12:108564265-108564583	136	145	+	GATTAGTCAT
MA1134.1	FOS::JUNB	9.91751	0.881162763	NC_000006.12:108564265-108564583	8	19	+	TATGACTAATAG
MA1134.1	FOS::JUNB	9.75661	0.878348186	NC_000006.12:108564265-108564583	135	146	-	AATGACTAATCA
MA1135.1	FOSB::JUNB	9.04035	0.876772833	NC_000006.12:108564265-108564583	187	196	+	TCTGAGTAAT
MA1135.1	FOSB::JUNB	9.01469	0.876304739	NC_000006.12:108564265-108564583	9	18	-	TATTAGTCAT
MA0490.2	JUNB	10.0507	0.875872279	NC_000006.12:108564265-108564583	135	147	+	TGATTAGTCATTT
MA1138.1	FOSL2::JUNB	8.86388	0.872936829	NC_000006.12:108564265-108564583	9	18	-	TATTAGTCAT
MA1138.1	FOSL2::JUNB	8.74434	0.870762707	NC_000006.12:108564265-108564583	187	196	+	TCTGAGTAAT
MA0490.1	JUNB	6.00921	0.867581209	NC_000006.12:108564265-108564583	135	145	+	TGATTAGTCAT
MA0490.1	JUNB	5.88253	0.865943019	NC_000006.12:108564265-108564583	186	196	+	TTCTGAGTAAT
MA0490.2	JUNB	9.40945	0.864523317	NC_000006.12:108564265-108564583	7	19	-	CTATTAGTCATAA
MA0490.2	JUNB	9.03933	0.857972716	NC_000006.12:108564265-108564583	186	198	-	ACATTACTCAGAA
MA1137.1	FOSL1::JUNB	9.11837	0.855933996	NC_000006.12:108564265-108564583	7	19	-	CTATTAGTCATAA
MA1137.1	FOSL1::JUNB	9.09435	0.85537554	NC_000006.12:108564265-108564583	135	147	+	TGATTAGTCATTT
MA1132.1	JUN::JUNB	8.28004	0.853611148	NC_000006.12:108564265-108564583	89	98	+	TGTGACCCAT
MA0490.1	JUNB	4.82724	0.852296972	NC_000006.12:108564265-108564583	88	98	+	TTGTGACCCAT
MA1135.1	FOSB::JUNB	7.50096	0.848693634	NC_000006.12:108564265-108564583	89	98	+	TGTGACCCAT

MA1134.1	FOS::JUNB	7.97161	0.847123866	NC_000006.12:108564265-108564583	186	197	-	CATTACTCAGAA
MA1138.1	FOSL2::JUNB	7.38592	0.846055597	NC_000006.12:108564265-108564583	89	98	+	TGTGACCCAT
MA1132.1	JUN::JUNB	7.9218	0.845523968	NC_000006.12:108564265-108564583	136	145	+	GATTAGTCAT
MA1135.1	FOSB::JUNB	7.2662	0.844411443	NC_000006.12:108564265-108564583	188	197	-	CATTACTCAG
MA1137.1	FOSL1::JUNB	8.5595	0.842942181	NC_000006.12:108564265-108564583	186	198	+	TTCTGAGTAATGT
MA0490.1	JUNB	3.98191	0.841365776	NC_000006.12:108564265-108564583	9	19	-	CTATTAGTCAT
MA0490.1	JUNB	3.84579	0.839605606	NC_000006.12:108564265-108564583	90	100	-	AAATGGGTCAC
MA1137.1	FOSL1::JUNB	8.18597	0.834258808	NC_000006.12:108564265-108564583	88	100	+	TTGTGACCCATTT
MA1138.1	FOSL2::JUNB	6.72962	0.834118812	NC_000006.12:108564265-108564583	188	197	-	CATTACTCAG
MA1134.1	FOS::JUNB	7.19632	0.833562141	NC_000006.12:108564265-108564583	88	99	-	AATGGGTCACAA
MA1134.1	FOS::JUNB	7.19449	0.833530136	NC_000006.12:108564265-108564583	187	198	+	TCTGAGTAATGT
MA0490.1	JUNB	3.30847	0.832657358	NC_000006.12:108564265-108564583	188	198	-	ACATTACTCAG
MA1137.1	FOSL1::JUNB	8.09367	0.832113266	NC_000006.12:108564265-108564583	186	198	-	ACATTACTCAGAA
MA1132.1	JUN::JUNB	7.29265	0.831320855	NC_000006.12:108564265-108564583	9	18	-	TATTAGTCAT
MA1132.1	JUN::JUNB	7.13	0.827649175	NC_000006.12:108564265-108564583	187	196	+	TCTGAGTAAT
MA0490.2	JUNB	7.22591	0.825877435	NC_000006.12:108564265-108564583	186	198	+	TTCTGAGTAATGT
MA0490.2	JUNB	6.78659	0.818102065	NC_000006.12:108564265-108564583	88	100	+	TTGTGACCCATTT
MA1138.1	FOSL2::JUNB	5.29457	0.808017912	NC_000006.12:108564265-108564583	90	99	-	AATGGGTCAC
MA1132.1	JUN::JUNB	6.25011	0.807785632	NC_000006.12:108564265-108564583	188	197	-	CATTACTCAG
MA1135.1	FOSB::JUNB	5.12983	0.805443116	NC_000006.12:108564265-108564583	90	99	-	AATGGGTCAC
MA1134.1	FOS::JUNB	5.53535	0.804507479	NC_000006.12:108564265-108564583	89	100	+	TGTGACCCATTT

TRAF2

Matrix ID	Name	Score	Relative score	Sequence ID	Start	End	Strand	Predicted sequence
MA0490.1	JUNB	14.9192	0.98279805	NC_000009.12:136882569-136882916	143	153	-	GGGTGACTCAC
MA0490.1	JUNB	13.5379	0.96493557	NC_000009.12:136882569-136882916	141	151	+	GAGTGAGTCAC
MA1135.1	FOSB::JUNB	13.7803	0.96323167	NC_000009.12:136882569-136882916	143	152	-	GGTGACTCAC
MA1138.1	FOSL2::JUNB	13.6476	0.95994294	NC_000009.12:136882569-136882916	143	152	-	GGTGACTCAC
MA1134.1	FOS::JUNB	13.3306	0.94086707	NC_000009.12:136882569-136882916	142	153	+	AGTGAGTCACCC
MA1132.1	JUN::JUNB	12.0866	0.93954481	NC_000009.12:136882569-136882916	143	152	-	GGTGACTCAC
MA1134.1	FOS::JUNB	12.4605	0.92564687	NC_000009.12:136882569-136882916	141	152	-	GGTGACTCACTC
MA1137.1	FOSL1::JUNB	11.9075	0.92077136	NC_000009.12:136882569-136882916	141	153	-	GGGTGACTCACTC
MA1138.1	FOSL2::JUNB	11.3216	0.91763827	NC_000009.12:136882569-136882916	142	151	+	AGTGAGTCAC
MA1135.1	FOSB::JUNB	11.0665	0.91373011	NC_000009.12:136882569-136882916	142	151	+	AGTGAGTCAC
MA0490.2	JUNB	11.351	0.89888557	NC_000009.12:136882569-136882916	141	153	-	GGGTGACTCACTC
MA1137.1	FOSL1::JUNB	10.0784	0.87825235	NC_000009.12:136882569-136882916	141	153	+	GAGTGAGTCACCC
MA1132.1	JUN::JUNB	8.34466	0.85506987	NC_000009.12:136882569-136882916	142	151	+	AGTGAGTCAC
MA0490.2	JUNB	8.8275	0.85422354	NC_000009.12:136882569-136882916	141	153	+	GAGTGAGTCACCC

CLU

Matrix ID	Name	Score	Relative score	Sequence ID	Start	End	Strand	Predicted sequence
MA1135.1	FOSB::JUNB	13.4597	0.957383433	NC_000008.11:c27614985-27614632	198	207	-	CATGACTCAC
MA1138.1	FOSL2::JUNB	13.3426	0.954396178	NC_000008.11:c27614985-27614632	198	207	-	CATGACTCAC
MA0490.1	JUNB	12.5822	0.952578007	NC_000008.11:c27614985-27614632	196	206	+	GCGTGAGTCAT
MA0490.1	JUNB	12.0251	0.945373935	NC_000008.11:c27614985-27614632	198	208	-	GCATGACTCAC
MA1135.1	FOSB::JUNB	12.7823	0.945028249	NC_000008.11:c27614985-27614632	197	206	+	CGTGAGTCAT
MA1134.1	FOS::JUNB	13.4756	0.943402574	NC_000008.11:c27614985-27614632	197	208	+	CGTGAGTCATGC
MA1138.1	FOSL2::JUNB	12.6712	0.942185195	NC_000008.11:c27614985-27614632	197	206	+	CGTGAGTCAT
MA1132.1	JUN::JUNB	12.162	0.941246682	NC_000008.11:c27614985-27614632	198	207	-	CATGACTCAC
MA1134.1	FOS::JUNB	12.4321	0.925149003	NC_000008.11:c27614985-27614632	196	207	-	CATGACTCACGC
MA0490.2	JUNB	12.8159	0.924812897	NC_000008.11:c27614985-27614632	196	208	-	GCATGACTCACGC
MA1137.1	FOSL1::JUNB	11.5908	0.913409038	NC_000008.11:c27614985-27614632	196	208	-	GCATGACTCACGC
MA0490.2	JUNB	10.7742	0.888677629	NC_000008.11:c27614985-27614632	196	208	+	GCGTGAGTCATGC
MA1137.1	FOSL1::JUNB	10.2734	0.882783798	NC_000008.11:c27614985-27614632	196	208	+	GCGTGAGTCATGC
MA1132.1	JUN::JUNB	8.69961	0.863082983	NC_000008.11:c27614985-27614632	197	206	+	CGTGAGTCAT
